# Cortical atrophy in chronic subdural hematoma from ultra-structures to physical properties

**DOI:** 10.1038/s41598-023-30135-8

**Published:** 2023-02-28

**Authors:** Pietro Familiari, Pierfrancesco Lapolla, Michela Relucenti, Ezio Battaglione, Loredana Cristiano, Veronica Sorrentino, Sara Aversa, Alessia D’Amico, Pierfabrizio Puntorieri, Lucia Bruzzaniti, Andrea Mingoli, Gioia Brachini, Giuseppe Barbaro, Anthony Kevin Scafa, Giancarlo D’Andrea, Alessandro Frati, Veronica Picotti, Luigi Valentino Berra, Vincenzo Petrozza, Stefania Nottola, Antonio Santoro, Placido Bruzzaniti

**Affiliations:** 1 Department of Human Neuroscience, Sapienza University of Rome, Rome, Italy; 2 Nuffield Department of Surgical Sciences, University of Oxford, John Radcliffe Oxford University Hospital, Headington, Oxford, OX3 9DU UK; 3 Department of Anatomical, Histological, Medical Legal Sciences and Locomotor Apparatus, Sapienza University of Rome, Rome, Italy; 4 Department of Surgery “Pietro Valdoni”, Sapienza University of Rome, Rome, Italy; 5 Department of Medico-Surgical Sciences and Biotechnologies, Sapienza University of Rome, Rome, Italy; 6 Department of Life, Health and Environmental Sciences, University of L’Aquila, L’Aquila, Italy; 7 Department of Experimental Medicine, Sapienza, University of Rome, Rome, Italy; 8 Unit of Rehabilitation, Istituto Neurotraumatologico Italiano, Rome, Italy; 9 DICEAM Department, University Mediterranea of Reggio Calabria, Reggio Calabria, Italy; 10 Neurosurgery Division of “Spaziani” Hospital, Frosinone, Italy; 11 Department of Neurosurgery, IRCCS Neuromed Pozzilli IS, Isernia, Italy; 12 Division of Neurosurgery, Policlinico Tor Vergata, University Tor Vergata of Rome, Rome, Italy

**Keywords:** Electron microscopy, Brain, Inflammation, Biomedical engineering, Brain injuries, Blood-brain barrier, Neuro-vascular interactions, Neural ageing

## Abstract

Several theories have tried to elucidate the mechanisms behind the pathophysiology of chronic subdural hematoma (CSDH). However, this process is complex and remains mostly unknown. In this study we performed a retrospective randomised analysis comparing the cortical atrophy of 190 patients with unilateral CSDH, with 190 healthy controls. To evaluate the extent of cortical atrophy, CT scan images were utilised to develop an index that is the ratio of the maximum diameter sum of 3 cisterns divided by the maximum diameter of the skull at the temporal lobe level. Also, we reported, for the first time, the ultrastructural analyses of the CSDH using a combination of immunohistochemistry methods and transmission electron microscopy techniques. Internal validation was performed to confirm the assessment of the different degrees of cortical atrophy. Relative Cortical Atrophy Index (RCA index) refers to the sum of the maximum diameter of three cisterns (insular cistern, longitudinal cerebral fissure and cerebral sulci greatest) with the temporal bones' greatest internal distance. This index, strongly related to age in healthy controls, is positively correlated to the preoperative and post-operative maximum diameter of hematoma and the midline shift in CSDH patients. On the contrary, it negatively correlates to the Karnofsky Performance Status (KPS). The Area Under the Receiver Operating Characteristics (AUROC) showed that RCA index effectively differentiated cases from controls. Immunohistochemistry analysis showed that the newly formed CD-31 positive microvessels are higher in number than the CD34-positive microvessels in the CSDH inner membrane than in the outer membrane. Ultrastructural observations highlight the presence of a chronic inflammatory state mainly in the CSDH inner membrane. Integrating these results, we have obtained an etiopathogenetic model of CSDH. Cortical atrophy appears to be the triggering factor activating the cascade of transendothelial cellular filtration, inflammation, membrane formation and neovascularisation leading to the CSDH formation.

## Introduction

Age-related physiological cortical atrophy, known as Normal Brain Ageing (NBA), results in changes in brain structure without clinical changes in neurological status^1^.

Chronic Subdural Hematoma (CSDH) has a broad population impact, as the annual incidence of the condition is estimated to be between 1.72 percent and 20.6 percent per 100,000 people, per year, in the elderly population field^2,3^. This trend is related to the increase in population life expectancy^4^.

Clinical manifestations of CSDH are variable and depend on the mass effect exerted by CSDH on the underlying brain parenchyma. Onset symptoms include headache, mental status changes, hemiparesis, and gait disturbance up to coma^5^. Burr hole craniotomy appears to be the most used procedure for surgical evacuation, and outcomes are generally favourable. However, middle meningeal artery embolisation represents one of the therapeutic tools in treating CSDH^3^.

Several theories arose over time to explain the pathophysiology of CSDH, and a number of clinical factors must be considered in the treatment of CSDH^2,3,6–12^. In particular, understanding the underlying pathophysiological processes related to angiogenesis, fibrinolysis, and inflammation is essential for developing potential drug treatments^13^.

Different studies have analysed the ultrastructure of CSDH membranes concerning their formation and the presence of membranes surrounding the hematoma^14–17^. In addition, other ultrastructural studies have highlighted singular aspects of the membranes, referred to as the "outer membrane" and "inner membrane" of CSDH^18–20^. However, there are no systematic immunohistochemical studies on the "outer membrane" and "inner membrane" of CSDH.

Our study aims to assess whether there is a relationship between the degree of cortical atrophy and CSDH development by correlating clinical data with immunohistochemistry and ultrastructural microscopy analysis of the "outer" and "inner" membranes of CSDH. Our study brings new multidisciplinary evidence, explaining the mechanisms by which cortical atrophy can result as a triggering factor in the pathophysiology of CSDH formation.

## Materials and methods

### Study cohort and inclusion criteria

This study compared the cortical atrophy of 190 patients (CSDH group) with unilateral CSDH and 190 healthy volunteers (control group). Both groups of patients were randomly and retrospectively selected at Policlinico Umberto I University Hospital of Sapienza University of Rome between January 2018 and December 2021. The inclusion criteria for the control group consist of healthy patients with no history of prior neuromuscular, neurological, chronic renal failure (stage > II), severe heart failure, liver cirrhosis, organ transplantation, severe episodes of dehydration, alcoholism, substance abuse, rheumatoid arthritis, lupus, and infectious diseases of the central nervous system. (1) The healthy control group did not report any neurosurgical or neurological disorders during this specific follow-up period (January 2018 and December 2021). Regarding the age, patients older than 40 with a mean age of 63 years were selected. Patients with newly diagnosed unilateral with CSDH who underwent craniotomy surgery by burr hole and evacuation were included in the CSDH group, these patients included did not present important co-morbidities and reported ASA score ≤ II and were not under antiplatelet and/or anticoagulant therapy. Patients with bilateral subdural hematoma, with a known diagnosis of dementia, ischemic and hemorragic stroke, intraparenchymal and subarachnoid haemorrhage, hydrocephalus, tumors of the brain, other neurological disorders and patients under anti-coagulant and anti-platelet therapy were excluded from the CSDH group. Patients with recurrence of CSDH were excluded. From the CSDH group, a subgroup of 20 patients were randomly picked upon their informed consent to perform the analysis of their "outer" and "inner" subdural hematoma membranes were randomly picked and analysed by light microscopy, transmission electron microscopy, and by immunohistochemistry techniques. The "inner membrane" was harvested after re-expansion of the parenchyma after the hematoma evacuation. Harvesting this tissue may be a complex procedure when there is poor visibility in the operative field due to the small diameter of the burr hole is small or no/poor re-expansion of the brain parenchyma after evacuation. Opening of the inner membrane is used for exploratory purposes, with microsurgical instrumentation and with the necessary intraoperative microscope aid, and this is to exclude the formation of further small chambers between the two members, which often occurs. Complete removal of the inner membrane is not recommended due to the risk of damaging the underlying parenchyma and increased chances of post-operative seizure.

### Relative cortical atrophy index

We developed the RCA index concept from the observation that age-related atrophy of cortical circumvolutions is associated with cortical cisternae enlargement. Measurement of the maximum diameter of cortical cisternae, in axial CT sections, is an accurate, reproducible and easily index in clinical practice. Parameters of this index were adopted from the study of Chrzan et al.^1^. To adequately describe the phenomenon of atrophy, three cisternae have been identified that are easily found and placed in different planes and anatomical locations. Their sum related to the maximum diameter of the cranial box was considered a rigorous and usable parameter in clinical practice to quantify cortical atrophy, and for this reason, a retrospective case–control validation was carried out in our institution. The possible variability in millimetres that is operator-dependent in the measurement of the parameters does not significantly deviate from the results obtained from the confidence intervals. Parameters to assess cortical atrophy were measured in the contralateral hemisphere of the hematoma by using axial CT scan images.

**Figure 1:**
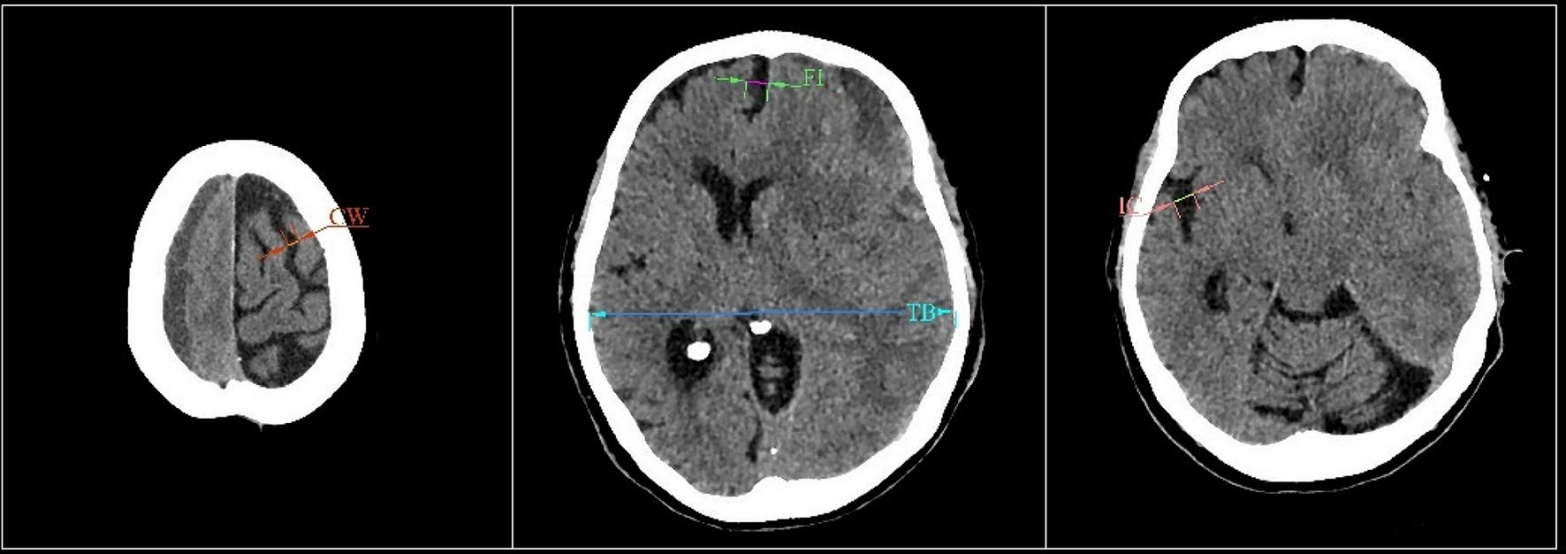
RCA Index is developed on the parameters adopted from Chrzan et al.^1^: the Insular Cisterns width (IC), the longitudinal cerebral fissure width in the anterior part (FI), and the cerebral Sulci greatest Width at the skull vault (SW) in mm in the hemisphere contralateral to the hematoma related to the Temporal Bone greatest internal distance (TB). These parameters are measured on CT scan axial images.

To objectively measure the degree of cortical atrophy, an RCA index was utilised given by the ratio of the sum of the maximum size of the diameters of the insular cistern width (IC), the longitudinal cerebral fissure width in anterior part (FI) and the cerebral sulci greatest width at the skull vault (SW) in mm in the hemisphere contralateral to the hematoma related to the temporal bones' greatest internal distance (TB) in mm. This index has an absolute value (not a unit of measurement) and it has been revealed effective in characterising the degree of cortical atrophy, Fig. 1.

($RCA index$) (space not correct= RCAindex RCA index)

$${\text{Relative}} \, {\text{cortical}} \, {\text{ atrophy}} \, {\text{ index }} ({\text{RCA}} \, {\text{index}})=\frac{(FI+IC+SW)}{TB} \frac{{\text{mm}}}{{\text{mm}}}$$

FI, IC, and SW diameters were measured in mm, considering the cistern's maximum size (cistern's width). TB was measured at the Flechsig Cut level and measured in mm. Measurements were obtained using Infinitt Pacs 7.0 and Centricity Universal Viewer programs.

In the control group, the RCA index was calculated from brain CT of healthy controls and this control group did not develop any neurosurgical pathology during the period January 2018–December 2021. This criterium was adopted to reduce the bias resulting in other pathologies altering the measurement.

Age and its correlation with the RCA index were also evaluated in this group. Subjects with signs or who would later develop neurological conditions were excluded.

In the group of patients with CSDH, the preoperative maximum diameter (PreMD) of the hematoma and the maximum post-operative diameter (PostMD) at 30 and 90 days after evacuation surgery were evaluated. Karnofsky Performance Status (KPS) at 30 days, onset symptoms, the occurrence of comorbidities. In the present study, a subdural hematoma with a maximum diameter greater than 10 mm or midline shift greater than 5 mm 30 days after evacuation surgery is considered recurrent.

The diagnosis of recurrence in CSDH is the result of a complex clinical evaluation that takes into account the radiological parameters of the residual hematoma, the compressive effects on the parenchyma and the resulting neurological symptomatology. Most CSDH studies define recurrence as the need to reoperate previous treated hematomas. One of the commonly used parameters is the ones introduced by Stanisic and Pripp^21^ who developed the Oslo CSDH Grading System that predicts post-operative hematoma recurrence requiring reoperation based on hematoma density, preoperative hematoma volume, and post-operative residual cavity volume^3^.

### Etiopathogenetic model of CSHD

The mechanism by which cortical atrophy triggers the vicious cycle that leads to the formation of this hematoma to the alteration of fluid dynamic and pressure balances between the subdural and subarachnoid spaces is not entirely clear. Therefore an etiopathogenetic model of CSHD formation based on cortical atrophy was created based on clinical, immunohistochemical, and ultrastructural findings.

**Figure 2:**
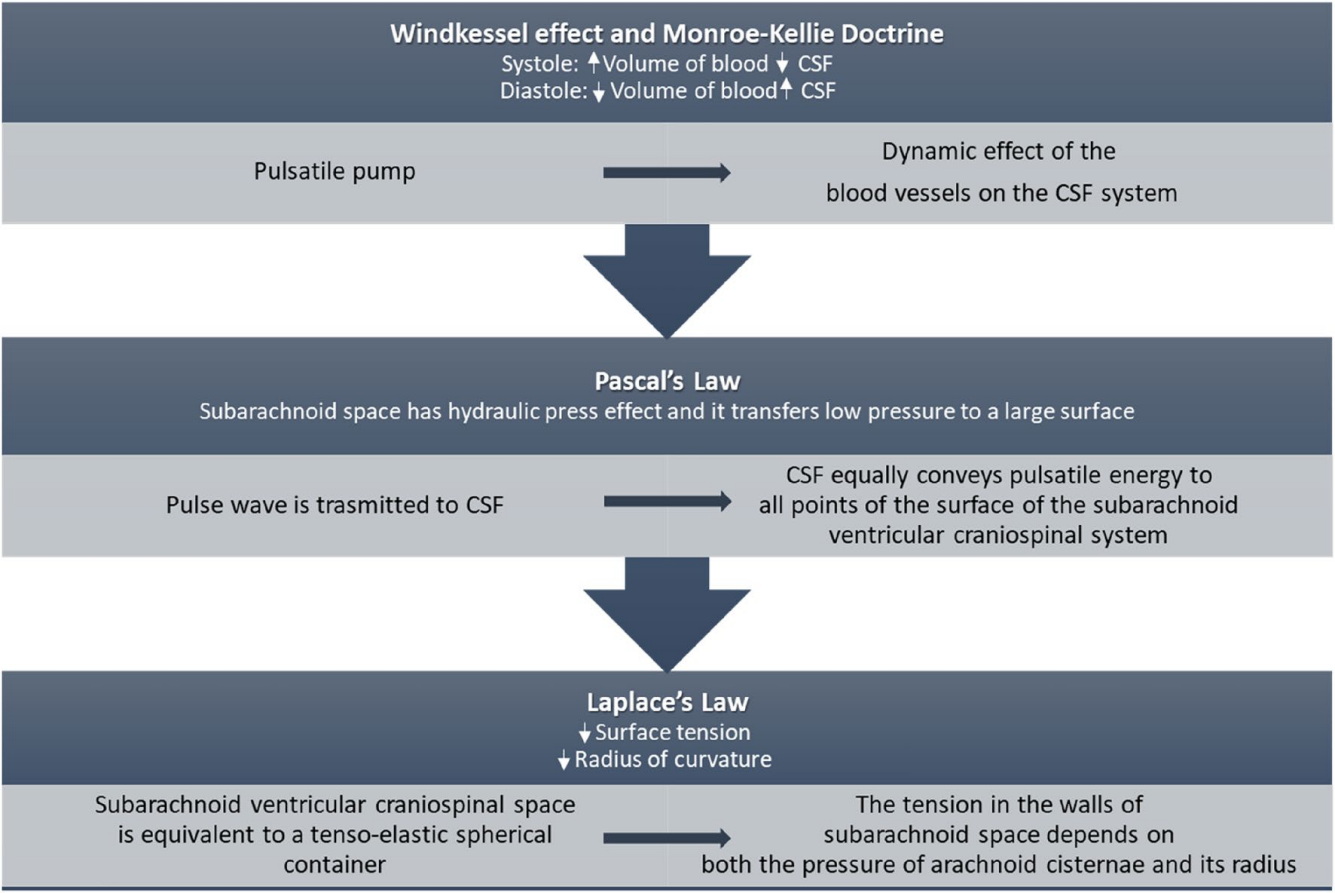
Pressure distribution in the subarachnoid ventricular craniospinal system.

It was necessary to develop a hydrodynamic model of the applied pressure balances in the subarachnoid ventricular craniospinal system (Fig. 2). The Fluid dynamics of the craniospinal system model we used is the one proposed by Benninghaus et al.^22^. This model exemplifies the craniospinal CSF system by schematising it as an isotopic tensor-elastic vessel containing incompressible Newtonian fluid that transfers pulsatile energy directly into the CSF. The pressure distribution in the realised model follows the following fluid dynamics principles: Pascal's law and Stevino's principle. The liquor flow is approximated to a laminar flow of an incompressible fluid under steady-state motion and thus follows Poiseuille's Law. Subarachnoid space is approximated to a tensile-elastic duct with isotopic material in which the summation of forces exerted on the duct wall is the orthogonal force applied to the duct wall that is equal to the wall surface tension according to Laplace's law. The contribution of this model can be important in evaluating the compliance of the subarachnoid ventricular craniospinal system in diseases such as CSHD and normotensive hydrocephalus (Fig. 2). This fluid dynamic model of pressure distribution in the subarachnoid ventricular craniospinal system is developed as it is necessary to explain the mechanism by which cortical atrophy triggers the cascade leading to CSDH formation.

### Light and transmission electron microscopy workflow

#### Sample preparation protocol for transmission electron microscopy

Samples were prepared according to the following procedure :Fixation: a solution of 2.5% glutaraldehyde in PBS 0.1 M pH 7.4 was used, and samples were immersed in the solution at least for 48 h at 4 °C^23^. Washing: samples were removed from the fixation solution and then rinsed with PBS. Post-fixation: samples were post‐fixed using a solution of osmium tetroxide 1.33% in dH_2_O (Agar Scientific, Stansted, UK) for 2 h. Washing: samples were removed from the post-fixation solution and then rinsed several times with PBS (overall time 20 min). Impregnation: samples were incubated with a solution of 1% tannic acid in dH2O (Sigma-Aldrich, St. Louis, MO, USA) for 40 min. Washing: samples were removed from the impregnation solution and then rinsed several times with PBS (overall time 20 min). Dehydration steps: specimens underwent dehydration steps in ascending ethanol series (30%, 70%, 95%, 100% v/v, three times each). Substitution: samples were incubated in propylene oxide (BDH Italia, Milan, Italy) in two passages of 20 min each. First Resin: samples were embedded in a mixture of 50:50 propylene oxide and epoxy resin Agar 100 (SIC, Rome, Italy) overnight at 25 °C under the chemical fume hood. Inclusion. Finally, samples were embedded in Agar 100 resin and put on a stove at 60 °C for 48 h.

#### Sectioning and staining procedure for light microscopy

Sectioning: semithin sections (1 µm thick) were cut by a diamond knife and then collected on glass slides. Staining: Azur II solution was used to stain in blue the semithin sections, which were then observed by light microscopy (Carl Zeiss Axioskop‐40, Zeiss, Germany). Light microscopy observation of 1 µm thick epoxy resin semithin sections allows imaging at high magnification (1000 ×) and with excellent resolution and is usually performed before ultrathin sectioning to provide a wide field imaging of the sample and allow correlative imaging.

#### Ultrathin sectioning and staining procedure for transmission electron microscopy

Ultrathin sectioning: using an ultramicrotome (Leica EM UC6, Vienna, Austria) ultrathin sections (80–90 nm) for transmission electron microscopy were cut. They were then collected on 100‐mesh copper grids (Assing, Rome, Italy). Staining procedure: ultrathin sections on copper grids were stained using Uranyless solution and Lead Citrate solution (UranyLess EM Stain, Lead Citrate 3% in Airless Bottle, Electron Microscopy Sciences, Hatfield, PA, USA). Imaging: samples were observed by a transmission electron microscope (Carl Zeiss EM10, Thornwood, NY) set with an accelerating voltage of 60 kV. Images were acquired by a CCD digital camera (AMT CCD, Deben UK Ltd, Suffolk, UK).

### Immunohistochemistry protocol

Twenty outer membrane and inner membrane samples from CSDH patients were analysed. All samples were collected and fixed in 4% neutral buffered formalin, descaled overnight in Osteodec (Bio-Optica, Milan, Italy), dehydrated in ethanol, and embedded in paraffin. Samples were cut into 2 μm thick sections using a microtome. Slices were dewaxed in xylene, rehydrated through a graded series of ethanol solutions, and washed in distilled water. Morphological analyses were performed on hematoxylin and eosin-stained sections. Collagen fibers presence was studied by Masson Trichrome histochemical staining with aniline blue (Bio-Optica Milan, Italy).

All immunohistochemical procedures were performed using Bond Max Fully Automatic IHC staining System (Leica Biosystem, Wetzlar, Germany). Serial sections were subjected to antigen retrieval according to the manufacturer's instructions (Bond Epitope Retrieval Solution 2 Product No: AR9640, Leica Biosystem, Wetzlar, Germany), and 3% hydrogen peroxide in methanol was applied to block endogenous peroxidase activity. Then, slices were incubated overnight at 4 °C with the following antibodies: CD34 (Ready to use. Product No: PA012, Leica Biosystem, Wetzlar, Germany) was used to identify endothelial vessels; CD31 (Ready to use. Product No: PA0414 Leica Biosystems, Wetzlar, Germany) was used to identify angiogenesis. After three washing steps with Phosphate Buffer Saline (PBS), slices were processed as indicated by the manufacturer's instructions (Dako LSAB2 System-HRP, Product No. K0673, Santa Clara, USA). Slices were lightly counterstained with hematoxylin, dehydrated in ethanol, cleared in xylene, and mounted with glass coverslips. Images were acquired using a digital slide scanner microscope system (D-Sight, Menarini, Florence, Italy).

### Statistical analysis

We performed a comparison of the distributions of the means of RCA index in the CSHD group versus the Control Group by Levene's Test for Equality of Variances and T-test for Equality of Means.

The distributions of data has the binormal model and both groups were mixtures of Gaussian (MG) distributions. Linear correlation measurements were performed using Pearson's correlation coefficient.

In the control group Linear regression analysis is used to predict the value of RCA index based on the value of age. In the CSHD group simultaneous multiple linear regression model with ANOVA was utilised to show the inference of RCA index parameters on the following parameters: KPS PostOp, Shift post 90, MDPreop, MDPost 90, Shift pre, Age, KPS PreOp, MDPost 30, Shift post 30. Receiver operating characteristic (ROC) analysis, which yields indices of accuracy such as the area under the curve (AUC), is increasingly being used to evaluate the performances of RCA index.

Statistical analysis and related graphical outputs were performed using IBM SPSS Statistics V.25.

### Institutional review board

The study was conducted according to the guidelines of the Declaration of Helsinki and approved by the Ethics Committee of Policlinico Umberto I Rome (study approval of the Board of the Department of Human Neurosciences—Sapienza University of Rome).

### Informed consent

Informed consent was obtained from all subjects involved in the study.

## Results

### Internal validation of RCA index: a retrospective case–control clinical analysis

#### Control group

In order to validate RCA index, its distribution within the control group was assessed. This validation step is crucial as referring to general cortical atrophy; this index would also be able to quantify it in the general population.

The variable known to be linked to cortical atrophy is age, which is why the correlation between RCA index and age was sought in the control group. The strong correlation between these two variables suggests that the RCA index adequately describes the degree of cortical atrophy. We calculated the frequency distribution of the RCA index in the Control group shown in Table 1.

**Table 1:** Descriptive analysis of RCA index in the Control group.

RCA index	Statistic	Std. error
Mean	0.137	0.003
95% CI for mean		
Lower bound	0.131	
Upper bound	0.144	
5% trimmed mean	0.136	
Median	0.132	
Variance	0.002	
Std. deviation	0.045	
Minimum	0.053	
Maximum	0.269	
Range	0.216	
Interquartile range	0.074	
Skewness	0.367	0.176
Kurtosis	− 0.650	0.351
Pearson correlation	1	0.850
		0.001
	0.389	139.477
	0.002	0.738
	190	190
Bias	0	0.001
Std. error	0	0.017
95%CI		
Lower	1	0.816
Upper	1	0.882

The parametric analysis of the frequency of the RCA index in the Control group has a normal distribution. This index has a parametric trend with a distribution of 0.137 around the mean with a Std. Error value of 0.003 and a confidence interval between 0.130 and 0.143. The distribution and parameters of the descriptive analysis in the control group is continuous with values centred on the mean value.

The mean age is 62.98 years (STD 19.15 Std. Error 1.33) and reports a positive correlation with the RCA index with Pearson Correlation 0.850 P = 0.001 (Table 2). The correlation found between RCA index and the age parameter is particularly significant.

**Table 2:** Descriptive statistics of CSDH group.

	Mean	Std. deviation
Age	78.56	7.641
RCA index	0.177	0.035
MDPreop	22,962	5.376
MDPost 30	10.682	5.372
MDPost 90	4.308	3.136
Shift pre	8.965	3.623
Shift post 30	3.312	2.510
Shift post 90	1.88	1.277
KPS PreOp	58.16	14.412
KPS PostOp	86.63	9.440

**Figure 3:**
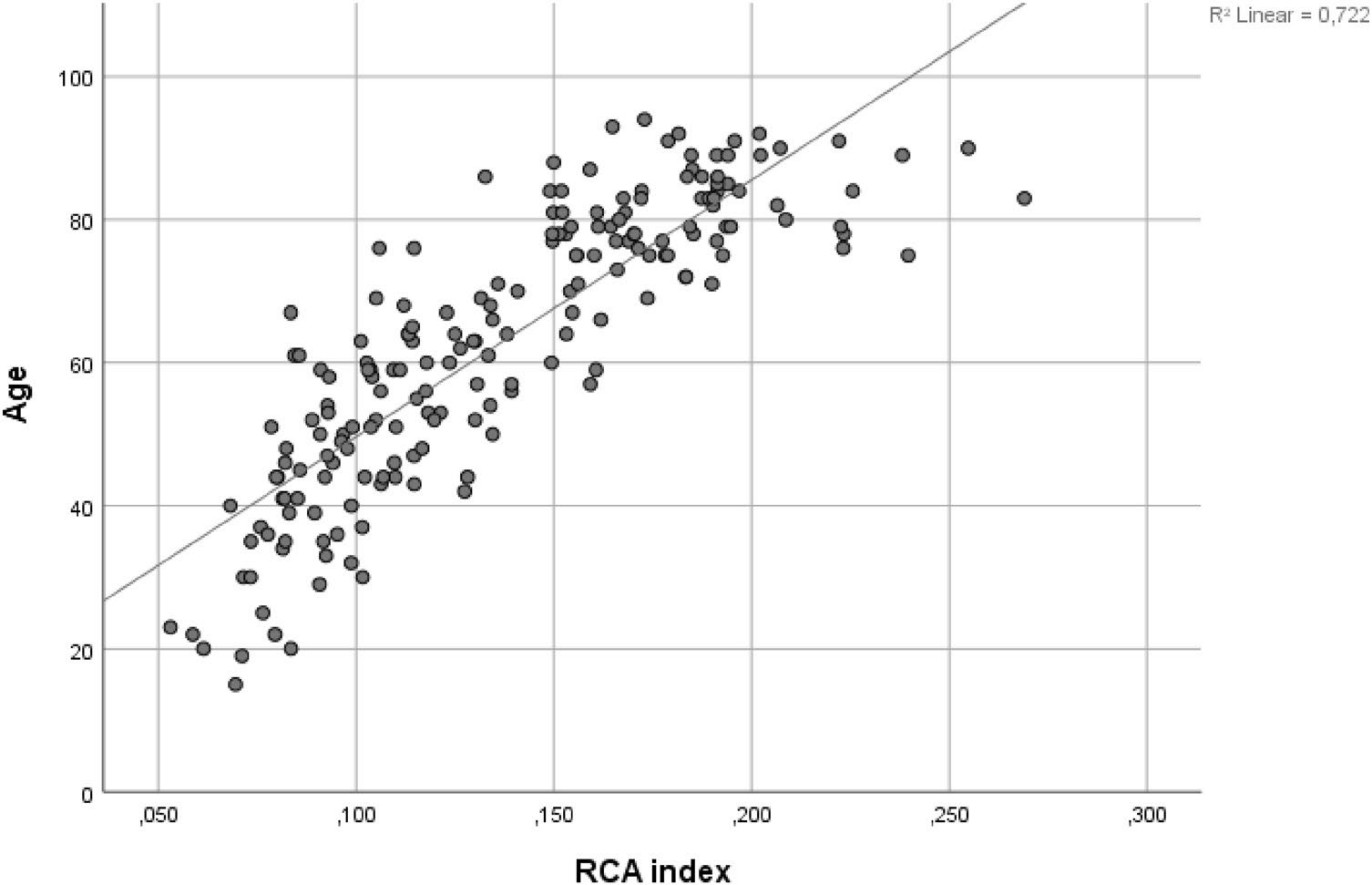
Age curve for RCA index.

In the control group, the RCA index has a linear correlation with age (R Square 0.722 and Std. Error of the Estimate 0.023) (Fig. 3).

The simple univariate regression analysis model with ANOVA test between Age and RCA index (Mean Square 0.28, F 487.64 with P 0.001) indicates a linear relationship between the two parameters. This is confirmed by standard regression analysis of the residuals (0.040 ≤ Predicted Value ≤ 0.199; Mean = 0.137; Std. Deviation = 0.038) which are homogeneously distributed around zero (− 0.062 ≤ Residual Value ≤ 0.092; Mean = 0.000; Std. Deviation = 0.023) and have a normal distribution.

A statistically significant linear correlation between the RCA index and the patient's age was found in the control group. This demonstrates that the parameters used to calculate the index effectively describe cortical atrophy since it is strongly related to age. The regression analysis and the regression standardised residual allow asserting that the trend of RCA index is linearly, Fig. 3, correlated with age with strong statistical significance.

#### CSDH group

The CSDH group consisted of 74 women and 116 men with a mean age of 78.56 years of which 89 had left CSDH and 101 had right CSDH. The mean Preop KPS is 58.16, MDPreop 22.99 and a mean preoperative shift of 8.96. (The mean postOp KPS was 86.63) with a MDPost 30 of 10.68 mm (and an MDPost 90 of 4.39 mm while the post 30-day shift was 3.31 mm and the post 90-day shift was 1.88 mm (Table 2).

Pearson correlation analysis shows a statistically significant (2-tailed) positive correlation in the CSDH Group between RCA index and the variables Age (r = 0.512; p = 0.001), MD Preop (r = 0.286; p = 0.001), and MD Post 30 (r = 0.283; p = 0.001) while it has a negative correlation with KPS PreOp (r = − 0.255; p = 0.001) and KPS PostOp (r = − 0.334; p = 0.001). Multivariate analysis shows RCA index a correlation with MDPost 30 (Mean Square 67.211; F 64.956; Sig. 0.001), with MDPost 90 (Mean Square 15.317; F 3.167; Sig. 0.001), Shift post 30 (F 237.319; Sig. 0.001). We created a multivariate regression model with ANOVA to show the inference of RCA index parameters on the parameters: KPS PostOp, Shift post 90, MDPreop, MDPost 90, Shift pre, Age, KPS PreOp, MDPost 30, Shift post 30. This linear regression model exhibits significant statistical inference (F14.479 and P0.001). The analysis of individual variables in the model, shown in Table 3, showed a higher correlation with the parameters Age (P = 0.001), MDPost 30 (P = 0.007), Shift pre (P = 0.002), Shift post 30 (P = 0.001), and KPS PreOp (P = 0.013).

**Table 3:** Correlation of the RCA index with the following parameters: age, MDPost 30, Shift pre, Shift post 30, and KPS PreOp.

Model	Unstandardised coefficients	Standardised coefficients	t	Sig	Unstandardised coefficients
(Constant)	0.036	0.035		1.032	0.303
Age	0.002	0.001	0.440	6.757	0.001
MDPreop	0.001	0.001	0.094	1.378	0.170
MDPost 30	0.001	0.001	0.220	2.733	0.007
MDPost 90	0.001	0.001	− 0.042	− 0.670	0.503
Shift pre	0.002	0.001	0.246	3.118	0.002
Shift post 30	-0.004	0.001	− 0.286	− 3.544	0.001
Shift post 90	0.001	0.002	0.037	0.609	0.543
KPS PreOp	0.001	0.001	− 0.169	− 2.512	0.013
KPS PostOp	0.001	0.001	− 0.092	− 1.333	0.184

The model's validity is confirmed by standard regression analysis of the residuals, which are homogeneously distributed and have a normal distribution. The analysis performed in this group asserts that the RCA index is positively related to the preoperative maximum diameter of hematoma and the post-operative at 30 and 90 days after surgery. Therefore, this parameter is related to the preoperative midline shift and its reduction in post-operative. Eventually, this parameter is negatively related to the preoperative KPS of patients.

#### Comparison of control group and CSDH group

We compared the value of the RCA index in the Control Group and CSDH Group, Table 4. Between the two groups in terms of the RCA index, there is a statistically significant difference and in particular, at Levene's Test for Equality of Variances, they present F 22.18 (P = 0.001). The statistical difference with high significance between the two groups was confirmed in the t-test for Equality of Means with T = 9.6 (P = 0.001; Std. Error Difference 0.004).

**Table 4:** Comparison of the RCA index value in the Control group and the CSDH group.

Group	Statistic	Bootstrap			
		Bias	Std. err	95% CI	
				Lower	Upper
RCA Index					
Control					
N	190				
Mean	0.137	0.001	0.003	0.131	0.144
Std. deviation	0.045	− 0.001	0.002	0.042	0.049
Std. error mean	0.003				
Case					
N	190				
Mean	0.177	− 0.001	0.002	0.172	0.181
Std. deviation	0.035	− 0.001	0.002	0.031	0.038
Std. error mean	0.002				

**Figure 4:**
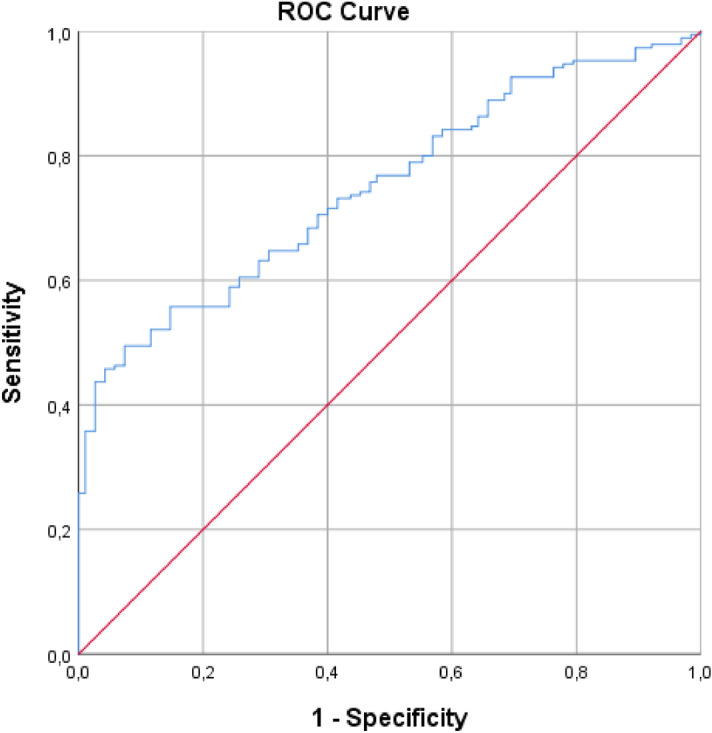
The area under the ROC curve AUROC.

Given the significance of the difference between the two groups, we evaluated whether RCA index compared them to ROC Curve, Fig. 4.

The values of Area Under ROC Curve (AUROC) is 0.749 (Std. Error = 0.025; Asymptotic Sig. = 0.001; Asymptotic 95% Confidence Interval: Lower Bound = 0.701 and Upper Bound = 0.798); this suggests that the RCA index is reliable in detecting patients with CSDH. The parameter we studied results effective in identifying patients from healthy controls.

## Morphological observations

### Immunohistochemistry

**Figure 5:**
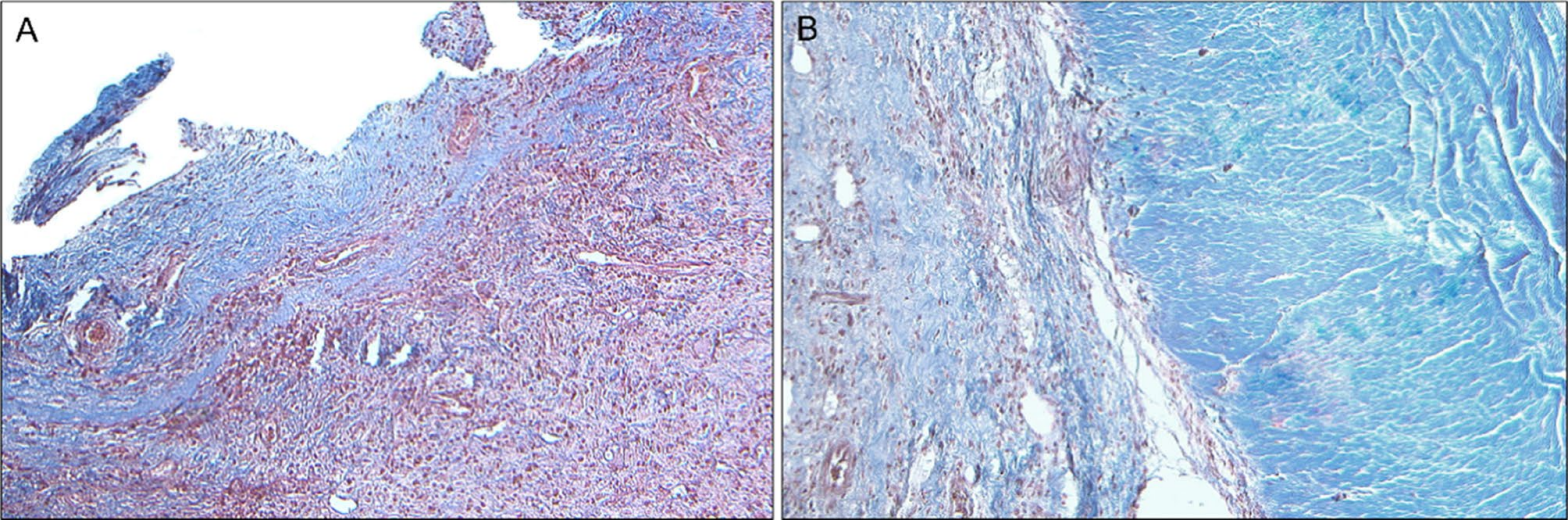
Light microscopy on paraffin sections of the inner membrane (**A**) and outer membrane (**B**) surrounding a CSDH. Collagen fibers are stained in blue, by Masson Trichrome with aniline blue histochemical staining (Bio-Optica); (**A**) 5 ×; (**B**) 10 ×.

Histological examination using histochemical Trichrome staining shows a considerable increase in the fibrotic component of the outer and inner membrane of the subdural hematoma (Fig. 5).

**Figure 6:**
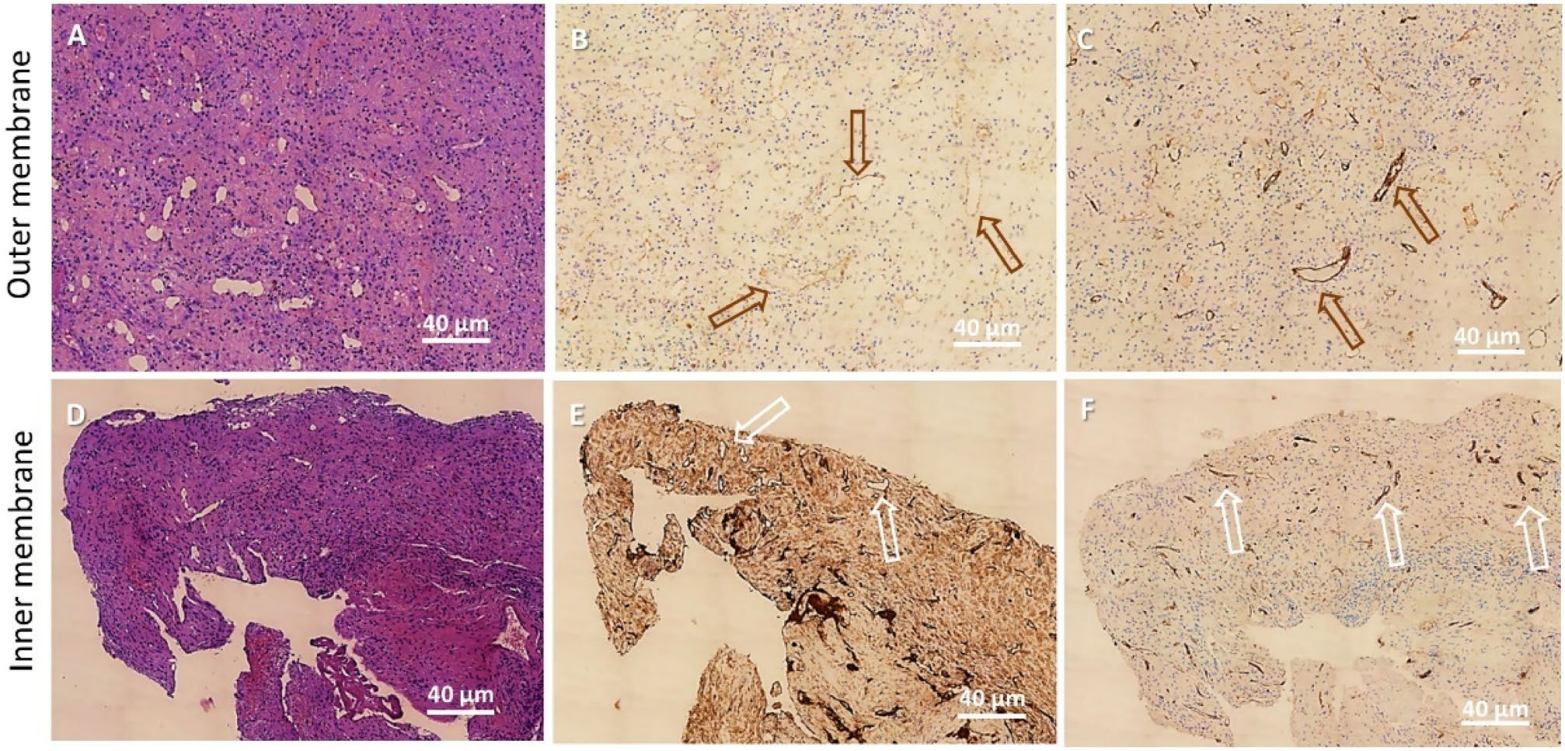
Hematoxylin and eosin histochemical staining of outer membrane (**A**) and inner membrane (**D**). Immunohistochemical modifications of the outer membrane (**B**–**C**) and the inner membrane (**E**–**F**) surrounding a CSDH. An increase in fibrotic tissue and neovascularisation are the main aspects detected. The neoformed CD31+ vessels (arrows), (**B**: outer membrane; **E**: inner membrane), appear to increase in number in the inner membrane and seem to be arranged in a different distribution pattern compared to CD34 + vessels (**C**: outer membrane; **F**: inner membrane). Haematoxylin and Eosin 100 × (**A**, **D**); CD31 stain 100 × (**B**, **E**); CD34 stain 100 × (**C**, **F**). Bar = 40 µm.

Vessel distribution was studied by immunohistochemical staining for CD31 and CD34. A significant increase in vascular components resulted positive for CD31 compared to CD34 was observed in the inner membrane of CSDH, in all observed samples. Histologic modifications of the outer membrane and inner membrane surrounding subdural hematoma are shown in Fig. 6. In addition to fibrosis, there is evidence of marked neoformation of capillary vessels, CD31+ (**B**, **E**), which display a different pattern of distribution from vessels CD34+ in terms of number (increase) and arrangement (**C**, **F**). Evaluation of the vessel components was performed in double-blind by two expert pathologists on immunohistochemistry sections.

### Light microscopy

The capsule of chronic subdural hematoma was composed of an outer membrane adhering to the dura mater, and the inner membrane, on the arachnoid side.

**Figure 7:**
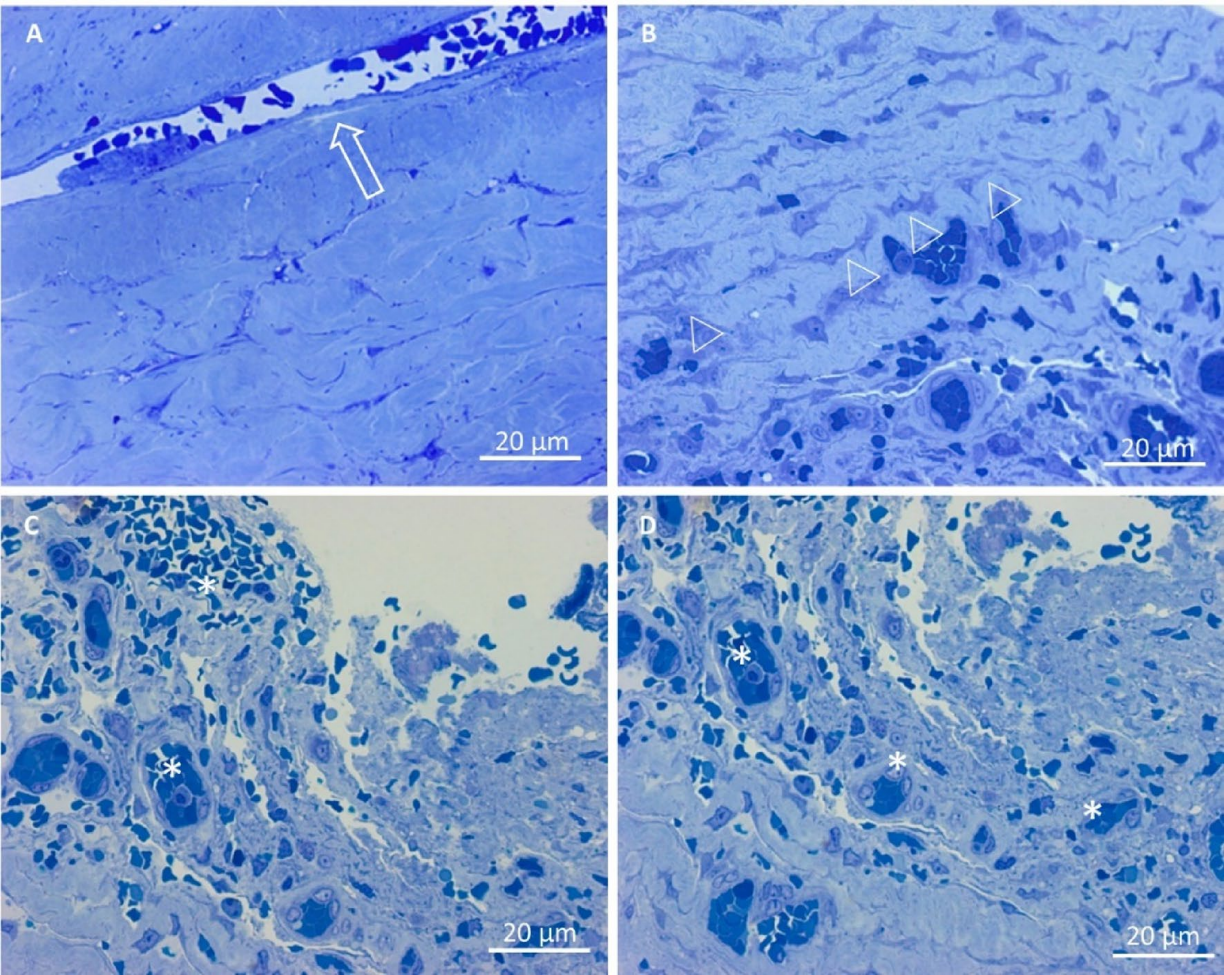
Light microscopy on semi-thin sections stained with methylene blue. (**A**) CSDH outer membrane. Flattened cells were surrounded by large bundles of collagen fibers, strictly packed and interwoven. A macro capillary was observed (arrows). (**B**) CSDH inner membrane. Various types of cells were arranged as regular ties (arrowheads), in a loose network of collagen fibers. (**C**, **D**) In the side of the inner membrane facing the hematoma cavity, an increased number of various-sized capillaries (asterisks) were detected along with a disorganised network of collagen fibers. (**A**–**D**): 400 ×, Bar = 20 µm.

LM analysis on semi-thin sections (Fig. 7) highlights that outer membrane was constituted by compact and large bundles of collagen fibres, strictly packed and interwoven, surrounding fibroblasts, capillaries, and macro capillaries (A).

In the inner membrane, collagen fibres are loosely interwoven and various cell types comprising fibroblasts, smooth muscle cells, mastocytes, and other blood-derived cells were detected. Cells were arranged more regularly, in comparison to the appearance of the outer membrane (B). On the side of the inner membrane facing the hematoma cavity, it was noticeable an increased number of various-sized capillaries and macro capillaries. In addition, the collagen network showed a very disorganised structure if compared to the deeper layers of the inner membrane. Numerous erythrocytes were also observed infiltrating this part of the inner membrane facing the hematoma cavity (C, D).

### Transmission electron microscopy

#### The CSDH outer membrane analysis

**Figure 8:**
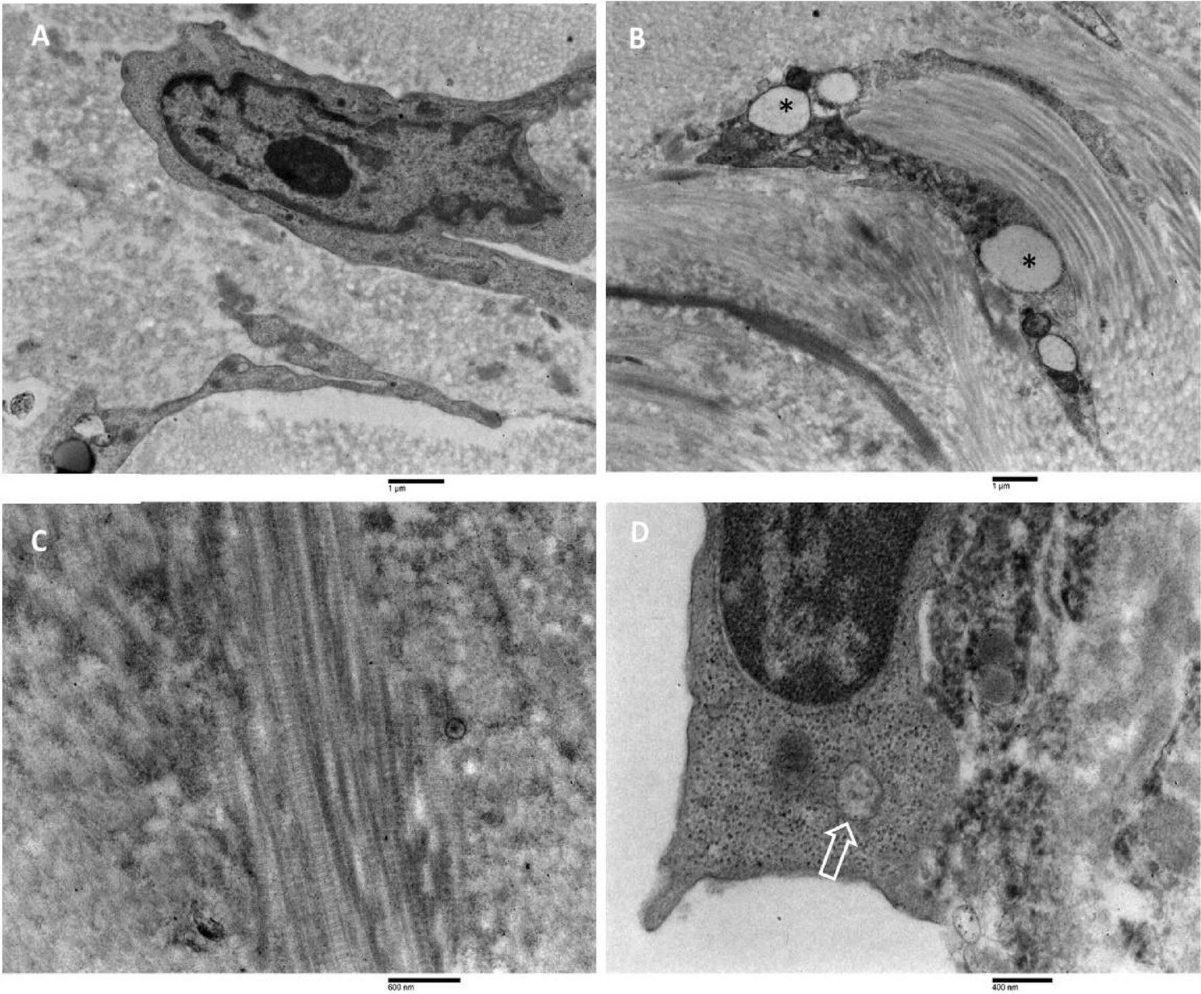
Transmission electron microscopy of the CSDH outer membrane. (**A**, **B**) flattened and elongated cells containing typical organelles in their cytoplasm were observed. Note, in (**B**), dilated vesicles of the rough endoplasmic reticulum (asterisks); A = 16,800 ×, bar = 1 µm; B = 13,400 ×, bar = 1 µm. (**C**) collagen fibers were strictly packed, running in perpendicular planes. Collagen fibers showed the typical cross-banded axial periodicity of 64 nm. 35,900 ×, bar = 600 nm. (**D**) endothelial cells showed the presence of multivesicular bodies (arrow). 44,900 ×, bar = 400 nm.

The outer membrane (Fig. 8) is mainly composed of fibroblasts that appeared as elongated or flattened cells possessing oval nuclei with small, condensed chromatin. These cells contain in their cytoplasm typical organelles such as rough endoplasmic reticulum, often appearing as dilated vesicles, free ribosomes, Golgi apparatus, mitochondria, and lipid droplets. Microvesicles and multivesicular bodies were also observed (A, B). Large amounts of collagen fibers running in oblique directions, as well as in perpendicular planes, were observed (C). Collagen fibers showed the typical cross-banded axial periodicity of 64 nm. Capillaries and macro capillaries were also observed. Endothelial cells possessed macropinocytotic vesicles and multivesicular bodies (D).

#### The CSDH inner membrane analysis

**Figure 9:**
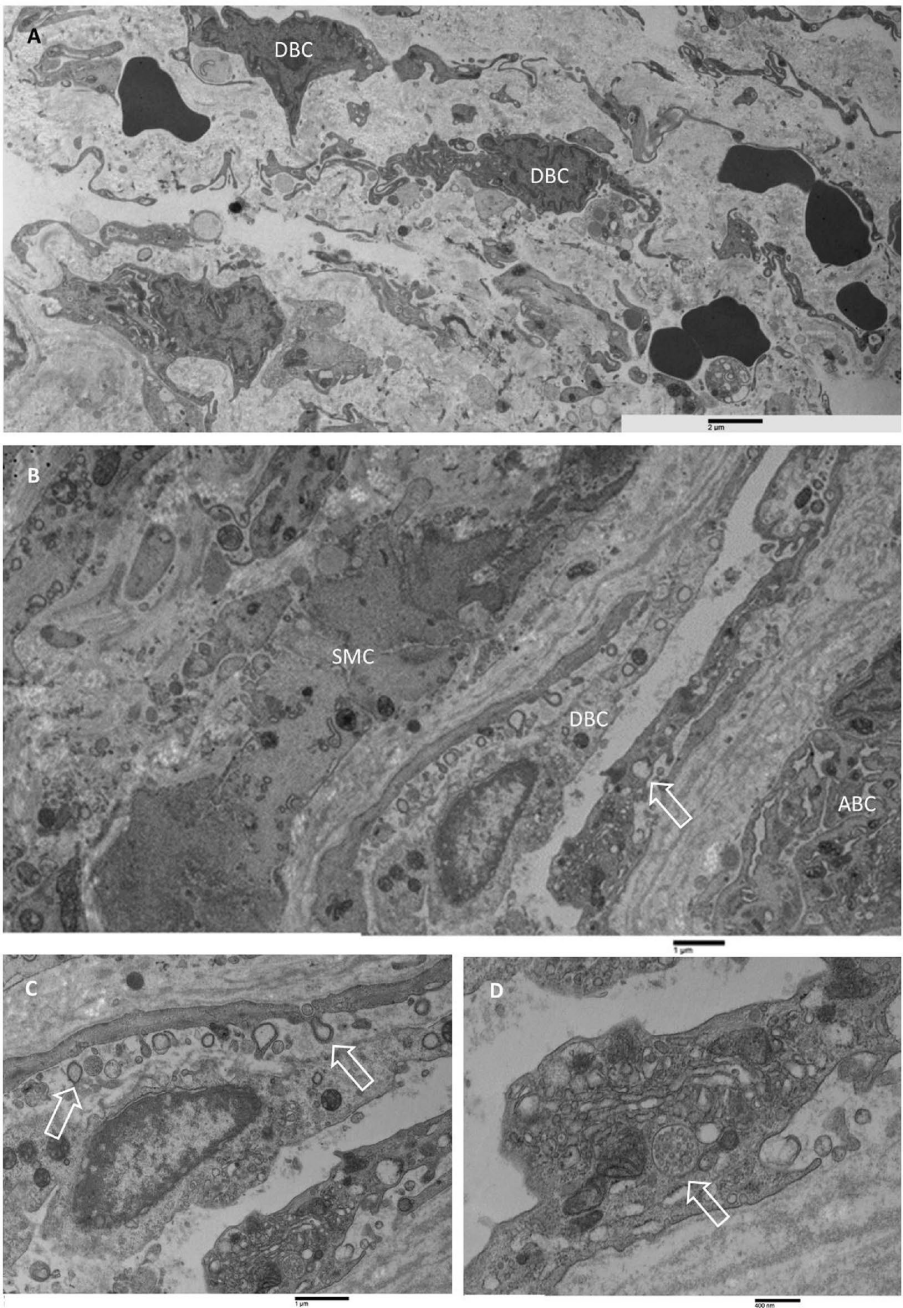
TEM analysis of the CSDH inner membrane. (**A**) Inner membrane facing to hematoma cavity. A loose collagen fibers network was evidenced. Several tiers of irregular-shaped cells (dural border cells, DBC) were present. Nuclei showed marginal condensation of heterochromatin; red blood cells were detected in the extracellular matrix. 8770 ×, bar = 2 µm. (**B**) Inner membrane facing arachnoidal border cells (ABC). Elongated cells were detected resembling dural border cells (DBC); rough endoplasmic reticulum vesicles were often dilated (arrow), and spindle-shaped cells resembled smooth muscle cells (SMC) were detected. 8770 ×, bar = 1 µm. (**C**, **D**) Closer views of (**B**). In (**C**) elongated cells showed cytoplasm containing swollen mitochondria and numerous microvesicles in the cytoplasm as well as in the extracellular matrix (arrows). In (**D**) a multivesicular body in the cytoplasm was observed (arrow). (**C**): 21,300 ×; bar = 1 µm. (**D**) 44,900 ×; bar = 400 nm.

The inner membrane facing to hematoma cavity (Fig. 9) was characterised by the presence of a loose collagen fibres network, in which several tiers of irregularly shaped cells were present, resembling the dural border cells layer, and running almost parallel to the surface of the inner membrane. Red blood cells are also observed in the extracellular matrix among cells. Cells had irregular and elongated nuclei with a marginal condensation of heterochromatin. Some nucleoli were also observed. In the cytoplasm, swollen mitochondria, enlarged endoplasmic reticulum, and numerous vesicles, as well as multivesicular bodies, were observed (A). The ultrastructural appearance of the part of the inner membrane facing arachnoidal cells was very similar to the other described above. Elongated cells had irregular nuclei in which a marginal condensation of heterochromatin was observed; in the cytoplasm, numerous organelles were observed, such as mitochondria, granular endoplasmic reticulum, Golgi apparatus, microvesicles, and multivesicular bodies (B–D). Scattered spindle-shaped cells resembling smooth muscle cells were also observed (B). Their cytoplasm was mostly occupied by microfilaments oriented mainly to the long axis of the cells. Dense bodies, caveolae, and a discontinued basal lamina-like material were observed.

**Figure 10:**
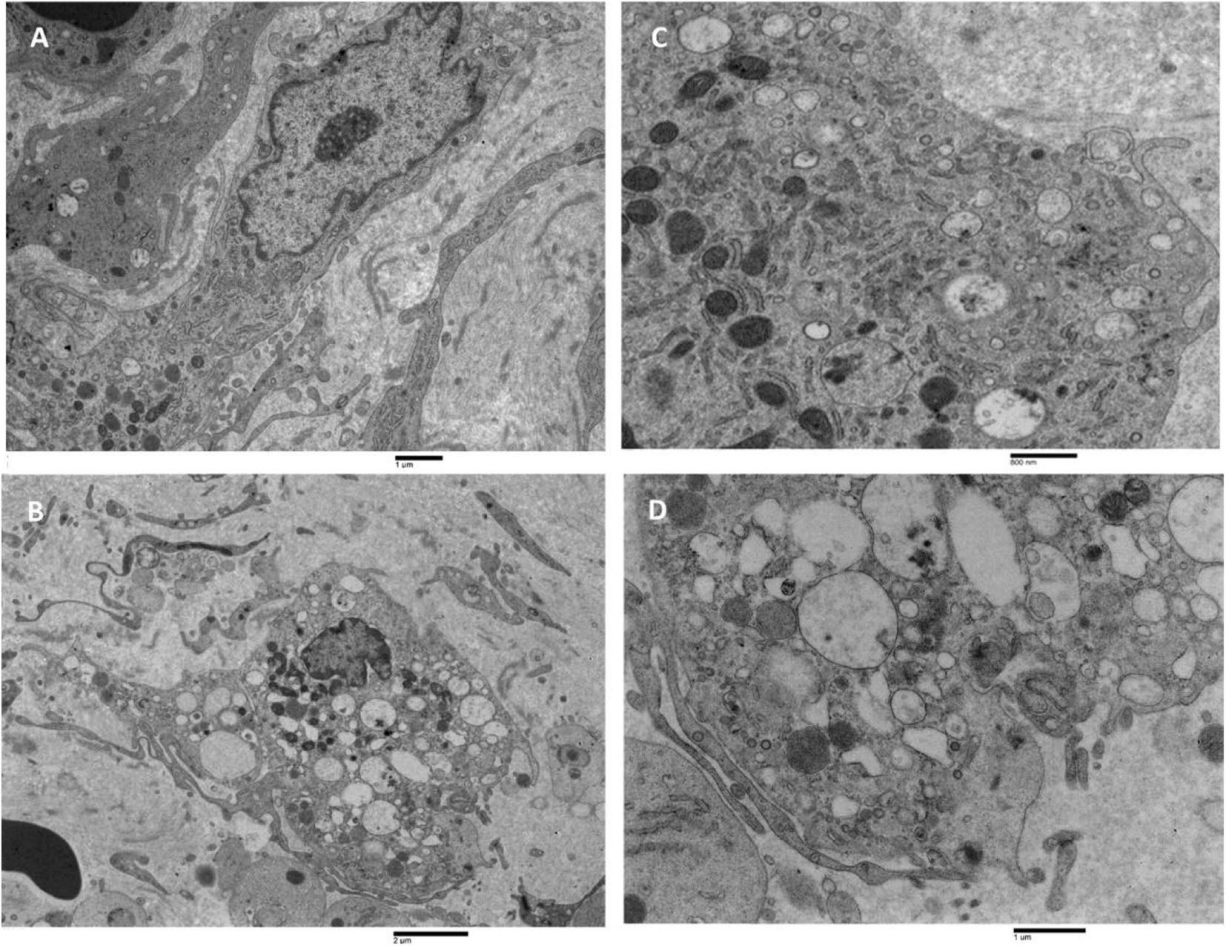
TEM analysis of the CSDH inner membrane. (**A**, **B**) Large cells with irregular nuclei with marginated chromatin and prominent nucleolus (in **A**); their cytoplasm is very rich in cellular organelles such as rough endoplasmic reticulum, Golgi apparatus, mitochondria; dilated rough endoplasmic reticulum (in **B**). (**A**) 14,000 ×, bar = 1 µm; (**B**) 10,900 ×, bar = 2 µm. (**C**, **D**) Closer view of large cells cytoplasm showing the ultrastructure of dilated rough endoplasmic reticulum (typically structured in **C**), swollen mitochondria, numerous microvesicles, and multivesicular bodies. (**C**) 24,800 ×, bar = 800 nm; D: 21,300 ×, bar = 1 µm.

A common ultrastructural feature of the inner membrane (Fig. 10) was the presence of large cells with irregular nuclei having marginated chromatin and prominent nucleoli (A); in the cytoplasm, a considerable number of organelles such as granular endoplasmic reticulum, mitochondria, multivesicular bodies, free polyribosomes and numerous microvesicles ranging from 100 nm to 1 µm were detected (B); in some cells, the rough endoplasmic reticulum vesicles were strongly dilated; dilated vesicles were discretely electron-dense, possibly due to the presence of secretory material in the lumen and were sparsely populated by ribosomes. Near dilated endoplasmic reticulum vesicles, swollen mitochondria were founded (C, D).

**Figure 11:**
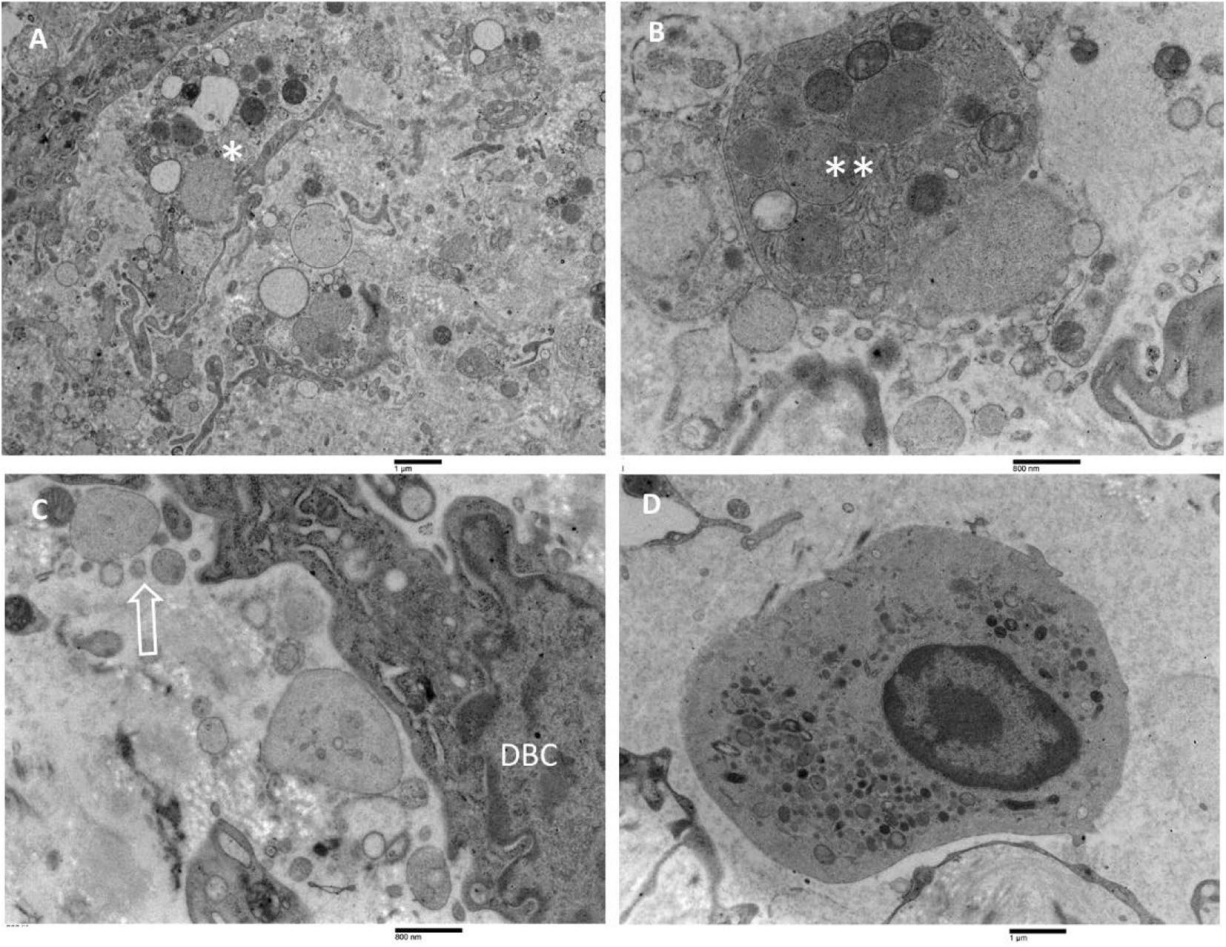
Transmission electron microscopy of the CSDH inner membrane. (**A**–**C**) In the extracellular matrix, a loose network of collagen fibres and a presence of disintegrating cells (asterisk), apoptotic bodies (double asterisks), various-sized microvesicles, and exosomes (arrow). DBC = dural border cells. (**A**) 14,000 ×, bar = 1 µm; (**B**, **C**) 24,800 ×, bar = 800 nm. (**D**) a macrophage in the extracellular matrix. 16,800 ×, bar = 1 µm.

Another common ultrastructural feature of the inner membrane (Fig. 11) was the presence, in the extracellular matrix of the inner membrane, of disintegrating cells, fibrin network, blood pigmentation, red blood cells, and many other membranous particles such as apoptotic bodies, microparticles, microvesicles and exosomes (A–C). In addition, inflammatory cells, macrophages, and often degranulated eosinophils were observed in the extracellular matrix (D).

**Figure 12:**
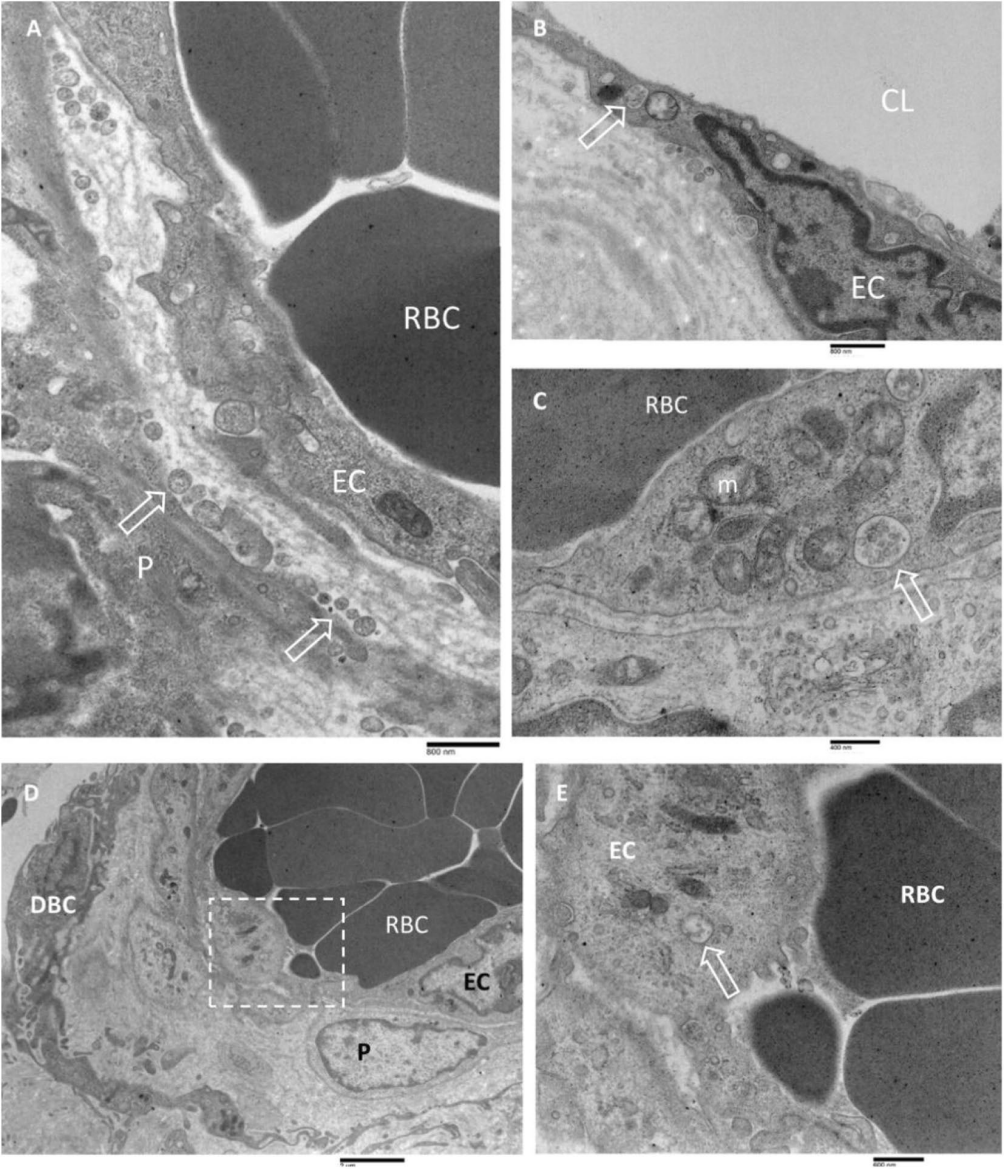
Transmission electron microscopy of capillaries in the CSDH inner membrane. (**A**) In the space between endothelial cells (EC) and pericytes (P) numerous microvesicles and exosomes (arrows) were seen. The basement membrane was discontinuous. RBC: red blood cells. 24,800 ×, bar = 800 nm. (**B**) Endothelial cell (EC) with an irregular nucleus, possessing a multivesicular body in the cytoplasm (arrows). Some microvesicles were detected in the extracellular space beneath the plasma membrane. Capillary lumen: CL; extracellular matrix: EM. 24,800 ×, bar = 800 nm. (**C**) Endothelial cells containing swollen mitochondria (m) and multivesicular bodies in the cytoplasm (arrow). Red blood cell: RBC; pericyte: P. 44,900 ×, bar = 400 nm. (**D**) dilated capillary containing numerous red blood cells (RBC) in the lumen. As provided by clear cytoplasm, some pericytes (P) were observed surrounding endothelial cells (EC), DBC: dural border cell. 10,900 ×, bar = 2 µm. (**E**) A closer view of a boxed area in (**D**). An endothelial cell (EC) contains in their cytoplasm some mitochondria, numerous micropinocytosis vesicles, and multivesicular bodies (arrow). 28,600 ×, bar = 600 nm.

A large number of capillaries were commonly detected in the part of the inner membrane facing the hematoma cavity (Fig. 12A). Detected capillaries showed various diameter sizes (measuring 20 to 30 µm in maximal diameter), containing platelets offering multiple degrees of electron density (not shown in the photographs) and packed red blood cells. The endothelial cells (B) possessed large nuclei and irregular profiles with short surface microvilli and often discontinuous basement membranes. Their cytoplasm was rich in free ribosomes, pinocytotic vesicles, and swollen mitochondria; the presence of multivesicular bodies was also frequently detected (C). Pericytes (D) varied remarkably in size and shape, and electron density. They were more highly branched, with cytoplasmic extensions contacting neighbouring pericytes; in their cytoplasm, free ribosomes and pinocytotic vesicles were detected, as well as granular endoplasmic reticulum often dilated. Finally, very interesting was the presence of many microvesicles and exosomes (E) in the space between endothelial cells and surrounding pericytes in most of the capillaries observed.

## Discussion

### Clinical studies

Cortical atrophy related to normal brain aging can be defined as widening cortical arachnoid cisternae. Moreover, a simultaneous increase in the size of the internal and external cerebrospinal fluid (CSF) spaces is characteristic of the physiological process of aging^24^.

Jang et al.^25^ show that depressed brain volume > 50 cm^3^ was an independent predictor of CSDH recurrence following surgical treatment. In addition, Yang et al. define cortical atrophy as the ratio expressed in % between the CSF volume divided by the volume of the intracranial space [the cerebrospinal fluid (CSF) volume to intracranial space (ICS) volume: A = 100 × vol (CSF)/vol (ICS)] and concludes that cortical atrophy is associated with the development of CSDH and the risk increases after age 65^7^.

Other authors^25^ consider brain volume reduction to be a prognostic factor. It is not clarified whether brain volume reduction is related to cortical or subcortical atrophy and the pathogenetic mechanism that would lead to the formation of CSDH. Age-related cortical atrophy is a para physiological phenomenon not associated with dementia^1^.

The parameters used in this study to describe cortical atrophy are the sum of FI, IC, and SW, which denote the increase in the volume of cortical cisternae related to TB's maximum internal temporal diameter. In describing this phenomenon, it proved to be very useful not to consider the diameter of a single cistern but their sum related to the diameter of the skull.

RCA is an index describing the increase of cortical cisterna concerning the skull, and it is significantly higher in the CSDH group.

This analysis showed that the enlargement of cortical cisternae relative to the size of the skull is the triggering factor. This parameter may simultaneously be the etiologic factor and an independent prognostic factor on the CSDH maximum diameter at 30 and 90 days post-operatively and midline shift in the post-operative time at 30 days.

The RCA index based on the size of cortical cisternae related to the inner diameter of the cranial box showed that in both healthy subjects (control group) and patients with CSDH, the degree of cortical atrophy is associated with patients' age with solid significance. In our investigation, cortical atrophy not associated with dementia appears to be the trigger for the biophysical phenomena that underlie the etiopathogenetic cascade.

This allowed us to conclude that CSDH correlates with an excessive increase in cisternae size. RCA also shows a positive correlation with PreMD and with PostMD. It has a negative correlation with KPS PreOp and KPS PostOp.

Considering our observations, head trauma is a precipitating event that speeds up the ongoing degenerative process by promoting bleeding and establishing a locoregional inflammatory process. It often leads to the performance of CT scans with the finding of the hematoma without being implicated in its formation. Many authors have investigated side asymmetries of the cranial vault to explain the localisation and prevalence of sides in CSDH^26^. The parietal region at the parietal eminence is the most frequent localisation, followed by the frontal region. This is related to the increased curvature increase cranial vault curvature and to the reduction of the radius of curvature. The dilation of the cortical arachnoid cisterns due to cortical atrophy has a particular effect in the region (parietal and frontal draft) where the degree of curvature (K = 1/r) is greater. In these regions the arachnoid cisterns develop a lower surface tension due to their anatomical structure (Pint-Pest = (2 Ϯ)/R). The widening of the arachnoid cisterns in the regions of greatest curvature in response to cortical atrophy (Monroe–Kellie Doctrine) facilitates the passage of fluids from the subarachnoid to the subdural space. This process leads to an increased amount of CSF in the subdural space and the formation of the hygroma that precedes the appearance of the hematoma. Compression does not develop on the brain parenchyma until the pressure in the subdural space is less than the pressure in the subarachnoid space.

### Immunohistochemical and ultrastructural observations

#### Immunochemistry

The study of vessel distribution by immunohistochemical staining for CD31 and CD34 showed a significant increase in the vascular components resulted positive for CD31, compared to CD34 vessels, in the inner membrane of chronic subdural hematoma. CD31 and CD34 are well-known markers of progenitor cells in blood vessels and stromal tissues^27^. CD31, also known as Platelet endothelial cell adhesion molecule-1 (PECAM-1 or CD31) is a well-known marker of endothelial cells and a key factor for the adhesion and accumulation of platelets. CD31 plays roles in cell proliferation, apoptosis, migration, and cellular immunity^28^. CD34 is a cell surface glycoprotein and functions as a cell-to-cell adhesion factor. It can also mediate the attachment of hematopoietic stem cells to the extracellular matrix of the bone marrow or directly to stromal cells. Cells expressing CD34 (CD34+ cells) are usually found in the umbilical cord and bone marrow as hematopoietic cells or in mesenchymal stem cells, endothelial progenitor cells, endothelial cells of blood vessels but not in lymphatic cells (except pleural lymphatic cells)^29^.

Microvessel density in the CSDH outer membrane was identified using an anti-CD31 antibody in a previous study by Nanko et al.^30^. Our histochemistry observations, comparing the results obtained using CD31 and CD34 antibodies in both the outer and inner membranes, show that the newly formed CD31-positive vessels are higher in number than the CD34-positive vessels in the inner membrane than in the outer membrane. The predominance of CD31 positive vessels over CD34 positive ones in the inner membrane of the CSDH could be related to an attempt by the inner membrane to promote "circumscription and resorption" of the pre-existing hygroma. Instead, the increased presence of newly formed vessels in the CSDH inner membrane, which may also be related to hypoxia and VEGF factor expression^31,32^, resulting in excessive development of fragile and hyper permeabilised microvessels, as observed at electron microscopy level in this study. Newly formed vessels could be pre-existent to the sloughing that leads to the development and the enlargement of CSDH. Neovessels, as shown in Rauff et al.^33^, advance through stromal matrices by reorganising matrix fibres, and inducing large deformations in the stroma. Moreover, newly formed vessels secrete proteolytic enzymes and release matrix-bound cytokines^33^. Accordingly, it is logical to think that neoformed microvessels, which are particularly numerous around dural border cells, may create an area of lower resistance where subsequently, the sloughing that leads to the formation of the outer membrane and inner membrane surrounding the CSDH cavity slowly occurs.

#### Transmission electron microscopy

Ultrastructural results of the CSDH outer membrane showed the presence of flattened fibroblasts surrounded by large amounts of collagen fibres network; in addition, capillaries and microcapillaries were also observed, as previously reported^14–16,18,20,34^. In the matrix and endothelial cells were detected microvesicles and multivesicular bodies. Their presence is strictly related to a pathobiological role in disease^35^, and as critical regulators of the inflammatory response^36^.

Ultrastructural results of the CSDH inner membrane showed the typical presence of regular ties of aligned dural border cells in a loose collagen fibres network extracellular matrix, clearly confirming the origin of the inner membrane from the mechanical separation of dura mater and arachnoid in the line of natural shear, as classically described by Haines et al.^15^ and Haines et al.^16^. There are several standard features in all the observed CSDH inner membranes, some previously described and others not found in the literature data. The presence of a loose connective tissue matrix, the presence of cells resembling dural border cells, the presence of cellular debris, fibrin, and fibrinoid material as well as the presence of eosinophils and macrophages in the extracellular matrix were previously detected in the CSDH inner membrane^17^. The presence of smooth muscle cells in the inner membrane was also previously reported^19^.

New interesting ultrastructural findings observed in the CSDH inner membrane were, for the first time, also reported: (A) the presence of very large cells with the dilated rough endoplasmic reticulum, swollen mitochondria, and autophagic bodies; (B) the presence of a large number of apoptotic bodies, microvesicles, and exosomes in the extracellular matrix; (C) the presence of multivesicular bodies in the cytoplasm of dural border cells; (D) the presence of neoformed irregular and dilated microvessels located mainly in the inner membrane facing the hematoma cavity; (E) the presence of large numbers of microvesicles and exosomes in the space between endothelial cells and surrounding pericytes, in most of the capillaries observed; (F) the presence of multivesicular bodies in the endothelial cells.

The dilated rough endoplasmic reticulum, accompanied by degranulation and disaggregation of polyribosomes, resulting in free polyribosomes in the cytoplasm, could be a sign of endoplasmic reticulum oxidative stress, which has a significant impact on cell function through activation of the unfolded protein response (UPR), inhibiting new protein translation. Numerous observations suggest that rough endoplasmic reticulum stress can initiate inflammation in different pathological conditions^37–40^. Furthermore, mitochondrial swelling and consequent dysfunction are also notably involved in the pathogenesis of several human diseases associated with oxidative stress^41–44^.

The presence of extracellular vesicles and exosomes in the extracellular matrix, as well as the presence of numerous multivesicular bodies in the cytoplasm of dural border cells and endothelial cells, could also be correlated with the promotion of endothelial dysfunction and inflammation, as already demonstrated in several other diseases^45–48^. In addition, very interesting were the results of Gao et al.^49^ demonstrating that hematoma-derived exosomes of CSDH patients promote abnormal angiogenesis and inhibit hematoma absorption through miR-144-5p.

The presence of smooth muscle cells, already described by Kawano and Suzuki^19^ in the CSDH inner membrane is also likely related to chronic inflammation, as suggested by the same authors. In addition, new experimental evidence in obstructive pulmonary disease, correlates oxidative stress-induced mitochondria dysfunction with smooth muscle remodelling^43^, giving more strengthening to this hypothesis.

In summary, the ultrastructural observations in our study of the CSDH inner membrane highlight the presence of a chronic inflammatory state, which is probably the causative factor of hematoma formation, simultaneously with a minimal traumatic event.

It is also interesting to note that another experimental study shows that cerebrovascular expression of proteins related to inflammation, oxidative stress, and neurotoxicity is higher in ageing, where changes in the microvasculature are found to contribute to these changes in the ageing brain^50^.

Probably, the activation of dural border cells leads them to a fibroproliferative state in which there are high concentrations of the procollagen propeptides of type I and type III collagens in subdural fluid from which the outer and inner capsule of the hematoma is formed leading to the organisation of the collection^51^. Activation of fibroblasts in human CSDH outer membranes and activation of STAT3^52^ in CSDH endothelial cells, along with the overexpression of implicated placental growth factor (PlGF), and vascular endothelial growth factor (VEGF) are antagonised by a high-affinity soluble receptor, namely soluble VEGF receptor-1 (sVEGFR-1) which result in significantly higher concentrations in hematoma fluid than in serum^53^. High levels of PGE2 concentrations are also thought to reflect the increased activity of COX-2 expression in the dura mater and outer membrane^54^. The activation of inflammation, membrane formation, angiogenesis, and fibrinolysis processes determine CSDH progression^13^. Eotaxin-3 expression is involved in CSDH formation and growth, which has been found in CSDH fluid, inducing eosinophils into the outer membrane, resulting in elevation of TGF-b^55^. In addition, high levels of inflammatory cytokines were significantly correlated with recurrence and layering CSDH^56^.

### Etiopathogenesis hypothesis for subdural chronic hematoma

The brain parenchyma is a visco-elastic tissue^57^ within the cranial subarachnoid space. Intracranial pressure (ICP), resulting from the heart pump and synchronous with the sphygmic wave, is transmitted to the ventricular system, and it is equal at all points of the surface of the subarachnoid space. The pressure difference between two different horizontal planes is equal to the weight of the corresponding vertical column of CSF according to Pascal's law^58^. The characteristics of the ICP can be influenced by extracranial factors and exhibit a pulsatile pattern synchronous with the sphygmic wave^59^.

The dura mater consists of five layers consisting of compact and dense collagen joined with fibroblasts and elastic fibres in a mucopolysaccharide matrix^60^) and is generally considered an isotropic material^61^, an elastic modulus 70 ± 44 MPa, a tensile strength 7 ± 4 MPa, maximum strain 11 ± 3 percent and maximum force 21 ± 18 N^62^.

A virtual space exists between the dura mater and the arachnoid, as the two layers are contiguous^15,16^.

The arachnoid is an avascular membrane composed of collagen and elastic fibres, and has a variable thickness in many places, being formed by several layers of cells. Its outer dural aspect is smoother while its innermost trabeculae emerge to bridge the subarachnoid space^63^. The collagenous innermost portion of the arachnoid is the arachnoid reticular cell layer which consists of loosely arranged cells anchored by desmosomes to the inner aspect of the arachnoid barrier cell layer. These cells are impermeable to the cerebrospinal fluid due to tight intercellular junctions. A distinct continuous basal lamina separates the arachnoid reticular cell layer from the arachnoid barrier cell layer. An additional layer of flattened branching cells is present along the inner surface of the arachnoid reticular cell layer named the arachnoid border cell layer^64,65^.

**Figure 13:**
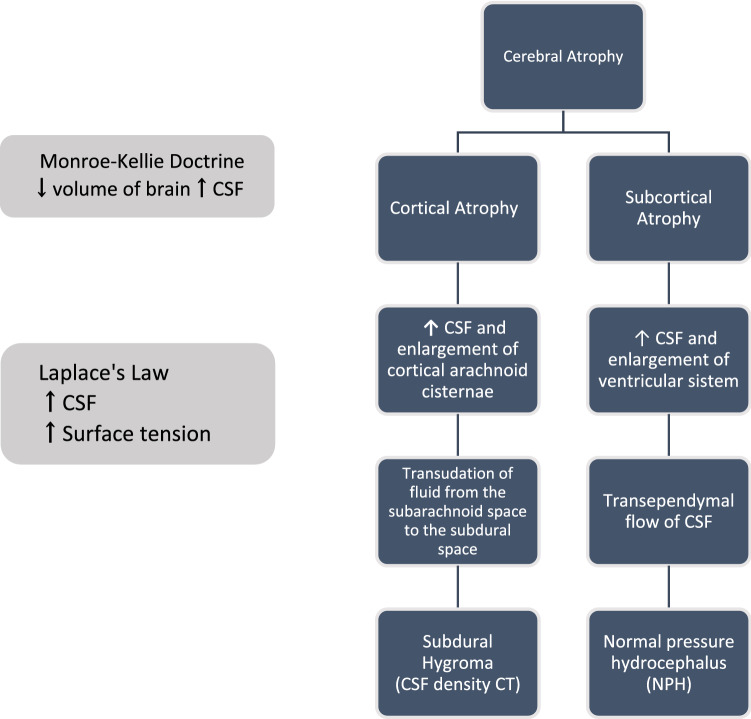
Atrophy cortical and subcortical.

The sum of encephalic parenchyma volume, CSF volume and intracranial blood volume is constant^66^. According to the Monroe Kelly doctrine, the sum of the brain parenchyma volume, CSF volume and intracranial blood volume remains constant. The reduction in the volume of the brain parenchyma in cortical atrophy is compensated by the widening of the cortical cisterns while in subcortical atrophy it is compensated by the widening of the ventricular cavities. The authors believe that cortical atrophy is the first cause of the etiopathogenesis of CSDH while subcortical atrophy is the initial cause of normotensive hydrocephalus (NPH), see Fig. 13.

Brain atrophy occurring as consequence of physiological aging in NBA is compensated by the increase in the amount of CSF received in the cortical arachnoid cisterns. This results in a widening of the cortical cisternae to increase the surface tension of the arachnoid membrane. Exceeded compliance increases CSF flow through the arachnoid and the amount of CSF in the subarachnoid space. This results in increased pressure in the subdural space and increased distance between the layer of dural border cells and the outer face of the arachnoid. This phenomenon is critical at the parietal and frontal eminence level, where the maximum curvature of the cortex is present, and the maximum surface tension developed by the arachnoid is lower. This phenomenon leads to the formation of hygroma, which is a CSF collection filtered by the arachnoid that often has no compressive effect on the parenchyma because the pressure of the subdural space is less than that of the cortical subarachnoid space^67^. Reversal of the relationship between the forces of the two areas results in compression of the parenchyma. However, this theory of hygromas formation leading to a CSDH can only be applied to the chronic subdural form and cannot explain how in many cases an acute subdural hematoma conservatively treated transforms into a CSDH, and these cases can present with subacute hematoma in which cortical atrophy may act through different mechanisms compared to the chronic form.

This phenomenon also explains the increased reabsorption of CSF ventricular ependyma at the regions of maximum curvature of the frontal and occipital horns in normotensive hydrocephalus that follows subcortical atrophy, Fig. 13.

By Laplace's law, it is verified that Pint-Pest = 2 Ϯ/R, i.e., that the surface tension Ϯ developed by the arachnoid in response to the resultant of external and internal pressure acting on the arachnoid membrane is directly proportional to the radius of curvature (curve radius R).

The surface tension results lower in the zones of primary curvatures of the parietal and frontal bones because in these regions (the Parietal and Frontal curve radius), the radius of curvature is smaller. The Parietal length results are greater than the frontal length, while the Parietal curve radius and Parietal angle degrees are less than those of the frontal bone^68^. Consequently, the maximum surface tension that the arachnoid can develop in response to the internal pressure of the cranial subarachnoid system is less in the areas of maximum parietal and frontal curvature and between the two is less at the level of the parietal bone. This explains the anatomical distribution of CSDH because the low surface tension promotes CSF permeability through the arachnoid^69^. In fact, at the parietal and frontal draft level, the surface tension and contact surface between the arachnoid system and the dura mater are lower.

Moreover, cortical atrophy results in an increased gap between the dura mater and the arachnoid plane, especially at the level of the parietal curve. In addition, distension of the arachnoid membrane occurs to increase its surface tension and counterbalance the internal CSF pressure. This process leads to the slow formation of strata of hygroma with CSF-like density which rarely has compressive effects on the adjacent parenchyma and enlargement of the arachnoid cisterns. Separation of the interface between the dura mater and arachnoid and increased CSF volume in the cortical arachnoid cisternae lead to the formation of the subdural hygroma. The arachnoid possesses sodium–potassium-ATPase and ENaC ion channels. The role of Na–K-ATPase regulates the ion permeability of the leptomeninges. At the same time, ENaC channels are located toward the subarachnoid space, and these regulate cerebrospinal fluid turnover at this interface^70^.

Bridging veins located in the subarachnoid space are distended. Local endothelial-mediated release of nitric oxide (NO) and other vasodilators leading to relaxation of smooth muscle cells within the vessel walls and diameter lumen enlargement and dilation is viewed here as a global phenomenon in which the entire vessel responds to mechanical or chemical stimulus^71^.

Unlike to what occurs in the event of a rupture of the bridging vein wall that is associated with acute or subacute subdural haemorrhage with significant neurological deficits that rapidly set in chronic subdural hematoma, a trickle of blood cells and serum occurs due to mechanical distension of the vessel wall that follows hygroma formation. The distension explains the timing of the hematoma formation which can exceed 2–3 months. This allows, following the activation of the Dural Border Cells (DBCs) and a local inflammatory state, the formation of neo-membranes that circumscribe the hematoma and impede the direct contact of the blood and inflammatory cells with the subarachnoid surface. In acute and subacute subdural hematomas with rapid and important bleeding, the direct insult of the blood and its degradation products on the arachnoid membrane occurs with the onset of rapid and severe neurological deficits associated with epileptic seizures. In CSDH the blood cells are slowly filtered through the wall of the bridging veins without major damage to the wall and the activation of the DBCs and an inflammatory state manages to circumscribe the hematoma avoiding the direct insult to the arachnoid membrane. In this case, neurological deficits are established slowly and progressively and are related to the compression exerted and, notoriously, often regress completely when the compression disappears. It basically has a mass effect on the parenchyma without causing insult and direct damage. Even minor trauma at this point facilitates the passage into the arachnoid transudate of blood cells and inflammatory cells from dural vessels received between the layers of the dura mater.

The formation of the inner subdural membrane is related to the dural border cells activation, and its fibroelastic structure is due to collagen deposition by this layer of cells. In addition, the production of cytokines by these cells activates a locoregional inflammatory process. Inflammation results in vasodilatation and self-fuels the collection's formation to a considerable size with compressive effects on the parenchyma. Nevertheless, the contents of the serum-hematologic-inflammatory collection never come in direct contact with the arachnoid plane because it is divided by the inner hematoma membrane. In addition, the gap between the inner layers of the dura mater and the arachnoid plane that occurs in cortical atrophy leads to hypoxia phenomena due to the departure from the arachnoid plane. Hypoxia leads to overexpression of VEGF, followed by neovascularisation phenomena with vessels of irregular calibre and course.

**Figure 14:**
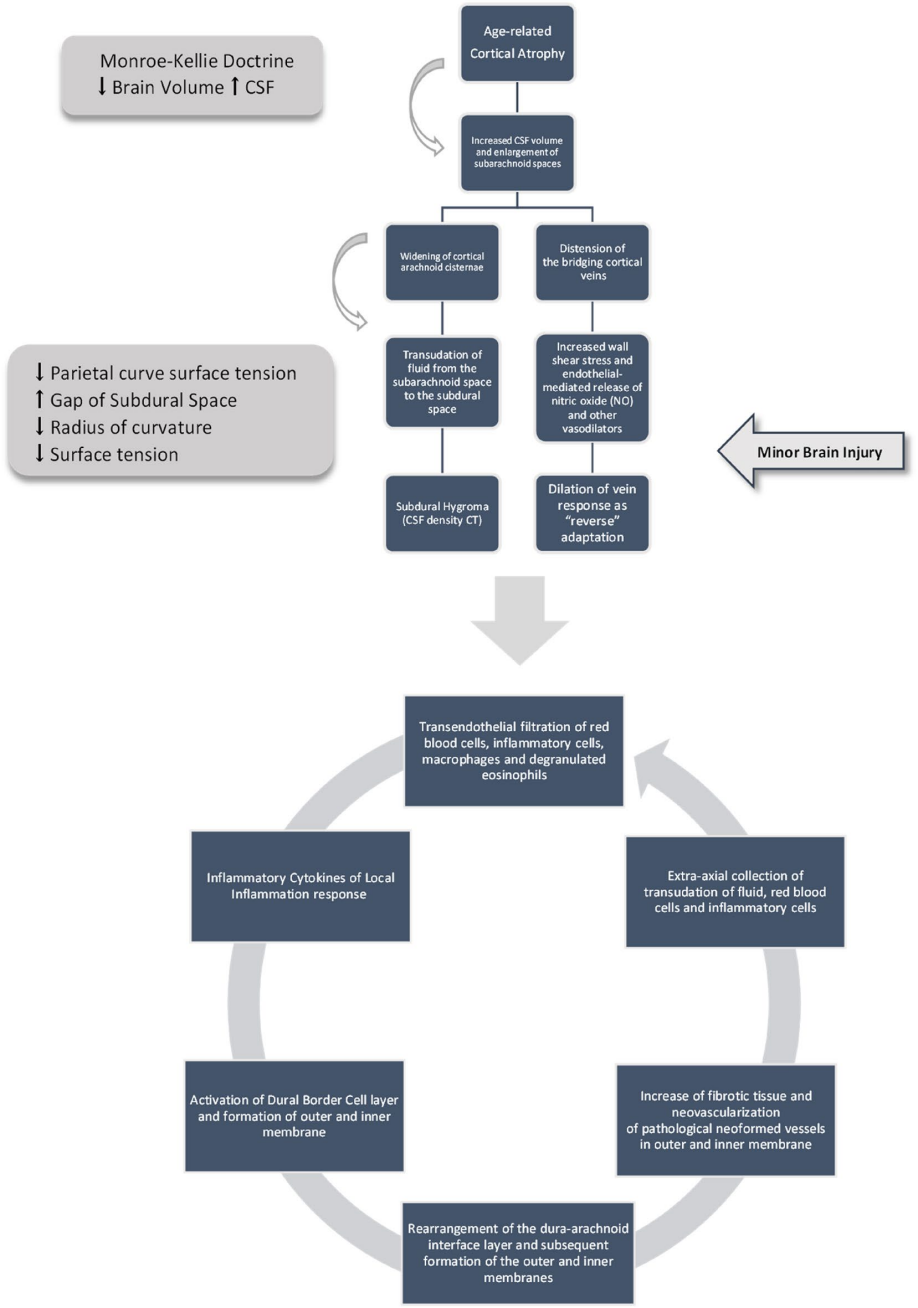
Cortical atrophy in the etiopathogenesis of chronic subdural hematoma (CSDH).

The latter membrane protects the arachnoid plane, which is neither irritated nor invaded but only compressed and displaced. And this explains the very rapid recovery of neurological deficits after evacuation surgery because it is an extracranial collection with compressive but not direct irritative or injurious effects on the arachnoid plane (Fig. 14).

## Conclusions

Our hypothesis for the etiopathogenesis of CSDH, based on clinical, ultrastructural, and immunohistochemical results, indicates that the cortical atrophy is the triggering factor of trans endothelial cellular filtration, inflammatory cascade and neo-angiogenesis leading to the formation of CSDH.

Clinical observations showed that cortical atrophy, measured with the RCA index, is strongly related to age in healthy controls. The index used in this analysis has effectively distinguished between cases and healthy controls. Cortical atrophy is a predictive factor of the maximum diameter of preoperative and post-operative CSDH and the shift of the preoperative and postoperative midline. Therefore, it has a negative correlation with the preoperative KPS of patients.

Immunohistochemistry observations showed that the newly formed CD-31 positive microvessels are higher in number than the CD34-positive microvessels in the CSDH inner membrane than in the outer membrane, suggesting that activation of angiogenesis process during CSDH growth is related to the VFGT production upon hypoxic stimuli. Hypoxia leads to overexpression of VEGF, followed by neovascularisation with vessels of irregular calibre and neovascularisation of membranes. Ultrastructural observations highlight a chronic inflammatory state in the CSDH inner membrane, which can be the main causative factor of hematoma formation.

The proposed translational model elucidates the mechanisms by which cortical atrophy can result as the main triggering factor in the pathophysiology of CSDH formation and identifies the vicious cycle that determines its slow and progressive growth. Also, it clarifies the specific anatomical site of CSDH localisation, time of onset, clinical symptomatology and neurological implications associated with this condition.

## Supplementary Information

Supplementary Information 1.

Supplementary Information 2.

Supplementary Information 3.

Supplementary Information 4.

Supplementary Information 5.

Supplementary Information 6.

Supplementary Information 7.

Supplementary Information 8.

Supplementary Information 9.

Supplementary Information 10.

Supplementary Information 11.

## Data Availability

All data generated or analysed during this study are included in this published article and its Supplementary information files.
