# Supplementary material for: Cortical atrophy in chronic subdural hematoma from ultra-structures to physical properties
Source: Sci Rep. 2023 Feb 28;13:3400. doi: 10.1038/s41598-023-30135-8 (PMC9975247; doi:10.1038/s41598-023-30135-8)
Supplement: Supplementary file 1 — Supplementary Information 1. [file 41598_2023_30135_MOESM1_ESM.doc]

GET
  FILE='C:\Users\Placido\Desktop\articolo atrofia e sottodurale cronico\controlli\controlli correlazioni.sav'.
DATASET NAME Dataset1 WINDOW=FRONT.
BOOTSTRAP
  /SAMPLING METHOD=SIMPLE
  /VARIABLES INPUT=RCAindex
  /CRITERIA CILEVEL=95 CITYPE=PERCENTILE  NSAMPLES=1000
  /MISSING USERMISSING=EXCLUDE.


Bootstrap


Notes	
Output Created	01-AUG-2021 15:56:14	
Comments		
Input	Data	C:\Users\Placido\Desktop\articolo atrofia e sottodurale cronico\controlli\controlli correlazioni.sav	
	Active Dataset	Dataset1	
	Filter	<none>	
	Weight	<none>	
	Split File	<none>	
Syntax	BOOTSTRAP
  /SAMPLING METHOD=SIMPLE
  /VARIABLES INPUT=RCAindex
  /CRITERIA CILEVEL=95 CITYPE=PERCENTILE  NSAMPLES=1000
  /MISSING USERMISSING=EXCLUDE.	
Resources	Processor Time	00:00:00,00	
	Elapsed Time	00:00:00,01	


[Dataset1] C:\Users\Placido\Desktop\articolo atrofia e sottodurale cronico\controlli\controlli correlazioni.sav


Bootstrap Specifications	
Sampling Method	Simple	
Number of Samples	1000	
Confidence Interval Level	95,0%	
Confidence Interval Type	Percentile	

FREQUENCIES VARIABLES=RCAindex
  /BARCHART FREQ
  /ORDER=ANALYSIS.


Frequencies


Notes	
Output Created	01-AUG-2021 15:56:15	
Comments		
Input	Data	C:\Users\Placido\Desktop\articolo atrofia e sottodurale cronico\controlli\controlli correlazioni.sav	
	Active Dataset	Dataset1	
	Filter	<none>	
	Weight	<none>	
	Split File	<none>	
	N of Rows in Working Data File	120361	
Missing Value Handling	Definition of Missing	User-defined missing values are treated as missing.	
	Cases Used	Statistics are based on all cases with valid data.	
Syntax	FREQUENCIES VARIABLES=RCAindex
  /BARCHART FREQ
  /ORDER=ANALYSIS.	
Resources	Processor Time	00:00:09,62	
	Elapsed Time	00:00:10,40	


Statistics	
RCA index  	
	Statistic	Bootstrap	
		Bias	Std. Error	95% Confidence Interval	
				Lower	Upper	
N	Valid	190	0	0	190	190	
	Missing	0	0	0	0	0	


RCA index	
	Frequency	Percent	Valid Percent	Cumulative Percent	Bootstrap for Percent	
					Bias	Std. Error	95% Confidence Interval	
							Lower	Upper	
Valid	,053	1	,5	,5	,5	,0	,5	,0	1,6	
	,059	1	,5	,5	1,1	,0	,5	,0	1,6	
	,061	1	,5	,5	1,6	,0	,5	,0	2,1	
	,068	1	,5	,5	2,1	,0	,5	,0	1,6	
	,069	1	,5	,5	2,6	,0	,5	,0	1,6	
	,071	1	,5	,5	3,2	,0	,5	,0	1,6	
	,071	1	,5	,5	3,7	,0	,5	,0	1,6	
	,073	1	,5	,5	4,2	,0	,5	,0	1,6	
	,073	1	,5	,5	4,7	,0	,6	,0	2,1	
	,076	1	,5	,5	5,3	,0	,5	,0	1,6	
	,076	1	,5	,5	5,8	,0	,5	,0	1,6	
	,078	1	,5	,5	6,3	,0	,5	,0	1,6	
	,079	1	,5	,5	6,8	,0	,5	,0	2,1	
	,080	1	,5	,5	7,4	,0	,5	,0	1,6	
	,080	1	,5	,5	7,9	,0	,5	,0	1,6	
	,080	1	,5	,5	8,4	,0	,5	,0	1,6	
	,081	1	,5	,5	8,9	,0	,5	,0	1,6	
	,081	1	,5	,5	9,5	,0	,6	,0	2,1	
	,082	1	,5	,5	10,0	,0	,5	,0	1,6	
	,082	1	,5	,5	10,5	,0	,5	,0	1,6	
	,082	1	,5	,5	11,1	,0	,5	,0	1,6	
	,082	1	,5	,5	11,6	,0	,5	,0	2,1	
	,083	1	,5	,5	12,1	,0	,5	,0	1,6	
	,083	1	,5	,5	12,6	,0	,5	,0	1,6	
	,084	1	,5	,5	13,2	,0	,5	,0	1,6	
	,084	1	,5	,5	13,7	,0	,5	,0	1,6	
	,085	1	,5	,5	14,2	,0	,5	,0	1,6	
	,086	1	,5	,5	14,7	,0	,5	,0	1,6	
	,086	1	,5	,5	15,3	,0	,5	,0	1,6	
	,089	1	,5	,5	15,8	,0	,5	,0	1,6	
	,090	1	,5	,5	16,3	,0	,5	,0	1,6	
	,091	1	,5	,5	16,8	,0	,5	,0	1,6	
	,091	1	,5	,5	17,4	,0	,5	,0	1,6	
	,091	1	,5	,5	17,9	,0	,5	,0	1,6	
	,092	1	,5	,5	18,4	,0	,5	,0	1,6	
	,092	1	,5	,5	18,9	,0	,5	,0	1,6	
	,092	1	,5	,5	19,5	,0	,5	,0	1,6	
	,093	1	,5	,5	20,0	,0	,5	,0	1,6	
	,093	1	,5	,5	20,5	,0	,5	,0	1,6	
	,093	1	,5	,5	21,1	,0	,5	,0	1,6	
	,093	1	,5	,5	21,6	,0	,5	,0	1,6	
	,094	1	,5	,5	22,1	,0	,5	,0	1,6	
	,095	1	,5	,5	22,6	,0	,5	,0	1,6	
	,096	1	,5	,5	23,2	,0	,5	,0	1,6	
	,097	1	,5	,5	23,7	,0	,5	,0	1,6	
	,098	1	,5	,5	24,2	,0	,5	,0	1,6	
	,099	1	,5	,5	24,7	,0	,5	,0	1,6	
	,099	1	,5	,5	25,3	,0	,5	,0	1,6	
	,099	1	,5	,5	25,8	,0	,5	,0	1,6	
	,101	1	,5	,5	26,3	,0	,5	,0	1,6	
	,102	1	,5	,5	26,8	,0	,5	,0	1,6	
	,102	1	,5	,5	27,4	,0	,5	,0	1,6	
	,102	1	,5	,5	27,9	,0	,5	,0	1,6	
	,103	1	,5	,5	28,4	,0	,5	,0	1,6	
	,103	1	,5	,5	28,9	,0	,5	,0	1,6	
	,104	1	,5	,5	29,5	,0	,5	,0	1,6	
	,104	1	,5	,5	30,0	,0	,5	,0	1,6	
	,104	1	,5	,5	30,5	,0	,5	,0	1,6	
	,105	1	,5	,5	31,1	,0	,5	,0	1,6	
	,105	1	,5	,5	31,6	,0	,5	,0	1,6	
	,106	1	,5	,5	32,1	,0	,5	,0	1,6	
	,106	1	,5	,5	32,6	,0	,5	,0	1,6	
	,106	1	,5	,5	33,2	,0	,5	,0	2,1	
	,107	1	,5	,5	33,7	,0	,5	,0	1,6	
	,109	1	,5	,5	34,2	,0	,6	,0	1,6	
	,110	1	,5	,5	34,7	,0	,5	,0	1,6	
	,110	1	,5	,5	35,3	,0	,5	,0	1,6	
	,110	1	,5	,5	35,8	,0	,5	,0	1,6	
	,111	1	,5	,5	36,3	,0	,5	,0	1,6	
	,112	1	,5	,5	36,8	,0	,5	,0	1,6	
	,113	1	,5	,5	37,4	,0	,5	,0	1,6	
	,113	1	,5	,5	37,9	,0	,6	,0	1,6	
	,114	1	,5	,5	38,4	,0	,5	,0	1,6	
	,114	1	,5	,5	38,9	,0	,5	,0	1,6	
	,115	1	,5	,5	39,5	,0	,5	,0	1,6	
	,115	1	,5	,5	40,0	,0	,5	,0	1,6	
	,115	1	,5	,5	40,5	,0	,5	,0	1,6	
	,115	1	,5	,5	41,1	,0	,5	,0	1,6	
	,117	1	,5	,5	41,6	,0	,5	,0	1,6	
	,118	1	,5	,5	42,1	,0	,5	,0	1,6	
	,118	1	,5	,5	42,6	,0	,5	,0	1,6	
	,118	1	,5	,5	43,2	,0	,5	,0	1,6	
	,120	1	,5	,5	43,7	,0	,5	,0	2,1	
	,121	1	,5	,5	44,2	,0	,5	,0	1,6	
	,123	1	,5	,5	44,7	,0	,5	,0	1,6	
	,124	1	,5	,5	45,3	,0	,5	,0	1,6	
	,125	1	,5	,5	45,8	,0	,5	,0	1,6	
	,126	1	,5	,5	46,3	,0	,5	,0	2,1	
	,127	1	,5	,5	46,8	,0	,5	,0	1,6	
	,128	1	,5	,5	47,4	,0	,5	,0	1,6	
	,130	1	,5	,5	47,9	,0	,5	,0	1,6	
	,130	1	,5	,5	48,4	,0	,5	,0	1,6	
	,130	1	,5	,5	48,9	,0	,5	,0	1,6	
	,131	1	,5	,5	49,5	,0	,5	,0	2,1	
	,132	1	,5	,5	50,0	,0	,5	,0	1,6	
	,133	1	,5	,5	50,5	,0	,5	,0	1,6	
	,133	1	,5	,5	51,1	,0	,5	,0	1,6	
	,134	1	,5	,5	51,6	,0	,5	,0	1,6	
	,134	1	,5	,5	52,1	,0	,5	,0	1,6	
	,135	1	,5	,5	52,6	,0	,5	,0	1,6	
	,135	1	,5	,5	53,2	,0	,5	,0	1,6	
	,136	1	,5	,5	53,7	,0	,5	,0	1,6	
	,138	1	,5	,5	54,2	,0	,5	,0	1,6	
	,139	2	1,1	1,1	55,3	,0	,8	,0	2,6	
	,141	1	,5	,5	55,8	,0	,5	,0	1,6	
	,149	1	,5	,5	56,3	,0	,5	,0	1,6	
	,149	1	,5	,5	56,8	,0	,5	,0	1,6	
	,150	1	,5	,5	57,4	,0	,5	,0	2,1	
	,150	1	,5	,5	57,9	,0	,5	,0	1,6	
	,150	1	,5	,5	58,4	,0	,5	,0	1,6	
	,150	1	,5	,5	58,9	,0	,5	,0	1,6	
	,151	1	,5	,5	59,5	,0	,5	,0	1,6	
	,152	1	,5	,5	60,0	,0	,5	,0	1,6	
	,152	1	,5	,5	60,5	,0	,5	,0	1,6	
	,153	1	,5	,5	61,1	,0	,5	,0	1,6	
	,153	1	,5	,5	61,6	,0	,5	,0	1,6	
	,154	1	,5	,5	62,1	,0	,5	,0	1,6	
	,154	1	,5	,5	62,6	,0	,5	,0	1,6	
	,155	1	,5	,5	63,2	,0	,5	,0	1,6	
	,156	1	,5	,5	63,7	,0	,5	,0	1,6	
	,156	1	,5	,5	64,2	,0	,5	,0	1,6	
	,156	1	,5	,5	64,7	,0	,5	,0	1,6	
	,159	1	,5	,5	65,3	,0	,5	,0	1,6	
	,159	1	,5	,5	65,8	,0	,5	,0	1,6	
	,160	1	,5	,5	66,3	,0	,5	,0	1,6	
	,161	1	,5	,5	66,8	,0	,5	,0	1,6	
	,161	1	,5	,5	67,4	,0	,5	,0	1,6	
	,161	1	,5	,5	67,9	,0	,5	,0	1,6	
	,162	1	,5	,5	68,4	,0	,5	,0	1,6	
	,164	1	,5	,5	68,9	,0	,5	,0	1,6	
	,165	1	,5	,5	69,5	,0	,5	,0	1,6	
	,166	1	,5	,5	70,0	,0	,5	,0	2,1	
	,166	1	,5	,5	70,5	,0	,5	,0	1,6	
	,167	1	,5	,5	71,1	,0	,5	,0	1,6	
	,168	1	,5	,5	71,6	,0	,5	,0	1,6	
	,168	1	,5	,5	72,1	,0	,5	,0	1,6	
	,169	1	,5	,5	72,6	,0	,5	,0	1,6	
	,170	1	,5	,5	73,2	,0	,5	,0	1,6	
	,170	1	,5	,5	73,7	,0	,5	,0	1,6	
	,171	1	,5	,5	74,2	,0	,5	,0	1,6	
	,172	1	,5	,5	74,7	,0	,5	,0	1,6	
	,172	1	,5	,5	75,3	,0	,5	,0	1,6	
	,173	1	,5	,5	75,8	,0	,5	,0	1,6	
	,174	1	,5	,5	76,3	,0	,5	,0	1,6	
	,174	1	,5	,5	76,8	,0	,5	,0	1,6	
	,177	1	,5	,5	77,4	,0	,5	,0	1,6	
	,178	1	,5	,5	77,9	,0	,5	,0	1,6	
	,179	1	,5	,5	78,4	,0	,5	,0	1,6	
	,179	1	,5	,5	78,9	,0	,5	,0	1,6	
	,182	1	,5	,5	79,5	,0	,5	,0	1,6	
	,183	1	,5	,5	80,0	,0	,5	,0	1,6	
	,183	1	,5	,5	80,5	,0	,5	,0	1,6	
	,184	1	,5	,5	81,1	,0	,5	,0	1,6	
	,184	1	,5	,5	81,6	,0	,5	,0	1,6	
	,185	1	,5	,5	82,1	,0	,5	,0	1,6	
	,185	1	,5	,5	82,6	,0	,5	,0	1,6	
	,185	1	,5	,5	83,2	,0	,5	,0	1,6	
	,187	1	,5	,5	83,7	,0	,5	,0	1,6	
	,187	1	,5	,5	84,2	,0	,5	,0	1,6	
	,189	1	,5	,5	84,7	,0	,5	,0	1,6	
	,190	1	,5	,5	85,3	,0	,5	,0	1,6	
	,190	1	,5	,5	85,8	,0	,5	,0	1,6	
	,190	1	,5	,5	86,3	,0	,5	,0	1,6	
	,191	1	,5	,5	86,8	,0	,5	,0	1,6	
	,191	1	,5	,5	87,4	,0	,5	,0	1,6	
	,191	1	,5	,5	87,9	,0	,5	,0	1,6	
	,191	1	,5	,5	88,4	,0	,5	,0	1,6	
	,191	1	,5	,5	88,9	,0	,5	,0	1,6	
	,193	1	,5	,5	89,5	,0	,5	,0	1,6	
	,194	1	,5	,5	90,0	,0	,5	,0	1,6	
	,194	1	,5	,5	90,5	,0	,5	,0	1,6	
	,194	1	,5	,5	91,1	,0	,5	,0	1,6	
	,195	1	,5	,5	91,6	,0	,5	,0	1,6	
	,196	1	,5	,5	92,1	,0	,5	,0	2,1	
	,197	1	,5	,5	92,6	,0	,5	,0	1,6	
	,202	1	,5	,5	93,2	,0	,5	,0	1,6	
	,202	1	,5	,5	93,7	,0	,5	,0	1,6	
	,207	1	,5	,5	94,2	,0	,5	,0	1,6	
	,207	1	,5	,5	94,7	,0	,5	,0	1,6	
	,209	1	,5	,5	95,3	,0	,5	,0	1,6	
	,222	1	,5	,5	95,8	,0	,5	,0	1,6	
	,223	1	,5	,5	96,3	,0	,5	,0	2,1	
	,223	1	,5	,5	96,8	,0	,6	,0	2,1	
	,223	1	,5	,5	97,4	,0	,5	,0	1,6	
	,226	1	,5	,5	97,9	,0	,5	,0	1,6	
	,238	1	,5	,5	98,4	,0	,5	,0	1,6	
	,240	1	,5	,5	98,9	,0	,6	,0	1,6	
	,255	1	,5	,5	99,5	,0	,5	,0	1,6	
	,269	1	,5	,5	100,0	,0	,5	,0	2,1	
	Total	190	100,0	100,0		,0	,0	100,0	100,0	

DESCRIPTIVES VARIABLES=RCAindex
  /STATISTICS=MEAN STDDEV VARIANCE RANGE MIN MAX KURTOSIS.


Descriptives


Notes	
Output Created	01-AUG-2021 15:57:38	
Comments		
Input	Data	C:\Users\Placido\Desktop\articolo atrofia e sottodurale cronico\controlli\controlli correlazioni.sav	
	Active Dataset	Dataset1	
	Filter	<none>	
	Weight	<none>	
	Split File	<none>	
	N of Rows in Working Data File	190	
Missing Value Handling	Definition of Missing	User defined missing values are treated as missing.	
	Cases Used	All non-missing data are used.	
Syntax	DESCRIPTIVES VARIABLES=RCAindex
  /STATISTICS=MEAN STDDEV VARIANCE RANGE MIN MAX KURTOSIS.	
Resources	Processor Time	00:00:00,00	
	Elapsed Time	00:00:00,01	


Descriptive Statistics	
	N	Range	Minimum	Maximum	Mean	Std. Deviation	
	Statistic	Statistic	Statistic	Statistic	Statistic	Statistic	
RCA index	190	,216	,053	,269	,13716	,045340	
Valid N (listwise)	190						

Descriptive Statistics	
	Variance	Kurtosis	
	Statistic	Statistic	Std. Error	
RCA index	,002	-,650	,351	
Valid N (listwise)				

*Nonparametric Tests: One Sample.
NPTESTS
  /ONESAMPLE TEST (FI IC CS Dtmax Age Sex RCAindex RCAaverage RCASTD)
  /MISSING SCOPE=ANALYSIS USERMISSING=EXCLUDE
  /CRITERIA ALPHA=0.05 CILEVEL=95.


Nonparametric Tests


Notes	
Output Created	01-AUG-2021 15:59:46	
Comments		
Input	Data	C:\Users\Placido\Desktop\articolo atrofia e sottodurale cronico\controlli\controlli correlazioni.sav	
	Active Dataset	Dataset1	
	Filter	<none>	
	Weight	<none>	
	Split File	<none>	
	N of Rows in Working Data File	190	
Syntax	NPTESTS
  /ONESAMPLE TEST (FI IC CS Dtmax Age Sex RCAindex RCAaverage RCASTD)
  /MISSING SCOPE=ANALYSIS USERMISSING=EXCLUDE
  /CRITERIA ALPHA=0.05 CILEVEL=95.	
Resources	Processor Time	00:00:00,34	
	Elapsed Time	00:00:00,43	


null : null


BAYES ONESAMPLE
  /MISSING SCOPE=ANALYSIS
  /CRITERIA CILEVEL=95
  /INFERENCE DISTRIBUTION=NORMAL VARIABLES=RCAindex ANALYSIS=POSTERIOR
  /PRIOR VARDIST=DIFFUSE MEANDIST=DIFFUSE.


Bayesian One-Sample


Notes	
Output Created	01-AUG-2021 16:00:58	
Comments		
Input	Data	C:\Users\Placido\Desktop\articolo atrofia e sottodurale cronico\controlli\controlli correlazioni.sav	
	Active Dataset	Dataset1	
	Filter	<none>	
	Weight	<none>	
	Split File	<none>	
	N of Rows in Working Data File	190	
Missing Value Handling	Definition of Missing	User-defined missing values are treated as missing.	
	Cases Used	Each statistic is based on all valid data for the analysis variable(s) used in computing the statistic.	
Weight Handling	not applicable	
Syntax	BAYES ONESAMPLE
  /MISSING SCOPE=ANALYSIS
  /CRITERIA CILEVEL=95
  /INFERENCE DISTRIBUTION=NORMAL VARIABLES=RCAindex ANALYSIS=POSTERIOR
  /PRIOR VARDIST=DIFFUSE MEANDIST=DIFFUSE.	
Resources	Processor Time	00:00:00,25	
	Elapsed Time	00:00:00,43	


Posterior Distribution Characterization for One-Sample Mean	
	N	Posterior	95% Credible Interval	
		Mode	Mean	Variance	Lower Bound	Upper Bound	
RCA index	190	,13716	,13716	,000	,13064	,14369	


EXAMINE VARIABLES=RCAindex
  /PLOT BOXPLOT STEMLEAF HISTOGRAM
  /COMPARE GROUPS
  /STATISTICS DESCRIPTIVES
  /CINTERVAL 95
  /MISSING LISTWISE
  /NOTOTAL.


Explore


Notes	
Output Created	01-AUG-2021 16:02:13	
Comments		
Input	Data	C:\Users\Placido\Desktop\articolo atrofia e sottodurale cronico\controlli\controlli correlazioni.sav	
	Active Dataset	Dataset1	
	Filter	<none>	
	Weight	<none>	
	Split File	<none>	
	N of Rows in Working Data File	190	
Missing Value Handling	Definition of Missing	User-defined missing values for dependent variables are treated as missing.	
	Cases Used	Statistics are based on cases with no missing values for any dependent variable or factor used.	
Syntax	EXAMINE VARIABLES=RCAindex
  /PLOT BOXPLOT STEMLEAF HISTOGRAM
  /COMPARE GROUPS
  /STATISTICS DESCRIPTIVES
  /CINTERVAL 95
  /MISSING LISTWISE
  /NOTOTAL.	
Resources	Processor Time	00:00:00,77	
	Elapsed Time	00:00:00,46	


Case Processing Summary	
	Cases	
	Valid	Missing	Total	
	N	Percent	N	Percent	N	Percent	
RCA index	190	100,0%	0	0,0%	190	100,0%	


Descriptives	
	Statistic	Std. Error	
RCA index	Mean	,13716	,003289	
	95% Confidence Interval for Mean	Lower Bound	,13068		
		Upper Bound	,14365		
	5% Trimmed Mean	,13567		
	Median	,13212		
	Variance	,002		
	Std. Deviation	,045340		
	Minimum	,053		
	Maximum	,269		
	Range	,216		
	Interquartile Range	,074		
	Skewness	,367	,176	
	Kurtosis	-,650	,351	


RCA index


RCA index Stem-and-Leaf Plot

 Frequency    Stem &  Leaf

     2,00        0 .  55
    13,00        0 .  6667777777777
    34,00        0 .  8888888888888888999999999999999999
    34,00        1 .  0000000000000000001111111111111111
    22,00        1 .  2222222233333333333333
    20,00        1 .  44444445555555555555
    25,00        1 .  6666666666666777777777777
    26,00        1 .  88888888888999999999999999
     5,00        2 .  00000
     7,00        2 .  2222233
     1,00        2 .  5
     1,00        2 .  6

 Stem width:  ,100
 Each leaf:        1 case(s)
