# Supplementary material for: Cortical atrophy in chronic subdural hematoma from ultra-structures to physical properties
Source: Sci Rep. 2023 Feb 28;13:3400. doi: 10.1038/s41598-023-30135-8 (PMC9975247; doi:10.1038/s41598-023-30135-8)
Supplement: Supplementary file 2 — Supplementary Information 2. [file 41598_2023_30135_MOESM2_ESM.doc]

GET DATA
  /TYPE=XLSX
  /FILE='C:\Users\Placido\Desktop\articolo atrofia e sottodurale cronico\casi.xlsx'
  /SHEET=name 'casi'
  /CELLRANGE=FULL
  /READNAMES=ON
  /DATATYPEMIN PERCENTAGE=95.0
  /HIDDEN IGNORE=YES.
EXECUTE.
DATASET NAME Dataset1 WINDOW=FRONT.
CORRELATIONS
  /VARIABLES=RCAindex Shiftpost30 KPSPostOp
  /PRINT=TWOTAIL NOSIG
  /MISSING=PAIRWISE.


Correlazioni


Note	
Output creato	01-JUL-2021 19:21:40	
Commenti		
Input	Dataset attivo	Dataset1	
	Filtro	<nessuno>	
	Peso	<nessuno>	
	File suddiviso	<nessuno>	
	N di righe nel file di dati di lavoro	190	
Gestione valori mancanti	Definizione di mancante	I valori mancanti definiti dall'utente vengono trattati come mancanti.	
	Casi utilizzati	Le statistiche per ciascuna coppia di variabili sono basate su tutti i casi con dei dati validi per tale coppia.	
Sintassi	CORRELATIONS
  /VARIABLES=RCAindex Shiftpost30 KPSPostOp
  /PRINT=TWOTAIL NOSIG
  /MISSING=PAIRWISE.	
Risorse	Tempo processore	00:00:00,00	
	Tempo trascorso	00:00:00,00	


[Dataset1] 


Correlazioni	
	RCA index	Shift post 30	KPS PostOp	
RCA index	Correlazione di Pearson	1	-,090	-,333**	
	Sign. (a due code)		,214	,000	
	N	190	190	190	
Shift post 30	Correlazione di Pearson	-,090	1	-,046	
	Sign. (a due code)	,214		,531	
	N	190	190	190	
KPS PostOp	Correlazione di Pearson	-,333**	-,046	1	
	Sign. (a due code)	,000	,531		
	N	190	190	190	

**. La correlazione è significativa a livello 0,01 (a due code).	

CORRELATIONS
  /VARIABLES=RCAindex Shiftpost30 KPSPostOp Shiftpost90 MDPost30 MDPost90
  /PRINT=TWOTAIL NOSIG
  /STATISTICS DESCRIPTIVES
  /MISSING=PAIRWISE.


Correlazioni


Note	
Output creato	01-JUL-2021 19:23:18	
Commenti		
Input	Dataset attivo	Dataset1	
	Filtro	<nessuno>	
	Peso	<nessuno>	
	File suddiviso	<nessuno>	
	N di righe nel file di dati di lavoro	190	
Gestione valori mancanti	Definizione di mancante	I valori mancanti definiti dall'utente vengono trattati come mancanti.	
	Casi utilizzati	Le statistiche per ciascuna coppia di variabili sono basate su tutti i casi con dei dati validi per tale coppia.	
Sintassi	CORRELATIONS
  /VARIABLES=RCAindex Shiftpost30 KPSPostOp Shiftpost90 MDPost30 MDPost90
  /PRINT=TWOTAIL NOSIG
  /STATISTICS DESCRIPTIVES
  /MISSING=PAIRWISE.	
Risorse	Tempo processore	00:00:00,02	
	Tempo trascorso	00:00:00,02	


Statistica descrittiva	
	Media	Deviazione std.	N	
RCA index	,176912157809137	,034633202786244	190	
Shift post 30	3,312631578947367	2,510833336452650	190	
KPS PostOp	86,63	9,440	190	
Shift post 90	1,88	1,277	190	
MDPost 30	10,682631578947370	5,372806644502401	190	
MDPost 90	4,308315789473684	3,136305970061689	190	


Correlazioni	
	RCA index	Shift post 30	KPS PostOp	Shift post 90	
RCA index	Correlazione di Pearson	1	-,090	-,333**	,026	
	Sign. (a due code)		,214	,000	,723	
	N	190	190	190	190	
Shift post 30	Correlazione di Pearson	-,090	1	-,046	,253**	
	Sign. (a due code)	,214		,531	,000	
	N	190	190	190	190	
KPS PostOp	Correlazione di Pearson	-,333**	-,046	1	,010	
	Sign. (a due code)	,000	,531		,892	
	N	190	190	190	190	
Shift post 90	Correlazione di Pearson	,026	,253**	,010	1	
	Sign. (a due code)	,723	,000	,892		
	N	190	190	190	190	
MDPost 30	Correlazione di Pearson	,283**	,348**	-,212**	,166*	
	Sign. (a due code)	,000	,000	,003	,022	
	N	190	190	190	190	
MDPost 90	Correlazione di Pearson	,059	,177*	-,117	,133	
	Sign. (a due code)	,419	,014	,108	,068	
	N	190	190	190	190	

Correlazioni	
	MDPost 30	MDPost 90	
RCA index	Correlazione di Pearson	,283**	,059	
	Sign. (a due code)	,000	,419	
	N	190	190	
Shift post 30	Correlazione di Pearson	,348**	,177*	
	Sign. (a due code)	,000	,014	
	N	190	190	
KPS PostOp	Correlazione di Pearson	-,212**	-,117	
	Sign. (a due code)	,003	,108	
	N	190	190	
Shift post 90	Correlazione di Pearson	,166*	,133	
	Sign. (a due code)	,022	,068	
	N	190	190	
MDPost 30	Correlazione di Pearson	1	,395**	
	Sign. (a due code)		,000	
	N	190	190	
MDPost 90	Correlazione di Pearson	,395**	1	
	Sign. (a due code)	,000		
	N	190	190	

**. La correlazione è significativa a livello 0,01 (a due code).	
*. La correlazione è significativa a livello 0,05 (a due code).	

CORRELATIONS
  /VARIABLES=RCAindex Age
  /PRINT=TWOTAIL NOSIG
  /STATISTICS DESCRIPTIVES
  /MISSING=PAIRWISE.


Correlazioni


Note	
Output creato	01-JUL-2021 19:29:31	
Commenti		
Input	Dataset attivo	Dataset1	
	Filtro	<nessuno>	
	Peso	<nessuno>	
	File suddiviso	<nessuno>	
	N di righe nel file di dati di lavoro	190	
Gestione valori mancanti	Definizione di mancante	I valori mancanti definiti dall'utente vengono trattati come mancanti.	
	Casi utilizzati	Le statistiche per ciascuna coppia di variabili sono basate su tutti i casi con dei dati validi per tale coppia.	
Sintassi	CORRELATIONS
  /VARIABLES=RCAindex Age
  /PRINT=TWOTAIL NOSIG
  /STATISTICS DESCRIPTIVES
  /MISSING=PAIRWISE.	
Risorse	Tempo processore	00:00:00,00	
	Tempo trascorso	00:00:00,02	


Statistica descrittiva	
	Media	Deviazione std.	N	
RCA index	,176912157809137	,034633202786244	190	
Age	78,56	7,641	190	


Correlazioni	
	RCA index	Age	
RCA index	Correlazione di Pearson	1	,512**	
	Sign. (a due code)		,000	
	N	190	190	
Age	Correlazione di Pearson	,512**	1	
	Sign. (a due code)	,000		
	N	190	190	

**. La correlazione è significativa a livello 0,01 (a due code).	

CORRELATIONS
  /VARIABLES=RCAindex Age
  /PRINT=TWOTAIL NOSIG
  /STATISTICS DESCRIPTIVES
  /MISSING=PAIRWISE.


Correlazioni


Note	
Output creato	01-JUL-2021 19:31:43	
Commenti		
Input	Dataset attivo	Dataset1	
	Filtro	<nessuno>	
	Peso	<nessuno>	
	File suddiviso	<nessuno>	
	N di righe nel file di dati di lavoro	190	
Gestione valori mancanti	Definizione di mancante	I valori mancanti definiti dall'utente vengono trattati come mancanti.	
	Casi utilizzati	Le statistiche per ciascuna coppia di variabili sono basate su tutti i casi con dei dati validi per tale coppia.	
Sintassi	CORRELATIONS
  /VARIABLES=RCAindex Age
  /PRINT=TWOTAIL NOSIG
  /STATISTICS DESCRIPTIVES
  /MISSING=PAIRWISE.	
Risorse	Tempo processore	00:00:00,02	
	Tempo trascorso	00:00:00,01	


Statistica descrittiva	
	Media	Deviazione std.	N	
RCA index	,176912157809137	,034633202786244	190	
Age	78,56	7,641	190	


Correlazioni	
	RCA index	Age	
RCA index	Correlazione di Pearson	1	,512**	
	Sign. (a due code)		,000	
	N	190	190	
Age	Correlazione di Pearson	,512**	1	
	Sign. (a due code)	,000		
	N	190	190	

**. La correlazione è significativa a livello 0,01 (a due code).	

* Builder di grafico.
GGRAPH
  /GRAPHDATASET NAME="graphdataset" VARIABLES=RCAindex Age MISSING=LISTWISE REPORTMISSING=NO
  /GRAPHSPEC SOURCE=INLINE
  /FITLINE TOTAL=YES.
BEGIN GPL
  SOURCE: s=userSource(id("graphdataset"))
  DATA: RCAindex=col(source(s), name("RCAindex"))
  DATA: Age=col(source(s), name("Age"))
  GUIDE: axis(dim(1), label("RCA index"))
  GUIDE: axis(dim(2), label("Age"))
  GUIDE: text.title(label("Dispersione semplice con curva di adattamento di Age per RCA index"))
  ELEMENT: point(position(RCAindex*Age))
END GPL.


GGraph


Note	
Output creato	01-JUL-2021 19:35:18	
Commenti		
Input	Dataset attivo	Dataset1	
	Filtro	<nessuno>	
	Peso	<nessuno>	
	File suddiviso	<nessuno>	
	N di righe nel file di dati di lavoro	190	
Sintassi	GGRAPH
  /GRAPHDATASET NAME="graphdataset" VARIABLES=RCAindex Age MISSING=LISTWISE REPORTMISSING=NO
  /GRAPHSPEC SOURCE=INLINE
  /FITLINE TOTAL=YES.
BEGIN GPL
  SOURCE: s=userSource(id("graphdataset"))
  DATA: RCAindex=col(source(s), name("RCAindex"))
  DATA: Age=col(source(s), name("Age"))
  GUIDE: axis(dim(1), label("RCA index"))
  GUIDE: axis(dim(2), label("Age"))
  GUIDE: text.title(label("Dispersione semplice con curva di adattamento di Age per RCA index"))
  ELEMENT: point(position(RCAindex*Age))
END GPL.	
Risorse	Tempo processore	00:00:01,24	
	Tempo trascorso	00:00:00,47	
