# Supplementary material for: Cortical atrophy in chronic subdural hematoma from ultra-structures to physical properties
Source: Sci Rep. 2023 Feb 28;13:3400. doi: 10.1038/s41598-023-30135-8 (PMC9975247; doi:10.1038/s41598-023-30135-8)
Supplement: Supplementary file 3 — Supplementary Information 3. [file 41598_2023_30135_MOESM3_ESM.doc]

GET DATA
  /TYPE=XLSX
  /FILE='C:\Users\Placido\Desktop\articolo atrofia e sottodurale cronico\confronto\nuova analisi\confronto casi controlli.xlsx'
  /SHEET=name 'Foglio1'
  /CELLRANGE=FULL
  /READNAMES=ON
  /DATATYPEMIN PERCENTAGE=95.0
  /HIDDEN IGNORE=YES.
EXECUTE.
DATASET NAME Dataset1 WINDOW=FRONT.
BOOTSTRAP
  /SAMPLING METHOD=SIMPLE
  /VARIABLES TARGET=RCAIndex INPUT=Group
  /CRITERIA CILEVEL=95 CITYPE=PERCENTILE  NSAMPLES=1000
  /MISSING USERMISSING=EXCLUDE.


Bootstrap


Notes	
Output Created	04-AUG-2021 20:50:51	
Comments		
Input	Active Dataset	Dataset1	
	Filter	<none>	
	Weight	<none>	
	Split File	<none>	
Syntax	BOOTSTRAP
  /SAMPLING METHOD=SIMPLE
  /VARIABLES TARGET=RCAIndex INPUT=Group
  /CRITERIA CILEVEL=95 CITYPE=PERCENTILE  NSAMPLES=1000
  /MISSING USERMISSING=EXCLUDE.	
Resources	Processor Time	00:00:00,02	
	Elapsed Time	00:00:00,03	


[Dataset1] 


Bootstrap Specifications	
Sampling Method	Simple	
Number of Samples	1000	
Confidence Interval Level	95,0%	
Confidence Interval Type	Percentile	

T-TEST GROUPS=Group(0 1)
  /MISSING=ANALYSIS
  /VARIABLES=RCAIndex
  /CRITERIA=CI(.95).


T-Test


Notes	
Output Created	04-AUG-2021 20:50:51	
Comments		
Input	Active Dataset	Dataset1	
	Filter	<none>	
	Weight	<none>	
	Split File	<none>	
	N of Rows in Working Data File	240707	
Missing Value Handling	Definition of Missing	User defined missing values are treated as missing.	
	Cases Used	Statistics for each analysis are based on the cases with no missing or out-of-range data for any variable in the analysis.	
Syntax	T-TEST GROUPS=Group(0 1)
  /MISSING=ANALYSIS
  /VARIABLES=RCAIndex
  /CRITERIA=CI(.95).	
Resources	Processor Time	00:00:02,48	
	Elapsed Time	00:00:02,82	


Group Statistics	
	Group	Statistic	Bootstrap	
			Bias	Std. Error	95% Confidence Interval	
					Lower	Upper	
RCAIndex	Control	N	190					
		Mean	,13716	,00008	,00328	,13097	,14356	
		Std. Deviation	,045340	-,000077	,001887	,041793	,048993	
		Std. Error Mean	,003289					
	Case	N	190					
		Mean	,17691	-,00009	,00241	,17215	,18147	
		Std. Deviation	,034633	-,000094	,001559	,031606	,037718	
		Std. Error Mean	,002513					


Independent Samples Test	
	Levene's Test for Equality of Variances	t-test for Equality of Means	
	F	Sig.	t	df	
					
RCAIndex	Equal variances assumed	22,184	,000	-9,603	378	
	Equal variances not assumed			-9,603	353,538	

Independent Samples Test	
	t-test for Equality of Means	
	Sig. (2-tailed)	Mean Difference	Std. Error Difference	
				
RCAIndex	Equal variances assumed	,000	-,039749	,004139	
	Equal variances not assumed	,000	-,039749	,004139	

Independent Samples Test	
	t-test for Equality of Means	
	95% Confidence Interval of the Difference	
	Lower	Upper	
RCAIndex	Equal variances assumed	-,047887	-,031610	
	Equal variances not assumed	-,047889	-,031608	


Bootstrap for Independent Samples Test	
	Mean Difference	Bootstrap	
		Bias	Std. Error	Sig. (2-tailed)	
					
RCAIndex	Equal variances assumed	-,039749	,000169	,004076	,001	
	Equal variances not assumed	-,039749	,000169	,004076	,001	

Bootstrap for Independent Samples Test	
	Bootstrap	
	95% Confidence Interval	
	Lower	Upper	
RCAIndex	Equal variances assumed	-,047428	-,031691	
	Equal variances not assumed	-,047428	-,031691	

ONEWAY RCAIndex BY Group
  /STATISTICS DESCRIPTIVES EFFECTS HOMOGENEITY
  /MISSING ANALYSIS
  /POSTHOC=BONFERRONI ALPHA(0.05).


Oneway


Notes	
Output Created	04-AUG-2021 20:53:00	
Comments		
Input	Active Dataset	Dataset1	
	Filter	<none>	
	Weight	<none>	
	Split File	<none>	
	N of Rows in Working Data File	380	
Missing Value Handling	Definition of Missing	User-defined missing values are treated as missing.	
	Cases Used	Statistics for each analysis are based on cases with no missing data for any variable in the analysis.	
Syntax	ONEWAY RCAIndex BY Group
  /STATISTICS DESCRIPTIVES EFFECTS HOMOGENEITY
  /MISSING ANALYSIS
  /POSTHOC=BONFERRONI ALPHA(0.05).	
Resources	Processor Time	00:00:00,00	
	Elapsed Time	00:00:00,00	


Warnings	
Post hoc tests are not performed for RCAIndex because there are fewer than three groups.	


Descriptives	
RCAIndex  	
	N	Mean	Std. Deviation	Std. Error	95% Confidence Interval for Mean	
					Lower Bound	
Control	190	,13716	,045340	,003289	,13068	
Case	190	,17691	,034633	,002513	,17196	
Total	380	,15704	,044937	,002305	,15251	
Model	Fixed Effects			,040343	,002070	,15297	
	Random Effects				,019874	-,09549	

Descriptives	
RCAIndex  	
	95% Confidence Interval for Mean	Minimum	Maximum	Between- Component Variance	
	Upper Bound				
Control	,14365	,053	,269		
Case	,18187	,100	,265		
Total	,16157	,053	,269		
Model	Fixed Effects	,16111				
	Random Effects	,40956			,000781	


Test of Homogeneity of Variances	
	Levene Statistic	df1	df2	Sig.	
RCAIndex	Based on Mean	22,184	1	378	,000	
	Based on Median	20,345	1	378	,000	
	Based on Median and with adjusted df	20,345	1	363,365	,000	
	Based on trimmed mean	21,510	1	378	,000	


ANOVA	
RCAIndex  	
	Sum of Squares	df	Mean Square	F	Sig.	
Between Groups	,150	1	,150	92,219	,000	
Within Groups	,615	378	,002			
Total	,765	379				

ROC RCAIndex BY Group (0)
  /PLOT=CURVE(REFERENCE)
  /PRINT=SE COORDINATES
  /CRITERIA=CUTOFF(INCLUDE) TESTPOS(SMALL) DISTRIBUTION(FREE) CI(95)
  /MISSING=EXCLUDE.


ROC Curve


Notes	
Output Created	04-AUG-2021 20:55:05	
Comments		
Input	Active Dataset	Dataset1	
	Filter	<none>	
	Weight	<none>	
	Split File	<none>	
	N of Rows in Working Data File	380	
Missing Value Handling	Definition of Missing	User-defined missing values are treated as missing.	
	Cases Used	Statistics are based on all cases with valid data for all variables in the analysis.	
Syntax	ROC RCAIndex BY Group (0)
  /PLOT=CURVE(REFERENCE)
  /PRINT=SE COORDINATES
  /CRITERIA=CUTOFF(INCLUDE) TESTPOS(SMALL) DISTRIBUTION(FREE) CI(95)
  /MISSING=EXCLUDE.	
Resources	Processor Time	00:00:00,61	
	Elapsed Time	00:00:00,39	


Case Processing Summary	
Group	Valid N (listwise)	
Positive	190	
Negative	190	


Area Under the Curve	
Test Result Variable(s):   RCAIndex  	
Area	Std. Error	Asymptotic Sig.	Asymptotic 95% Confidence Interval	
			Lower Bound	Upper Bound	
,749	,025	,000	,701	,798	


Coordinates of the Curve	
Test Result Variable(s):   RCAIndex  	
Positive if Less Than or Equal To	Sensitivity	1 - Specificity	
,00000	,000	,000	
,05588	,005	,000	
,06004	,011	,000	
,06479	,016	,000	
,06884	,021	,000	
,07032	,026	,000	
,07132	,032	,000	
,07240	,037	,000	
,07335	,042	,000	
,07464	,047	,000	
,07616	,053	,000	
,07704	,058	,000	
,07810	,063	,000	
,07902	,068	,000	
,07969	,074	,000	
,07999	,079	,000	
,08077	,084	,000	
,08144	,089	,000	
,08168	,095	,000	
,08199	,100	,000	
,08209	,105	,000	
,08222	,111	,000	
,08270	,116	,000	
,08326	,121	,000	
,08350	,126	,000	
,08399	,132	,000	
,08480	,137	,000	
,08537	,142	,000	
,08570	,147	,000	
,08735	,153	,000	
,08919	,158	,000	
,09014	,163	,000	
,09084	,168	,000	
,09098	,174	,000	
,09139	,179	,000	
,09191	,184	,000	
,09225	,189	,000	
,09253	,195	,000	
,09272	,200	,000	
,09283	,205	,000	
,09303	,211	,000	
,09362	,216	,000	
,09466	,221	,000	
,09576	,226	,000	
,09652	,232	,000	
,09726	,237	,000	
,09825	,242	,000	
,09878	,247	,000	
,09893	,253	,000	
,09938	,258	,000	
,10046	,258	,011	
,10139	,263	,011	
,10160	,268	,011	
,10191	,274	,011	
,10242	,279	,011	
,10285	,284	,011	
,10334	,289	,011	
,10369	,295	,011	
,10387	,300	,011	
,10452	,305	,011	
,10504	,311	,011	
,10548	,316	,011	
,10608	,321	,011	
,10629	,326	,011	
,10659	,332	,011	
,10817	,337	,011	
,10957	,342	,011	
,10978	,347	,011	
,10999	,353	,011	
,11035	,358	,011	
,11086	,358	,026	
,11159	,363	,026	
,11261	,368	,026	
,11321	,374	,026	
,11373	,379	,026	
,11421	,384	,026	
,11442	,389	,026	
,11464	,395	,026	
,11468	,400	,026	
,11501	,405	,026	
,11601	,411	,026	
,11715	,416	,026	
,11772	,421	,026	
,11804	,426	,026	
,11901	,432	,026	
,11980	,437	,026	
,12062	,437	,042	
,12216	,442	,042	
,12327	,447	,042	
,12431	,453	,042	
,12505	,458	,042	
,12571	,458	,058	
,12651	,463	,058	
,12707	,463	,074	
,12781	,468	,074	
,12890	,474	,074	
,12988	,479	,074	
,13016	,484	,074	
,13040	,489	,074	
,13068	,495	,074	
,13085	,495	,084	
,13115	,495	,100	
,13147	,495	,116	
,13212	,500	,116	
,13305	,505	,116	
,13368	,511	,116	
,13395	,516	,116	
,13397	,521	,116	
,13402	,521	,132	
,13430	,521	,147	
,13455	,526	,147	
,13521	,532	,147	
,13703	,537	,147	
,13876	,542	,147	
,14009	,553	,147	
,14088	,558	,147	
,14209	,558	,158	
,14355	,558	,174	
,14468	,558	,184	
,14593	,558	,195	
,14692	,558	,211	
,14797	,558	,226	
,14873	,558	,242	
,14922	,563	,242	
,14950	,568	,242	
,14964	,574	,242	
,14976	,579	,242	
,14990	,584	,242	
,15004	,589	,242	
,15071	,589	,258	
,15163	,595	,258	
,15205	,600	,258	
,15217	,605	,258	
,15224	,605	,274	
,15271	,605	,289	
,15315	,611	,289	
,15365	,616	,289	
,15425	,621	,289	
,15453	,626	,289	
,15513	,632	,289	
,15562	,632	,305	
,15573	,637	,305	
,15595	,642	,305	
,15733	,647	,305	
,15863	,647	,321	
,15888	,647	,337	
,15911	,647	,353	
,15921	,653	,353	
,15940	,658	,353	
,15987	,658	,368	
,16043	,663	,368	
,16079	,668	,368	
,16108	,674	,368	
,16157	,679	,368	
,16234	,684	,368	
,16358	,684	,384	
,16461	,689	,384	
,16531	,695	,384	
,16592	,700	,384	
,16630	,705	,384	
,16654	,705	,400	
,16707	,711	,400	
,16767	,716	,400	
,16792	,716	,416	
,16849	,721	,416	
,16952	,726	,416	
,17018	,732	,416	
,17033	,732	,426	
,17046	,732	,437	
,17079	,737	,437	
,17124	,737	,453	
,17152	,742	,453	
,17187	,742	,468	
,17215	,747	,468	
,17258	,753	,468	
,17317	,758	,468	
,17354	,758	,479	
,17391	,763	,479	
,17431	,768	,479	
,17485	,768	,489	
,17553	,768	,500	
,17654	,768	,516	
,17734	,768	,532	
,17779	,774	,532	
,17846	,779	,532	
,17883	,784	,532	
,17935	,789	,532	
,18042	,789	,542	
,18128	,789	,553	
,18243	,795	,553	
,18331	,800	,553	
,18339	,800	,568	
,18358	,805	,568	
,18404	,811	,568	
,18454	,816	,568	
,18487	,821	,568	
,18516	,826	,568	
,18540	,832	,568	
,18640	,832	,584	
,18740	,837	,584	
,18781	,842	,584	
,18827	,842	,600	
,18875	,842	,616	
,18915	,842	,632	
,18954	,847	,632	
,18994	,847	,642	
,19013	,853	,642	
,19034	,858	,642	
,19072	,863	,642	
,19111	,863	,658	
,19122	,868	,658	
,19128	,874	,658	
,19137	,879	,658	
,19144	,884	,658	
,19192	,889	,658	
,19240	,889	,668	
,19258	,889	,684	
,19317	,895	,684	
,19371	,900	,684	
,19390	,900	,695	
,19401	,905	,695	
,19438	,911	,695	
,19522	,916	,695	
,19631	,921	,695	
,19750	,926	,695	
,19823	,926	,705	
,19925	,926	,716	
,20031	,926	,732	
,20078	,926	,747	
,20156	,926	,763	
,20216	,932	,763	
,20442	,937	,763	
,20668	,942	,763	
,20705	,942	,779	
,20761	,947	,779	
,20832	,947	,795	
,20922	,953	,795	
,21060	,953	,811	
,21197	,953	,826	
,21257	,953	,842	
,21335	,953	,853	
,21630	,953	,868	
,21971	,953	,884	
,22150	,953	,895	
,22236	,958	,895	
,22289	,963	,895	
,22327	,968	,895	
,22360	,974	,895	
,22403	,974	,905	
,22491	,974	,921	
,22575	,979	,921	
,22600	,979	,937	
,22689	,979	,953	
,23292	,979	,968	
,23890	,984	,968	
,24691	,989	,968	
,25451	,989	,984	
,26001	,995	,984	
,26712	,995	1,000	
1,00000	1,000	1,000	
