# Supplementary material for: Cortical atrophy in chronic subdural hematoma from ultra-structures to physical properties
Source: Sci Rep. 2023 Feb 28;13:3400. doi: 10.1038/s41598-023-30135-8 (PMC9975247; doi:10.1038/s41598-023-30135-8)
Supplement: Supplementary file 4 — Supplementary Information 4. [file 41598_2023_30135_MOESM4_ESM.doc]

GET DATA
  /TYPE=XLSX
  /FILE='C:\Users\Placido\Desktop\articolo atrofia e sottodurale cronico\casi\casi.xlsx'
  /SHEET=name 'casi'
  /CELLRANGE=FULL
  /READNAMES=ON
  /DATATYPEMIN PERCENTAGE=95.0
  /HIDDEN IGNORE=YES.
EXECUTE.
DATASET NAME Dataset1 WINDOW=FRONT.
SET TLook=None Small=0.0001 SUMMARY=None THREADS=AUTO TFit=Both DIGITGROUPING=No LEADZERO=No TABLERENDER=light.
FREQUENCIES VARIABLES=Age Sex RCAindex MDPreop MDPost30 MDPost90 Shiftpre Shiftpost30 Shiftpost90
    KPSPreOp KPSPostOp Side
  /NTILES=4
  /STATISTICS=STDDEV VARIANCE RANGE MINIMUM MAXIMUM MEAN MEDIAN
  /BARCHART FREQ
  /ORDER=ANALYSIS.


Frequencies


Notes	
Output Created	01-AUG-2021 23:47:10	
Comments		
Input	Active Dataset	Dataset1	
	Filter	<none>	
	Weight	<none>	
	Split File	<none>	
	N of Rows in Working Data File	190	
Missing Value Handling	Definition of Missing	User-defined missing values are treated as missing.	
	Cases Used	Statistics are based on all cases with valid data.	
Syntax	FREQUENCIES VARIABLES=Age Sex RCAindex MDPreop MDPost30 MDPost90 Shiftpre Shiftpost30 Shiftpost90
    KPSPreOp KPSPostOp Side
  /NTILES=4
  /STATISTICS=STDDEV VARIANCE RANGE MINIMUM MAXIMUM MEAN MEDIAN
  /BARCHART FREQ
  /ORDER=ANALYSIS.	
Resources	Processor Time	00:00:03,84	
	Elapsed Time	00:00:01,91	


[Dataset1] 


Statistics	
	Age	Sex	RCA index	MDPreop	MDPost 30	MDPost 90	
N	Valid	190	190	190	190	190	190	
	Missing	0	0	0	0	0	0	
Mean	78,56		,17691	22,96211	10,68263	4,30832	
Median	80,00		,17553	22,00000	11,20000	4,00000	
Std. Deviation	7,641		,034633	5,376919	5,372807	3,136306	
Variance	58,386		,001	28,911	28,867	9,836	
Range	37		,166	28,300	22,700	15,000	
Minimum	60		,100	10,000	,000	,000	
Maximum	97		,265	38,300	22,700	15,000	
Percentiles	25	73,00		,15012	19,00000	6,00000	1,00000	
	50	80,00		,17553	22,00000	11,20000	4,00000	
	75	84,00		,20110	26,85000	14,62500	6,00000	

Statistics	
	Shift pre	Shift post 30	Shift post 90	KPS PreOp	KPS PostOp	
N	Valid	190	190	190	190	190	
	Missing	0	0	0	0	0	
Mean	8,96579	3,31263	1,88	58,16	86,63	
Median	8,00000	3,00000	1,00	60,00	90,00	
Std. Deviation	3,623284	2,510833	1,277	14,412	9,440	
Variance	13,128	6,304	1,631	207,700	89,123	
Range	18,100	9,000	4	80	50	
Minimum	2,000	,000	0	10	50	
Maximum	20,100	9,000	4	90	100	
Percentiles	25	6,57500	1,00000	1,00	57,50	80,00	
	50	8,00000	3,00000	1,00	60,00	90,00	
	75	11,00000	5,30000	3,00	60,00	90,00	

Statistics	
	Side	
N	Valid	190	
	Missing	0	
Mean		
Median		
Std. Deviation		
Variance		
Range		
Minimum		
Maximum		
Percentiles	25		
	50		
	75		


Frequency Table


Age	
	Frequency	Percent	Valid Percent	Cumulative Percent	
Valid	60	3	1,6	1,6	1,6	
	61	3	1,6	1,6	3,2	
	62	2	1,1	1,1	4,2	
	63	5	2,6	2,6	6,8	
	64	2	1,1	1,1	7,9	
	66	2	1,1	1,1	8,9	
	69	3	1,6	1,6	10,5	
	70	3	1,6	1,6	12,1	
	71	9	4,7	4,7	16,8	
	72	6	3,2	3,2	20,0	
	73	11	5,8	5,8	25,8	
	74	5	2,6	2,6	28,4	
	75	6	3,2	3,2	31,6	
	76	7	3,7	3,7	35,3	
	77	5	2,6	2,6	37,9	
	78	13	6,8	6,8	44,7	
	79	6	3,2	3,2	47,9	
	80	13	6,8	6,8	54,7	
	81	8	4,2	4,2	58,9	
	82	11	5,8	5,8	64,7	
	83	9	4,7	4,7	69,5	
	84	13	6,8	6,8	76,3	
	85	31	16,3	16,3	92,6	
	87	3	1,6	1,6	94,2	
	88	3	1,6	1,6	95,8	
	90	2	1,1	1,1	96,8	
	96	3	1,6	1,6	98,4	
	97	3	1,6	1,6	100,0	
	Total	190	100,0	100,0		


Sex	
	Frequency	Percent	Valid Percent	Cumulative Percent	
Valid	f	74	38,9	38,9	38,9	
	m	116	61,1	61,1	100,0	
	Total	190	100,0	100,0		


RCA index	
	Frequency	Percent	Valid Percent	Cumulative Percent	
Valid	,100	2	1,1	1,1	1,1	
	,111	3	1,6	1,6	2,6	
	,120	3	1,6	1,6	4,2	
	,125	3	1,6	1,6	5,8	
	,127	3	1,6	1,6	7,4	
	,131	2	1,1	1,1	8,4	
	,131	3	1,6	1,6	10,0	
	,131	3	1,6	1,6	11,6	
	,134	3	1,6	1,6	13,2	
	,134	3	1,6	1,6	14,7	
	,141	2	1,1	1,1	15,8	
	,143	3	1,6	1,6	17,4	
	,144	2	1,1	1,1	18,4	
	,146	2	1,1	1,1	19,5	
	,146	3	1,6	1,6	21,1	
	,148	3	1,6	1,6	22,6	
	,148	3	1,6	1,6	24,2	
	,150	3	1,6	1,6	25,8	
	,152	3	1,6	1,6	27,4	
	,152	3	1,6	1,6	28,9	
	,156	3	1,6	1,6	30,5	
	,159	3	1,6	1,6	32,1	
	,159	3	1,6	1,6	33,7	
	,159	3	1,6	1,6	35,3	
	,160	3	1,6	1,6	36,8	
	,163	3	1,6	1,6	38,4	
	,167	3	1,6	1,6	40,0	
	,168	3	1,6	1,6	41,6	
	,170	2	1,1	1,1	42,6	
	,170	2	1,1	1,1	43,7	
	,171	3	1,6	1,6	45,3	
	,172	3	1,6	1,6	46,8	
	,173	2	1,1	1,1	47,9	
	,174	2	1,1	1,1	48,9	
	,175	2	1,1	1,1	50,0	
	,176	3	1,6	1,6	51,6	
	,177	3	1,6	1,6	53,2	
	,180	2	1,1	1,1	54,2	
	,181	2	1,1	1,1	55,3	
	,183	3	1,6	1,6	56,8	
	,185	3	1,6	1,6	58,4	
	,188	3	1,6	1,6	60,0	
	,188	3	1,6	1,6	61,6	
	,189	3	1,6	1,6	63,2	
	,190	2	1,1	1,1	64,2	
	,191	3	1,6	1,6	65,8	
	,192	2	1,1	1,1	66,8	
	,192	3	1,6	1,6	68,4	
	,194	2	1,1	1,1	69,5	
	,198	2	1,1	1,1	70,5	
	,198	2	1,1	1,1	71,6	
	,200	3	1,6	1,6	73,2	
	,200	3	1,6	1,6	74,7	
	,201	3	1,6	1,6	76,3	
	,207	3	1,6	1,6	77,9	
	,208	3	1,6	1,6	79,5	
	,210	3	1,6	1,6	81,1	
	,211	3	1,6	1,6	82,6	
	,213	3	1,6	1,6	84,2	
	,213	2	1,1	1,1	85,3	
	,214	3	1,6	1,6	86,8	
	,219	3	1,6	1,6	88,4	
	,221	2	1,1	1,1	89,5	
	,224	2	1,1	1,1	90,5	
	,224	3	1,6	1,6	92,1	
	,226	3	1,6	1,6	93,7	
	,226	3	1,6	1,6	95,3	
	,228	3	1,6	1,6	96,8	
	,254	3	1,6	1,6	98,4	
	,265	3	1,6	1,6	100,0	
	Total	190	100,0	100,0		


MDPreop	
	Frequency	Percent	Valid Percent	Cumulative Percent	
Valid	10,000	3	1,6	1,6	1,6	
	13,200	3	1,6	1,6	3,2	
	14,000	3	1,6	1,6	4,7	
	15,000	4	2,1	2,1	6,8	
	17,000	3	1,6	1,6	8,4	
	17,400	5	2,6	2,6	11,1	
	18,000	15	7,9	7,9	18,9	
	18,300	3	1,6	1,6	20,5	
	18,800	3	1,6	1,6	22,1	
	19,000	6	3,2	3,2	25,3	
	19,500	3	1,6	1,6	26,8	
	20,000	20	10,5	10,5	37,4	
	20,300	3	1,6	1,6	38,9	
	20,400	3	1,6	1,6	40,5	
	21,000	5	2,6	2,6	43,2	
	21,700	3	1,6	1,6	44,7	
	22,000	12	6,3	6,3	51,1	
	22,200	3	1,6	1,6	52,6	
	22,500	6	3,2	3,2	55,8	
	22,600	3	1,6	1,6	57,4	
	23,000	3	1,6	1,6	58,9	
	23,200	3	1,6	1,6	60,5	
	24,300	3	1,6	1,6	62,1	
	24,500	2	1,1	1,1	63,2	
	25,000	12	6,3	6,3	69,5	
	26,000	5	2,6	2,6	72,1	
	26,100	3	1,6	1,6	73,7	
	26,800	3	1,6	1,6	75,3	
	27,000	5	2,6	2,6	77,9	
	28,000	4	2,1	2,1	80,0	
	28,100	3	1,6	1,6	81,6	
	29,000	3	1,6	1,6	83,2	
	30,000	17	8,9	8,9	92,1	
	31,000	6	3,2	3,2	95,3	
	31,700	2	1,1	1,1	96,3	
	32,000	2	1,1	1,1	97,4	
	34,000	2	1,1	1,1	98,4	
	38,300	3	1,6	1,6	100,0	
	Total	190	100,0	100,0		


MDPost 30	
	Frequency	Percent	Valid Percent	Cumulative Percent	
Valid	,000	11	5,8	5,8	5,8	
	3,000	10	5,3	5,3	11,1	
	4,000	3	1,6	1,6	12,6	
	5,000	12	6,3	6,3	18,9	
	6,000	16	8,4	8,4	27,4	
	7,800	3	1,6	1,6	28,9	
	7,900	3	1,6	1,6	30,5	
	8,000	13	6,8	6,8	37,4	
	8,800	2	1,1	1,1	38,4	
	9,000	3	1,6	1,6	40,0	
	10,000	8	4,2	4,2	44,2	
	10,700	3	1,6	1,6	45,8	
	11,000	8	4,2	4,2	50,0	
	11,400	3	1,6	1,6	51,6	
	11,800	2	1,1	1,1	52,6	
	12,000	4	2,1	2,1	54,7	
	12,100	2	1,1	1,1	55,8	
	12,400	5	2,6	2,6	58,4	
	12,500	3	1,6	1,6	60,0	
	13,000	11	5,8	5,8	65,8	
	13,700	3	1,6	1,6	67,4	
	14,000	13	6,8	6,8	74,2	
	14,500	2	1,1	1,1	75,3	
	15,000	12	6,3	6,3	81,6	
	15,400	3	1,6	1,6	83,2	
	16,000	6	3,2	3,2	86,3	
	16,700	3	1,6	1,6	87,9	
	17,000	2	1,1	1,1	88,9	
	17,200	3	1,6	1,6	90,5	
	17,600	3	1,6	1,6	92,1	
	17,700	3	1,6	1,6	93,7	
	18,800	3	1,6	1,6	95,3	
	20,000	3	1,6	1,6	96,8	
	21,000	3	1,6	1,6	98,4	
	22,700	3	1,6	1,6	100,0	
	Total	190	100,0	100,0		


MDPost 90	
	Frequency	Percent	Valid Percent	Cumulative Percent	
Valid	,000	25	13,2	13,2	13,2	
	1,000	25	13,2	13,2	26,3	
	1,220	4	2,1	2,1	28,4	
	2,000	4	2,1	2,1	30,5	
	3,000	29	15,3	15,3	45,8	
	4,000	12	6,3	6,3	52,1	
	5,000	7	3,7	3,7	55,8	
	5,500	6	3,2	3,2	58,9	
	5,800	2	1,1	1,1	60,0	
	6,000	30	15,8	15,8	75,8	
	7,000	11	5,8	5,8	81,6	
	7,600	4	2,1	2,1	83,7	
	8,000	15	7,9	7,9	91,6	
	8,300	4	2,1	2,1	93,7	
	8,700	5	2,6	2,6	96,3	
	9,000	2	1,1	1,1	97,4	
	11,000	2	1,1	1,1	98,4	
	12,000	1	,5	,5	98,9	
	15,000	2	1,1	1,1	100,0	
	Total	190	100,0	100,0		


Shift pre	
	Frequency	Percent	Valid Percent	Cumulative Percent	
Valid	2,000	3	1,6	1,6	1,6	
	4,000	11	5,8	5,8	7,4	
	4,500	3	1,6	1,6	8,9	
	5,000	14	7,4	7,4	16,3	
	6,000	14	7,4	7,4	23,7	
	6,500	2	1,1	1,1	24,7	
	6,600	2	1,1	1,1	25,8	
	6,700	3	1,6	1,6	27,4	
	7,000	19	10,0	10,0	37,4	
	7,400	3	1,6	1,6	38,9	
	7,700	3	1,6	1,6	40,5	
	8,000	19	10,0	10,0	50,5	
	8,500	3	1,6	1,6	52,1	
	8,600	3	1,6	1,6	53,7	
	9,000	13	6,8	6,8	60,5	
	9,300	3	1,6	1,6	62,1	
	9,600	3	1,6	1,6	63,7	
	10,000	18	9,5	9,5	73,2	
	11,000	5	2,6	2,6	75,8	
	11,900	3	1,6	1,6	77,4	
	12,000	14	7,4	7,4	84,7	
	13,000	5	2,6	2,6	87,4	
	13,800	3	1,6	1,6	88,9	
	14,000	3	1,6	1,6	90,5	
	15,000	10	5,3	5,3	95,8	
	16,500	2	1,1	1,1	96,8	
	18,000	3	1,6	1,6	98,4	
	20,100	3	1,6	1,6	100,0	
	Total	190	100,0	100,0		


Shift post 30	
	Frequency	Percent	Valid Percent	Cumulative Percent	
Valid	,000	33	17,4	17,4	17,4	
	1,000	20	10,5	10,5	27,9	
	2,000	20	10,5	10,5	38,4	
	2,400	2	1,1	1,1	39,5	
	2,500	3	1,6	1,6	41,1	
	2,900	2	1,1	1,1	42,1	
	3,000	33	17,4	17,4	59,5	
	3,400	3	1,6	1,6	61,1	
	4,000	10	5,3	5,3	66,3	
	4,200	3	1,6	1,6	67,9	
	4,500	3	1,6	1,6	69,5	
	5,000	9	4,7	4,7	74,2	
	5,300	3	1,6	1,6	75,8	
	5,400	6	3,2	3,2	78,9	
	6,000	9	4,7	4,7	83,7	
	6,200	2	1,1	1,1	84,7	
	6,300	3	1,6	1,6	86,3	
	6,600	6	3,2	3,2	89,5	
	7,000	9	4,7	4,7	94,2	
	8,400	3	1,6	1,6	95,8	
	8,500	3	1,6	1,6	97,4	
	8,700	3	1,6	1,6	98,9	
	9,000	2	1,1	1,1	100,0	
	Total	190	100,0	100,0		


Shift post 90	
	Frequency	Percent	Valid Percent	Cumulative Percent	
Valid	0	27	14,2	14,2	14,2	
	1	69	36,3	36,3	50,5	
	2	11	5,8	5,8	56,3	
	3	66	34,7	34,7	91,1	
	4	17	8,9	8,9	100,0	
	Total	190	100,0	100,0		


KPS PreOp	
	Frequency	Percent	Valid Percent	Cumulative Percent	
Valid	10	5	2,6	2,6	2,6	
	40	36	18,9	18,9	21,6	
	50	6	3,2	3,2	24,7	
	60	100	52,6	52,6	77,4	
	70	21	11,1	11,1	88,4	
	80	19	10,0	10,0	98,4	
	90	3	1,6	1,6	100,0	
	Total	190	100,0	100,0		


KPS PostOp	
	Frequency	Percent	Valid Percent	Cumulative Percent	
Valid	50	3	1,6	1,6	1,6	
	60	5	2,6	2,6	4,2	
	70	8	4,2	4,2	8,4	
	80	43	22,6	22,6	31,1	
	90	109	57,4	57,4	88,4	
	100	22	11,6	11,6	100,0	
	Total	190	100,0	100,0		


Side	
	Frequency	Percent	Valid Percent	Cumulative Percent	
Valid	left	89	46,8	46,8	46,8	
	righ	101	53,2	53,2	100,0	
	Total	190	100,0	100,0		


Bar Chart
