# Supplementary material for: Cortical atrophy in chronic subdural hematoma from ultra-structures to physical properties
Source: Sci Rep. 2023 Feb 28;13:3400. doi: 10.1038/s41598-023-30135-8 (PMC9975247; doi:10.1038/s41598-023-30135-8)
Supplement: Supplementary file 5 — Supplementary Information 5. [file 41598_2023_30135_MOESM5_ESM.doc]

GET DATA
  /TYPE=XLSX
  /FILE='C:\Users\Placido\Desktop\articolo atrofia e sottodurale cronico\controlli\controlli.xlsx'
  /SHEET=name 'controlli '
  /CELLRANGE=FULL
  /READNAMES=ON
  /DATATYPEMIN PERCENTAGE=95.0
  /HIDDEN IGNORE=YES.
EXECUTE.
DATASET NAME Dataset1 WINDOW=FRONT.
CORRELATIONS
  /VARIABLES=Age RCAindex
  /PRINT=TWOTAIL NOSIG
  /MISSING=PAIRWISE.


Correlazioni


Note	
Output creato	01-JUL-2021 19:49:30	
Commenti		
Input	Dataset attivo	Dataset1	
	Filtro	<nessuno>	
	Peso	<nessuno>	
	File suddiviso	<nessuno>	
	N di righe nel file di dati di lavoro	190	
Gestione valori mancanti	Definizione di mancante	I valori mancanti definiti dall'utente vengono trattati come mancanti.	
	Casi utilizzati	Le statistiche per ciascuna coppia di variabili sono basate su tutti i casi con dei dati validi per tale coppia.	
Sintassi	CORRELATIONS
  /VARIABLES=Age RCAindex
  /PRINT=TWOTAIL NOSIG
  /MISSING=PAIRWISE.	
Risorse	Tempo processore	00:00:00,00	
	Tempo trascorso	00:00:00,01	


[Dataset1] 


Correlazioni	
	Age	RCA index	
Age	Correlazione di Pearson	1	,850**	
	Sign. (a due code)		,000	
	N	190	190	
RCA index	Correlazione di Pearson	,850**	1	
	Sign. (a due code)	,000		
	N	190	190	

**. La correlazione è significativa a livello 0,01 (a due code).	

* Builder di grafico.
GGRAPH
  /GRAPHDATASET NAME="graphdataset" VARIABLES=RCAindex Age MISSING=LISTWISE REPORTMISSING=NO
  /GRAPHSPEC SOURCE=INLINE
  /FITLINE TOTAL=YES.
BEGIN GPL
  SOURCE: s=userSource(id("graphdataset"))
  DATA: RCAindex=col(source(s), name("RCAindex"))
  DATA: Age=col(source(s), name("Age"))
  GUIDE: axis(dim(1), label("RCA index"))
  GUIDE: axis(dim(2), label("Age"))
  GUIDE: text.title(label("Dispersione semplice con curva di adattamento di Age per RCA index"))
  ELEMENT: point(position(RCAindex*Age))
END GPL.


GGraph


Note	
Output creato	01-JUL-2021 19:50:57	
Commenti		
Input	Dataset attivo	Dataset1	
	Filtro	<nessuno>	
	Peso	<nessuno>	
	File suddiviso	<nessuno>	
	N di righe nel file di dati di lavoro	190	
Sintassi	GGRAPH
  /GRAPHDATASET NAME="graphdataset" VARIABLES=RCAindex Age MISSING=LISTWISE REPORTMISSING=NO
  /GRAPHSPEC SOURCE=INLINE
  /FITLINE TOTAL=YES.
BEGIN GPL
  SOURCE: s=userSource(id("graphdataset"))
  DATA: RCAindex=col(source(s), name("RCAindex"))
  DATA: Age=col(source(s), name("Age"))
  GUIDE: axis(dim(1), label("RCA index"))
  GUIDE: axis(dim(2), label("Age"))
  GUIDE: text.title(label("Dispersione semplice con curva di adattamento di Age per RCA index"))
  ELEMENT: point(position(RCAindex*Age))
END GPL.	
Risorse	Tempo processore	00:00:01,20	
	Tempo trascorso	00:00:00,46	

	ù±ËAnn.Ízµä2ÿüç?éòDÿYYÍEJJ»¢qUWWÛÛÛÓ·¶¶¶555üdé£µµ5EÐb<ÐUUÕH³nÝ»wïÒR¡¨¨¨¦¦&A²7nÜð÷÷g¬[·®ÎæHNÝ£/²Px÷ÝwW®ÈËËþýï&h"'.]rpp`uL´ÍÍÍCÚ/ñFUi6!yÖIî)w>?6Ï?ÿ<Í©ªªb_yåÙ³g³­P¶¹Ø °°ÌPXèÐHY(%¸H­ø(â[þ¤ôÌì÷ðõ3A&áýñÇiúõôô¨««Ó4ÿ÷EPæiGhiIIsªËðº$H±_þÍ_àöíÛt°H¥ÈÏDüú&r~ö»×þóÉSuuuYè¦i%ÌLró£;ÍÔ××éÒ¥;ýÍå/Fþ·Ü®¡¡a ¯ø+Ò_i¶Â¦ÿö·¿ñÓÖÖÜ)éÁ_ ::zÐüðÉÉ Äþâ³TÝÆ¤	Ú@·ÏÏ?ÿ>ôû%ÂH¢*å&¤1?î|èíí¥"m=ÿDït+RTT¤ùÌ[ñõõÌ+H¶º$.à@+j~"[þ¤ôÌì÷ðIfFüLyx·ß~uÑ4+0;qâÄ@§¹¹9?))Ou^Ä¯V·K¾ÅæÒG£øEX<â?~âGDd¯_~ùeú¸ûvNJJ¢é¬¬,Ü(a~`òO×ÉeØõâÛo¿¥iVC÷`þbQQQ?üð«¿à®4>^ºt¦¯^½Ê¿H±322è^ÎÝÞ´iM/^¼øÐ¤ü%IÝèL)³LºS>"9tèºIÂ¾bWvúH¯ùÙÚ@·ÏÈÈH¶§ÝÝÝ,irÅG$DUÊMHc~üó!>>æüö·¿¥iz§éuëÖ±uuué#nnn(3,°ú H£&"'(²¢xmï +JsRzftø?F^l¾ºråÊÏ5¡4½páÂ~¥÷7lH§º¯KÉJBM¶;ýE¦U<â?¥øÙë®®.SSSºî=zo±æ¦ùquüeèB@Óti ÝüñGARß÷¿àPYY¤	ÉÇù+òÿº6­­­Í_ü>ª««óä(èâÈþÁ>"9tèËÿÈ*bX6ï6Ðímºß*!)÷K$DUÊMHc~üónQ/½Ó4¿ÂÌTîÐNNNe¤¾ºÿ>W<#¥¸x"+úøÒT>AfÄÏ2Ïê¹9ÆÆÆ4«²d;Âý:X¤ÙMÉSK&×¥AÍïå_æ;¢4æ7hÄ&Jñ#"ºÚÚZ®Á2êya~`ª]th¦¦¦¦ä2L¸ë÷¸U¿·s>:áÅ*ï´Éë@Â:Ðu³ßüð4'"4PÅoí¦"å~¤0¨J¹	iÿ| i:ååå»ºº('ªªªÜ·W¯^ÔHJiâ)¸xÒ¯(ø8ì¥<·ñ3aäá---í×h¾Â2¼.õ,-ðÞï1rwwïèè¨3«1A Tú­HèX=5ý÷À-æ¦ùÔÂñÅ_Ð¿[V¡Àý[eqOñÓßMþ_Iö´»»[zEÙf÷HAD¿Â*9G$?RæDú=ô&=PÐÄËü¸âÉÒA÷K$DUÊMî©ì¸(°çÖ­[Ç=Å°¶¶¦9¿ùÍo.^¼H7]ñÌp?,ÎªHââ	¬(~nE)Ïí!éø0òðz÷+=4_2?)Ou_úýKjYùtNNEÖ&®nÐ ÏíÄxèX=/c 'ÌLó£kÁ©S§ØëÕEò9?öRcc£äã.¤ù?þÈ®zÏ?ÿ<ûiSJJÝïYC3û.anMÇÅÅ±ç:ú`HJJæÊ()s"ýj~Mü9¿ôôtÚ4»åi¿DRIT¥Ü»->|¾¢[R>ýôSîyöÚÚZA¹TCC%ÅHf=çÄzôèÛ©AÕD$qñEVdgÔ£>$?¬(ýI5¤ßHÞÄÏßcöìÙ§ý@èg2°¯Kâ§=Ïl&»:HÇ´°°.­´+W®°â4ÖXX ÏüÄÈ^Óf×úå²6=üRLóÇü$áZ°öAáÏ_ßØîÓÜc"mÍÞï½.anM755	X¡ë÷høQ$?RæDú=Ôüôm¹r)÷K$DUÊM°¢;þ.KãatdeNü8Îêêêü8È¥T	ZzoQ$qñEVdm8¸2uÁG¥?©ô[lÂeFüLaxóóó^æÐüÜÜÖÎß:ÙÒ0~Â2¼.zÚ²jÎXTUUoÝº%eg~âGDd¯Y^V¬Î,yëÖ­¸QÂüÀd6?º&úúú<yr ýÜ¾;ûKJ·%º(tuuñûôÓOèBãäätõêU~:ÕÕÕ4=Î&rt+ÜtïþàòÇÔQÊ9~5¿&r¡gu±MgffJg")$ªÒlî$Ð,ÖqP£!YæÔÖÖF9¤tÈQèè¬þ·p°®ïèvHY²Ã9ñÄEYñ·¿ý-ë#ú>¬(ýI5¤ß 3âgÂÃË¥¿ùìdà0«ªªbMMYãÛmiNu^=í¿ûî;&ë"ñÖ[oÑE²Í6±iÓ&®êYÊ ÃüÄÈ@M>ÊÌ=¤AgÈ#q¯ù í¥ hVRÅD­§§=fmm0?AÐl°çüÄÀOxj°*!ÄA@&<zô(==ÝÐÐýLhb×®]øó0?ó0?cÍãÇ`~2¦°°ðü£lÓlkkûòË/qº b£Çÿ÷ÿéOB±ÑãþýûO±Ã¶÷î½VôÉÇL ÿý÷7oÞÄ`~ãW_õ÷¿ÿ½ÌÿÜð¾ûî;ÿcAÄüã	===µksöç|õÕW+bÿûßÿó?ÿgùÁü"AÄ`~RÑÜÜ ÷TÅ©8,,ÌÀü`~Ç b0?©xðàÁéwOÜýæîÌÀü`~Ç b0¿Aèíí½víZö¾lº÷ôôLÜÁüÌæ1x"óãÞ½¥¯¶¶¶NôÁüÌæ1x"óëîîîj2_Í¼~ýzooï$ØØßovþ½^ú/íÏÞÿõ×_ÓÌ~úéî7wxð`IÉub|þvïoá]/í*8»éw§Ø~Ño[¾­ð5Z¸° VìwùÁü`~ðMó»wïÞÁ¼§ß=ýàÁI±13?nd(_6M|ùþçÞÿýñãyTTTÔ××ÓÄgvêÔ©~¡è[È?ÿ_ÿõ_4ñå_Ê?ÔïóùÁüà1ïæ÷øñãsçÎ½VÔÜÜ<É"6öæ÷»ÉÙÃ¦ûÛßCï|òIîÜ]/íbíi>|øFÙô±¸¨øÿüçÏåsthÎ¾¬¬³UZ®Òú]^²öfýôÓO4ñ¯ýË6WJVrþË»_tùÁü`~ð;óûü³£z÷i÷:UvwwWWW?zôHJó»ví« &£9'Oü¦ñøë_ÿÊÜëð¡Ã4Mííí¯¾ò*[aê&¹|¿ÙdmKrãïÜûö`&ÍùàÄçÌæØø2¿²#eô¢d'kÄÆ²Ìï_ÿúWEEÅ­·ØL2¡÷ßÿÃª¥4?&pÜÝ»vs®ÆìÇüäON¿Pr-Éå¥=ÀÇ¥ ø¶­­íõ×3ÿ·ëi7ÉkEæÌæØ82¿ÞÞ^ºÆfíÍª««EOÅü'Oäåæ¢º¤¤ù	æ	úÓ!	#­lúKÓÎkI.ÏOp 2¿YûHX¶Ò´`ÅË/úé§ü9?<øáìÙ³>`~0?< bãÈüî¿ôõÒÇOLÄ19Æ¹ù¤JäÓ#7¿·½ýñÇÓÙ/ævíííÝÝÝ|ðäZËKÃéwOñÅ4Aïoá÷ìCÒùæÑ7?~,2Àü`~0?x@ÄÆù=yòäâóÞ¹sgDlìÍï§~T(þâ·Ìï?Àí¶µµýÜ÷Dæ÷¼úÊ«×¯_ÎÞÍ:£ù¶å[ÁîPj³?GyÉ9æóùÁc"öôÍ¯··t!óÕÌêßUO©ç±Ðó0@[Ìæà1Ø6?Ö£èµ¢÷îMµÁüÁåËÌæà1ØÄ3¿ÞÞÞÚËµ¯f^¼pqj6½ùÌ bðDlJ-ziyyùýïïOÙÁüÌæ1x"6ÉÍïÉ'ç?<_ôZb*ó0?@Äà1Ød6¿7ofïË&ó¨w70?@Äà1Ø7¿~÷ôÁ¼---ÌÀü`~ðDMNóëíí½víZÖÞ¬ÚËµL0?xÌMióknn.((8vìôý÷ÂüùÁü"AÄ&ùuww_¼p1÷@îW_àÀüÌæAÄà1Ø¤5¿;wîó~÷ôÃùÁüà1<æ×ÑÑAÂ³?§¹¹1ùÌÁc±ÉIkkëÉ'3_Í<ÿáù'O  0?óùÁc1x"69innÎ=0ïà?þñDæ`~0?xÌMNXKÌW3?®ýùóÇÀüà1Ø¤¥é/M¹rÏ=ûàÁ!Û`~æóÇ bðDlÂ@ªGÂw(ÿÉóùóÇÀüà1Ød£··÷úõë¯f^¼p?ü.ÌæÌóÇ b¶¶¶â£o½ÿýÁW0?0?xÌMzzzj/×fïË¾Q£ßáwa~0?`~ð<ÕÊ?tªâTGGÈ20?0?xÌM`<yrþÃóE¯Ýýæî vóùóÇÀüà1Ø¤··÷Úµk¯fÖ^®å·äùÁü`æ×ÖÖ¦¯¯Ï¤K®®îôéÓmmm/]ºÔïÌM)è:YôZQéë¥÷îÝrÌñh~W¯^µ¶¶~æg,**êí·ß¦¢¢¢èèè~çÀü`~ðDlÐÓÓSóQMÖÞ,ºÐõÛæó`"ßÂùæ§©©É®nÝÝÝ¬,PrÀüJJJ>)W®xñâg5>ùäDêêê9kkk±18Ç>úè#'ó0¿þ²Å3¿éÓ§¦%çÌ¯ªªê¿eÉ(ý½þofÄ®^½8 bñí·ßëX^nÞ_~9¼îÜ¹síÚ59Òóç?ÿy4"ó0¿AÌoÚ´iÜ´¼¼|¿sPÛÚ^Ô]"bÏÿøyÖÞ¬óïîîv"¨íEm/Ãü´µµÙÅÞiºß90?<ttt8~¢ôõÒÖÖÖ&óù01ÌoõêÕo½õMÐTTT¿s`~0?x"6ù¸víZÖÞ,);mùÁü$æwõêÕÙ³gO6MWW=+9æóÇ bûßß?úÆÑÇOÉóù0yÌo$Àü`~ðDlâòøñãêßUÌ;xçÎÙ¦óùóÇÀüà1Ø8âó?~ùjæÙ³g<y"óÄa~0?`~ð<°òI?&ÌæÌæóÇ bºººÌW3/^¸8E0?0?xÌÈÆJ_/=qüÄýïïÁ¶`~0?`~ð<<yòäüçåºûÍÝ1³LÌ<æAÄÆ¯¿þ:_ö¹sç?~<fùÁüùÁc`~ðDlLyðàÁéwOÌ;ØÒÒ2ÆùÁüùÁc`~ðDlèííecrÔ|T3áwa~0?`~0?<×Ü»w¯  àØ±cmmmO+0?0?xÌ.ÝÝÝ5ÕdíÍºyóæÓÍ	ÌæÌóÇ b£HssóÁ¼§ß=ýàÁ§Ì<æR]]½sçÎôôôÊÊJDlØtttª8µ7ëë¯¿'YùÁüùÁc`~ðÓÕÕµdÉ==½ÐÐàÅÓLDlHôööÒ%(óÕÌóÕ19`~0?`~0?Ìoø¼òÊ+fffÛ·mÿÍÎßÐ&,,,2221é¹ÿýý¢×Êf·¼Áü`~Àüà10¿)á1íííÒ,æêêºæù5LûØ+vm¬½=ÌOº»»/^¸½/ûFýÞÞÞq1Ì<æ7=¦§§'##CUUU[[ÞSSSiÈò^ØÄ7?úheeóCùÎ=;®ªwa~0?`~ðß2¿ØØX-I[ÈáèÝÜÜü¥^Y>..ÎÇÛo~Þ^Þ«V­ùððáÃ>ø ÷@îW_5ÎÏ1Ì<æ7i=¦©©ICC#=-Ó¸ä=z4Ð*­­­þþþ´½ÕÔÔ(_¿ôöö~þÇÏ³öfÕ^®/LùÁüùÁü`~`t=¦ªªÊÝÍ_G/++«úúzµZZZ/^LÂ§ªªØØØ(Í¶È&+++óóóé½³³s#FÂZQQQXXXSS3fÖÑÑQv¤ìèGï¢c0?0?xÌoÒ_]]yÀüfÏ-ó2</ccc?1Xmm­²²²KeÃÃÃCÊæ,Ã¦···örmö¾ìk×®Ï0?0?xÌoÊ_WW¦¦æÊ¨ö-_¾ÜÔÔTæ¥bÞÞÞÜVÂÃÃµ´´Dêe±.öñ÷ÑÙÉ9,,lTå©ôõÒS§:::&Ü9óùóÇÀü&­ùýÜWì§¦¦æááA6æææFÓ2/kmmÕÖÖÎØÁ/Y;wnmmíD~ì...üM§mMSRRÞ§>|xþÃóE¯Ýýæî=Ç`~0?`~ðßd6?ffùùù			999---2I³½½Í5:::ªªªóæÍ333Ô)eÊdä·#G+((ÐAAdÄÒÒÒüýý['y/94_bJÓöì!Í¥ÚØØ9sæóæóù§i~2çÑ£G¤zi[Ó2vd<ûì³¬ãnäÛ·opC;wîÔÕÕ]¿n=ëPZ___ ±ââbCCC~ã¸d~2Üß¶¶¶òòò¢×îÝ»'ø*&&´õMÛ­ªªùÁüùÁü`~`ò_VVÖ<ûyº]R=&@É/&[[[p+­­­©)©ü>¥ø²yyy9:82íÓÒÔzóÍ7eµ³µk÷¼¼§æ£îînÁWuuu´-n¤;z=·ú9ÌÌæ&ùEFFFGGóÍ/cG¼¼¼²²2)$11qäÙÕÔÔ¸ººªq-,,øÏ)²uvv.Y²DIII»#GH¦ÖÞÞN	VWWÓi)eîÝ»Wúzéã'ÚÚÚú] ¬¬ÌÏÏOCMMÍ1ëÔF¶æ7ÁüùÁü"6ùÍ/&&fEäA£555vÐeÕp¸¶¶ÖÉÉIàUÆFÆüJd~Äh»=ÛW\L>êìäìèè¨  pøðañM?~üøÜ¹sÍÍÍ"Æ;!¦A£f³9ô !ùóùDlªßñãÇMLLøCÌ÷)Û­?)+)Ç®å¶BÆ©££Ã7Ki"V]]­¥¥ÅF¸9ir£þFö¾ìó¬ÞÐÔÔ¤¢¢Âæ8|Q¸³³óx>výßPCóæóØ2?"""äoEä²1g'gýÑ¨"$ùPWW|nõsþþþªªªßµ4		XÁ/£lSjKvttª8u(ÿPkk«9,((ÐÐÐ£úxûhjjJ9äÉ¸2?éCóæóØT4¿ûJþV­ZEÒ³k×®~«Y;;;·mÛæäädgg ÒÍJqq1yÕ+hÅôôtGGGZæÄÅÅÑ·dpø£·mÚ´ÉÞÎÖÊÈÈà×·R²ùZCI[IÝ¨¿Áß´¨O]j(oÁÁÁIII#oÎ<0Ûü¤ÌÌ bS×üÄééé!Y±±±Ù·!ac»»»^¿åf¦fÏ­~l#4$TQQQ__ßÕÅuÐæµð044tvv§µÈ8ù]¶t_kè#¿@ëþ÷÷¾qôÄñãpL£­­­4Ñ¶ù"â`~0?ÁüÄ(,,´°°àw³GÖ X¬ªªTß1ÊÜ¹sItøÒïü%''ÚX[[çää°eª««555Y§ô¢	uuuÖë^ww÷Åsöç|ýõ×ã6VVqæ'"ó0?@ÄF×»ï¾;Ã=ö¿ñññvvvÅvîÜÌ_LWWwCÜAWä£££`Q+,YÂ÷K555SSS8ú4Í¼ûÍÝÜ¹gÏøðáDãÍ"2o.Dæ`~0?x"6*ÔÕÕéééÑ­WAA¡¸¸xé_Y6&ôk~¡!¡ó#¹tEù	VæßÏÕ¦·û ±&Õ;qüDÎþñN[&PGn~á'	ó0?<-ZZZ444"#"¹[»NEEÅÞ©~k7nÜ(X¬ßÚ^;[;Am¯ä|ó¯íð§?ý)koVÍG5O<a¬í(#4?ó0?<ÒÓÓ|~Õ!ðs«377Ð;5òñññ2iáÁÒ×K;6[rqZxùÁüà1ØØÁzV!'''¥9sÜqçÎ555ãj¿HÈô´µµÝÜÜD~ôôô$Ù¬¯¯§SRRìíìY?&"¢ÃïÕ´ÏÚÚVÙ±c@û<yrþÃóÙû²oÔßèííX§E#55UhÀü`~Àüà10¿	éàY·	Ò8«««­LµaÃY6r(KÎNÎááá>Þ>***Çí&¤iÝÜÜ³?çÜ¹s?ÆÉóùóÇÀü2t®ªªªíqcàïÜ¹sÐ×®]kkkË=F+êêê9rdì¾¾>ßh7ÄmP¡ Û!.ÄÍïÁ§ß=0ïà;wpÁü`~Àüà10?©èììíÖgÎQSS7o£££JJJ4Ew:::[¶:à%ÆCÐHC·nÝÊÏ»GIII¿7Ðüa_ooïµk×XK¡Éó0?óùÁü¦hÄª««MMMI°TUU]]][ZZFo[=zÿý÷óòòÈ¤ßê^111aaaO=ndcZZfffÊÊÊmçÊ+I©9ñ¥ kjjÒü   éeë×üÚÚÚ^+:vìMàgóùóùÁü¤N!r)R¬ÀÀ@É£2d¨cxøúúçñÍÏÑÁq×®]O7n$pK.eVJ9$ÛÛ´%la ("mk³³ ÎÛ·m÷ñö!A2ÎõôôÔ|Tµ7ëó?Bn`~0?`~0?ßPpssãúØc/;û²²²ñc~JJJdT$LäRó=çëëë···?Ý¸Ã	:ästtçÆc/##£Ä±÷îª8õàÁüú`~0?`~0?ßÐPVRÔ¥oÞ¼yü?___ò?55µ+V°Ë`mm½éMî	ûôÏÏÏï7ÎQQQ%%%ä·oß4b><wîÜí´æóæóù7'òÄÏÏo÷îÝãÊüÆ!>>>î	,^²jÕ*UUÕ´­i²Àòòò9sæâìãí£  @éx-ðRTTÜ¿¿È]ùwÞÉ|5óüç'Ä0?0?ÌojcmÃGmIÚBîRWWó§°°ÐØØ8=-ëkFOO¯¢¢bÉ%înîÞ­y~ÍÌ3¿þúk%%%.Î^Ø4úôåËsa×ÐÐèw,»ûßß­ðµ¼Ü<²üÜ`~0?`~0?ßèêêZ¹r¥§§Âo¾9ª[æG¤¤¤¨©©ýRhçåEÞÆtvv:::úxû8;9+++WWWIÏ5733SWW_0£¼¼<M³ìFùé÷öö²Âõë×ñ[ùÁüùÁü`~²Ô¤°°°¤¤Dü³aóèÑ£´´4ò!%%%ûââb2¤ØØXÖÌüùóëëëÇrËÊÊ,,,HsÍÍÍóòòNCCCIüRÒ_ÚÞÖÔ8p ¢¢¢µµæTUU±ÀèèèÿÐÐÐM/l²³µXÁLÐÃÃ#0 l<ríÚµ>ùÎÉ[W>rä)ÌæÌæó¿9½=ëyÙÒedvvvlNÆ°°02Â1ÒÒRuuuöÞ¸&&&)))£ºÅ¦¦&eee®juì¢¨¨ÏÍg?O^^>|Q8¾Ýº5<<üÇüy¼îÜ¹SWW5d]«¯¯_PPóùóùÁü¦:f¦fü­sæÌáÏ!ùsssÌtuuerÈÑ+5%$Lú¥×&ùÅdÜÒ%¿tH¯ÐÐ3göñÌ;·¼¼||_kk«Ëí¦6)((jO0?0?Ìo°sçNÓxxx,?ìL¶Û%©­­­®®æJÒØØheiµÛö5Ï¯Y¹) ««kUUå²®®¤m±<TVVª««ÉM>]UUÉÙÙÙÎÎnöìÙ4>º!NNNæWSSãîî.È°ECCÃÔ4¿ööv	lã¡Ë!0?xÌïi²÷nA§Çnnn~~~ü9©)©jjj2Ü(#ØÉÉ¶EN¹wï^6¿©©I[[ôËØÈØÞÎ^QQÑaIÝ³e²]Rò9s²[R:999++«Y³fÉËË[ZZêëëÓLVC:ßs~IIIgg'Ù!"E`Û¶müÒñi~¤³óæÍÁ(=':ÎÍ¯  Î(g:Ù>óæóùM]è,%	K~1S¡3gòç¸¸¸DEEÉpt']Ë§r±3§Mµ"N_É¤¨¦½½]SS3lá/cÍ%lL.G"¿$ËäòWPPX»f­Âò$rÒ×KOUZ¿~=-O¾XSS3>Íl ¿GÃ+tttzzz¦ù?~ëÖ;qs"ÅaTGÁùóÇÀüÆ;ÙÙÙ$~~~ä=¤¦¦¦Û·oWVV¦9aaafffNNN2-1110 _E^âííM_åçç»¹¹ñ¿Ú·nÕ2Ùnee¥K6À?ÀÙÙ&ÈóXÓîefjF3KKKÏ=(ÿÐÝoîþÜ×«««	2OÚÇM<>[xÔÖÖ^xxÐ1¥Òô7Ð'æÂo£Í?Ù`~Àü`~0¿©iÁ¾ûÈÉrssI~î+Û½wrrryyyWW·Eò$`#qs"IE#STSÊÉÉÉd»þ~þ,MÒ8ÚPÚÖ4R"Áæ¼¼¼ÒÓÒ³öf;wîñãÇÜê===)))ééé¬´Ozó«®®&177&¡cÚÚÚJ&Ãú¯æGÿd£³p'ÌÌæÆ¢ÌÉÇÇG0H.URRÂâøé0Éd»¤_,Ùà;PTTð»6voæ^~»F¬¸¸XKK+by9½ëèèV&½ù-[¶Lð_;Ù`~Àü`~0?0æGR¥¡¡ÉÕçjjj²Kgg'ÑÂÐ¬OR%ý'OÊd»===¶¶¶^¼(ñ´­iJJJAAþ~þÚÚÚ¬Â7##cÇöCêýD<bííí´!¾NÑqU7ÍÊç×pZOe~eùóùÁü&$]]]W®ï½÷XSò°úúú¡:¹­%þ]]±±ñ¹¥¥¥ÜW666ô­µ5Ò+¯¼"Ã¤±³³#Õ£Ägö¡ªªª¬¬L~¿!>jE¥¥%Í¡ùÕÔÔ8;9*]]dÕZæ7(UUUjjj¦¦¦sçÎ¥Ó§OO¸ÁüÌæ1ÙSVVFÒcnn®;GWGG'"">ZYYihhxzzJÓÂiåÊêêêäU***K.Q(?ÚùÜ_Fûí·ùß¶´´>RÏÃ´Ë©)©;3v&lL`cPþek~ósttùåéÌí>dûÄ*ÌÌæ7Q)//5k7x9YÚÖ4Ö³»»»««ë  ::8²RèÝÞÎô±ß%é¬¬¤Ì¯ð¥­½]¸p´oÇ¶E,033R"Öö×JÖöJÿ!ÌÀüÌæ1CÆµ·%á#YaÚÇá¡££#þüÉÜìÙ³ùc¾QývÅ×o#1Ûßª8 wëÖ­ä¸111$d¡!¡äduuu24¿ûZx×ò[xäååMåæóæù=eÈxø]èIVææêvæÌªªª<Ü=kYZZ¬èÑ£GÐÕÕ566åLq,ûÚ¸yó&9_íåÚîîîûz?¾¹¹yddä0:½¾WÚAÿ'Oæää,]º6WVV6NzWùÁüùÁü`~Sò6j«MQQÖ½ÈÅýÉÈÈ_æ·Ûvuuu~_WW¥ÕÊ¨+"Wÿ±Uè#YÑhïæýïïãèã':::dæZC···Ó.Ï³Ç"@ú»páÂ©&0?Ãüjkk­­­§OnkkõêUC/ºu±9.]ùÁü&.ÅÅÅt2ó1i316!?ã4.8(ØÆÆfÐ§ãX)l-OOÏÐÐPþöì±³³ã 	Ú(-üb2MjÇÝÝÝ/ÚuóæMÙ¦<$ó[³f¿äïÈ#0?ó0¿q¦¦æ·ß~Kô®¯¯OQQQ¬AbQQQtt´¤ù½ÿþû÷dÊW_õÉ'ÜØ(¨¬¬ììäÌÆí¥wmmmWWWRë×¯ÿå/¹sçH7nÜ°··§]\ÈäèQCC÷-MnÛ J~Y´ÝÔÔÔQÚ¯æææKÿqé@ÎãÇ766J¿"·¿ü½äÖ­[W®2M---þ,!!!²ÚÓÛ·oÿ3mHý]ùýï/ódaæ7¦¦¦ôÇýý¢iæ½½½¬8¹ Àü=zC¦ÔÕÕÕÔÔÜØèðÑGåäädff~üñÇôñôéÓôñ­·Þ:uêIªªªºº:é`aaá@)|ðÁæææ$$sÜqqqô´r¥WLL	bUUÕhìÝ,WE¯J~1v÷îÝR®HÁöwÆÓ§OWSS£Ý!3ù9FIñ+ÄY¼¼¼F¸§.]òóó£Ä)ÈÒïéSáÓO?ýÿøüÜzÄ`æ7þ9Ý·yæzgtKà¾åO£¶µ½ÉºËöövò   ¦,d****ükÅf>[[OÏ_õlçà¸k×®QÚQ+·¦ne¶lÛ ¥©õæoº·vvvÆFÆlxÊ¿¡¡áK/½4ÂÚ^__ß°°0ÙF ««ËÌÌlÁìÑLé÷µ½¨íæ'ËíÛ·º»»ÓÄ´iÓ¸oåååa~0¿Ii~ä%tÂóeÊÉÉIr]É%#GÐ"&Oô¢	EEE¯^ä(i[ÓæÎ×××¦èa¸EÎþr ;vòÛ,kkkº.ÛR=eee~3ä$;yù544P"aÃ¶oÛ.«ÛXÛðÃ.åÂü`~ÀüÄ,á£k+ëÞ%¯³0?ßÄ¢±±1??Ýºu©©©o¾ùfzzúÎ;?øàûAw-),Z´H°$)N||¼­Nï+V¬àZþÖ××¯]»ÖÕÕ588¸¢¢bØA?ÆêßUöegg²­®®ÎÖíÅÈ¶¶¶Õ­¬¬$»êHÇ$¾¾¾äðEá¬RØGC©a~0?¦ù¹»»ßºu]¸]\hbõêÕo½õMÐTTÌæ7q©¬¬TUUõñö	'¿÷öò&sRêfò­bKÒÉDâââK®]§¨¨(x²ÍÚÚº¶¶¿â3gh³fÍ	QUQF_'ÍÍÍgÏüø1é£»Û·m³µ±íììIíÅç×Hvj8öì¦¦¦ÌÙ·o©¤`Oéß)¹/ÌæÌoøÜ½®§ôNÓ4çêÕ«t'6m®®îgóùMPZ[[Éº¸þüè¦¡¡AÆ¶5<ì-ùÅd®#yóæ%&&J¦S]]Mk	ÔÖÖä×ÿÒ¯_gJ[§ôõôôÈW8q¡tN<)ý.<xðàô»§æüê«¯¸C¯¬¬LÇmÚÕÕÕÌÌÌÝÍýúõ"I±½HJL¢Õ-]Æ­dkk+é£OÝüX27òÛS###Ú¡Góùó>0?ßDA²xU²fK/!WSPP ðôð$[²dÉ@û<xPEEÅÛËÛ××wÎ9´ä_ÿúWccccoo¯££#¨0¥­wrcÇ±mtåÊRæü&koVÍG5ìéÚÚÚ3fXZZzyyÑ.èëë§¦¤T`)¹NNrrr,077'klll©ßÏ¦ô§ÂËßS??¿Ý»wÃü`~Àü`~0?ð+ýýýæGÆÃJÉÉHzÈÃÂÂhæ=ÄëaÊËË)M/¶ä£G*++óòò¢¢¢LMMI"­­­YýF(MeAo/´Q~Óïììv-ÙªªjPPÐáÃ¾q´­­­ß7lØààààÍ)J>¤H"J9QRRrrrª©©a½k×.Ê9å_²mÇø1?BIQiqøbþoÞ¼æóæóù_A6ölþÓxÉ/&ËËË³­îîî,ká>bbbæÎË¦IÝÈKTTT¼¼¼È·HYøæçêâ!©¤«««­í[^Ü±sÇö>>>"X²ÝëÊ¨"y£(WUUURî×81?77·åü=µ·³/++ùÁüùÁü`~àWtuuçÙØØ°á%HHËÌ_@.4sæÌ-I[è+GG2¤ám¢®®ÒäJÝè°1aÚ´iQQQ4=gÎ99¹%0û¶HMM­µµU2Bþ#$="»Æïë.vm¬¶¶¶ 3BÉ¼11XæGWVÖ¶öÅÇÛv| rJÌÌoÝø«ªªúk èZ[[[]]ÝÒÒ2	¢Dûnanakk«  À®Ù¢¢¢æÇÅÅ:tï@Iö±¢¥¥Ås#­¤Éõ!/'OÚ÷Æoôk-;wîÔ,*))áááÚò¹ãÇK7MMMñ&ÀãÍü~îÚ7BñññéWa~0?`~0¿©k~tæÌckcëááA·Ìõë×KÓÈéÓ§I#ÜÜÜÈ^~ùå¥¦¦&®ÔTVðfccSYYÉq¬ð)//_°`À®TTTøi»zzzdX;;;+W®ð¹víÚÿûÍÿÛ°áW#ÿRéØÊÿ@ýÛIærBXÊ³qb~äy®.®úúú=:ÖÖÖ0?0?Ìïß466d¬ZÉnöéiéfff¿ùÍoÄduj±kc¹çáÈ92ÑEÆ°téRNhGÞÉ0_.=,_¾|ÆüÇû<=<ÕÕÕÉäØÇèèhÒ¯Zýþ÷÷¾qôPþ¡?ü<ë8æÇÝÜ%ÓaÞYÅüÈkéìï9XÓÏ×ÏÈÈµ½0?`~0?ßÿïáá!pÂÀÀ@<ÁÄÄÄÀ@þZ+"WxOô@`u9;;»»»khhô;2ïH¨¨¨ø¥²¯³hg'gsçÎ¯ØÙÙùùùòéåáîE¿£=/ï©½:myùåÉJiyZkîÜ¹¤h#¬âøñãìíì-,,¤¯ÇæW__O'è1ÛÐÐýùÁüùÁü`~ÿGrr²`À	zÉÉÉ'H¶!èy.qs¢É$Ukk+ÙðóÏ?4÷|ñÅÖÖÖlä1777~¡`gggzzº££#¿6ÖØØxàÀÍ7ÓØº]]]eeeäÓ»ví9s¦àpDGG§¦¤¾¸åERÒ®½-ý²âââiUUÕ_¤'#cå[$Oûöí£¬[·ÎßßßÜÜ|Ñ¢EÒüÇùQdÈÑssu;sæl7DGyÛ¶mNNN"GæóæóæWRRB>!èCî÷â	Üøøøð×"4ñÚÚÚéÓ§ûúø&lL]k``@È&#!033suqÝ·¾uww×ÓÓje±½½ÀªIûH%##"I¯-]¦®®ÎTDµDñôð§ZZX:88¼r/å<&&¶H¯¢¢Â=ì8ÍïöíÛ~yhZ²ÓìÒÓÓCÎgcc3£óùóù=ó#Y¡[ãÂÐì~IwzýAGkiiÑÐÐ á*gÍ%ó=>ÍOKKkñâÅüÁaiN@@âæú«Q@H´ir,-M-îy»6Y®]Ç¥¹~Ýz555Ò;z740(¸¥¥eNNÎHö½¡¡.÷ !Þ¶%Þ¢y´ðvtpd×Ð¡quuõõõa»|¿ÆQùÁüùÁüùýÜWùhcc£§§gmõKæ+¯¼"Muuu´ÖzóÍ7'SÄòò-Ád¡¡¡ÚÚÚô­££c||<ÿ+8;;»¡n=//oUôª-I[h]]]]:.Ì¹sçþîw¿355UTTdÃ¯^²dÉHö½¬¬L`lâWó£¡¢¢bee¥®®¾páÂ?þ( 22k5£óùóù=óc´´´Ô××µ¢°©©©¡¡a|¶%ó1cÀüBC8óc=óq/Á¡:Áú9ûs.]ºD79ïfbbBægldLæÇúæÎ1róóZà%¹Å	a~²=ÊeÌoEäeÌÌï)Òc´´´.¯í5k«íMMMuvúÕCÎÎÎÒ×¾^zªâTGG_bäääøEk_ÃjÕÕÕõõõÌÿU'|#¯íUQQáw3QjÇÂÂBòàaeÌ<æ7ÌïÓO?1c£ùÐ²¥ËHû455YñRgg§µÍÊ¨ÑÑÑ¶¶¶ÒÔ6>yòäüçsöçÜ¹sGòÛÚD`@às«ó÷÷§-VWWÿÜ×ÖäOII¶H[¹D0((häE°ä$¸¬···7mqÐ|§ùõôôRÀta~0?`~ðßÄ3?O¬9sæ¬Zµ?¾iAaaá²eË,YBþÔ¯Ñ2%%%l7oó;wîñãÇm±¡¡!11´/!!±obbbddäééyäÈYµf -m+888))I¼wªGæóæùMó	]]]VV+£V®^½zýúõi[Ó¤Q«)±ÉÌæÌóü³gÏ;;;~¹sçNï`~0?0?Ì#ÄÕÕuÓß-[¶lôö¥««ëÛo¿ùÁü`~Àü`~0?0¦óäÉç^Ð)LLLLXXØhìÅÍ7gÏ=úô3éÍ50?ÌÌæÆÂcî~s÷`ÞÁÔÔÔùóçóÍÏÞÞ~÷îÝ2ßyyy-I[h+^Ø¤©¡9z#ìÁü`~0?`~0?Ìïº»»O¿:÷@î;wZ[[ÝÜÜâûpvv¶±±ù8Äºuëø#|$¿üì³ÏÆ¶`~0?0?ÌoÔillÌÏÏOLL,,,­Ðê1%%%7oÎÊÊoóæÍyk/×ÿqë&''ÛÛÙÛÙÙmÜ¸qTÌÉÉIrØ7mmí~÷««««¼¼"¹÷îá	Ìæóæóù"ªªª^¼ÂÃÃuttÆÆü(úúú6Ö6aaa>Þ>'OJ.vÿûûGß8zìØ1þcÆ+æÌÃ×¾3gÎlmm,ùèÑ#GGG3S³ðEá¹¹¹0?ÌÌæ7^ 5kÖúuë9­!k133U?ÆâC®¹`Á¿ÇLÛ·AII©¥¥[ ···æ£ý¿ôÒü´BôñÇËÉÉrùÁ9s$CôK¤½=×î$qs"É_]]Ìæóæóù***<==UzzzMMM£m~dN$üççèE)//g½Vtò>|ºQÊÈÈ6m±±ñ/Î7²²ò×_-¹¡¡!kÂ½ÚèæóùóùÁüFÂÂBùYYYÕ××sË9rÄÜÜÞóòòFî1övö3úlÚ××7??¿ ÷æÍ½½½ã!P_~ùebbâ²eËöìÙÃe²² £ðððÍ7Ãü`~0?`~0?ß¸ ¶¶VOOï+É/&äq#¨nÙ²EWW7vm,«511ÇØiii=·ú9JPII¥Ì½¿<gÎÅÉÿ&V0ÝÜÜ-]Æß[Û²²2ÌæÌæó/xyy9:8¦¦¤²Îê_zé%öUSSû½hZQQ±±±qxCBIZ°1+SUUeO¦mMóóõKJLz½äõûßß¤_(+&&vgû¶í>Þ>fffCÃü`~0?`~0¿Éc~t¯¬¬ÌÏÏ§÷j.===ÕÕÕÃÚ~ÎIV´µµÕÔÔöìÙÃµ]¨ªªrwsTÈº¸¸Ð>Ïc,--ù©M>¶ºcû.ÈªqÉS9FgÎÑìÖ××W²ý/ÌæóæóðæGó-,,üýüíííuttÄ»¦*9·µµ¥8::*++×ÖÖiooXW]]­­Àüììì$×ÒcÈôõõÃíÚµë²7þùÏ^(ót(£z(]]]Ã[æóùóùwóóòòòööæ?×¯§§7Ôj¾QEÃ?~É_¿¾hjj®ZÉ­­¡¡!ÉcccòïR¶¦nMÜxùòåQÝ2Z33³°aãùÁü`~0?`~0¿	`~¤AÚÚÚr,ssó¡vä6zôCgA×¥½PSSsss#Uòôô$í¾À¯_ill$tvv]»Ûv²ÀcÇvêëë-Ì-ãêÁü`~0?`~0¿a~B«psu;sæL¿úÄÅÅvHíß¿ôkÏ¡­­­¥e#88øìÙ³"«·¶¶æçç'$$äääð»Y¶Ç477ïÙ³gÇö2/Dªª*WWWéÌæóæóýG¬³³SII)mkt/~xdNjjj>Þ>^Ø»6lñâÅ£-9	QTTX¸9Þ)·cã1×¯_ÏÚuíÚµ±ì¨ïöíÛZZZüROcóùÁüùÁüXÄÌÍÍZmß¶ÝÕÕÕ××·_#Ïã÷LAÉ?É/&ËÉÉzrÙ ÿ#5FCÔ!yÌ½÷J_/-;RÖÖÖ6öÇ.88ØÑÁ"~`~0?0?ßx§¾¾¾²²ÞGõ^>PÄh£«V­"y²²²RWW_¸pá@m 444ã¦§§5'~MMMuuõ ­4¸nÚ´åPEEÅÔÔTØW³¥6JóøñãsçÎeïË¾Q+êkjj¢CFgõØ4³ ­DDDÐÑa9F0?ÌÌoüBááîaddDïúúú£wGmîÙâ*FæÇ¯ue¯C5¿ââbUUUg'gÚYé[HtvvR+**æGIù1ç;ÿáynLÒÐØØX555ÒM;;;MMMÛ#9F0?ÌÌoüââââµÀËÓÓ3,,ì©4ÄÄÄøùùñk¤°WW°1aÖ¬YCrò?e%åøøx.©¤Ì¹­­­¤¸doæ^A»2]sss®ìõ3Ô¶#ÌæÌæ7 õõõÉ¥TTTF©Pgäkmm522rvr&ti_\Üj¨CBBÃø$$$)Ï­~V§é7ß|Sêíí½~ýzæ«Uçª>ûì3Á·JJJù»àîî+Ìæóæó£ªªÊÃÝCPqiii9J÷ND¬½½=+++88xÕªU'OêVVVm"ó÷÷j6Ö¬YCêI&ºxñâYE©¥¥¥èµ¢òòrÒéììÔÖÖ²ððð7d5111,,Þ'½Áü`~0?`~0¿©¯¯'qù©««Û2¿±<B0ôíPËü~îëVåîîCR¥¡¡7Â¼=~üøìÙ³9ûsnÔßñUUÕ-I[ø»àéé9P_ee%W<I«¢¢2fÂü`~0?`~0¿qà9¿ÐÐÐQÚÖxØÈócÌ??((s/R1EEEÒÁagìÎ;Ùû²/^¸ÈµäÈc¤ÎïÑ£Gjjj±kcùãÎikkÃÞX`~0?0?ßX@áââbddäææötÛöÖÖÖé;7)..VRRbmUUU=:Ô-vuu/Æs#.))Fþ>|xâøCùþÒ$Ç°¶½***®®®¶¶¶"#ÂÑ|'''AÕ°±ñíÛ·a~æóæ7ÍÁúó£[æSéÏoØ466Î7ÕÚÚZAA!++KÊÉn«û^÷Ëdóóõõ-,,jR7êoÌ;H'g¿ñÖ_MMMggç@Ó·£®YXXÍo0?ÌÌoJ#Ûñèèè,]È,qs")`EEÅØìQdD$§SéiéZZZ½tÇ-½ôTÅ©QòòZ999~øøxRä±éüæóùóùÁüd±GGGA+]ssó±Ù<UUÕ   M/l151²Ä´»»»úwÕü£ç1999äÇláeKÑô0j·a~0?ó0?Ìï)GÌÇÇ',,LP)''7Ôtêëë÷íÛR^^>¤Â02ÅxyysÚGgÚîÝ»ÓÓÓ?.é_õÕÁ¼gÏüøñP=¦³³|wóæÍYYYÒ?«W]]Bù¨ªªÜçÌæóæóæ7þügÖÓÓ¯^Ød``0¤tÔÕÕýýýÃÃÃ­­­ílíD2H--­à²RSÓ   NþHõN? äoCÑÓ××·±¶¡¼TUUO<æóùóùMróËÏÏ!?cuÌjõëÖ3íËØagg&:õõõ¤üÇàÜÝÜ×¯_?ìUUUÍ3'5%ËMff&õù?ÏÞ]óQMww÷ð<ÜtÁÄmPRRÂ m0?ÌÌo··7ÙOÄò?y^^^jjj~~~]]]Ò§³oß>~©aòÉ:::ÃÎX\ÜÅKø	Æ®*½ôèGÛÚÚí1===³fÍâúðã:_,//ÇyóùÁüùÁüÆ©ùåææÎ7tÍÀÀ ##cH¢Æabb¶0ëBd+À?@NNîâÅCJ'%%%|Q¸àIAÊ·@UUÕÜ¹söYyyyUUÕ6?¸hÑ¢~jäj[S·~ñ_ôööÄc:;;555YõõõÍÏÏÇyóùÁüùÁüÆ#eeeJJJ;26½°ÉÊÒ*  `é,^¼XWWWP´FæG©_òòrsss~:Ë.srrbß9slË-é&É­üíÞ½ÛÙÉ`ÂÆ+WÊÄcttt[ý2åJ___ãÃü`~æ`~0?,ÑÖÖæmß¶lfîBW^köÞÊ¨ôÑÏÏ/,,ÌÙÙYútººº]\Ø0¸Ë#øòQÞ¹UWW'ã$(Áööv##£ÀÀÀÛwü2TÚªZ~$fð=LTSSóf;;;òæI<ÌæóæóÀtvvjhhê+¼W_ùÍ7ßÉÉÉM6MII)4$ñëj¥áÑ£Gäg´¢­­mrr2¹Z¿µ«æ/°´´Ü¼y³HuuuäCRö<Ü=êëëeè1UUU666´×ééé¸CfÌæÌæ7±ééé!aÙ¶ÏØ´i×/EEÅH¶¨prt²²²Ú½w¿)ÜÿþþÑ7)óùÁüùÁü¦AAAüøX¿$MMMÃKM²Og'çuëÖ#©ãÇëééq½±lIÚ¢¡¡áÞ?·òòòduuuÕéìÊÚuýúuÙV¿Âc1ÌÌo¢rëÖ-~_Äo¿ýöHÌÉÉQQQaOøÍ;×ÅÅexý0ÚäR«V­"466¦iVÒ>eeåììlÁº÷îÝ+z­èèG<xùÁü`~Àü`~6Æ®®.¹Îþýûe[E»|ùraaaBBÂÞ½F&*-99¹¼¼|xÄVVV^Ø$Øßß<2??ßÛÛÛÝÝãÆßñãÇImllâ7ÄïÍÜóæÍáuÚAÄ`~0?`~0¿§@NNV`@ Â|Ïù¤A±kc-,,­S(bË/	á·wRRR¿Ó|2cr¾ã7jjjÂc`~0?0?ßÄ µµlÏØÈØÇÇßG·#WPQQXA2G¯°°°Y³f566J.YWWgffFËð»9sæ(5³Ç b0?0?©©©aý+**Z³&''RÄnß¾]YYI.%ÃbEéïéé©¯¯§eX¥smm-i®ºº:) S¿Ú×ÛÛKB,èíe®ÙÔioo§ãòÎ;ïp$ÌæÌæ'3HzÈuÈ`Ó¶¦ñ&8(xÛ¶m2Y×Ò¥KÕÔÔ\ÍÍõôôdhN==¶©©©±±qKK`;333sw---???2­û:¨ô®¹¹¹  `ïÞ½ÜÀqÜËØÈx$Ý5KR\¬ªªJ:î0ÏA^^þðáÃ¸*Áü`~0?¦´ùuuuÅÅÅÑMîëlÐºxéêêN>ÝÖÖöÒ¥K0¿!©²rìÚX;gç>F8¼16Xzz:	W¾NUiDD££#W!KòJ§¿©mÈÈÐ8ZÒÕÕdq <xpúÝÓó~õÕWMMM***üæ +£VRæeØ¦ººdÛDâæDJ¿²²&ÌæÀÔ5¿W^y%;;»··´ÏÔÔæDEE±.B¢££%ÍïÊ+?ÉºÇÇü4)8ö¬ººº·iµµuLLÌ²¥Ëôôô6oÞ<SUUe¦q/ûÓ§O|mmmýEòí¬62eO2Á[·neíÍªù¨æÿ÷ÙÃkhh>·ú9oooÚË/Ë0J!!!Ë#øÙ[¹Âßßÿ' ä1d~ôÇù!ÒÓÚÚJæ'ódaæ7¶¶¶ú5MMMÖ³Fww·¾¾¾¤ùíÛ·¯JÖ;w®j²PPPfcccbbbeeåãã³uëÖ>ø@°Íyá,XàáîpæÌ¡FìÔ©S³fÍb=§8;;¹ºº:99dÌÅÅhÑ¢E´õÄÄDÉ¼±é 6ÖÑÁqûöíÜ2ñññü,ìEkåååñzï½÷rrr233O<)ØKÊ3åþiÈö@§ò»§fÅ~³gÏj:thbccÅÃ5)L¿JDlêDæ`~0úôÃ+((ÐûÆlÿ[Ôöd`VV+£VÒËÒÒÒÏÏOúZZ.bJJJd$ªªª¤täæ/1cÆ=«TTTÓ._¶Ö¢MÇÄÄD,066&SÜt» ÌO_O_XYYicm#(ó£µ(Ü2ÿñóìÙtÎRG"S.[º=Ú_ÿ¡)GGGv¤VD®(í¨íEm/23¿[·n(**N6>jkk¿÷Þ²Ê"¥yôèQøóÿìääÄæpßÊËËÃüFÔÔT;;;~&f¦f999CØÚµkIõOËÑQ£³¥¾¾¯q$÷±kcisssþ3²~7ýËs~¿zÎÏÆÆßÂHOOOð_hh(ûöÞ½¥¯)£;âS	r¿Ïù=v$GJ$?ÌæÌïâÅÏüÿ0'cÓEEE2É"y$7ÍJøhNww÷Ïµ½üoa~2ÄÑÑqCÜÁ#hÁÁÁÒ'q+--åkÑQspp())á¯©©¡9ì[BA»cÚô%K$·B9¡,Ú¶×ÈÈÈÂÜ!¯òóó£¼=yòäÜ¹sÙû²oÔßû¢>>ÅÅÅJJJ¬m/MäååÉäHõ.Ìæó@æG÷]ò¼[·nqæ÷ÙgÑ´²²²L²têÔ©ûJ===ibõêÕo½õMÐTTÌoÌ/>>	jjjÖÖÖ/½ôxW.beee>ÞÂ'ífÍ¥¥¥uèÐ!¾ù±ûík0::ZDeHFÏ9SWW7RVI*++ëëëiúæÍ¹rÉüÈÿÆC¨ÛÛÛ«««ßyçóçÏüH.ÌÀüÌodIôÁ&ùõööÒ´äxÃ£££cùòå×éîÕ«WI7i[ºººd0¿Ñ %%ÅÕÅëó<>(0U)&¿lhh¸wï^i"FZF¾Ê	ÜÑÈÐhñâÅ|ûa°Ú^A³ÒMT_>|øðèGIûîÜ¹3i<¤d.Ìæó0¿~ÐÖÖ&Ïcå|dcÝÝÝÛ·o§iÉV·cÌO&tvvþÒ×Å5>>ÞÓÓSÐN|N¤Ø±ôôtÄÍááádxË.#TTTd*3gÍ±<bÍókhÚÊúuëíììFØdþ~=óÕÌêßU³G&ÇÐ222bGjCÜæó0?óÚÚÚgúãÓO?ùMtùKII±·³×ÒÒ"c|VßBV<b[¶lù¥WSl¥`ii)¸sWWWÌ??  ÀÊÊ<fÇ#ô¢×èuïÞ½Aw¹¤¤dóæÍYYYÒÕÑØØXXXÈwÙ1ð:ÕwïÞýÂ/øøøÈ*?ùßà`'''Ö¶WAAîèMMMOk`~2´Æk_ûÒ¶¦©ªªJYæGÔ××óñSÈØ¡¡¡ÑÚÚ:ª9§Vÿ®:koÖõë×£¡¡AGGÇÑÑ144ÔÇÛvu.NMMÍL$ÇÎNÎ¿Íò¨zLRRIyHpHXX©iPPÐhóùÁüùk`~2§±±4hýºõ·âDDDH1².2*.ggg&É;wÔÃØØxaèB.â6())ÿùe4<eåèÑÜZáÂ±_Cõ9sæpPIÛØØ¼üòË0?óùóùÊÊJ??2èììRÄnß¾­¦¦fkkëëëknnnffFRõ¨æ­««K©ÞéwOÊ?t÷»R&BÑÓÓã÷_H/w~×3ÔÖÖZ[[êÁuuu¥¯)¶ÇÄÄÄ,Y¼¿]63ÌÀü`~®ù=3Ó§O×ÖÖÎÌÌùMZ[[+**òóókjj­Uì7bííígÎ¡È#¯boo¯©©©®®naaÑÐÐ0,566Î?Ö¬YyyÍ¼Qã`ÞA:TðFIYYZ	$µ°°Pd-»»`­áíÎP=&22bkvceeó0?£k~ÓúhÎÖ­[a~Sñ±êã¥K²2¶°°0--­¡>óGIÂôÍ¬ZIÊEÎwâø¡f)ac7f"6,//Ïï³RFAæP=&++ËÑÁo~>>>«V­ùÌÑ5¿EEEºÿðÃôÞmmmiúìÙ³d~tùÁüøÄÄÄð­ÅÝÝ=##cHØµk«ë¯ú±KOKß·aØcrÍ5¤ýR~fiµdÉñÃ¦¦&:ÃÙCÏ­~NMMmlÌ6affæáá¶5Ü7layêSlYóùÁü*ægiiIzÇï#íÇ¤94S]:Ãü&ùY[[óGòeÎD¤4ÊÊÊüü|AugRbÒÜ¹sGmJòFêf``>¨ÀUUU¹»¹yyihhÐZZ¿tcee5æ½Ãðööö5kÖð)))y¸¯M1Ìæ`~æ7Ä$úàpÂ'ÛÁ<`~Æü|||Ö<¿/mË.#4Ù_á54²³³ó÷ó×¥±|ÙrAWW×±ÜÍºº:[[Ás~zzzÃ(Ç b0?ÃüØ¸½K.e$ôNÓ¬îý¨íùIRPP`fj¶Ûvî:=ý'O§Iÿ.ÌÌÌÂ±µâãâSSR·§oçº5±²²ãE]]]tz¯ZÉïÕÅÔÔtzuÌæÀÓ1¿/öÛ¶÷Ò¥Kl"!!æó¡§§b``;¨-Õ××[[ÚÞ***JjýB'ÛÓêÉæÁü`~<ó#þö·¿YZZ*((pcxÐû*-Z4Æ»ó^yåsss:¸ÖÖÖÎI±ªªªmÛ¶¥§§WTTHcl´¼§§§ j¬1...99¹¼¼|F°tß8°yóæüü|íà10?ÌIb~ãÌÙ¹s§®®.×|UGG§¸¸xì#öñÇ'%&edü»³åÔT%%¥a;JûùÁü`~L-óëèèÈÎÎ=6ÌoÐÚÚª¨¨(è²NAAA¼Ý«l#ÖÝÝñÂÅyW­Zekk»%ieÞíììÆÛ¾Ãc`~0?SÂü~üñÇ·ß~[__ëÌæ7	¨©©aÝæmzaSÄòçV?>è02ØÍ7Éùj/×ÿõôô¤§§«ªªjkkÓjjjOõõõü,´UWWSæGR"ÈíûÈèÇ b0?Äüè¾K·Xccc®m²²òÚµk¿ýö[ß$ ¶¶ÖÑÑÑÞÎ«­­­©©¢¢âlÿ¯½ªªÀ?>krxÃÁyÄKyÈCE¡ÐÐPHmæÖdJ$CJiÉüµSä%ãW¯6®ð>2êO.ÆaVÓÂÔàÈb±Xý¹ç¿ïáy|¿ë,Öñ°ÏøxöÙOøÐ´C2±½¯íÝõÛ]r¦ÿí+oáyxxüp(áÙa111B½gÖÚÚzvèlÿ¶nÐ_PPüÜqX^ÃÄò#óò;wî²g%KKËûøRò¦ººº|ðAW×ü>iiiÆÆÆ_~ùåðM¬¯¯ïÌ3Û^ÚÖØØ8ðÆ¢Ò9ás¸ÉÇY³fÍ9ÓÑÑQ]EûäÏ´µµmkkÜ×n®1[PÙ·$uør8¶ Æ1Lù!?¢Q-?|&&&Buc^ä7þ233SÞZ§°µ¢¢¢aXGGGùîò½¯íýúo_¼dSS«««Â>u×âTeõ>ð£ Ã[XXÄÇÅ/[º,**Jþ3ð|qòC~Èh<Ë¯¶¶VïBä7¦knnÎÎÎNLLÊÕÎÎN­V«·Æ3))éé§ÜÄtïBo×w½½½6ì,ÙyþüyC¿+O¿°Ùaê£Z±288Xä'u_¨»ã£Ujkk+((?þ+tWËÓ)33S.ÏÉÉõ¼8!?äG4fä§P/66öÊ+Èo¬wðàA[[[åõ­yñó¬¬¬öïß/ggeëÊ/44tp¯ùéÞ<m,,,êêêOýéO*Þ^|ôý£7oÞ4ð×üÒÓÓMLL"##off&à3ðÑ~ë-ÖÖÖiii¢R9ÿüóÏãäüs ä÷¿é½ÏOþÖ"¿±Ûµk×LMMu_*[±ÒØØ¸½½ý¿üåtéëÖ®S.OIIüÎ¹ÛN¬ÿ]×r^Ý³³dgëÿ½ëÃÝìòÖçÉ-/JY¤ûàxà59kRK½ã£íííêé^]®(ÏçÁÇ01äüÆ§üÔ¿GÕÛ¶÷©§RÞùüîá,wu­úúú½µº?ü°òÜæÍV3fÌprròðð`·&Â8ùì_~Ùbï¾û®§§§ÞË.|qó6¨MtuuÉã7pù2£¢¢,,,núáº¹ÙÙÙM2åkiiqsuÓ»ú9sÊËËqòC~ÈùÝ¾üã¿ÿýï]\ØßýJ-òñö133ËÈÈ0|Ã"¿à `=ý¨kcµ¶¶þØ­b>___­VëãóÃ½¯X±BwÉ;v8hloµjÕ*å.d±3gÎ¨üpÀ^1|_]cc£<ñô¼@öÕW_5d;ß´iÓô®.D~Èù!?æ@ÈïÎ÷ÝweeeÈoKLLTv¿/þüx]eUìê_¬Vé­Ñh|íMåçç1'By×Ýºµë·nÝª|¶ººZ´ùt¦rË?ûÙÏ[üØ³ÍZeee¥o·¦¦ÆÞÞ^],é§IìYî]ngùËýàõ®.Ó<°À1Lù!?¢±*¿ûÛß¥K¦L¢»áá±Ø½·Í¢EÂ&¨ëW^1ðºuuuº¯	"SÊKnÂeKé~6mIZÀÃÖÖÖRoÄßÏ_o±à àC@mm­r^½ºXS®>uêÔììlüòcüß(Mo'ÊÉËËë®þ:à,XàîîàÀòòò¬¬¬ÂÂÂ[ëÚÙÙ©,óÓþtîÜ¹z÷®Õjet²F£é¿_Ñº*Yé¶²CõÁ'$$(¾ººún(»«GFFuW«_æ£À1Èù!?"äüF®¦¦&Qîk~rÞÖÖ¶½½·ÖÖÖæìì<ÓgfbbbtT´¥¥¥²ÝLÞ^Þnnÿ²D~^¾ñ§§çÚ5ku?)Ò»AYLÿßpÇ ?äüòÑÄX"¿øøxõPfá·Ü¡Æ"###""twbjjÚÒÒ¢»xhaÂBeõ¹ë|ðÁGQ¥áïï&]½zõW¿üÕª«Ôã¿-b¹ø©ÿÂ7o/AÈ8ðb#!£À1Èù!?"äüFº¶¶¶éÓ§»ºº:88ÄÄÄ(+[ï¶îîn!5å6;Lw[×ÖÖV'''ñ¥O±¦Hðçââ"KÚØØA»ºº¼wdÛKÛÎ5ËÎÎ6335k¯¯¯­­ímÍ5,6b2üò#B~ÈïþÔÛÛ+kjj÷jß÷·và"pÔËÝÜ¹sËÊÊÔeZZZ´Z­(M`djj*PÛðÌ¨¨¨µk×Ê½766ÊÇ¢ýCê19raýÀû4p±ÉQàäüòC~c;ôôtÕ:¢:î+pÏ=÷µµµ²ö ãã3Óg¦²Ì7öíÝ·uËÖÏ>ûl"Ç ?äüòÛÕÕÕYXX,^´xÓÆMÂ;o/ïäädÝÃlh459kt=4yòäØØØÏ|¼åÅ-o½õÖ©S§&Â(pòC~Èù!¿QWkkkeeeyy¹áÃ©­­9s¦±±ñÔ©StÒÜÜìåå¥»ñÔ%þþþ7l,ýMi[[ÛL¬½½½ªªª¬¬¬¾¾~Èfø(pòC~Èù!¿ÑÕLMMÃÂÂbbbÕÃfZ]=ô²aÇ5r^n3?/?!!ÁÜÜ6Ü;|ø°¥¥exxx|iï°Ç ?äGÈò·òkjjÒh4+Z¡®õöò^µjÕ=ÞìÌ3$üpàà¼õyêëéééÆÆÆ|òÉðMLï+Rõc!?äGü&ºü^zé¥è¨h½ãª988ÜãÍ¶´´8::.M_ùìÆgõ¹¶sçÎáXÿ¯(?/_,x/dâ&üùòC~c^~¹¹¹I?MÒÛ/©©©á· ¢qqq]Õ××ËçÎo/Þ´iËTþ;=Ù¼yóO¬­­íÑGµµµ¸0±ÿâÆô_ÃÄò#B~Èoª¬¬é3SIKR~K,6m²¹®»»ûK/íyuÏ7ß|Ó#_9¹ºº:thh'ÖÞÞnggûÃ»	$¸»¹ëÞãÊ"?üù!?"ä7Ñå×ÝÝíáá¡"==ÝÂÂByÝÎ´Ù 2kã1scúúú6oÞììì¬îØ/8(8,,L 6´ËÈÈ;w®úVE[[ÛÐY¡ÊW$ì³·³ß·oA~üòèòûþÖzÒàà`333kkkÚÚZÃ¯[QQ§·jÕÊÊêÂê2EEErV«µ´´hÑµk×=1¹®¨´®®Nï¸s.ÓºµëÜÝÝµ·úÝï~c!?äGüß÷öööAAANNNÍÍÍ_½²²R±MÝÉäÉtw¤'SRÿ9¸Åy¦¦¦û÷ïW?5kÖ,Ýyå$ÿôóõë;sÁ1Lù!?"äü²¸¸8²ï=9%%%ÙÙÙ~HÜºÿ¨Ë]«»ßó8::®Ï]ïîî^XX8T«ªª²µµU_ØË|:ÓÚÚZýV¾ðÂ¾¾¾êW!'??¿7âäGÈù!?ä÷Ï®]»&~RÞ§k&a!'ö¼º§´´tÛ¶m666Ë.=¶°LyÝÔ©Sjb,X²H÷qÎ·bÅå³ÝÝÝaaaÞ^ÞKR¤¥¥ùøøÇ±4pC~üù!¿±*¿ææfo½wéEGGoß¾]>[VVëííýØcé®îëënyq|T¶ä¨­­u¾UxxxvV¶zSÆÆÆC51yº·,'¦<<uÞÞ^yÀ>úhrrrQQÑxbabÈù!?ä7uvvZZZæçåëÊÓÓ³¦¦&77×ÉÉ)==]¼° A£Ñ466*vì|óÀß|óîMõd&75TÒ%%ýË~£¢¢rrrp11äüò34ÁøLÁß¦DoòëÌÖÖvîzYRyyyÿèWv|öÙgýo§­­M®²xÑbõxªÉ_qåO,WgccÓÒÒc!?äGüßûÿù·ß~ûóÏ?_½zµ©©éÏZ­6$$¤µµµ¢¢Bï<9­ÉY#òëéééSrK.?~ÜÉÉÉÍÍmÆr¥¥¥L¬³³³©©©½½ý¶KÊåÍÍÍÊªÛÚÚZ+++www9SWW7SøááäüòC~£½'OZXXkíµ&MH]¸pAþLo¯ÜV~^^^ýß<×ØØ(ÚyÍð<yòþýûEªÕÜWNNF£ñððPÐÙÖÖ¦.ÓÒÒòðÃ;;;ûøø#åZ666ÓÜ§É%Ãýf¾ÜÜë><üòC~üßHø"DKy4E]-+ÙèîOèfee¥»¶wñâÅâ³þ7¥»weÆJ;;;CÆ«LLØ7cÆ½ÍÊÃèììtppX°PÙQKvV¶pêÔ©Ê%r-oÁßðª¸¸XP«zNïááäüùòC~c£5kÖÌ3GoS³Éf555º­_¿^$·>omÎZÝ-<të¿UGRRRBB!ûðÃlãY ¼¼<88¸ÿÔ½D®kff6|ûjÖÝ ÞÃÃ1Èùò#äüÆFQQQ¢(½5¹&MRöäòý­Ý£4|Ø°³d§!ÜY¼xqSSSÿRöá§;ÙYÙîîîLì­·Þ`2ëÖ­ÓÛWyýW@ëþu¯ªªZ¾|yrròæÍßõm«kµÚx8ù!?B~üßØ(##cÞ¼yº É|:sòäÉÊÞÏ5+z¹èÝwß½yóæo*;û¾èÞTZZÈÒðÁvvvÙ õÀÀ@åaøùùôz³µµU·½ÈÉÉyè¡èìì¬wlß»ÍÒÒrÝÚu·x8ù!?B~üßØ¨±±ÑÄÄD9Ì²ÎÔÙÉY£ÑÈo±êêê%;¿øüÃoJx¤ÞrÄ¶ÛKÿÅÅÅ«{ôGÇ8p@Üfdd¤¾ X_ ·üÃÛSRTöÇÄÄ(7(ßSÝ7&Î	óØcÝ#||TªÇ ?äGÈòKÛö¾ñÆ?Ñ×/UUUÕ·ÿ¨rLÃ;|ø°00pÖ¬Y¢À^xÁð¢æÏ/b		>º²N9==QÊ"a¹¹¹«¿¿ÀÔÆÆæüùó®®®3<g:88ûÔWõþy½2EòÀîeJÝÝÝIIIr#zÇ ?äGÈòcûóëêêzíµ×6lØPYY¹sçÎ=¯î¹zõê oª¡¡¡¶¶¶µµuknn>666ª»hY¼x±àOô¶áâ?Qàãië=òç¼¦¦F¦»í³Ï>ôÓ$½uÁâÅù>úè£ÂM[[[¨öþÇ01äüò»§zzzª««w¼²ãüùówûRß°N¬°°00 PoÓÇ|¬ªªòæ¡®8Sbbbhhèà^ww·GDDDA~ÜÔ?R«ÕÖ××ãäüùòC~cR~~úiñöbßmÉqå×ÕÕ%ðËÏËÌ%.LtppøÅÞÞ^YÞßÏMÎ®¨Ñhý¿²²r¦ÏL]z.I]cò#äGÈù1ùÝ¸qCÙãÊ+ßß:<Ú®]»/_õþûï««På¯fyyyEEÅ¥KFX~Òµk×ä!	øpa³Ãy]gggFFrÐÐÐù««·îXN¦¦¦8ù!?B~üßXßÙ?ÝöÒ¶#ïQ^êkhhÐyyyÛÛÛºV¬Xaeeiff¶uëÖ	5±^ziîÜ¹zÇ,SâäüùòC~cC~W¯^ÝóêßUÈïwåöövssó|pIê8~¾~êR=6Ö6C»»Q>±¦¦&F£î­fÓÆM!Á!Ãz¤8ÃÄò#B~Èohêëëkø°aÛKÛÎ5ÓÝC0çîîîëë«wH4SSSeÓZÝ­%ä	eåÃ[ZZÅÆÄº¸¸ÄÅÅuwwãäüùòC~#çÞÞÞ^xaúôé³3gNîx+W®ì,Ùùæ7ÿþ÷¿ëª¬¬Ln*bNDÿ7´é¯vÙÒeóçÏ¿Ç/cwtt|ùñãÇGùS¥½½]p__ÙcòC~DÈï_:zôèO~òÏG+¿¼ÄFFF¾¾¾ÇOòKOO«),KKKÓjµµµµ?¶ð7þð?½é§Því­tw²2c¥F£×ßð96mºG^WWgaa²áëÖ®òñö¹ïÂ1Lò#òëííPåºÿ~9³k×.áÑ¸_cc£în-]vÛÍúúúÎ9³åÅ-ÕÕÕý_êÓ-22ÒÒÂÒw¦o~^¾Â>++«ÂÂBKKËGyDD(§	vvvêáqÞäiîÓ*++ùÃ1Lù!?"äwwTUù	Sw³õôô8;;÷_EEÅ'CÚGT__ÿÉ0÷«_ýJoS9Y[[?~±ÿüÏÿÜ^´]Nræ·)×3g±±ñ<`jjjff¶hÑ¢ûöýö·¿uttÿi4¤îµÞÿýÒÒRµÞ]ÿX'OoÞ#OLL|üñÇ?!Ã:uêÔ<Çcw51C~ÍÞmÈßúóÿ$ÔSågdd¤~V÷¼*?qLûvéÒ%1Pû0·cÇ9ásôPfnnþùç+È(ÞùÃ;[·l=zô¨7üÛÚÚäÿú±±±B=¦ÇC=$¿Ñ._¾¬Þ¸ZNNÕ¬Y¾¾¾²|UUÕoÿË/¿´°°ÐµRNqqq«W¯n'c'N`Lløºxñâÿ÷3Ã»páÂpLyò»C)))òûýÇúÿå7iÒ$õ³&&&ãfmokk«î®Fä¢|VôV¼½øÍo~ûí·¸ñ§zÊÓÓS9X¬­­åëÒ[lóæÍº;|yòçOþïxûºïÌÏË·µµ½¿ÇCcÝ%#Öö²¶hìÉï'ÿÕjÝËG9?nä÷ý­ý°þ¢£¢EfAAA®®®¾7oyïÈ®ßîúâó/Ë¸59kt_óóóë¿?Ñá§Vè.6;¬°°ð·/ÓÃÃc¦ÏÌÄÄÄ¸¸8ÌÌL~ÀpC~ÈùÊ¥K¾þúërF>¦¦¦'ùI---Û·oÏÊÊ*))û=×t®x±|EêQ×Qgg§YïxÑÑÑrGzK;õ>ýôÓÜKwwwEEEvvösÏ=wäÈ1w¤cÃÄ!?B~£Q~'O2eÊ¤IO>=Îä§ÖÑÑ±çÕ=r÷ØÚÚêíÃÏÓÓ³¦¦Fo1?ÿeKé.,Ã1Lò#?ò3°q ¿¾¾¾S§Nn-<sæî19î¥;v8;;++|7mÜíááÑbA;õ½)))×®]C~8!?äGüßÐwåÊòÝåo¼ñFGG!Ë÷öö¶´´té«óòògxÎÐjµ!!!­­­·]¬¢¢B£ÑxÍðê<ÕÕÕµ©©é^&&j[èììäÇ01äüòûßzzzêþ£®xñÅ¼Jmm­«ÛôéÓ­¬¬ª««^^&,ÛÛÛfbwwwss³hrÐáÕÕÕEFF2¼ÌÌÌ222ºººøÃ1Lù!?"äü¾?ûÇ³E/yï²Ù²!)L[þÄru,òÏö6ùåS§NT/þäGøÃ1Lù!?"ä7¡åwýúõßU^¹rå®®ð³ýLo;Üø¸øÑð]¨¬¬ôæ¡ûØfffý÷ H8!?äGü&üúúúä¡nyqKýõØÃÛÛ;;+[¥´´´bÅpïB¹¹¹9;;;11Q>Þö¯oaaáÜhý#Ñyyyñ§Ç01äüßDß×ûzÏ«*~W!¿w,X²HAÕò'EFD.[º,!!ÁÖÖöW^¦	×ÔÔØÙÙÅÇÅËÅÅÆY[[÷ß;Lee¥ÀTÙpÛ£abÈù!¿ñ,¿£ïÝñÊ/ÞËN[êêêìíí½ôYZZªÓ5Á!oWWÕ?R½¯+5Þ_¾üòK­V¸0Q=ñ¬Y³¤üÈá&üò@ò;þ|ÑËEuÿQgøýëììT¶ºÝ½·¨Ëw¦¯µµµÞªÕÈÈòòrÝk	Îw ¹º[   ½ûêMìðáÃ®®®3<g:88ÄÄÄðabÈù!¿"¿7n|ëà®ßîºzõê oDàåááagggiiÜÒÒ"«¨¨céilîÜ¹eeeÊµ8 öR®lø|-²±±q¸Õ¾ûêëëgÍ¥w_rÞ6ÅÊÄò·¹¦¦¦©©é^>c!?äGüÆüÎþñláÖÂ£ï½úäïF£yäG6mÜ$§	VVV¿îînSSSõÀÊêjÂD¹øÏÞÎ~ÅS+ËÃÃÂýüüq,#¤ÌÏËWÖêÊí<ÿüó¦®Ï]¯Þï¿fcxà&üòò»~ýúÞÜûÚÞ¯ÿöõ=>yóæé¾Ø6'|NNNÎ÷·¼acc£rWèíå¬ðNt¨°O=¹ººöß&£uuu...ÊùÔ·ôÉ­mÜ¸ÑÑÑQ¹Mùèîî¾nÝº!·2!&üòKòx5|Ø°³dçùóçäð»ÞÞÞÊöêI¨«|V0çããcll<uêÔå"B½³ÑÑÑÛ·o¿ãÝÍ«¿g­V+ÓØ½·§§§Ü°ïW^éÿ"òÃ1Lù!?"ä7ä÷Ùgo/þÕïoÜ¸1TcþüùéééºKLLbÅWÏÕµê)$8¤ªªêw'×		ÑÛ³¥¥¥!kabÈù!¿	!¿o¿ý¶ººzÇ+;¾øü¡prrRßc·&g­­­ÞFµºµ··ÛØØxN÷ôõõUWÚ.b¹¹¹¹|êwwíÚ5¹ý%©KTù/Z´h¬cpC~Èù%ùõõõ:ujË[Þ÷Ý!|©O·Õ«W[YYEEEEDD¼»æªªªðððü­V1'"àáxà7Þ0ðîdÂÄØXWW×°°0u÷.ÈÇ01äüßÄß+WJKK÷¾¶÷^vÚbHeeeååå.]xIY,66Vy¹.---.6.!!aúôéMMMwõµççç?ôÐCÆÆÆÎÎÎ6mRv%üpC~ÈùMDù)Çä(Þ^|ñâÅQ5'''Ýs×­]gjjªlüa`uuu-ÛYýÕÊVÃÈÇ01äüßD²%Ç¡ÿsèïÿû(Fdd¤ºC>qË¿ýÛ¿ÝÕ-ÈUt7+ÑÝS òÃ1Lù!?"ä7QäwãÆwß·èå¢+W®ÚituuÛ,--µZ­Õ/¼pWÇÒ«ó8:nrËuuu%%%UUU.~8!?äGüÆüÎþñì¶¶yïÈÍ7ÇÄXwÐÞîînQãg6èí>ZïÀ]\¢£¢CC4Í®]»øÃ1Lù!?"ä7¶å÷Í7ßTü®BNòkt"<EbbbâãâUö­þÅjQ]kk«ÞbË/TßSn6ÙLæÆÏabÈù!¿1)¿¾¾¾^øõgÿxvHÉ1&jkksvvöóóKLL¶´´Ü·o_ÿÅärÝùÊÉßßßýEabÈù!¿Q'¿>ø`gÉÎ7¼ùÍ7ßL´gIgggYYYffæÖ­[o»V«Õ;`TT!Ç#ÃÄò#B~£H~7nÜxë­·6¿°ùüùó<c~,[[[½cO6­¦¦Éà&üòòëëë;sæÌ·üN8ÁÓevìØáìì¼&goÓÆMÑQÑòOCöùL8!?äGüî¿ü:::Êwïúí®«W¯Þ÷½Ó]»v­¾¾¾®®NIkkkMM|wµCæ(//ÏØØxç­Vðp@uuµéÒ%y´(Ç01äüßh_oooÃ[;¦ì	åþÊ¯ªªÊlbb¢ÑhBCüüüìììü÷Xgg§ü1n¿ÕñãÇ§L:mÚ477·¶¶6~äpC~Èù"ù	YJKK_õûë×¯«ÞGù577[[[?ùó'ùì/gÏíêêZ_ ×ÖÖvtrJ&#ZU÷ó2Þ|__ßAìSÇC~Èù½ünÞ¼yä½#Â¾/>ÿ¢¿cîü²³³ccc<dgeënE!,**ßË/k4©ÊÉÙÉ?Õ8!?äGüî¿üÎ5ÛöÒ¶nû¢Ô_bbâ²¥ËMùyùzNIJJzúé§é®eYYYóçÏÏÍÍmii¹«ëþ×ý×ôéÓõmè¬ÐÃóScòC~DÈï¾ÉïÛo¿ÝûÚÞ=¯î¹zõê-s_óS¡ajjªl<«ÂÃÃé5¿ÒÒRÅQQQÎ»êåË--,õ^ósqqáO5abÈù!¿û&¿ÆÆÆÂ­6|Lö>¿ÖÖV¹SÝýó-I]âàà`ø»ôþù>¿yßÌ3ÙÂÇ01äüß_GGGeeåo¼!gqÌýÝ¶×ÔÔ4  @Ù¶wòäÉ³fÍòõõö544Ç=Êd¢£¢õÖÕº¹º]ºtÉpù?~|þüùlÛcòC~DÈï~ÊïæÍõÔï,ÙyñâEÃsß÷çWw«ööve~õõõÃtw111zò1cÆmÝ6ðÄä*nllmÄ1Lùò#ä7þåwög·VWW÷ôô~÷]~#@ÍÖÖ6?/_eßÊ'O6mb8!?äGüFü®_¿þæ7wì¼råÊÝÞætÌêÕ«ÝÜÜÒÓÓ³³²ìììöíÛÇÄpC~Èùù:ujË[¼wä®^êà©¬¬>zBBÂÝ¾¡ùá&üò»òûúo_ïm¯ä·Þ oÇ01ÃÄò#B~£Z~===Gß?ºãöA~8!?äüÆ­áòüIDATü.^¼XôrQuuõÍ7qòÃ1Lò#òÛ¼yóîÝ»KKK8&A~8!?B~üÆüöíÛ7$/õá&còC~DÈoTw·ÇíÅ1ÈÇ01äGÈò3imm­¬¬,//ò/ùá&üòC~£¨ÒÒRè¨èäääîînäc!?äGüÆcä+µ··_ýÕÊ1Ö6<³ÁÛË»  ùá&FÈù!¿ñæ¬¬¬ÄÄDõèºrN:ùá&FÈù!¿ñæa_zzº®üädllüp#äüßxsLAAATd.û-]æééüp#äüßxsL[[­­íâEöe>éààpðàAäc!?äGüÌ1MMM555ò±··÷þ~½NNNnnn3fÌÐh4¥¥¥£á»üpC~ÈùùÉù°Ùa®®®òÑÙÙ900ðÚµk÷ý«nmmmnnîêê%ßäcòC~DÈo<È/$$$2"rÓÆMÊÖðððÄÄDIÈÇ01äüßx_SS«««Ê>ezÖÖÖ²O&äcòC~DÈo¯¶¶6lvÞ^T¼¼¼ôþuuumß¾ýÑG]¼xqEEÅÈ¿P&5þüÜÜÜäc!?äGüîÚ1MMMÎÎÎz¯ùYXXè¾æ×ÙÙèíå½$uÉcsss7oÞH¾¯¨¨ÈÞÞ>!!aÙÒeQQQuuuÈÇ01äüò»kÇè½ÏO¥»ðsÏ=ççç§. g»wï/¿¥¥E$ªÏMNP;;»~Ýùá&üòòó!!!®®®¡¡¡·Ý¶W.Y±RwuðcKNN/¿¢¢B`ª·>Zí¥KabÈ9 ?B~Èo0Qöç'xú¿&ò[µj.¼ÒÒÒFR~ÑÑÑzò>:òÃ1Lù!?äGüÞ1¹¹¹³BféÂËÇÇ§¨¨hd¾üææf++«õ¹ëÕòçOÊ%ÝÝÝÈÇ01äÇ!?ä7Äéììtuuü­ZµjeÆJ??¿ÜÂ£  ÀÉÉ)---;+;))ÉÂÂâÐ¡C#ü]@~8!?äGü&ü¤ööva°oãÆÂ>¹¤¤¤dÝºuòQÎ÷*++ccc§OÐÐÐ «Òòòò¬¬¬ÂÂÂa]ÿüpC~ÈùMùéÕØØhkkjeeÕÜÜ<òiiiqpp©Ñh>üðCäcò#äGÈouúôé###å×|tttK|;6Êå×ÝÝ-ÞJ[¦¾ñ.%%ÅÃÃcäwï<sæÌ	Õ±üå666ÈÇ01äGÈßhÉÓÓó£>3ôòò3©©©û÷ï3»víJKKë/¿wÞyçoCÚg&Üu9âãã£·±­ÈU`tW·sùòåùþô§?ÉêîZNbÁÿ÷ÿÛ0t/ÉÄN<ÉØð%¿CN:ÅïÓO?!B~w©©©|´³³ëëë3===ÎÎÎýå÷ê«¯~<¤	b=:¸ëõßÍàÕ«?~<11ÑÒÒÒÖÖÖl²ÙÚµk÷0Þzë­iÓ¦é=ààà_~ùãaè^&61;qâcbLl´Mì>òEüM~`233åz¡îùÑ¹¶·µµÕÌÌLw7+ÙYÙBX·ö	ÌÏË+f>éêêºoß¾A<¹;¹SÝcäXYYÓv¬íeÝ%cm/kßàûî»ïRSS-M4I½ÜÄÄdËOZ³f»»û§V(×ó¡¡ÁÉÉIwíÊZ­vp£¤¤ÄÑÑqÙÒe"½½¼W¬X1Lß/äcòC~DÈoð?~ò^ù§Ð§§§çû[kû3hÊOÚ½·§§§±±±°ÏðcøÅÆÆê­¢/YïxqWSSããã#cêÔ©6m¾­LabÈù!¿ÁÔÐÐßÑÑ¡^²téÒ×_]ÎÈÇÔÔÔ1!¿ÁUUU®Ë¾Ïl°´´ár ?ÃÄò#B~#³³óOtKN<9eÊI&9::>zË¯½½]§¬&VNà¹sçþïòÃ1Lù!?"ä7'ùký¬F£NJJruuC ?ÃÄ!?B~ÈïþÔÒÒ²ûö¬¬¬±²/(äcòC~DÈùMabÈù!?äüÇ01äüòC~8Ç01äüòC~8òC~DÈù!?ÃÄù!?"äüabüò+éíí-//_¼xqrrrQQÑè?hòÃ1Lù!?"äüSgggHH·÷Ô%ròòòéêêB~8òC~DÈo¼Éoýúõ~~~6nRÚæ1Í£¨¨ùábbÈù!¿ñ&¿ÀÀÀ+UöÉé±Å%''#?CLù!?"ä7å·jÕ*]ù¥¥¥!?CLù!?"ä7å;+d®ü|YÛc!?äGüÆ¡ü:;;=<<«nÀ8òC~DÈoÊOÁ_nn®¿¿_ffæµk×ÆÖwùá&üòC~%&còC~DÈù!?Â1Lù!?"äüÁ1Lù!?"äü!&üòC~ÈÇ01B~Èù!?äc!?äGüpòÃ1Lùò#äüpÃ1Lùò#äüpÃ1Lù!?"äüá&üòC~Èá&üòC~ÈpC~Èù!?äcC~Èù!?äc!?äGüòÃ1Lò#B~8ùá&FÈù!?üpC~üù!?ÃÄpC~üù!?ÃÄpC~Èù!?bb8!?äGüòcb8!?äGüò#ÃÄò#B~ÈùábbÈù!?äüp11äüòC~8òC~DÈÇ ?ÃÄù!?"äcabÈ!?äcabÈ!?äcabÈù!?äGLÇ01äüòC~ÌÇ01äüòC~còC~DÈù!?CLù!?"äü!&üòC~ÈÇ01B~Èùáäc!?äGüpòÃ1Lùò#äüpÃ1Lùò#äüpÃ1Lù!?"äüá&üòC~ÈpC~Èù!?äG8!?äGüòÃ1ÄÄò#B~ÈùábbÈù!?äüp#äüA~8òC~DÈÇ ?ÃÄ!?B~c!ùååèèhddäëëìØ1äüp#äüÆ­üRSS÷ïß/gvíÚüabüÑ¸]__éééqvvÖûìï½9o¨GL11&ÆÄî­ÚÚZðAÈïî222ºíy""""oò4izÞÄÄï"Ñ¸V«íééùþÖÚ^9ÏwhÜÊoéÒ¥¯¿þº©©©|Æ­üN<9eÊI&9::>ï"Ñ¸!?""""ûòëÜ¯¾úê':ÝvñzuttèîïpâÌg¨&ÆslàN> ×ò÷÷WvÌsìn'Æslà.¢äÉ<Ç&üú·ãí·ßÎÈÈxñz!ÉoIåoÉDÏPMçØÀyzz~ôÑGræàÁ^^^<Ç11c'èyçwäÌÍÎÎçÑ_ÿãvÈ¯Kå7ÂË×KiáÂ---º8óªñ3<SSScÏ1«««ó÷÷ç9F4ä×ÿ¸ò¿çøøx9tùòåÛ.3^/¹o°c&Î|jb<Çìã?ÎÌÌä96ñ»c½½½æææòyðàAcDH~·ã¯ý«òÁþË×Kç3¡Ï1Cúî»ïRSS»ººxbb<ÇìèÑ£Ê~ûyMù|Üå§¢ÿ2ãõÁ9fâÌg¨&ÆsìW222¾úê+cÏ±»]óÃsh¢È¯ÿq;<==[[[ß¤ñññ·]f¼^28ÇLùÕÄx¥££çØ 'Æslàd>.þÖfÑm>­&®ü¿ÄýÛññÇûøøÈÿ£¢¢äæm¯nÝóg>C51cOÌÙÙYo$<Çîvb<ÇØÙ³ge>sçÎU^(å9F4þå§¼cbLÑøßæÍù>11&ÆÄ#¢	!?""""B~DDDDüù!?""""B~DDDDüùò#""""äGDDDDÈ!?""""B~DDDDÈ!?""""B~D4?±ÿ©©i@@À®]»ô<tèÐüùóÍÌÌd;;»/¾øBoÞÞ^¹ºÜ|óßé½<`¾qDDÈîU~º©åååõ_ÀÜÜüO>ÑÓ¡úÙêêjäGDühÔÉO÷µk×Ê...Ê%ü±üsÒ¤I%%%]]]rÉéÓ§ÝÜÜäÂÙ³gë^7%%E.ÌÌÌ/LDDÈî	RÊ_åüO<!ÿÜ¶mî.]ªüãÿ«÷ööZ[[Ëyß©rþÜ¹s±±±¦·zê©§DêÂõõõrkþþþGÕÀòYå³uuuÊ/_§:::*@>ÊyYF.ç;NDüßÿö×¿þUY·+ÞR.qqqþå/ø¦öïß¯¼à'çWß|óMågbb¢»9--Mù¬òrcÿÏ677ðt/W%úë_ÿZþ¹aÃ9#çùv!?"äwb'NP1222dëüùóe1åZ§Oó.4P~<ò³¯¯¯¸¸XÙ@Dù¬²ú855õ»[u¯+ËùcÇÉù'Oê±»»Ú´iâÂ½÷ê*òû~ýë_·¶¶ªË"¿e«z»Þvù	ûþ÷*ÿ433~ýõ×Ê?e1Ýë÷ßèD½ueÖó!?"ú_	¹Þ~ûmÅR³gÏ¾~ýººV«ÿüç?p;öì¹-"årCäw/ÕM%£²:((o4ò#"Z;vLïvÒÒ¥Kå¢¢"Ý+*Ûd¨ï«º­üäò"Ñ¯¾úJùç×_­ûYåÁÛ~iÊz^¥Ç!!?¢+?é7¿ùrá#Gt9hddTVV&Ìêëë;qâòrZDD,ð¿üEÎ;;;ëÝø)Snûb¡áòSÞØ§¼Ï¯««KyÛúYå¹¹¹òÍ~ýýýO]¿~ÝÎÎN.)((Z­V÷UL""B~DÈïÅÇÇËª]ôéeiiyáÂùlIIb,½ÛÉåÅÅÅ_ss³ò.Cue®îgûoùûöÛo+R¶çU64V^³ÌËËãÛMDüþíW_emm-?ñÄê¯¿þúÜ¹sMMM-9V¯^åÊåS²ðÙ³gõnGÙÂ·ÿÛìtâÄ	___ñÜKÿýùÕÕÕÉí+©¢¢B¹P<ªÈõ»ï¾ûþÖër^.Gò'"B~DDDDüùò#"""B~DDDDüùò#""""äGDDDDÈ!?""""B~DDDDÈ!?""""B~DDDDüùò#""""äGDDDDÈò#""""äGDDDDÈ!?""""ºý?U>'z±§ÖBIEND®B`
