# Supplementary material for: Cortical atrophy in chronic subdural hematoma from ultra-structures to physical properties
Source: Sci Rep. 2023 Feb 28;13:3400. doi: 10.1038/s41598-023-30135-8 (PMC9975247; doi:10.1038/s41598-023-30135-8)
Supplement: Supplementary file 7 — Supplementary Information 7. [file 41598_2023_30135_MOESM7_ESM.doc]

Custom Tables


Notes	
Output Created	04-AUG-2021 19:24:33	
Comments		
Input	Data	C:\Users\Placido\Desktop\articolo atrofia e sottodurale cronico\casi\analisi ult.sav	
	Active Dataset	Dataset1	
	Filter	<none>	
	Weight	<none>	
	Split File	<none>	
	N of Rows in Working Data File	190	
Syntax	CTABLES
  /VLABELS VARIABLES=Age Sex Side RCAindex MDPreop MDPost30 MDPost90 Shiftpre Shiftpost30
    Shiftpost90 KPSPreOp KPSPostOp
    DISPLAY=LABEL
  /TABLE Age [MEAN] + Sex [COUNT F40.0, UCOUNT F40.0, ROWPCT.COUNT PCT40.1] + Side [COUNT F40.0,
    UCOUNT F40.0, ROWPCT.COUNT PCT40.1] + RCAindex [MEAN] + MDPreop [MEAN] + MDPost30 [MEAN] + MDPost90
    [MEAN] + Shiftpre [MEAN] + Shiftpost30 [MEAN] + Shiftpost90 [MEAN] + KPSPreOp [MEAN] + KPSPostOp
    [MEAN]
  /CATEGORIES VARIABLES=Sex Side ORDER=A KEY=VALUE EMPTY=EXCLUDE
  /CRITERIA CILEVEL=95
  /TITLES
    TITLE='Characteristics of CSDH Group'
  /COMPARETEST TYPE=MEAN ALPHA=0.05 ADJUST=BONFERRONI ORIGIN=COLUMN INCLUDEMRSETS=YES
    CATEGORIES=ALLVISIBLE MEANSVARIANCE=TESTEDCATS MERGE=YES STYLE=SIMPLE SHOWSIG=NO.	
Resources	Processor Time	00:00:00,02	
	Elapsed Time	00:00:00,01	


Characteristics of CSDH Group	
	Mean	Count	
Age	79		
Sex	f		74	
	m		116	
Side	left		89	
	righ		101	
RCA index	,177		
MDPreop	22,962		
MDPost 30	10,683		
MDPost 90	4,308		
Shift pre	8,966		
Shift post 30	3,313		
Shift post 90	2		
KPS PreOp	58		
KPS PostOp	87		

ONEWAY MDPreop MDPost30 MDPost90 Shiftpre Shiftpost30 Shiftpost90 KPSPreOp KPSPostOp Age BY RCAindex
  /STATISTICS DESCRIPTIVES EFFECTS
  /MISSING ANALYSIS.


Oneway


Notes	
Output Created	04-AUG-2021 19:30:32	
Comments		
Input	Data	C:\Users\Placido\Desktop\articolo atrofia e sottodurale cronico\casi\analisi ult.sav	
	Active Dataset	Dataset1	
	Filter	<none>	
	Weight	<none>	
	Split File	<none>	
	N of Rows in Working Data File	190	
Missing Value Handling	Definition of Missing	User-defined missing values are treated as missing.	
	Cases Used	Statistics for each analysis are based on cases with no missing data for any variable in the analysis.	
Syntax	ONEWAY MDPreop MDPost30 MDPost90 Shiftpre Shiftpost30 Shiftpost90 KPSPreOp KPSPostOp Age BY RCAindex
  /STATISTICS DESCRIPTIVES EFFECTS
  /MISSING ANALYSIS.	
Resources	Processor Time	00:00:00,08	
	Elapsed Time	00:00:00,05	


ANOVA	
	Sum of Squares	df	Mean Square	F	Sig.	
MDPreop	Between Groups	5464,227	69	79,192	.	.	
	Within Groups	,000	120	,000			
	Total	5464,227	189				
MDPost 30	Between Groups	5331,706	69	77,271	74,678	,000	
	Within Groups	124,167	120	1,035			
	Total	5455,873	189				
MDPost 90	Between Groups	1278,709	69	18,532	3,832	,000	
	Within Groups	580,374	120	4,836			
	Total	1859,082	189				
Shift pre	Between Groups	2481,228	69	35,960	.	.	
	Within Groups	,000	120	,000			
	Total	2481,228	189				
Shift post 30	Between Groups	1186,010	69	17,189	375,023	,000	
	Within Groups	5,500	120	,046			
	Total	1191,510	189				
Shift post 90	Between Groups	130,216	69	1,887	1,272	,124	
	Within Groups	178,000	120	1,483			
	Total	308,216	189				
KPS PreOp	Between Groups	39255,263	69	568,917	.	.	
	Within Groups	,000	120	,000			
	Total	39255,263	189				
KPS PostOp	Between Groups	16844,211	69	244,119	.	.	
	Within Groups	,000	120	,000			
	Total	16844,211	189				
Age	Between Groups	11034,863	69	159,926	.	.	
	Within Groups	,000	120	,000			
	Total	11034,863	189				

CORRELATIONS
  /VARIABLES=Age RCAindex MDPreop MDPost30 MDPost90 Shiftpre Shiftpost30 Shiftpost90 KPSPreOp
    KPSPostOp
  /PRINT=TWOTAIL NOSIG
  /STATISTICS DESCRIPTIVES
  /MISSING=PAIRWISE.


Correlations


Notes	
Output Created	04-AUG-2021 19:35:52	
Comments		
Input	Data	C:\Users\Placido\Desktop\articolo atrofia e sottodurale cronico\casi\analisi ult.sav	
	Active Dataset	Dataset1	
	Filter	<none>	
	Weight	<none>	
	Split File	<none>	
	N of Rows in Working Data File	190	
Missing Value Handling	Definition of Missing	User-defined missing values are treated as missing.	
	Cases Used	Statistics for each pair of variables are based on all the cases with valid data for that pair.	
Syntax	CORRELATIONS
  /VARIABLES=Age RCAindex MDPreop MDPost30 MDPost90 Shiftpre Shiftpost30 Shiftpost90 KPSPreOp
    KPSPostOp
  /PRINT=TWOTAIL NOSIG
  /STATISTICS DESCRIPTIVES
  /MISSING=PAIRWISE.	
Resources	Processor Time	00:00:00,02	
	Elapsed Time	00:00:00,02	


Descriptive Statistics	
	Mean	Std. Deviation	N	
Age	78,56	7,641	190	
RCA index	,17691	,034633	190	
MDPreop	22,96211	5,376919	190	
MDPost 30	10,68263	5,372807	190	
MDPost 90	4,30832	3,136306	190	
Shift pre	8,96579	3,623284	190	
Shift post 30	3,31263	2,510833	190	
Shift post 90	1,88	1,277	190	
KPS PreOp	58,16	14,412	190	
KPS PostOp	86,63	9,440	190	


Correlations	
	Age	RCA index	MDPreop	MDPost 30	MDPost 90	
Age	Pearson Correlation	1	,512	,157	,261	,142	
	Sig. (2-tailed)		,000	,030	,000	,051	
	N	190	190	190	190	190	
RCA index	Pearson Correlation	,512	1	,286	,283	,059	
	Sig. (2-tailed)	,000		,000	,000	,419	
	N	190	190	190	190	190	
MDPreop	Pearson Correlation	,157	,286	1	,437	,090	
	Sig. (2-tailed)	,030	,000		,000	,216	
	N	190	190	190	190	190	
MDPost 30	Pearson Correlation	,261	,283	,437	1	,395	
	Sig. (2-tailed)	,000	,000	,000		,000	
	N	190	190	190	190	190	
MDPost 90	Pearson Correlation	,142	,059	,090	,395	1	
	Sig. (2-tailed)	,051	,419	,216	,000		
	N	190	190	190	190	190	
Shift pre	Pearson Correlation	-,224	,029	,177	-,059	-,026	
	Sig. (2-tailed)	,002	,686	,015	,421	,725	
	N	190	190	190	190	190	
Shift post 30	Pearson Correlation	-,129	-,090	,152	,348	,177	
	Sig. (2-tailed)	,075	,214	,037	,000	,014	
	N	190	190	190	190	190	
Shift post 90	Pearson Correlation	-,012	,026	-,079	,166	,133	
	Sig. (2-tailed)	,869	,723	,280	,022	,068	
	N	190	190	190	190	190	
KPS PreOp	Pearson Correlation	,037	-,255	-,155	-,071	,090	
	Sig. (2-tailed)	,610	,000	,033	,334	,219	
	N	190	190	190	190	190	
KPS PostOp	Pearson Correlation	-,334	-,333	-,073	-,212	-,117	
	Sig. (2-tailed)	,000	,000	,320	,003	,108	
	N	190	190	190	190	190	

Correlations	
	Shift pre	Shift post 30	Shift post 90	KPS PreOp	
Age	Pearson Correlation	-,224	-,129	-,012	,037	
	Sig. (2-tailed)	,002	,075	,869	,610	
	N	190	190	190	190	
RCA index	Pearson Correlation	,029	-,090	,026	-,255	
	Sig. (2-tailed)	,686	,214	,723	,000	
	N	190	190	190	190	
MDPreop	Pearson Correlation	,177	,152	-,079	-,155	
	Sig. (2-tailed)	,015	,037	,280	,033	
	N	190	190	190	190	
MDPost 30	Pearson Correlation	-,059	,348	,166	-,071	
	Sig. (2-tailed)	,421	,000	,022	,334	
	N	190	190	190	190	
MDPost 90	Pearson Correlation	-,026	,177	,133	,090	
	Sig. (2-tailed)	,725	,014	,068	,219	
	N	190	190	190	190	
Shift pre	Pearson Correlation	1	,565	,149	-,226	
	Sig. (2-tailed)		,000	,040	,002	
	N	190	190	190	190	
Shift post 30	Pearson Correlation	,565	1	,253	-,096	
	Sig. (2-tailed)	,000		,000	,187	
	N	190	190	190	190	
Shift post 90	Pearson Correlation	,149	,253	1	-,044	
	Sig. (2-tailed)	,040	,000		,548	
	N	190	190	190	190	
KPS PreOp	Pearson Correlation	-,226	-,096	-,044	1	
	Sig. (2-tailed)	,002	,187	,548		
	N	190	190	190	190	
KPS PostOp	Pearson Correlation	,051	-,046	,010	,417	
	Sig. (2-tailed)	,485	,531	,892	,000	
	N	190	190	190	190	

Correlations	
	KPS PostOp	
Age	Pearson Correlation	-,334	
	Sig. (2-tailed)	,000	
	N	190	
RCA index	Pearson Correlation	-,333	
	Sig. (2-tailed)	,000	
	N	190	
MDPreop	Pearson Correlation	-,073	
	Sig. (2-tailed)	,320	
	N	190	
MDPost 30	Pearson Correlation	-,212	
	Sig. (2-tailed)	,003	
	N	190	
MDPost 90	Pearson Correlation	-,117	
	Sig. (2-tailed)	,108	
	N	190	
Shift pre	Pearson Correlation	,051	
	Sig. (2-tailed)	,485	
	N	190	
Shift post 30	Pearson Correlation	-,046	
	Sig. (2-tailed)	,531	
	N	190	
Shift post 90	Pearson Correlation	,010	
	Sig. (2-tailed)	,892	
	N	190	
KPS PreOp	Pearson Correlation	,417	
	Sig. (2-tailed)	,000	
	N	190	
KPS PostOp	Pearson Correlation	1	
	Sig. (2-tailed)		
	N	190	

NONPAR CORR
  /VARIABLES=Age RCAindex MDPreop MDPost30 MDPost90 Shiftpre Shiftpost30 Shiftpost90 KPSPreOp
    KPSPostOp
  /PRINT=BOTH TWOTAIL NOSIG
  /MISSING=PAIRWISE.


Nonparametric Correlations


Notes	
Output Created	04-AUG-2021 19:35:52	
Comments		
Input	Data	C:\Users\Placido\Desktop\articolo atrofia e sottodurale cronico\casi\analisi ult.sav	
	Active Dataset	Dataset1	
	Filter	<none>	
	Weight	<none>	
	Split File	<none>	
	N of Rows in Working Data File	190	
Missing Value Handling	Definition of Missing	User-defined missing values are treated as missing.	
	Cases Used	Statistics for each pair of variables are based on all the cases with valid data for that pair.	
Syntax	NONPAR CORR
  /VARIABLES=Age RCAindex MDPreop MDPost30 MDPost90 Shiftpre Shiftpost30 Shiftpost90 KPSPreOp
    KPSPostOp
  /PRINT=BOTH TWOTAIL NOSIG
  /MISSING=PAIRWISE.	
Resources	Processor Time	00:00:00,02	
	Elapsed Time	00:00:00,03	
	Number of Cases Allowed	241979 cases	


Correlations	
	Age	RCA index	MDPreop	MDPost 30	MDPost 90	Shift pre	Shift post 30	Shift post 90	KPS PreOp	KPS PostOp	
Kendall's tau_b	Age	Correlation Coefficient	1,000	,388	,085	,143	,099	-,091	-,034	-,001	,077	-,266	
		Sig. (2-tailed)	.	,000	,098	,005	,059	,076	,514	,989	,172	,000	
		N	190	190	190	190	190	190	190	190	190	190	
	RCA index	Correlation Coefficient	,388	1,000	,210	,160	,046	,031	-,062	,018	-,146	-,223	
		Sig. (2-tailed)	,000	.	,000	,001	,367	,535	,224	,737	,008	,000	
		N	190	190	190	190	190	190	190	190	190	190	
	MDPreop	Correlation Coefficient	,085	,210	1,000	,367	,090	,160	,127	-,037	-,066	-,076	
		Sig. (2-tailed)	,098	,000	.	,000	,083	,002	,014	,506	,242	,185	
		N	190	190	190	190	190	190	190	190	190	190	
	MDPost 30	Correlation Coefficient	,143	,160	,367	1,000	,335	-,039	,226	,128	,014	-,235	
		Sig. (2-tailed)	,005	,001	,000	.	,000	,448	,000	,022	,807	,000	
		N	190	190	190	190	190	190	190	190	190	190	
	MDPost 90	Correlation Coefficient	,099	,046	,090	,335	1,000	,041	,197	,115	,017	-,164	
		Sig. (2-tailed)	,059	,367	,083	,000	.	,435	,000	,044	,762	,005	
		N	190	190	190	190	190	190	190	190	190	190	
	Shift pre	Correlation Coefficient	-,091	,031	,160	-,039	,041	1,000	,432	,127	-,144	,021	
		Sig. (2-tailed)	,076	,535	,002	,448	,435	.	,000	,024	,011	,709	
		N	190	190	190	190	190	190	190	190	190	190	
	Shift post 30	Correlation Coefficient	-,034	-,062	,127	,226	,197	,432	1,000	,196	-,030	-,142	
		Sig. (2-tailed)	,514	,224	,014	,000	,000	,000	.	,001	,604	,015	
		N	190	190	190	190	190	190	190	190	190	190	
	Shift post 90	Correlation Coefficient	-,001	,018	-,037	,128	,115	,127	,196	1,000	-,061	-,062	
		Sig. (2-tailed)	,989	,737	,506	,022	,044	,024	,001	.	,324	,322	
		N	190	190	190	190	190	190	190	190	190	190	
	KPS PreOp	Correlation Coefficient	,077	-,146	-,066	,014	,017	-,144	-,030	-,061	1,000	,380	
		Sig. (2-tailed)	,172	,008	,242	,807	,762	,011	,604	,324	.	,000	
		N	190	190	190	190	190	190	190	190	190	190	
	KPS PostOp	Correlation Coefficient	-,266	-,223	-,076	-,235	-,164	,021	-,142	-,062	,380	1,000	
		Sig. (2-tailed)	,000	,000	,185	,000	,005	,709	,015	,322	,000	.	
		N	190	190	190	190	190	190	190	190	190	190	
Spearman's rho	Age	Correlation Coefficient	1,000	,535	,126	,200	,138	-,126	-,036	,000	,094	-,330	
		Sig. (2-tailed)	.	,000	,082	,006	,058	,084	,620	,996	,197	,000	
		N	190	190	190	190	190	190	190	190	190	190	
	RCA index	Correlation Coefficient	,535	1,000	,301	,240	,066	,060	-,082	,024	-,189	-,298	
		Sig. (2-tailed)	,000	.	,000	,001	,365	,409	,261	,744	,009	,000	
		N	190	190	190	190	190	190	190	190	190	190	
	MDPreop	Correlation Coefficient	,126	,301	1,000	,493	,117	,225	,178	-,049	-,087	-,091	
		Sig. (2-tailed)	,082	,000	.	,000	,108	,002	,014	,504	,231	,212	
		N	190	190	190	190	190	190	190	190	190	190	
	MDPost 30	Correlation Coefficient	,200	,240	,493	1,000	,433	-,050	,296	,170	,018	-,301	
		Sig. (2-tailed)	,006	,001	,000	.	,000	,492	,000	,019	,807	,000	
		N	190	190	190	190	190	190	190	190	190	190	
	MDPost 90	Correlation Coefficient	,138	,066	,117	,433	1,000	,055	,261	,149	,024	-,204	
		Sig. (2-tailed)	,058	,365	,108	,000	.	,454	,000	,041	,747	,005	
		N	190	190	190	190	190	190	190	190	190	190	
	Shift pre	Correlation Coefficient	-,126	,060	,225	-,050	,055	1,000	,570	,163	-,176	,023	
		Sig. (2-tailed)	,084	,409	,002	,492	,454	.	,000	,024	,015	,750	
		N	190	190	190	190	190	190	190	190	190	190	
	Shift post 30	Correlation Coefficient	-,036	-,082	,178	,296	,261	,570	1,000	,241	-,042	-,172	
		Sig. (2-tailed)	,620	,261	,014	,000	,000	,000	.	,001	,568	,018	
		N	190	190	190	190	190	190	190	190	190	190	
	Shift post 90	Correlation Coefficient	,000	,024	-,049	,170	,149	,163	,241	1,000	-,071	-,072	
		Sig. (2-tailed)	,996	,744	,504	,019	,041	,024	,001	.	,329	,324	
		N	190	190	190	190	190	190	190	190	190	190	
	KPS PreOp	Correlation Coefficient	,094	-,189	-,087	,018	,024	-,176	-,042	-,071	1,000	,425	
		Sig. (2-tailed)	,197	,009	,231	,807	,747	,015	,568	,329	.	,000	
		N	190	190	190	190	190	190	190	190	190	190	
	KPS PostOp	Correlation Coefficient	-,330	-,298	-,091	-,301	-,204	,023	-,172	-,072	,425	1,000	
		Sig. (2-tailed)	,000	,000	,212	,000	,005	,750	,018	,324	,000	.	
		N	190	190	190	190	190	190	190	190	190	190	

REGRESSION
  /MISSING LISTWISE
  /STATISTICS COEFF OUTS R ANOVA
  /CRITERIA=PIN(.05) POUT(.10)
  /NOORIGIN
  /DEPENDENT RCAindex
  /METHOD=ENTER Age MDPreop MDPost30 MDPost90 Shiftpre Shiftpost30 Shiftpost90 KPSPreOp KPSPostOp
  /SCATTERPLOT=(*ZPRED ,*ZRESID)
  /RESIDUALS HISTOGRAM(ZRESID) NORMPROB(ZRESID).


Regression


Notes	
Output Created	04-AUG-2021 19:44:54	
Comments		
Input	Data	C:\Users\Placido\Desktop\articolo atrofia e sottodurale cronico\casi\analisi ult.sav	
	Active Dataset	Dataset1	
	Filter	<none>	
	Weight	<none>	
	Split File	<none>	
	N of Rows in Working Data File	190	
Missing Value Handling	Definition of Missing	User-defined missing values are treated as missing.	
	Cases Used	Statistics are based on cases with no missing values for any variable used.	
Syntax	REGRESSION
  /MISSING LISTWISE
  /STATISTICS COEFF OUTS R ANOVA
  /CRITERIA=PIN(.05) POUT(.10)
  /NOORIGIN
  /DEPENDENT RCAindex
  /METHOD=ENTER Age MDPreop MDPost30 MDPost90 Shiftpre Shiftpost30 Shiftpost90 KPSPreOp KPSPostOp
  /SCATTERPLOT=(*ZPRED ,*ZRESID)
  /RESIDUALS HISTOGRAM(ZRESID) NORMPROB(ZRESID).	
Resources	Processor Time	00:00:03,58	
	Elapsed Time	00:00:35,45	
	Memory Required	8624 bytes	
	Additional Memory Required for Residual Plots	552 bytes	


Variables Entered/Removed	
Model	Variables Entered	Variables Removed	Method	
1	KPS PostOp, Shift post 90, MDPreop, MDPost 90, Shift pre , Age, KPS PreOp, MDPost 30 , Shift post 30	.	Enter	


Model Summary	
Model	R	R Square	Adjusted R Square	Std. Error of the Estimate	
1	,648	,420	,391	,027029	


ANOVA	
Model	Sum of Squares	df	Mean Square	F	Sig.	
1	Regression	,095	9	,011	14,479	,000	
	Residual	,131	180	,001			
	Total	,227	189				


Coefficients	
Model	Unstandardized Coefficients	Standardized Coefficients	t	Sig.	
	B	Std. Error	Beta			
1	(Constant)	,036	,035		1,032	,303	
	Age	,002	,000	,440	6,757	,000	
	MDPreop	,001	,000	,094	1,378	,170	
	MDPost 30	,001	,001	,220	2,733	,007	
	MDPost 90	,000	,001	-,042	-,670	,503	
	Shift pre	,002	,001	,246	3,118	,002	
	Shift post 30	-,004	,001	-,286	-3,544	,001	
	Shift post 90	,001	,002	,037	,609	,543	
	KPS PreOp	,000	,000	-,169	-2,512	,013	
	KPS PostOp	,000	,000	-,092	-1,333	,184	


Residuals Statistics	
	Minimum	Maximum	Mean	Std. Deviation	N	
Predicted Value	,12316	,24943	,17691	,022443	190	
Residual	-,060993	,065492	,000000	,026377	190	
Std. Predicted Value	-2,395	3,231	,000	1,000	190	
Std. Residual	-2,257	2,423	,000	,976	190	


Bootstrap


Notes	
Output Created	09-AUG-2021 15:47:59	
Comments		
Input	Data	C:\Users\Placido\Desktop\articolo atrofia e sottodurale cronico\casi\analisi ult.sav	
	Active Dataset	Dataset1	
	Filter	<none>	
	Weight	<none>	
	Split File	<none>	
Syntax	BOOTSTRAP
  /SAMPLING METHOD=SIMPLE
  /VARIABLES TARGET=Age MDPreop MDPost30 KPSPreOp KPSPostOp INPUT=RCAindex
  /CRITERIA CILEVEL=95 CITYPE=PERCENTILE  NSAMPLES=1000
  /MISSING USERMISSING=EXCLUDE.	
Resources	Processor Time	00:00:00,02	
	Elapsed Time	00:00:00,01	


[Dataset1] C:\Users\Placido\Desktop\articolo atrofia e sottodurale cronico\casi\analisi ult.sav


Bootstrap Specifications	
Sampling Method	Simple	
Number of Samples	1000	
Confidence Interval Level	95,0%	
Confidence Interval Type	Percentile	

GLM Age MDPreop MDPost30 KPSPreOp KPSPostOp BY RCAindex
  /METHOD=SSTYPE(3)
  /INTERCEPT=INCLUDE
  /POSTHOC=RCAindex(BONFERRONI)
  /PLOT=PROFILE(RCAindex) TYPE=BAR ERRORBAR=NO MEANREFERENCE=NO
  /EMMEANS=TABLES(RCAindex)
  /CRITERIA=ALPHA(.05)
  /DESIGN= RCAindex.


BOOTSTRAP
  /SAMPLING METHOD=SIMPLE
  /VARIABLES TARGET=MDPreop MDPost30 KPSPreOp KPSPostOp Age INPUT=RCAindex
  /CRITERIA CILEVEL=95 CITYPE=PERCENTILE  NSAMPLES=1000
  /MISSING USERMISSING=EXCLUDE.


Bootstrap


Notes	
Output Created	09-AUG-2021 15:54:31	
Comments		
Input	Data	C:\Users\Placido\Desktop\articolo atrofia e sottodurale cronico\casi\analisi ult.sav	
	Active Dataset	Dataset1	
	Filter	<none>	
	Weight	<none>	
	Split File	<none>	
Syntax	BOOTSTRAP
  /SAMPLING METHOD=SIMPLE
  /VARIABLES TARGET=MDPreop MDPost30 KPSPreOp KPSPostOp Age INPUT=RCAindex
  /CRITERIA CILEVEL=95 CITYPE=PERCENTILE  NSAMPLES=1000
  /MISSING USERMISSING=EXCLUDE.	
Resources	Processor Time	00:00:00,02	
	Elapsed Time	00:00:00,02	


Bootstrap Specifications	
Sampling Method	Simple	
Number of Samples	1000	
Confidence Interval Level	95,0%	
Confidence Interval Type	Percentile	

GLM MDPreop MDPost30 KPSPreOp KPSPostOp Age BY RCAindex
  /METHOD=SSTYPE(3)
  /INTERCEPT=INCLUDE
  /PLOT=PROFILE(RCAindex) TYPE=BAR ERRORBAR=CI MEANREFERENCE=NO
  /PRINT=DESCRIPTIVE ETASQ OPOWER
  /CRITERIA=ALPHA(.05)
  /DESIGN= RCAindex.


General Linear Model


Notes	
Output Created	09-AUG-2021 15:54:31	
Comments		
Input	Data	C:\Users\Placido\Desktop\articolo atrofia e sottodurale cronico\casi\analisi ult.sav	
	Active Dataset	Dataset1	
	Filter	<none>	
	Weight	<none>	
	Split File	<none>	
	N of Rows in Working Data File	190190	
Missing Value Handling	Definition of Missing	User-defined missing values are treated as missing.	
	Cases Used	Statistics are based on all cases with valid data for all variables in the model.	
Syntax	GLM MDPreop MDPost30 KPSPreOp KPSPostOp Age BY RCAindex
  /METHOD=SSTYPE(3)
  /INTERCEPT=INCLUDE
  /PLOT=PROFILE(RCAindex) TYPE=BAR ERRORBAR=CI MEANREFERENCE=NO
  /PRINT=DESCRIPTIVE ETASQ OPOWER
  /CRITERIA=ALPHA(.05)
  /DESIGN= RCAindex.	
Resources	Processor Time	00:00:45,61	
	Elapsed Time	00:00:49,41	


Between-Subjects Factors	
	N	
RCA index	,100	2	
	,111	3	
	,120	3	
	,125	3	
	,127	3	
	,131	2	
	,131	3	
	,131	3	
	,134	3	
	,134	3	
	,141	2	
	,143	3	
	,144	2	
	,146	2	
	,146	3	
	,148	3	
	,148	3	
	,150	3	
	,152	3	
	,152	3	
	,156	3	
	,159	3	
	,159	3	
	,159	3	
	,160	3	
	,163	3	
	,167	3	
	,168	3	
	,170	2	
	,170	2	
	,171	3	
	,172	3	
	,173	2	
	,174	2	
	,175	2	
	,176	3	
	,177	3	
	,180	2	
	,181	2	
	,183	3	
	,185	3	
	,188	3	
	,188	3	
	,189	3	
	,190	2	
	,191	3	
	,192	2	
	,192	3	
	,194	2	
	,198	2	
	,198	2	
	,200	3	
	,200	3	
	,201	3	
	,207	3	
	,208	3	
	,210	3	
	,211	3	
	,213	3	
	,213	2	
	,214	3	
	,219	3	
	,221	2	
	,224	2	
	,224	3	
	,226	3	
	,226	3	
	,228	3	
	,254	3	
	,265	3	


Descriptive Statistics	
	RCA index	Mean	Std. Deviation	N	
MDPreop	,100	20,00000	,000000	2	
	,111	14,00000	,000000	3	
	,120	18,00000	,000000	3	
	,125	31,00000	,000000	3	
	,127	19,00000	,000000	3	
	,131	22,00000	,000000	2	
	,131	20,00000	,000000	3	
	,131	23,00000	,000000	3	
	,134	10,00000	,000000	3	
	,134	21,00000	,000000	3	
	,141	15,00000	,000000	2	
	,143	22,50000	,000000	3	
	,144	22,00000	,000000	2	
	,146	20,00000	,000000	2	
	,146	22,20000	,000000	3	
	,148	18,00000	,000000	3	
	,148	26,10000	,000000	3	
	,150	20,00000	,000000	3	
	,152	30,00000	,000000	3	
	,152	22,50000	,000000	3	
	,156	20,00000	,000000	3	
	,159	17,40000	,000000	3	
	,159	26,80000	,000000	3	
	,159	30,00000	,000000	3	
	,160	13,20000	,000000	3	
	,163	18,00000	,000000	3	
	,167	17,00000	,000000	3	
	,168	18,30000	,000000	3	
	,170	27,00000	,000000	2	
	,170	17,40000	,000000	2	
	,171	25,00000	,000000	3	
	,172	38,30000	,000000	3	
	,173	30,00000	,000000	2	
	,174	22,00000	,000000	2	
	,175	24,50000	,000000	2	
	,176	19,50000	,000000	3	
	,177	26,00000	,000000	3	
	,180	20,00000	,000000	2	
	,181	34,00000	,000000	2	
	,183	22,60000	,000000	3	
	,185	29,00000	,000000	3	
	,188	31,00000	,000000	3	
	,188	19,00000	,000000	3	
	,189	23,20000	,000000	3	
	,190	32,00000	,000000	2	
	,191	21,70000	,000000	3	
	,192	26,00000	,000000	2	
	,192	18,00000	,000000	3	
	,194	15,00000	,000000	2	
	,198	20,00000	,000000	2	
	,198	28,00000	,000000	2	
	,200	20,00000	,000000	3	
	,200	22,00000	,000000	3	
	,201	30,00000	,000000	3	
	,207	22,00000	,000000	3	
	,208	20,30000	,000000	3	
	,210	18,80000	,000000	3	
	,211	24,30000	,000000	3	
	,213	25,00000	,000000	3	
	,213	31,70000	,000000	2	
	,214	30,00000	,000000	3	
	,219	25,00000	,000000	3	
	,221	21,00000	,000000	2	
	,224	28,00000	,000000	2	
	,224	20,40000	,000000	3	
	,226	28,10000	,000000	3	
	,226	30,00000	,000000	3	
	,228	25,00000	,000000	3	
	,254	18,00000	,000000	3	
	,265	27,00000	,000000	3	
	Total	22,96211	5,376919	190	
MDPost 30	,100	9,50000	2,121320	2	
	,111	6,00000	,000000	3	
	,120	3,00000	,000000	3	
	,125	11,00000	,000000	3	
	,127	6,00000	,000000	3	
	,131	3,00000	,000000	2	
	,131	13,00000	,000000	3	
	,131	14,00000	,000000	3	
	,134	,00000	,000000	3	
	,134	16,00000	,000000	3	
	,141	3,00000	,000000	2	
	,143	8,00000	,000000	3	
	,144	13,00000	,000000	2	
	,146	6,00000	,000000	2	
	,146	5,00000	,000000	3	
	,148	13,00000	,000000	3	
	,148	17,70000	,000000	3	
	,150	10,00000	,000000	3	
	,152	15,40000	,000000	3	
	,152	12,40000	,000000	3	
	,156	8,00000	,000000	3	
	,159	5,00000	,000000	3	
	,159	17,60000	,000000	3	
	,159	16,00000	,000000	3	
	,160	5,00000	,000000	3	
	,163	14,00000	,000000	3	
	,167	9,33333	2,886751	3	
	,168	,00000	,000000	3	
	,170	12,40000	,000000	2	
	,170	8,80000	,000000	2	
	,171	18,80000	,000000	3	
	,172	3,00000	,000000	3	
	,173	12,00000	,000000	2	
	,174	10,00000	5,656854	2	
	,175	8,00000	,000000	2	
	,176	13,70000	,000000	3	
	,177	9,00000	5,196152	3	
	,180	8,50000	3,535534	2	
	,181	17,00000	,000000	2	
	,183	16,70000	,000000	3	
	,185	15,00000	,000000	3	
	,188	,00000	,000000	3	
	,188	13,00000	,000000	3	
	,189	7,80000	,000000	3	
	,190	12,10000	,000000	2	
	,191	15,00000	,000000	3	
	,192	12,00000	,000000	2	
	,192	4,00000	,000000	3	
	,194	11,80000	,000000	2	
	,198	,00000	,000000	2	
	,198	10,00000	,000000	2	
	,200	5,00000	,000000	3	
	,200	14,00000	,000000	3	
	,201	15,00000	,000000	3	
	,207	11,40000	,000000	3	
	,208	7,90000	,000000	3	
	,210	6,00000	,000000	3	
	,211	10,70000	,000000	3	
	,213	14,00000	,000000	3	
	,213	14,50000	,000000	2	
	,214	20,00000	,000000	3	
	,219	10,00000	,000000	3	
	,221	9,50000	2,121320	2	
	,224	15,00000	,000000	2	
	,224	12,50000	,000000	3	
	,226	17,20000	,000000	3	
	,226	22,70000	,000000	3	
	,228	8,00000	,000000	3	
	,254	9,00000	,000000	3	
	,265	21,00000	,000000	3	
	Total	10,68263	5,372807	190	
KPS PreOp	,100	80,00	,000	2	
	,111	60,00	,000	3	
	,120	60,00	,000	3	
	,125	60,00	,000	3	
	,127	90,00	,000	3	
	,131	40,00	,000	2	
	,131	60,00	,000	3	
	,131	40,00	,000	3	
	,134	60,00	,000	3	
	,134	60,00	,000	3	
	,141	60,00	,000	2	
	,143	60,00	,000	3	
	,144	60,00	,000	2	
	,146	60,00	,000	2	
	,146	60,00	,000	3	
	,148	80,00	,000	3	
	,148	60,00	,000	3	
	,150	60,00	,000	3	
	,152	60,00	,000	3	
	,152	60,00	,000	3	
	,156	40,00	,000	3	
	,159	80,00	,000	3	
	,159	60,00	,000	3	
	,159	70,00	,000	3	
	,160	60,00	,000	3	
	,163	60,00	,000	3	
	,167	60,00	,000	3	
	,168	40,00	,000	3	
	,170	80,00	,000	2	
	,170	60,00	,000	2	
	,171	60,00	,000	3	
	,172	60,00	,000	3	
	,173	40,00	,000	2	
	,174	60,00	,000	2	
	,175	80,00	,000	2	
	,176	60,00	,000	3	
	,177	80,00	,000	3	
	,180	40,00	,000	2	
	,181	60,00	,000	2	
	,183	70,00	,000	3	
	,185	60,00	,000	3	
	,188	60,00	,000	3	
	,188	60,00	,000	3	
	,189	60,00	,000	3	
	,190	10,00	,000	2	
	,191	60,00	,000	3	
	,192	80,00	,000	2	
	,192	40,00	,000	3	
	,194	60,00	,000	2	
	,198	40,00	,000	2	
	,198	80,00	,000	2	
	,200	60,00	,000	3	
	,200	70,00	,000	3	
	,201	40,00	,000	3	
	,207	50,00	,000	3	
	,208	70,00	,000	3	
	,210	70,00	,000	3	
	,211	50,00	,000	3	
	,213	60,00	,000	3	
	,213	60,00	,000	2	
	,214	60,00	,000	3	
	,219	40,00	,000	3	
	,221	40,00	,000	2	
	,224	40,00	,000	2	
	,224	70,00	,000	3	
	,226	40,00	,000	3	
	,226	10,00	,000	3	
	,228	60,00	,000	3	
	,254	40,00	,000	3	
	,265	70,00	,000	3	
	Total	58,16	14,412	190	
KPS PostOp	,100	100,00	,000	2	
	,111	90,00	,000	3	
	,120	100,00	,000	3	
	,125	90,00	,000	3	
	,127	100,00	,000	3	
	,131	90,00	,000	2	
	,131	80,00	,000	3	
	,131	80,00	,000	3	
	,134	90,00	,000	3	
	,134	90,00	,000	3	
	,141	90,00	,000	2	
	,143	90,00	,000	3	
	,144	90,00	,000	2	
	,146	90,00	,000	2	
	,146	90,00	,000	3	
	,148	90,00	,000	3	
	,148	80,00	,000	3	
	,150	90,00	,000	3	
	,152	80,00	,000	3	
	,152	90,00	,000	3	
	,156	80,00	,000	3	
	,159	90,00	,000	3	
	,159	90,00	,000	3	
	,159	90,00	,000	3	
	,160	80,00	,000	3	
	,163	90,00	,000	3	
	,167	90,00	,000	3	
	,168	90,00	,000	3	
	,170	100,00	,000	2	
	,170	70,00	,000	2	
	,171	90,00	,000	3	
	,172	90,00	,000	3	
	,173	90,00	,000	2	
	,174	90,00	,000	2	
	,175	100,00	,000	2	
	,176	90,00	,000	3	
	,177	100,00	,000	3	
	,180	80,00	,000	2	
	,181	90,00	,000	2	
	,183	80,00	,000	3	
	,185	90,00	,000	3	
	,188	100,00	,000	3	
	,188	90,00	,000	3	
	,189	90,00	,000	3	
	,190	60,00	,000	2	
	,191	80,00	,000	3	
	,192	100,00	,000	2	
	,192	90,00	,000	3	
	,194	100,00	,000	2	
	,198	90,00	,000	2	
	,198	90,00	,000	2	
	,200	90,00	,000	3	
	,200	90,00	,000	3	
	,201	60,00	,000	3	
	,207	80,00	,000	3	
	,208	90,00	,000	3	
	,210	50,00	,000	3	
	,211	70,00	,000	3	
	,213	90,00	,000	3	
	,213	80,00	,000	2	
	,214	80,00	,000	3	
	,219	80,00	,000	3	
	,221	90,00	,000	2	
	,224	90,00	,000	2	
	,224	90,00	,000	3	
	,226	90,00	,000	3	
	,226	80,00	,000	3	
	,228	90,00	,000	3	
	,254	70,00	,000	3	
	,265	80,00	,000	3	
	Total	86,63	9,440	190	
Age	,100	63,00	,000	2	
	,111	70,00	,000	3	
	,120	72,00	,000	3	
	,125	73,00	,000	3	
	,127	74,00	,000	3	
	,131	66,00	,000	2	
	,131	84,00	,000	3	
	,131	75,00	,000	3	
	,134	76,00	,000	3	
	,134	69,00	,000	3	
	,141	80,00	,000	2	
	,143	63,00	,000	3	
	,144	73,00	,000	2	
	,146	78,00	,000	2	
	,146	61,00	,000	3	
	,148	81,00	,000	3	
	,148	84,00	,000	3	
	,150	80,00	,000	3	
	,152	73,00	,000	3	
	,152	72,00	,000	3	
	,156	85,00	,000	3	
	,159	88,00	,000	3	
	,159	77,00	,000	3	
	,159	79,00	,000	3	
	,160	80,00	,000	3	
	,163	75,00	,000	3	
	,167	73,00	,000	3	
	,168	60,00	,000	3	
	,170	62,00	,000	2	
	,170	84,00	,000	2	
	,171	78,00	,000	3	
	,172	84,00	,000	3	
	,173	76,00	,000	2	
	,174	77,00	,000	2	
	,175	76,00	,000	2	
	,176	85,00	,000	3	
	,177	85,00	,000	3	
	,180	64,00	,000	2	
	,181	80,00	,000	2	
	,183	85,00	,000	3	
	,185	71,00	,000	3	
	,188	78,00	,000	3	
	,188	81,00	,000	3	
	,189	85,00	,000	3	
	,190	81,00	,000	2	
	,191	79,00	,000	3	
	,192	78,00	,000	2	
	,192	71,00	,000	3	
	,194	85,00	,000	2	
	,198	84,00	,000	2	
	,198	90,00	,000	2	
	,200	80,00	,000	3	
	,200	78,00	,000	3	
	,201	96,00	,000	3	
	,207	83,00	,000	3	
	,208	83,00	,000	3	
	,210	82,00	,000	3	
	,211	85,00	,000	3	
	,213	82,00	,000	3	
	,213	82,00	,000	2	
	,214	85,00	,000	3	
	,219	71,00	,000	3	
	,221	74,00	,000	2	
	,224	85,00	,000	2	
	,224	97,00	,000	3	
	,226	82,00	,000	3	
	,226	83,00	,000	3	
	,228	87,00	,000	3	
	,254	85,00	,000	3	
	,265	85,00	,000	3	
	Total	78,56	7,641	190	


Multivariate Tests	
Effect	Value	F	Hypothesis df	Error df	Sig.	
Intercept	Pillai's Trace	,994	19949,741	1,000	120,000	,000	
	Wilks' Lambda	,006	19949,741	1,000	120,000	,000	
	Hotelling's Trace	166,248	19949,741	1,000	120,000	,000	
	Roy's Largest Root	166,248	19949,741	1,000	120,000	,000	
RCAindex	Pillai's Trace	,977	74,678	69,000	120,000	,000	
	Wilks' Lambda	,023	74,678	69,000	120,000	,000	
	Hotelling's Trace	42,940	74,678	69,000	120,000	,000	
	Roy's Largest Root	42,940	74,678	69,000	120,000	,000	

Multivariate Tests	
Effect	Partial Eta Squared	Noncent. Parameter	Observed Power	
Intercept	Pillai's Trace	,994	19949,741	1,000	
	Wilks' Lambda	,994	19949,741	1,000	
	Hotelling's Trace	,994	19949,741	1,000	
	Roy's Largest Root	,994	19949,741	1,000	
RCAindex	Pillai's Trace	,977	5152,790	1,000	
	Wilks' Lambda	,977	5152,790	1,000	
	Hotelling's Trace	,977	5152,790	1,000	
	Roy's Largest Root	,977	5152,790	1,000	


Tests of Between-Subjects Effects	
Source	Dependent Variable	Type III Sum of Squares	df	Mean Square	F	
Corrected Model	MDPreop	5464,227	69	79,192	.	
	MDPost 30	5331,706	69	77,271	74,678	
	KPS PreOp	39255,263	69	568,917	.	
	KPS PostOp	16844,211	69	244,119	.	
	Age	11034,863	69	159,926	.	
Intercept	MDPreop	97542,144	1	97542,144	.	
	MDPost 30	20642,440	1	20642,440	19949,741	
	KPS PreOp	618135,000	1	618135,000	.	
	KPS PostOp	1386240,000	1	1386240,000	.	
	Age	1129430,400	1	1129430,400	.	
RCAindex	MDPreop	5464,227	69	79,192	.	
	MDPost 30	5331,706	69	77,271	74,678	
	KPS PreOp	39255,263	69	568,917	.	
	KPS PostOp	16844,211	69	244,119	.	
	Age	11034,863	69	159,926	.	
Error	MDPreop	,000	120	,000		
	MDPost 30	124,167	120	1,035		
	KPS PreOp	,000	120	,000		
	KPS PostOp	,000	120	,000		
	Age	,000	120	,000		
Total	MDPreop	105643,300	190			
	MDPost 30	27138,410	190			
	KPS PreOp	681900,000	190			
	KPS PostOp	1442800,000	190			
	Age	1183590,000	190			
Corrected Total	MDPreop	5464,227	189			
	MDPost 30	5455,873	189			
	KPS PreOp	39255,263	189			
	KPS PostOp	16844,211	189			
	Age	11034,863	189			

Tests of Between-Subjects Effects	
Source	Dependent Variable	Sig.	Partial Eta Squared	Noncent. Parameter	Observed Power	
Corrected Model	MDPreop	.	1,000	.	.	
	MDPost 30	,000	,977	5152,790	1,000	
	KPS PreOp	.	1,000	.	.	
	KPS PostOp	.	1,000	.	.	
	Age	.	1,000	.	.	
Intercept	MDPreop	.	1,000	.	.	
	MDPost 30	,000	,994	19949,741	1,000	
	KPS PreOp	.	1,000	.	.	
	KPS PostOp	.	1,000	.	.	
	Age	.	1,000	.	.	
RCAindex	MDPreop	.	1,000	.	.	
	MDPost 30	,000	,977	5152,790	1,000	
	KPS PreOp	.	1,000	.	.	
	KPS PostOp	.	1,000	.	.	
	Age	.	1,000	.	.	
Error	MDPreop					
	MDPost 30					
	KPS PreOp					
	KPS PostOp					
	Age					
Total	MDPreop					
	MDPost 30					
	KPS PreOp					
	KPS PostOp					
	Age					
Corrected Total	MDPreop					
	MDPost 30					
	KPS PreOp					
	KPS PostOp					
	Age					

ONEWAY Age MDPreop MDPost30 KPSPreOp KPSPostOp BY RCAindex
  /MISSING ANALYSIS.


Oneway


Notes	
Output Created	09-AUG-2021 15:57:58	
Comments		
Input	Data	C:\Users\Placido\Desktop\articolo atrofia e sottodurale cronico\casi\analisi ult.sav	
	Active Dataset	Dataset1	
	Filter	<none>	
	Weight	<none>	
	Split File	<none>	
	N of Rows in Working Data File	190	
Missing Value Handling	Definition of Missing	User-defined missing values are treated as missing.	
	Cases Used	Statistics for each analysis are based on cases with no missing data for any variable in the analysis.	
Syntax	ONEWAY Age MDPreop MDPost30 KPSPreOp KPSPostOp BY RCAindex
  /MISSING ANALYSIS.	
Resources	Processor Time	00:00:00,02	
	Elapsed Time	00:00:00,01	


ANOVA	
	Sum of Squares	df	Mean Square	F	Sig.	
Age	Between Groups	11034,863	69	159,926	.	.	
	Within Groups	,000	120	,000			
	Total	11034,863	189				
MDPreop	Between Groups	5464,227	69	79,192	.	.	
	Within Groups	,000	120	,000			
	Total	5464,227	189				
MDPost 30	Between Groups	5331,706	69	77,271	74,678	,000	
	Within Groups	124,167	120	1,035			
	Total	5455,873	189				
KPS PreOp	Between Groups	39255,263	69	568,917	.	.	
	Within Groups	,000	120	,000			
	Total	39255,263	189				
KPS PostOp	Between Groups	16844,211	69	244,119	.	.	
	Within Groups	,000	120	,000			
	Total	16844,211	189				

BOOTSTRAP
  /SAMPLING METHOD=SIMPLE
  /VARIABLES TARGET=RCAindex INPUT=  Age MDPreop MDPost30 KPSPreOp KPSPostOp
  /CRITERIA CILEVEL=95 CITYPE=PERCENTILE  NSAMPLES=1000
  /MISSING USERMISSING=EXCLUDE.


Bootstrap


Notes	
Output Created	09-AUG-2021 16:00:14	
Comments		
Input	Data	C:\Users\Placido\Desktop\articolo atrofia e sottodurale cronico\casi\analisi ult.sav	
	Active Dataset	Dataset1	
	Filter	<none>	
	Weight	<none>	
	Split File	<none>	
	N of Rows in Working Data File	190	
Syntax	BOOTSTRAP
  /SAMPLING METHOD=SIMPLE
  /VARIABLES TARGET=RCAindex INPUT=  Age MDPreop MDPost30 KPSPreOp KPSPostOp
  /CRITERIA CILEVEL=95 CITYPE=PERCENTILE  NSAMPLES=1000
  /MISSING USERMISSING=EXCLUDE.	
Resources	Processor Time	00:00:00,03	
	Elapsed Time	00:00:00,03	


Bootstrap Specifications	
Sampling Method	Simple	
Number of Samples	1000	
Confidence Interval Level	95,0%	
Confidence Interval Type	Percentile	

BOOTSTRAP
  /SAMPLING METHOD=SIMPLE
  /VARIABLES TARGET=RCAindex INPUT=  Age MDPreop KPSPreOp
  /CRITERIA CILEVEL=95 CITYPE=PERCENTILE  NSAMPLES=1000
  /MISSING USERMISSING=EXCLUDE.


REGRESSION
  /DESCRIPTIVES MEAN STDDEV CORR SIG N
  /MISSING LISTWISE
  /STATISTICS COEFF OUTS CI(95) R ANOVA
  /CRITERIA=PIN(.05) POUT(.10)
  /NOORIGIN
  /DEPENDENT RCAindex
  /METHOD=ENTER Age MDPreop KPSPreOp
  /SCATTERPLOT=(*ZPRED ,*ZRESID)
  /RESIDUALS HISTOGRAM(ZRESID) NORMPROB(ZRESID)
  /SAVE ZPRED.


BOOTSTRAP
  /SAMPLING METHOD=SIMPLE
  /VARIABLES TARGET=MDPreop MDPost30 MDPost90 Shiftpre Shiftpost30 Shiftpost90 KPSPreOp KPSPostOp
    INPUT=RCAindex Sex Age Side
  /CRITERIA CILEVEL=95 CITYPE=PERCENTILE  NSAMPLES=1000
  /MISSING USERMISSING=EXCLUDE.


Bootstrap


Notes	
Output Created	09-AUG-2021 16:09:30	
Comments		
Input	Data	C:\Users\Placido\Desktop\articolo atrofia e sottodurale cronico\casi\analisi ult.sav	
	Active Dataset	Dataset1	
	Filter	<none>	
	Weight	<none>	
	Split File	<none>	
Syntax	BOOTSTRAP
  /SAMPLING METHOD=SIMPLE
  /VARIABLES TARGET=MDPreop MDPost30 MDPost90 Shiftpre Shiftpost30 Shiftpost90 KPSPreOp KPSPostOp
    INPUT=RCAindex Sex Age Side
  /CRITERIA CILEVEL=95 CITYPE=PERCENTILE  NSAMPLES=1000
  /MISSING USERMISSING=EXCLUDE.	
Resources	Processor Time	00:00:00,03	
	Elapsed Time	00:00:00,03	


Bootstrap Specifications	
Sampling Method	Simple	
Number of Samples	1000	
Confidence Interval Level	95,0%	
Confidence Interval Type	Percentile	

GLM MDPreop MDPost30 MDPost90 Shiftpre Shiftpost30 Shiftpost90 KPSPreOp KPSPostOp BY RCAindex Sex
    Age Side
  /METHOD=SSTYPE(3)
  /INTERCEPT=INCLUDE
  /PRINT=DESCRIPTIVE ETASQ OPOWER
  /CRITERIA=ALPHA(.05)
  /DESIGN= RCAindex Sex Age Side RCAindex*Sex RCAindex*Age RCAindex*Side Sex*Age Sex*Side Age*Side
    RCAindex*Sex*Age RCAindex*Sex*Side RCAindex*Age*Side Sex*Age*Side RCAindex*Sex*Age*Side.


General Linear Model


Notes	
Output Created	09-AUG-2021 16:09:30	
Comments		
Input	Data	C:\Users\Placido\Desktop\articolo atrofia e sottodurale cronico\casi\analisi ult.sav	
	Active Dataset	Dataset1	
	Filter	<none>	
	Weight	<none>	
	Split File	<none>	
	N of Rows in Working Data File	21281	
Missing Value Handling	Definition of Missing	User-defined missing values are treated as missing.	
	Cases Used	Statistics are based on all cases with valid data for all variables in the model.	
Syntax	GLM MDPreop MDPost30 MDPost90 Shiftpre Shiftpost30 Shiftpost90 KPSPreOp KPSPostOp BY RCAindex Sex
    Age Side
  /METHOD=SSTYPE(3)
  /INTERCEPT=INCLUDE
  /PRINT=DESCRIPTIVE ETASQ OPOWER
  /CRITERIA=ALPHA(.05)
  /DESIGN= RCAindex Sex Age Side RCAindex*Sex RCAindex*Age RCAindex*Side Sex*Age Sex*Side Age*Side
    RCAindex*Sex*Age RCAindex*Sex*Side RCAindex*Age*Side Sex*Age*Side RCAindex*Sex*Age*Side.	
Resources	Processor Time	00:10:02,97	
	Elapsed Time	00:11:26,33	


Warnings	
The total number of pivot table cells across split files exceeds 2000000. This limit can be altered by using the SET MXCELLS command. If the working file was being created or modified during the current procedure, any changes were probably lost.	
Execution of this command stops.	


Between-Subjects Factors	
	N	
RCA index	,100	2	
	,111	3	
	,120	3	
	,125	3	
	,127	3	
	,131	2	
	,131	3	
	,131	3	
	,134	3	
	,134	3	
	,141	2	
	,143	3	
	,144	2	
	,146	2	
	,146	3	
	,148	3	
	,148	3	
	,150	3	
	,152	3	
	,152	3	
	,156	3	
	,159	3	
	,159	3	
	,159	3	
	,160	3	
	,163	3	
	,167	3	
	,168	3	
	,170	2	
	,170	2	
	,171	3	
	,172	3	
	,173	2	
	,174	2	
	,175	2	
	,176	3	
	,177	3	
	,180	2	
	,181	2	
	,183	3	
	,185	3	
	,188	3	
	,188	3	
	,189	3	
	,190	2	
	,191	3	
	,192	2	
	,192	3	
	,194	2	
	,198	2	
	,198	2	
	,200	3	
	,200	3	
	,201	3	
	,207	3	
	,208	3	
	,210	3	
	,211	3	
	,213	3	
	,213	2	
	,214	3	
	,219	3	
	,221	2	
	,224	2	
	,224	3	
	,226	3	
	,226	3	
	,228	3	
	,254	3	
	,265	3	
Sex	f	74	
	m	116	
Age	60	3	
	61	3	
	62	2	
	63	5	
	64	2	
	66	2	
	69	3	
	70	3	
	71	9	
	72	6	
	73	11	
	74	5	
	75	6	
	76	7	
	77	5	
	78	13	
	79	6	
	80	13	
	81	8	
	82	11	
	83	9	
	84	13	
	85	31	
	87	3	
	88	3	
	90	2	
	96	3	
	97	3	
Side	left	89	
	righ	101	


Descriptive Statistics	
	RCA index	Sex	Age	Side	Mean	Std. Deviation	N	
MDPreop	,100	m	63	righ	20,00000	,000000	2	
				Total	20,00000	,000000	2	
			Total	righ	20,00000	,000000	2	
				Total	20,00000	,000000	2	
		Total	63	righ	20,00000	,000000	2	
				Total	20,00000	,000000	2	
			Total	righ	20,00000	,000000	2	
				Total	20,00000	,000000	2	
	,111	m	70	righ	14,00000	,000000	3	
				Total	14,00000	,000000	3	
			Total	righ	14,00000	,000000	3	
				Total	14,00000	,000000	3	
		Total	70	righ	14,00000	,000000	3	
				Total	14,00000	,000000	3	
			Total	righ	14,00000	,000000	3	
				Total	14,00000	,000000	3	
	,120	m	72	righ	18,00000	,000000	3	
				Total	18,00000	,000000	3	
			Total	righ	18,00000	,000000	3	
				Total	18,00000	,000000	3	
		Total	72	righ	18,00000	,000000	3	
				Total	18,00000	,000000	3	
			Total	righ	18,00000	,000000	3	
				Total	18,00000	,000000	3	
	,125	m	73	left	31,00000	,000000	3	
				Total	31,00000	,000000	3	
			Total	left	31,00000	,000000	3	
				Total	31,00000	,000000	3	
		Total	73	left	31,00000	,000000	3	
				Total	31,00000	,000000	3	
			Total	left	31,00000	,000000	3	
				Total	31,00000	,000000	3	
	,127	f	74	righ	19,00000	,000000	3	
				Total	19,00000	,000000	3	
			Total	righ	19,00000	,000000	3	
				Total	19,00000	,000000	3	
		Total	74	righ	19,00000	,000000	3	
				Total	19,00000	,000000	3	
			Total	righ	19,00000	,000000	3	
				Total	19,00000	,000000	3	
	,131	m	66	left	22,00000	,000000	2	
				Total	22,00000	,000000	2	
			Total	left	22,00000	,000000	2	
				Total	22,00000	,000000	2	
		Total	66	left	22,00000	,000000	2	
				Total	22,00000	,000000	2	
			Total	left	22,00000	,000000	2	
				Total	22,00000	,000000	2	
	,131	f	84	righ	20,00000	,000000	3	
				Total	20,00000	,000000	3	
			Total	righ	20,00000	,000000	3	
				Total	20,00000	,000000	3	
		Total	84	righ	20,00000	,000000	3	
				Total	20,00000	,000000	3	
			Total	righ	20,00000	,000000	3	
				Total	20,00000	,000000	3	
	,131	m	75	righ	23,00000	,000000	3	
				Total	23,00000	,000000	3	
			Total	righ	23,00000	,000000	3	
				Total	23,00000	,000000	3	
		Total	75	righ	23,00000	,000000	3	
				Total	23,00000	,000000	3	
			Total	righ	23,00000	,000000	3	
				Total	23,00000	,000000	3	
	,134	m	76	left	10,00000	,000000	3	
				Total	10,00000	,000000	3	
			Total	left	10,00000	,000000	3	
				Total	10,00000	,000000	3	
		Total	76	left	10,00000	,000000	3	
				Total	10,00000	,000000	3	
			Total	left	10,00000	,000000	3	
				Total	10,00000	,000000	3	
	,134	m	69	righ	21,00000	,000000	3	
				Total	21,00000	,000000	3	
			Total	righ	21,00000	,000000	3	
				Total	21,00000	,000000	3	
		Total	69	righ	21,00000	,000000	3	
				Total	21,00000	,000000	3	
			Total	righ	21,00000	,000000	3	
				Total	21,00000	,000000	3	
	,141	m	80	righ	15,00000	,000000	2	
				Total	15,00000	,000000	2	
			Total	righ	15,00000	,000000	2	
				Total	15,00000	,000000	2	
		Total	80	righ	15,00000	,000000	2	
				Total	15,00000	,000000	2	
			Total	righ	15,00000	,000000	2	
				Total	15,00000	,000000	2	
	,143	f	63	righ	22,50000	,000000	3	
				Total	22,50000	,000000	3	
			Total	righ	22,50000	,000000	3	
				Total	22,50000	,000000	3	
		Total	63	righ	22,50000	,000000	3	
				Total	22,50000	,000000	3	
			Total	righ	22,50000	,000000	3	
				Total	22,50000	,000000	3	
	,144	f	73	righ	22,00000	,000000	2	
				Total	22,00000	,000000	2	
			Total	righ	22,00000	,000000	2	
				Total	22,00000	,000000	2	
		Total	73	righ	22,00000	,000000	2	
				Total	22,00000	,000000	2	
			Total	righ	22,00000	,000000	2	
				Total	22,00000	,000000	2	
	,146	m	78	righ	20,00000	,000000	2	
				Total	20,00000	,000000	2	
			Total	righ	20,00000	,000000	2	
				Total	20,00000	,000000	2	
		Total	78	righ	20,00000	,000000	2	
				Total	20,00000	,000000	2	
			Total	righ	20,00000	,000000	2	
				Total	20,00000	,000000	2	
	,146	f	61	left	22,20000	,000000	3	
				Total	22,20000	,000000	3	
			Total	left	22,20000	,000000	3	
				Total	22,20000	,000000	3	
		Total	61	left	22,20000	,000000	3	
				Total	22,20000	,000000	3	
			Total	left	22,20000	,000000	3	
				Total	22,20000	,000000	3	
	,148	f	81	righ	18,00000	,000000	3	
				Total	18,00000	,000000	3	
			Total	righ	18,00000	,000000	3	
				Total	18,00000	,000000	3	
		Total	81	righ	18,00000	,000000	3	
				Total	18,00000	,000000	3	
			Total	righ	18,00000	,000000	3	
				Total	18,00000	,000000	3	
	,148	f	84	left	26,10000	,000000	3	
				Total	26,10000	,000000	3	
			Total	left	26,10000	,000000	3	
				Total	26,10000	,000000	3	
		Total	84	left	26,10000	,000000	3	
				Total	26,10000	,000000	3	
			Total	left	26,10000	,000000	3	
				Total	26,10000	,000000	3	
	,150	f	80	left	20,00000	,000000	3	
				Total	20,00000	,000000	3	
			Total	left	20,00000	,000000	3	
				Total	20,00000	,000000	3	
		Total	80	left	20,00000	,000000	3	
				Total	20,00000	,000000	3	
			Total	left	20,00000	,000000	3	
				Total	20,00000	,000000	3	
	,152	m	73	left	30,00000	,000000	3	
				Total	30,00000	,000000	3	
			Total	left	30,00000	,000000	3	
				Total	30,00000	,000000	3	
		Total	73	left	30,00000	,000000	3	
				Total	30,00000	,000000	3	
			Total	left	30,00000	,000000	3	
				Total	30,00000	,000000	3	
	,152	m	72	righ	22,50000	,000000	3	
				Total	22,50000	,000000	3	
			Total	righ	22,50000	,000000	3	
				Total	22,50000	,000000	3	
		Total	72	righ	22,50000	,000000	3	
				Total	22,50000	,000000	3	
			Total	righ	22,50000	,000000	3	
				Total	22,50000	,000000	3	
	,156	m	85	left	20,00000	,000000	3	
				Total	20,00000	,000000	3	
			Total	left	20,00000	,000000	3	
				Total	20,00000	,000000	3	
		Total	85	left	20,00000	,000000	3	
				Total	20,00000	,000000	3	
			Total	left	20,00000	,000000	3	
				Total	20,00000	,000000	3	
	,159	m	88	righ	17,40000	,000000	3	
				Total	17,40000	,000000	3	
			Total	righ	17,40000	,000000	3	
				Total	17,40000	,000000	3	
		Total	88	righ	17,40000	,000000	3	
				Total	17,40000	,000000	3	
			Total	righ	17,40000	,000000	3	
				Total	17,40000	,000000	3	
	,159	m	77	left	26,80000	,000000	3	
				Total	26,80000	,000000	3	
			Total	left	26,80000	,000000	3	
				Total	26,80000	,000000	3	
		Total	77	left	26,80000	,000000	3	
				Total	26,80000	,000000	3	
			Total	left	26,80000	,000000	3	
				Total	26,80000	,000000	3	
	,159	f	79	righ	30,00000	,000000	3	
				Total	30,00000	,000000	3	
			Total	righ	30,00000	,000000	3	
				Total	30,00000	,000000	3	
		Total	79	righ	30,00000	,000000	3	
				Total	30,00000	,000000	3	
			Total	righ	30,00000	,000000	3	
				Total	30,00000	,000000	3	
	,160	f	80	righ	13,20000	,000000	3	
				Total	13,20000	,000000	3	
			Total	righ	13,20000	,000000	3	
				Total	13,20000	,000000	3	
		Total	80	righ	13,20000	,000000	3	
				Total	13,20000	,000000	3	
			Total	righ	13,20000	,000000	3	
				Total	13,20000	,000000	3	
	,163	m	75	left	18,00000	,000000	3	
				Total	18,00000	,000000	3	
			Total	left	18,00000	,000000	3	
				Total	18,00000	,000000	3	
		Total	75	left	18,00000	,000000	3	
				Total	18,00000	,000000	3	
			Total	left	18,00000	,000000	3	
				Total	18,00000	,000000	3	
	,167	m	73	left	17,00000	,000000	3	
				Total	17,00000	,000000	3	
			Total	left	17,00000	,000000	3	
				Total	17,00000	,000000	3	
		Total	73	left	17,00000	,000000	3	
				Total	17,00000	,000000	3	
			Total	left	17,00000	,000000	3	
				Total	17,00000	,000000	3	
	,168	f	60	left	18,30000	,000000	3	
				Total	18,30000	,000000	3	
			Total	left	18,30000	,000000	3	
				Total	18,30000	,000000	3	
		Total	60	left	18,30000	,000000	3	
				Total	18,30000	,000000	3	
			Total	left	18,30000	,000000	3	
				Total	18,30000	,000000	3	
	,170	m	62	righ	27,00000	,000000	2	
				Total	27,00000	,000000	2	
			Total	righ	27,00000	,000000	2	
				Total	27,00000	,000000	2	
		Total	62	righ	27,00000	,000000	2	
				Total	27,00000	,000000	2	
			Total	righ	27,00000	,000000	2	
				Total	27,00000	,000000	2	
	,170	f	84	righ	17,40000	,000000	2	
				Total	17,40000	,000000	2	
			Total	righ	17,40000	,000000	2	
				Total	17,40000	,000000	2	
		Total	84	righ	17,40000	,000000	2	
				Total	17,40000	,000000	2	
			Total	righ	17,40000	,000000	2	
				Total	17,40000	,000000	2	
	,171	f	78	left	25,00000	,000000	3	
				Total	25,00000	,000000	3	
			Total	left	25,00000	,000000	3	
				Total	25,00000	,000000	3	
		Total	78	left	25,00000	,000000	3	
				Total	25,00000	,000000	3	
			Total	left	25,00000	,000000	3	
				Total	25,00000	,000000	3	
	,172	f	84	righ	38,30000	,000000	3	
				Total	38,30000	,000000	3	
			Total	righ	38,30000	,000000	3	
				Total	38,30000	,000000	3	
		Total	84	righ	38,30000	,000000	3	
				Total	38,30000	,000000	3	
			Total	righ	38,30000	,000000	3	
				Total	38,30000	,000000	3	
	,173	m	76	righ	30,00000	,000000	2	
				Total	30,00000	,000000	2	
			Total	righ	30,00000	,000000	2	
				Total	30,00000	,000000	2	
		Total	76	righ	30,00000	,000000	2	
				Total	30,00000	,000000	2	
			Total	righ	30,00000	,000000	2	
				Total	30,00000	,000000	2	
	,174	m	77	left	22,00000	,000000	2	
				Total	22,00000	,000000	2	
			Total	left	22,00000	,000000	2	
				Total	22,00000	,000000	2	
		Total	77	left	22,00000	,000000	2	
				Total	22,00000	,000000	2	
			Total	left	22,00000	,000000	2	
				Total	22,00000	,000000	2	
	,175	m	76	left	24,50000	,000000	2	
				Total	24,50000	,000000	2	
			Total	left	24,50000	,000000	2	
				Total	24,50000	,000000	2	
		Total	76	left	24,50000	,000000	2	
				Total	24,50000	,000000	2	
			Total	left	24,50000	,000000	2	
				Total	24,50000	,000000	2	
	,176	m	85	left	19,50000	,000000	3	
				Total	19,50000	,000000	3	
			Total	left	19,50000	,000000	3	
				Total	19,50000	,000000	3	
		Total	85	left	19,50000	,000000	3	
				Total	19,50000	,000000	3	
			Total	left	19,50000	,000000	3	
				Total	19,50000	,000000	3	
	,177	m	85	left	26,00000	,000000	3	
				Total	26,00000	,000000	3	
			Total	left	26,00000	,000000	3	
				Total	26,00000	,000000	3	
		Total	85	left	26,00000	,000000	3	
				Total	26,00000	,000000	3	
			Total	left	26,00000	,000000	3	
				Total	26,00000	,000000	3	
	,180	f	64	righ	20,00000	,000000	2	
				Total	20,00000	,000000	2	
			Total	righ	20,00000	,000000	2	
				Total	20,00000	,000000	2	
		Total	64	righ	20,00000	,000000	2	
				Total	20,00000	,000000	2	
			Total	righ	20,00000	,000000	2	
				Total	20,00000	,000000	2	
	,181	f	80	left	34,00000	,000000	2	
				Total	34,00000	,000000	2	
			Total	left	34,00000	,000000	2	
				Total	34,00000	,000000	2	
		Total	80	left	34,00000	,000000	2	
				Total	34,00000	,000000	2	
			Total	left	34,00000	,000000	2	
				Total	34,00000	,000000	2	
	,183	f	85	righ	22,60000	,000000	3	
				Total	22,60000	,000000	3	
			Total	righ	22,60000	,000000	3	
				Total	22,60000	,000000	3	
		Total	85	righ	22,60000	,000000	3	
				Total	22,60000	,000000	3	
			Total	righ	22,60000	,000000	3	
				Total	22,60000	,000000	3	
	,185	m	71	righ	29,00000	,000000	3	
				Total	29,00000	,000000	3	
			Total	righ	29,00000	,000000	3	
				Total	29,00000	,000000	3	
		Total	71	righ	29,00000	,000000	3	
				Total	29,00000	,000000	3	
			Total	righ	29,00000	,000000	3	
				Total	29,00000	,000000	3	
	,188	m	78	righ	31,00000	,000000	3	
				Total	31,00000	,000000	3	
			Total	righ	31,00000	,000000	3	
				Total	31,00000	,000000	3	
		Total	78	righ	31,00000	,000000	3	
				Total	31,00000	,000000	3	
			Total	righ	31,00000	,000000	3	
				Total	31,00000	,000000	3	
	,188	m	81	righ	19,00000	,000000	3	
				Total	19,00000	,000000	3	
			Total	righ	19,00000	,000000	3	
				Total	19,00000	,000000	3	
		Total	81	righ	19,00000	,000000	3	
				Total	19,00000	,000000	3	
			Total	righ	19,00000	,000000	3	
				Total	19,00000	,000000	3	
	,189	m	85	righ	23,20000	,000000	3	
				Total	23,20000	,000000	3	
			Total	righ	23,20000	,000000	3	
				Total	23,20000	,000000	3	
		Total	85	righ	23,20000	,000000	3	
				Total	23,20000	,000000	3	
			Total	righ	23,20000	,000000	3	
				Total	23,20000	,000000	3	
	,190	f	81	righ	32,00000	,000000	2	
				Total	32,00000	,000000	2	
			Total	righ	32,00000	,000000	2	
				Total	32,00000	,000000	2	
		Total	81	righ	32,00000	,000000	2	
				Total	32,00000	,000000	2	
			Total	righ	32,00000	,000000	2	
				Total	32,00000	,000000	2	
	,191	f	79	left	21,70000	,000000	3	
				Total	21,70000	,000000	3	
			Total	left	21,70000	,000000	3	
				Total	21,70000	,000000	3	
		Total	79	left	21,70000	,000000	3	
				Total	21,70000	,000000	3	
			Total	left	21,70000	,000000	3	
				Total	21,70000	,000000	3	
	,192	f	78	left	26,00000	,000000	2	
				Total	26,00000	,000000	2	
			Total	left	26,00000	,000000	2	
				Total	26,00000	,000000	2	
		Total	78	left	26,00000	,000000	2	
				Total	26,00000	,000000	2	
			Total	left	26,00000	,000000	2	
				Total	26,00000	,000000	2	
	,192	f	71	left	18,00000	,000000	3	
				Total	18,00000	,000000	3	
			Total	left	18,00000	,000000	3	
				Total	18,00000	,000000	3	
		Total	71	left	18,00000	,000000	3	
				Total	18,00000	,000000	3	
			Total	left	18,00000	,000000	3	
				Total	18,00000	,000000	3	
	,194	m	85	left	15,00000	,000000	2	
				Total	15,00000	,000000	2	
			Total	left	15,00000	,000000	2	
				Total	15,00000	,000000	2	
		Total	85	left	15,00000	,000000	2	
				Total	15,00000	,000000	2	
			Total	left	15,00000	,000000	2	
				Total	15,00000	,000000	2	
	,198	m	84	righ	20,00000	,000000	2	
				Total	20,00000	,000000	2	
			Total	righ	20,00000	,000000	2	
				Total	20,00000	,000000	2	
		Total	84	righ	20,00000	,000000	2	
				Total	20,00000	,000000	2	
			Total	righ	20,00000	,000000	2	
				Total	20,00000	,000000	2	
	,198	m	90	left	28,00000	,000000	2	
				Total	28,00000	,000000	2	
			Total	left	28,00000	,000000	2	
				Total	28,00000	,000000	2	
		Total	90	left	28,00000	,000000	2	
				Total	28,00000	,000000	2	
			Total	left	28,00000	,000000	2	
				Total	28,00000	,000000	2	
	,200	m	80	left	20,00000	,000000	3	
				Total	20,00000	,000000	3	
			Total	left	20,00000	,000000	3	
				Total	20,00000	,000000	3	
		Total	80	left	20,00000	,000000	3	
				Total	20,00000	,000000	3	
			Total	left	20,00000	,000000	3	
				Total	20,00000	,000000	3	
	,200	m	78	left	22,00000	,000000	3	
				Total	22,00000	,000000	3	
			Total	left	22,00000	,000000	3	
				Total	22,00000	,000000	3	
		Total	78	left	22,00000	,000000	3	
				Total	22,00000	,000000	3	
			Total	left	22,00000	,000000	3	
				Total	22,00000	,000000	3	
	,201	m	96	left	30,00000	,000000	3	
				Total	30,00000	,000000	3	
			Total	left	30,00000	,000000	3	
				Total	30,00000	,000000	3	
		Total	96	left	30,00000	,000000	3	
				Total	30,00000	,000000	3	
			Total	left	30,00000	,000000	3	
				Total	30,00000	,000000	3	
	,207	m	83	left	22,00000	,000000	3	
				Total	22,00000	,000000	3	
			Total	left	22,00000	,000000	3	
				Total	22,00000	,000000	3	
		Total	83	left	22,00000	,000000	3	
				Total	22,00000	,000000	3	
			Total	left	22,00000	,000000	3	
				Total	22,00000	,000000	3	
	,208	f	83	righ	20,30000	,000000	3	
				Total	20,30000	,000000	3	
			Total	righ	20,30000	,000000	3	
				Total	20,30000	,000000	3	
		Total	83	righ	20,30000	,000000	3	
				Total	20,30000	,000000	3	
			Total	righ	20,30000	,000000	3	
				Total	20,30000	,000000	3	
	,210	m	82	left	18,80000	,000000	3	
				Total	18,80000	,000000	3	
			Total	left	18,80000	,000000	3	
				Total	18,80000	,000000	3	
		Total	82	left	18,80000	,000000	3	
				Total	18,80000	,000000	3	
			Total	left	18,80000	,000000	3	
				Total	18,80000	,000000	3	
	,211	m	85	left	24,30000	,000000	3	
				Total	24,30000	,000000	3	
			Total	left	24,30000	,000000	3	
				Total	24,30000	,000000	3	
		Total	85	left	24,30000	,000000	3	
				Total	24,30000	,000000	3	
			Total	left	24,30000	,000000	3	
				Total	24,30000	,000000	3	
	,213	m	82	left	25,00000	,000000	3	
				Total	25,00000	,000000	3	
			Total	left	25,00000	,000000	3	
				Total	25,00000	,000000	3	
		Total	82	left	25,00000	,000000	3	
				Total	25,00000	,000000	3	
			Total	left	25,00000	,000000	3	
				Total	25,00000	,000000	3	
	,213	m	82	righ	31,70000	,000000	2	
				Total	31,70000	,000000	2	
			Total	righ	31,70000	,000000	2	
				Total	31,70000	,000000	2	
		Total	82	righ	31,70000	,000000	2	
				Total	31,70000	,000000	2	
			Total	righ	31,70000	,000000	2	
				Total	31,70000	,000000	2	
	,214	m	85	righ	30,00000	,000000	3	
				Total	30,00000	,000000	3	
			Total	righ	30,00000	,000000	3	
				Total	30,00000	,000000	3	
		Total	85	righ	30,00000	,000000	3	
				Total	30,00000	,000000	3	
			Total	righ	30,00000	,000000	3	
				Total	30,00000	,000000	3	
	,219	f	71	righ	25,00000	,000000	3	
				Total	25,00000	,000000	3	
			Total	righ	25,00000	,000000	3	
				Total	25,00000	,000000	3	
		Total	71	righ	25,00000	,000000	3	
				Total	25,00000	,000000	3	
			Total	righ	25,00000	,000000	3	
				Total	25,00000	,000000	3	
	,221	f	74	righ	21,00000	,000000	2	
				Total	21,00000	,000000	2	
			Total	righ	21,00000	,000000	2	
				Total	21,00000	,000000	2	
		Total	74	righ	21,00000	,000000	2	
				Total	21,00000	,000000	2	
			Total	righ	21,00000	,000000	2	
				Total	21,00000	,000000	2	
	,224	m	85	righ	28,00000	,000000	2	
				Total	28,00000	,000000	2	
			Total	righ	28,00000	,000000	2	
				Total	28,00000	,000000	2	
		Total	85	righ	28,00000	,000000	2	
				Total	28,00000	,000000	2	
			Total	righ	28,00000	,000000	2	
				Total	28,00000	,000000	2	
	,224	f	97	righ	20,40000	,000000	3	
				Total	20,40000	,000000	3	
			Total	righ	20,40000	,000000	3	
				Total	20,40000	,000000	3	
		Total	97	righ	20,40000	,000000	3	
				Total	20,40000	,000000	3	
			Total	righ	20,40000	,000000	3	
				Total	20,40000	,000000	3	
	,226	f	82	left	28,10000	,000000	3	
				Total	28,10000	,000000	3	
			Total	left	28,10000	,000000	3	
				Total	28,10000	,000000	3	
		Total	82	left	28,10000	,000000	3	
				Total	28,10000	,000000	3	
			Total	left	28,10000	,000000	3	
				Total	28,10000	,000000	3	
	,226	f	83	righ	30,00000	,000000	3	
				Total	30,00000	,000000	3	
			Total	righ	30,00000	,000000	3	
				Total	30,00000	,000000	3	
		Total	83	righ	30,00000	,000000	3	
				Total	30,00000	,000000	3	
			Total	righ	30,00000	,000000	3	
				Total	30,00000	,000000	3	
	,228	m	87	righ	25,00000	,000000	3	
				Total	25,00000	,000000	3	
			Total	righ	25,00000	,000000	3	
				Total	25,00000	,000000	3	
		Total	87	righ	25,00000	,000000	3	
				Total	25,00000	,000000	3	
			Total	righ	25,00000	,000000	3	
				Total	25,00000	,000000	3	
	,254	m	85	left	18,00000	,000000	3	
				Total	18,00000	,000000	3	
			Total	left	18,00000	,000000	3	
				Total	18,00000	,000000	3	
		Total	85	left	18,00000	,000000	3	
				Total	18,00000	,000000	3	
			Total	left	18,00000	,000000	3	
				Total	18,00000	,000000	3	
	,265	m	85	righ	27,00000	,000000	3	
				Total	27,00000	,000000	3	
			Total	righ	27,00000	,000000	3	
				Total	27,00000	,000000	3	
		Total	85	righ	27,00000	,000000	3	
				Total	27,00000	,000000	3	
			Total	righ	27,00000	,000000	3	
				Total	27,00000	,000000	3	
	Total	f	60	left	18,30000	,000000	3	
				Total	18,30000	,000000	3	
			61	left	22,20000	,000000	3	
				Total	22,20000	,000000	3	
			63	righ	22,50000	,000000	3	
				Total	22,50000	,000000	3	
			64	righ	20,00000	,000000	2	
				Total	20,00000	,000000	2	
			71	left	18,00000	,000000	3	
				righ	25,00000	,000000	3	
				Total	21,50000	3,834058	6	
			73	righ	22,00000	,000000	2	
				Total	22,00000	,000000	2	
			74	righ	19,80000	1,095445	5	
				Total	19,80000	1,095445	5	
			78	left	25,40000	,547723	5	
				Total	25,40000	,547723	5	
			79	left	21,70000	,000000	3	
				righ	30,00000	,000000	3	
				Total	25,85000	4,546097	6	
			80	left	25,60000	7,668116	5	
				righ	13,20000	,000000	3	
				Total	20,95000	8,647873	8	
			81	righ	23,60000	7,668116	5	
				Total	23,60000	7,668116	5	
			82	left	28,10000	,000000	3	
				Total	28,10000	,000000	3	
			83	righ	25,15000	5,312909	6	
				Total	25,15000	5,312909	6	
			84	left	26,10000	,000000	3	
				righ	26,21250	10,067123	8	
				Total	26,18182	8,422923	11	
			85	righ	22,60000	,000000	3	
				Total	22,60000	,000000	3	
			97	righ	20,40000	,000000	3	
				Total	20,40000	,000000	3	
			Total	left	23,50714	4,500694	28	
				righ	23,10217	6,221843	46	
				Total	23,25541	5,603060	74	
		m	62	righ	27,00000	,000000	2	
				Total	27,00000	,000000	2	
			63	righ	20,00000	,000000	2	
				Total	20,00000	,000000	2	
			66	left	22,00000	,000000	2	
				Total	22,00000	,000000	2	
			69	righ	21,00000	,000000	3	
				Total	21,00000	,000000	3	
			70	righ	14,00000	,000000	3	
				Total	14,00000	,000000	3	
			71	righ	29,00000	,000000	3	
				Total	29,00000	,000000	3	
			72	righ	20,25000	2,464752	6	
				Total	20,25000	2,464752	6	
			73	left	26,00000	6,763875	9	
				Total	26,00000	6,763875	9	
			75	left	18,00000	,000000	3	
				righ	23,00000	,000000	3	
				Total	20,50000	2,738613	6	
			76	left	15,80000	7,941977	5	
				righ	30,00000	,000000	2	
				Total	19,85714	9,489970	7	
			77	left	24,88000	2,629068	5	
				Total	24,88000	2,629068	5	
			78	left	22,00000	,000000	3	
				righ	26,60000	6,024948	5	
				Total	24,87500	5,139136	8	
			80	left	20,00000	,000000	3	
				righ	15,00000	,000000	2	
				Total	18,00000	2,738613	5	
			81	righ	19,00000	,000000	3	
				Total	19,00000	,000000	3	
			82	left	21,90000	3,395880	6	
				righ	31,70000	,000000	2	
				Total	24,35000	5,368160	8	
			83	left	22,00000	,000000	3	
				Total	22,00000	,000000	3	
			84	righ	20,00000	,000000	2	
				Total	20,00000	,000000	2	
			85	left	20,78824	3,671322	17	
				righ	26,96364	2,688967	11	
				Total	23,21429	4,483114	28	
			87	righ	25,00000	,000000	3	
				Total	25,00000	,000000	3	
			88	righ	17,40000	,000000	3	
				Total	17,40000	,000000	3	
			90	left	28,00000	,000000	2	
				Total	28,00000	,000000	2	
			96	left	30,00000	,000000	3	
				Total	30,00000	,000000	3	
			Total	left	22,26557	5,230771	61	
				righ	23,34000	5,247525	55	
				Total	22,77500	5,243644	116	
		Total	60	left	18,30000	,000000	3	
				Total	18,30000	,000000	3	
			61	left	22,20000	,000000	3	
				Total	22,20000	,000000	3	
			62	righ	27,00000	,000000	2	
				Total	27,00000	,000000	2	
			63	righ	21,50000	1,369306	5	
				Total	21,50000	1,369306	5	
			64	righ	20,00000	,000000	2	
				Total	20,00000	,000000	2	
			66	left	22,00000	,000000	2	
				Total	22,00000	,000000	2	
			69	righ	21,00000	,000000	3	
				Total	21,00000	,000000	3	
			70	righ	14,00000	,000000	3	
				Total	14,00000	,000000	3	
			71	left	18,00000	,000000	3	
				righ	27,00000	2,190890	6	
				Total	24,00000	4,821825	9	
			72	righ	20,25000	2,464752	6	
				Total	20,25000	2,464752	6	
			73	left	26,00000	6,763875	9	
				righ	22,00000	,000000	2	
				Total	25,27273	6,262442	11	
			74	righ	19,80000	1,095445	5	
				Total	19,80000	1,095445	5	
			75	left	18,00000	,000000	3	
				righ	23,00000	,000000	3	
				Total	20,50000	2,738613	6	
			76	left	15,80000	7,941977	5	
				righ	30,00000	,000000	2	
				Total	19,85714	9,489970	7	
			77	left	24,88000	2,629068	5	
				Total	24,88000	2,629068	5	
			78	left	24,12500	1,807722	8	
				righ	26,60000	6,024948	5	
				Total	25,07692	3,946761	13	
			79	left	21,70000	,000000	3	
				righ	30,00000	,000000	3	
				Total	25,85000	4,546097	6	
			80	left	23,50000	6,480741	8	
				righ	13,92000	,985901	5	
				Total	19,81538	6,953877	13	
			81	righ	21,87500	6,266407	8	
				Total	21,87500	6,266407	8	
			82	left	23,96667	4,100915	9	
				righ	31,70000	,000000	2	
				Total	25,37273	4,820807	11	
			83	left	22,00000	,000000	3	
				righ	25,15000	5,312909	6	
				Total	24,10000	4,485811	9	
			84	left	26,10000	,000000	3	
				righ	24,97000	9,256715	10	
				Total	25,23077	8,031852	13	
			85	left	20,78824	3,671322	17	
				righ	26,02857	3,002417	14	
				Total	23,15484	4,257060	31	
			87	righ	25,00000	,000000	3	
				Total	25,00000	,000000	3	
			88	righ	17,40000	,000000	3	
				Total	17,40000	,000000	3	
			90	left	28,00000	,000000	2	
				Total	28,00000	,000000	2	
			96	left	30,00000	,000000	3	
				Total	30,00000	,000000	3	
			97	righ	20,40000	,000000	3	
				Total	20,40000	,000000	3	
			Total	left	22,65618	5,020593	89	
				righ	23,23168	5,683660	101	
				Total	22,96211	5,376919	190	
MDPost 30	,100	m	63	righ	9,50000	2,121320	2	
				Total	9,50000	2,121320	2	
			Total	righ	9,50000	2,121320	2	
				Total	9,50000	2,121320	2	
		Total	63	righ	9,50000	2,121320	2	
				Total	9,50000	2,121320	2	
			Total	righ	9,50000	2,121320	2	
				Total	9,50000	2,121320	2	
	,111	m	70	righ	6,00000	,000000	3	
				Total	6,00000	,000000	3	
			Total	righ	6,00000	,000000	3	
				Total	6,00000	,000000	3	
		Total	70	righ	6,00000	,000000	3	
				Total	6,00000	,000000	3	
			Total	righ	6,00000	,000000	3	
				Total	6,00000	,000000	3	
	,120	m	72	righ	3,00000	,000000	3	
				Total	3,00000	,000000	3	
			Total	righ	3,00000	,000000	3	
				Total	3,00000	,000000	3	
		Total	72	righ	3,00000	,000000	3	
				Total	3,00000	,000000	3	
			Total	righ	3,00000	,000000	3	
				Total	3,00000	,000000	3	
	,125	m	73	left	11,00000	,000000	3	
				Total	11,00000	,000000	3	
			Total	left	11,00000	,000000	3	
				Total	11,00000	,000000	3	
		Total	73	left	11,00000	,000000	3	
				Total	11,00000	,000000	3	
			Total	left	11,00000	,000000	3	
				Total	11,00000	,000000	3	
	,127	f	74	righ	6,00000	,000000	3	
				Total	6,00000	,000000	3	
			Total	righ	6,00000	,000000	3	
				Total	6,00000	,000000	3	
		Total	74	righ	6,00000	,000000	3	
				Total	6,00000	,000000	3	
			Total	righ	6,00000	,000000	3	
				Total	6,00000	,000000	3	
	,131	m	66	left	3,00000	,000000	2	
				Total	3,00000	,000000	2	
			Total	left	3,00000	,000000	2	
				Total	3,00000	,000000	2	
		Total	66	left	3,00000	,000000	2	
				Total	3,00000	,000000	2	
			Total	left	3,00000	,000000	2	
				Total	3,00000	,000000	2	
	,131	f	84	righ	13,00000	,000000	3	
				Total	13,00000	,000000	3	
			Total	righ	13,00000	,000000	3	
				Total	13,00000	,000000	3	
		Total	84	righ	13,00000	,000000	3	
				Total	13,00000	,000000	3	
			Total	righ	13,00000	,000000	3	
				Total	13,00000	,000000	3	
	,131	m	75	righ	14,00000	,000000	3	
				Total	14,00000	,000000	3	
			Total	righ	14,00000	,000000	3	
				Total	14,00000	,000000	3	
		Total	75	righ	14,00000	,000000	3	
				Total	14,00000	,000000	3	
			Total	righ	14,00000	,000000	3	
				Total	14,00000	,000000	3	
	,134	m	76	left	,00000	,000000	3	
				Total	,00000	,000000	3	
			Total	left	,00000	,000000	3	
				Total	,00000	,000000	3	
		Total	76	left	,00000	,000000	3	
				Total	,00000	,000000	3	
			Total	left	,00000	,000000	3	
				Total	,00000	,000000	3	
	,134	m	69	righ	16,00000	,000000	3	
				Total	16,00000	,000000	3	
			Total	righ	16,00000	,000000	3	
				Total	16,00000	,000000	3	
		Total	69	righ	16,00000	,000000	3	
				Total	16,00000	,000000	3	
			Total	righ	16,00000	,000000	3	
				Total	16,00000	,000000	3	
	,141	m	80	righ	3,00000	,000000	2	
				Total	3,00000	,000000	2	
			Total	righ	3,00000	,000000	2	
				Total	3,00000	,000000	2	
		Total	80	righ	3,00000	,000000	2	
				Total	3,00000	,000000	2	
			Total	righ	3,00000	,000000	2	
				Total	3,00000	,000000	2	
	,143	f	63	righ	8,00000	,000000	3	
				Total	8,00000	,000000	3	
			Total	righ	8,00000	,000000	3	
				Total	8,00000	,000000	3	
		Total	63	righ	8,00000	,000000	3	
				Total	8,00000	,000000	3	
			Total	righ	8,00000	,000000	3	
				Total	8,00000	,000000	3	
	,144	f	73	righ	13,00000	,000000	2	
				Total	13,00000	,000000	2	
			Total	righ	13,00000	,000000	2	
				Total	13,00000	,000000	2	
		Total	73	righ	13,00000	,000000	2	
				Total	13,00000	,000000	2	
			Total	righ	13,00000	,000000	2	
				Total	13,00000	,000000	2	
	,146	m	78	righ	6,00000	,000000	2	
				Total	6,00000	,000000	2	
			Total	righ	6,00000	,000000	2	
				Total	6,00000	,000000	2	
		Total	78	righ	6,00000	,000000	2	
				Total	6,00000	,000000	2	
			Total	righ	6,00000	,000000	2	
				Total	6,00000	,000000	2	
	,146	f	61	left	5,00000	,000000	3	
				Total	5,00000	,000000	3	
			Total	left	5,00000	,000000	3	
				Total	5,00000	,000000	3	
		Total	61	left	5,00000	,000000	3	
				Total	5,00000	,000000	3	
			Total	left	5,00000	,000000	3	
				Total	5,00000	,000000	3	
	,148	f	81	righ	13,00000	,000000	3	
				Total	13,00000	,000000	3	
			Total	righ	13,00000	,000000	3	
				Total	13,00000	,000000	3	
		Total	81	righ	13,00000	,000000	3	
				Total	13,00000	,000000	3	
			Total	righ	13,00000	,000000	3	
				Total	13,00000	,000000	3	
	,148	f	84	left	17,70000	,000000	3	
				Total	17,70000	,000000	3	
			Total	left	17,70000	,000000	3	
				Total	17,70000	,000000	3	
		Total	84	left	17,70000	,000000	3	
				Total	17,70000	,000000	3	
			Total	left	17,70000	,000000	3	
				Total	17,70000	,000000	3	
	,150	f	80	left	10,00000	,000000	3	
				Total	10,00000	,000000	3	
			Total	left	10,00000	,000000	3	
				Total	10,00000	,000000	3	
		Total	80	left	10,00000	,000000	3	
				Total	10,00000	,000000	3	
			Total	left	10,00000	,000000	3	
				Total	10,00000	,000000	3	
	,152	m	73	left	15,40000	,000000	3	
				Total	15,40000	,000000	3	
			Total	left	15,40000	,000000	3	
				Total	15,40000	,000000	3	
		Total	73	left	15,40000	,000000	3	
				Total	15,40000	,000000	3	
			Total	left	15,40000	,000000	3	
				Total	15,40000	,000000	3	
	,152	m	72	righ	12,40000	,000000	3	
				Total	12,40000	,000000	3	
			Total	righ	12,40000	,000000	3	
				Total	12,40000	,000000	3	
		Total	72	righ	12,40000	,000000	3	
				Total	12,40000	,000000	3	
			Total	righ	12,40000	,000000	3	
				Total	12,40000	,000000	3	
	,156	m	85	left	8,00000	,000000	3	
				Total	8,00000	,000000	3	
			Total	left	8,00000	,000000	3	
				Total	8,00000	,000000	3	
		Total	85	left	8,00000	,000000	3	
				Total	8,00000	,000000	3	
			Total	left	8,00000	,000000	3	
				Total	8,00000	,000000	3	
	,159	m	88	righ	5,00000	,000000	3	
				Total	5,00000	,000000	3	
			Total	righ	5,00000	,000000	3	
				Total	5,00000	,000000	3	
		Total	88	righ	5,00000	,000000	3	
				Total	5,00000	,000000	3	
			Total	righ	5,00000	,000000	3	
				Total	5,00000	,000000	3	
	,159	m	77	left	17,60000	,000000	3	
				Total	17,60000	,000000	3	
			Total	left	17,60000	,000000	3	
				Total	17,60000	,000000	3	
		Total	77	left	17,60000	,000000	3	
				Total	17,60000	,000000	3	
			Total	left	17,60000	,000000	3	
				Total	17,60000	,000000	3	
	,159	f	79	righ	16,00000	,000000	3	
				Total	16,00000	,000000	3	
			Total	righ	16,00000	,000000	3	
				Total	16,00000	,000000	3	
		Total	79	righ	16,00000	,000000	3	
				Total	16,00000	,000000	3	
			Total	righ	16,00000	,000000	3	
				Total	16,00000	,000000	3	
	,160	f	80	righ	5,00000	,000000	3	
				Total	5,00000	,000000	3	
			Total	righ	5,00000	,000000	3	
				Total	5,00000	,000000	3	
		Total	80	righ	5,00000	,000000	3	
				Total	5,00000	,000000	3	
			Total	righ	5,00000	,000000	3	
				Total	5,00000	,000000	3	
	,163	m	75	left	14,00000	,000000	3	
				Total	14,00000	,000000	3	
			Total	left	14,00000	,000000	3	
				Total	14,00000	,000000	3	
		Total	75	left	14,00000	,000000	3	
				Total	14,00000	,000000	3	
			Total	left	14,00000	,000000	3	
				Total	14,00000	,000000	3	
	,167	m	73	left	9,33333	2,886751	3	
				Total	9,33333	2,886751	3	
			Total	left	9,33333	2,886751	3	
				Total	9,33333	2,886751	3	
		Total	73	left	9,33333	2,886751	3	
				Total	9,33333	2,886751	3	
			Total	left	9,33333	2,886751	3	
				Total	9,33333	2,886751	3	
	,168	f	60	left	,00000	,000000	3	
				Total	,00000	,000000	3	
			Total	left	,00000	,000000	3	
				Total	,00000	,000000	3	
		Total	60	left	,00000	,000000	3	
				Total	,00000	,000000	3	
			Total	left	,00000	,000000	3	
				Total	,00000	,000000	3	
	,170	m	62	righ	12,40000	,000000	2	
				Total	12,40000	,000000	2	
			Total	righ	12,40000	,000000	2	
				Total	12,40000	,000000	2	
		Total	62	righ	12,40000	,000000	2	
				Total	12,40000	,000000	2	
			Total	righ	12,40000	,000000	2	
				Total	12,40000	,000000	2	
	,170	f	84	righ	8,80000	,000000	2	
				Total	8,80000	,000000	2	
			Total	righ	8,80000	,000000	2	
				Total	8,80000	,000000	2	
		Total	84	righ	8,80000	,000000	2	
				Total	8,80000	,000000	2	
			Total	righ	8,80000	,000000	2	
				Total	8,80000	,000000	2	
	,171	f	78	left	18,80000	,000000	3	
				Total	18,80000	,000000	3	
			Total	left	18,80000	,000000	3	
				Total	18,80000	,000000	3	
		Total	78	left	18,80000	,000000	3	
				Total	18,80000	,000000	3	
			Total	left	18,80000	,000000	3	
				Total	18,80000	,000000	3	
	,172	f	84	righ	3,00000	,000000	3	
				Total	3,00000	,000000	3	
			Total	righ	3,00000	,000000	3	
				Total	3,00000	,000000	3	
		Total	84	righ	3,00000	,000000	3	
				Total	3,00000	,000000	3	
			Total	righ	3,00000	,000000	3	
				Total	3,00000	,000000	3	
	,173	m	76	righ	12,00000	,000000	2	
				Total	12,00000	,000000	2	
			Total	righ	12,00000	,000000	2	
				Total	12,00000	,000000	2	
		Total	76	righ	12,00000	,000000	2	
				Total	12,00000	,000000	2	
			Total	righ	12,00000	,000000	2	
				Total	12,00000	,000000	2	
	,174	m	77	left	10,00000	5,656854	2	
				Total	10,00000	5,656854	2	
			Total	left	10,00000	5,656854	2	
				Total	10,00000	5,656854	2	
		Total	77	left	10,00000	5,656854	2	
				Total	10,00000	5,656854	2	
			Total	left	10,00000	5,656854	2	
				Total	10,00000	5,656854	2	
	,175	m	76	left	8,00000	,000000	2	
				Total	8,00000	,000000	2	
			Total	left	8,00000	,000000	2	
				Total	8,00000	,000000	2	
		Total	76	left	8,00000	,000000	2	
				Total	8,00000	,000000	2	
			Total	left	8,00000	,000000	2	
				Total	8,00000	,000000	2	
	,176	m	85	left	13,70000	,000000	3	
				Total	13,70000	,000000	3	
			Total	left	13,70000	,000000	3	
				Total	13,70000	,000000	3	
		Total	85	left	13,70000	,000000	3	
				Total	13,70000	,000000	3	
			Total	left	13,70000	,000000	3	
				Total	13,70000	,000000	3	
	,177	m	85	left	9,00000	5,196152	3	
				Total	9,00000	5,196152	3	
			Total	left	9,00000	5,196152	3	
				Total	9,00000	5,196152	3	
		Total	85	left	9,00000	5,196152	3	
				Total	9,00000	5,196152	3	
			Total	left	9,00000	5,196152	3	
				Total	9,00000	5,196152	3	
	,180	f	64	righ	8,50000	3,535534	2	
				Total	8,50000	3,535534	2	
			Total	righ	8,50000	3,535534	2	
				Total	8,50000	3,535534	2	
		Total	64	righ	8,50000	3,535534	2	
				Total	8,50000	3,535534	2	
			Total	righ	8,50000	3,535534	2	
				Total	8,50000	3,535534	2	
	,181	f	80	left	17,00000	,000000	2	
				Total	17,00000	,000000	2	
			Total	left	17,00000	,000000	2	
				Total	17,00000	,000000	2	
		Total	80	left	17,00000	,000000	2	
				Total	17,00000	,000000	2	
			Total	left	17,00000	,000000	2	
				Total	17,00000	,000000	2	
	,183	f	85	righ	16,70000	,000000	3	
				Total	16,70000	,000000	3	
			Total	righ	16,70000	,000000	3	
				Total	16,70000	,000000	3	
		Total	85	righ	16,70000	,000000	3	
				Total	16,70000	,000000	3	
			Total	righ	16,70000	,000000	3	
				Total	16,70000	,000000	3	
	,185	m	71	righ	15,00000	,000000	3	
				Total	15,00000	,000000	3	
			Total	righ	15,00000	,000000	3	
				Total	15,00000	,000000	3	
		Total	71	righ	15,00000	,000000	3	
				Total	15,00000	,000000	3	
			Total	righ	15,00000	,000000	3	
				Total	15,00000	,000000	3	
	,188	m	78	righ	,00000	,000000	3	
				Total	,00000	,000000	3	
			Total	righ	,00000	,000000	3	
				Total	,00000	,000000	3	
		Total	78	righ	,00000	,000000	3	
				Total	,00000	,000000	3	
			Total	righ	,00000	,000000	3	
				Total	,00000	,000000	3	
	,188	m	81	righ	13,00000	,000000	3	
				Total	13,00000	,000000	3	
			Total	righ	13,00000	,000000	3	
				Total	13,00000	,000000	3	
		Total	81	righ	13,00000	,000000	3	
				Total	13,00000	,000000	3	
			Total	righ	13,00000	,000000	3	
				Total	13,00000	,000000	3	
	,189	m	85	righ	7,80000	,000000	3	
				Total	7,80000	,000000	3	
			Total	righ	7,80000	,000000	3	
				Total	7,80000	,000000	3	
		Total	85	righ	7,80000	,000000	3	
				Total	7,80000	,000000	3	
			Total	righ	7,80000	,000000	3	
				Total	7,80000	,000000	3	
	,190	f	81	righ	12,10000	,000000	2	
				Total	12,10000	,000000	2	
			Total	righ	12,10000	,000000	2	
				Total	12,10000	,000000	2	
		Total	81	righ	12,10000	,000000	2	
				Total	12,10000	,000000	2	
			Total	righ	12,10000	,000000	2	
				Total	12,10000	,000000	2	
	,191	f	79	left	15,00000	,000000	3	
				Total	15,00000	,000000	3	
			Total	left	15,00000	,000000	3	
				Total	15,00000	,000000	3	
		Total	79	left	15,00000	,000000	3	
				Total	15,00000	,000000	3	
			Total	left	15,00000	,000000	3	
				Total	15,00000	,000000	3	
	,192	f	78	left	12,00000	,000000	2	
				Total	12,00000	,000000	2	
			Total	left	12,00000	,000000	2	
				Total	12,00000	,000000	2	
		Total	78	left	12,00000	,000000	2	
				Total	12,00000	,000000	2	
			Total	left	12,00000	,000000	2	
				Total	12,00000	,000000	2	
	,192	f	71	left	4,00000	,000000	3	
				Total	4,00000	,000000	3	
			Total	left	4,00000	,000000	3	
				Total	4,00000	,000000	3	
		Total	71	left	4,00000	,000000	3	
				Total	4,00000	,000000	3	
			Total	left	4,00000	,000000	3	
				Total	4,00000	,000000	3	
	,194	m	85	left	11,80000	,000000	2	
				Total	11,80000	,000000	2	
			Total	left	11,80000	,000000	2	
				Total	11,80000	,000000	2	
		Total	85	left	11,80000	,000000	2	
				Total	11,80000	,000000	2	
			Total	left	11,80000	,000000	2	
				Total	11,80000	,000000	2	
	,198	m	84	righ	,00000	,000000	2	
				Total	,00000	,000000	2	
			Total	righ	,00000	,000000	2	
				Total	,00000	,000000	2	
		Total	84	righ	,00000	,000000	2	
				Total	,00000	,000000	2	
			Total	righ	,00000	,000000	2	
				Total	,00000	,000000	2	
	,198	m	90	left	10,00000	,000000	2	
				Total	10,00000	,000000	2	
			Total	left	10,00000	,000000	2	
				Total	10,00000	,000000	2	
		Total	90	left	10,00000	,000000	2	
				Total	10,00000	,000000	2	
			Total	left	10,00000	,000000	2	
				Total	10,00000	,000000	2	
	,200	m	80	left	5,00000	,000000	3	
				Total	5,00000	,000000	3	
			Total	left	5,00000	,000000	3	
				Total	5,00000	,000000	3	
		Total	80	left	5,00000	,000000	3	
				Total	5,00000	,000000	3	
			Total	left	5,00000	,000000	3	
				Total	5,00000	,000000	3	
	,200	m	78	left	14,00000	,000000	3	
				Total	14,00000	,000000	3	
			Total	left	14,00000	,000000	3	
				Total	14,00000	,000000	3	
		Total	78	left	14,00000	,000000	3	
				Total	14,00000	,000000	3	
			Total	left	14,00000	,000000	3	
				Total	14,00000	,000000	3	
	,201	m	96	left	15,00000	,000000	3	
				Total	15,00000	,000000	3	
			Total	left	15,00000	,000000	3	
				Total	15,00000	,000000	3	
		Total	96	left	15,00000	,000000	3	
				Total	15,00000	,000000	3	
			Total	left	15,00000	,000000	3	
				Total	15,00000	,000000	3	
	,207	m	83	left	11,40000	,000000	3	
				Total	11,40000	,000000	3	
			Total	left	11,40000	,000000	3	
				Total	11,40000	,000000	3	
		Total	83	left	11,40000	,000000	3	
				Total	11,40000	,000000	3	
			Total	left	11,40000	,000000	3	
				Total	11,40000	,000000	3	
	,208	f	83	righ	7,90000	,000000	3	
				Total	7,90000	,000000	3	
			Total	righ	7,90000	,000000	3	
				Total	7,90000	,000000	3	
		Total	83	righ	7,90000	,000000	3	
				Total	7,90000	,000000	3	
			Total	righ	7,90000	,000000	3	
				Total	7,90000	,000000	3	
	,210	m	82	left	6,00000	,000000	3	
				Total	6,00000	,000000	3	
			Total	left	6,00000	,000000	3	
				Total	6,00000	,000000	3	
		Total	82	left	6,00000	,000000	3	
				Total	6,00000	,000000	3	
			Total	left	6,00000	,000000	3	
				Total	6,00000	,000000	3	
	,211	m	85	left	10,70000	,000000	3	
				Total	10,70000	,000000	3	
			Total	left	10,70000	,000000	3	
				Total	10,70000	,000000	3	
		Total	85	left	10,70000	,000000	3	
				Total	10,70000	,000000	3	
			Total	left	10,70000	,000000	3	
				Total	10,70000	,000000	3	
	,213	m	82	left	14,00000	,000000	3	
				Total	14,00000	,000000	3	
			Total	left	14,00000	,000000	3	
				Total	14,00000	,000000	3	
		Total	82	left	14,00000	,000000	3	
				Total	14,00000	,000000	3	
			Total	left	14,00000	,000000	3	
				Total	14,00000	,000000	3	
	,213	m	82	righ	14,50000	,000000	2	
				Total	14,50000	,000000	2	
			Total	righ	14,50000	,000000	2	
				Total	14,50000	,000000	2	
		Total	82	righ	14,50000	,000000	2	
				Total	14,50000	,000000	2	
			Total	righ	14,50000	,000000	2	
				Total	14,50000	,000000	2	
	,214	m	85	righ	20,00000	,000000	3	
				Total	20,00000	,000000	3	
			Total	righ	20,00000	,000000	3	
				Total	20,00000	,000000	3	
		Total	85	righ	20,00000	,000000	3	
				Total	20,00000	,000000	3	
			Total	righ	20,00000	,000000	3	
				Total	20,00000	,000000	3	
	,219	f	71	righ	10,00000	,000000	3	
				Total	10,00000	,000000	3	
			Total	righ	10,00000	,000000	3	
				Total	10,00000	,000000	3	
		Total	71	righ	10,00000	,000000	3	
				Total	10,00000	,000000	3	
			Total	righ	10,00000	,000000	3	
				Total	10,00000	,000000	3	
	,221	f	74	righ	9,50000	2,121320	2	
				Total	9,50000	2,121320	2	
			Total	righ	9,50000	2,121320	2	
				Total	9,50000	2,121320	2	
		Total	74	righ	9,50000	2,121320	2	
				Total	9,50000	2,121320	2	
			Total	righ	9,50000	2,121320	2	
				Total	9,50000	2,121320	2	
	,224	m	85	righ	15,00000	,000000	2	
				Total	15,00000	,000000	2	
			Total	righ	15,00000	,000000	2	
				Total	15,00000	,000000	2	
		Total	85	righ	15,00000	,000000	2	
				Total	15,00000	,000000	2	
			Total	righ	15,00000	,000000	2	
				Total	15,00000	,000000	2	
	,224	f	97	righ	12,50000	,000000	3	
				Total	12,50000	,000000	3	
			Total	righ	12,50000	,000000	3	
				Total	12,50000	,000000	3	
		Total	97	righ	12,50000	,000000	3	
				Total	12,50000	,000000	3	
			Total	righ	12,50000	,000000	3	
				Total	12,50000	,000000	3	
	,226	f	82	left	17,20000	,000000	3	
				Total	17,20000	,000000	3	
			Total	left	17,20000	,000000	3	
				Total	17,20000	,000000	3	
		Total	82	left	17,20000	,000000	3	
				Total	17,20000	,000000	3	
			Total	left	17,20000	,000000	3	
				Total	17,20000	,000000	3	
	,226	f	83	righ	22,70000	,000000	3	
				Total	22,70000	,000000	3	
			Total	righ	22,70000	,000000	3	
				Total	22,70000	,000000	3	
		Total	83	righ	22,70000	,000000	3	
				Total	22,70000	,000000	3	
			Total	righ	22,70000	,000000	3	
				Total	22,70000	,000000	3	
	,228	m	87	righ	8,00000	,000000	3	
				Total	8,00000	,000000	3	
			Total	righ	8,00000	,000000	3	
				Total	8,00000	,000000	3	
		Total	87	righ	8,00000	,000000	3	
				Total	8,00000	,000000	3	
			Total	righ	8,00000	,000000	3	
				Total	8,00000	,000000	3	
	,254	m	85	left	9,00000	,000000	3	
				Total	9,00000	,000000	3	
			Total	left	9,00000	,000000	3	
				Total	9,00000	,000000	3	
		Total	85	left	9,00000	,000000	3	
				Total	9,00000	,000000	3	
			Total	left	9,00000	,000000	3	
				Total	9,00000	,000000	3	
	,265	m	85	righ	21,00000	,000000	3	
				Total	21,00000	,000000	3	
			Total	righ	21,00000	,000000	3	
				Total	21,00000	,000000	3	
		Total	85	righ	21,00000	,000000	3	
				Total	21,00000	,000000	3	
			Total	righ	21,00000	,000000	3	
				Total	21,00000	,000000	3	
	Total	f	60	left	,00000	,000000	3	
				Total	,00000	,000000	3	
			61	left	5,00000	,000000	3	
				Total	5,00000	,000000	3	
			63	righ	8,00000	,000000	3	
				Total	8,00000	,000000	3	
			64	righ	8,50000	3,535534	2	
				Total	8,50000	3,535534	2	
			71	left	4,00000	,000000	3	
				righ	10,00000	,000000	3	
				Total	7,00000	3,286335	6	
			73	righ	13,00000	,000000	2	
				Total	13,00000	,000000	2	
			74	righ	7,40000	2,190890	5	
				Total	7,40000	2,190890	5	
			78	left	16,08000	3,724513	5	
				Total	16,08000	3,724513	5	
			79	left	15,00000	,000000	3	
				righ	16,00000	,000000	3	
				Total	15,50000	,547723	6	
			80	left	12,80000	3,834058	5	
				righ	5,00000	,000000	3	
				Total	9,87500	4,969550	8	
			81	righ	12,64000	,492950	5	
				Total	12,64000	,492950	5	
			82	left	17,20000	,000000	3	
				Total	17,20000	,000000	3	
			83	righ	15,30000	8,106294	6	
				Total	15,30000	8,106294	6	
			84	left	17,70000	,000000	3	
				righ	8,20000	4,643890	8	
				Total	10,79091	5,898043	11	
			85	righ	16,70000	,000000	3	
				Total	16,70000	,000000	3	
			97	righ	12,50000	,000000	3	
				Total	12,50000	,000000	3	
			Total	left	11,46786	6,581065	28	
				righ	10,98261	4,948190	46	
				Total	11,16622	5,582857	74	
		m	62	righ	12,40000	,000000	2	
				Total	12,40000	,000000	2	
			63	righ	9,50000	2,121320	2	
				Total	9,50000	2,121320	2	
			66	left	3,00000	,000000	2	
				Total	3,00000	,000000	2	
			69	righ	16,00000	,000000	3	
				Total	16,00000	,000000	3	
			70	righ	6,00000	,000000	3	
				Total	6,00000	,000000	3	
			71	righ	15,00000	,000000	3	
				Total	15,00000	,000000	3	
			72	righ	7,70000	5,148592	6	
				Total	7,70000	5,148592	6	
			73	left	11,91111	3,074266	9	
				Total	11,91111	3,074266	9	
			75	left	14,00000	,000000	3	
				righ	14,00000	,000000	3	
				Total	14,00000	,000000	6	
			76	left	3,20000	4,381780	5	
				righ	12,00000	,000000	2	
				Total	5,71429	5,589105	7	
			77	left	14,56000	5,032693	5	
				Total	14,56000	5,032693	5	
			78	left	14,00000	,000000	3	
				righ	2,40000	3,286335	5	
				Total	6,75000	6,497252	8	
			80	left	5,00000	,000000	3	
				righ	3,00000	,000000	2	
				Total	4,20000	1,095445	5	
			81	righ	13,00000	,000000	3	
				Total	13,00000	,000000	3	
			82	left	10,00000	4,381780	6	
				righ	14,50000	,000000	2	
				Total	11,12500	4,248949	8	
			83	left	11,40000	,000000	3	
				Total	11,40000	,000000	3	
			84	righ	,00000	,000000	2	
				Total	,00000	,000000	2	
			85	left	10,28235	2,734784	17	
				righ	16,03636	5,715815	11	
				Total	12,54286	4,972102	28	
			87	righ	8,00000	,000000	3	
				Total	8,00000	,000000	3	
			88	righ	5,00000	,000000	3	
				Total	5,00000	,000000	3	
			90	left	10,00000	,000000	2	
				Total	10,00000	,000000	2	
			96	left	15,00000	,000000	3	
				Total	15,00000	,000000	3	
			Total	left	10,40984	4,431730	61	
				righ	10,33455	6,045605	55	
				Total	10,37414	5,235533	116	
		Total	60	left	,00000	,000000	3	
				Total	,00000	,000000	3	
			61	left	5,00000	,000000	3	
				Total	5,00000	,000000	3	
			62	righ	12,40000	,000000	2	
				Total	12,40000	,000000	2	
			63	righ	8,60000	1,341641	5	
				Total	8,60000	1,341641	5	
			64	righ	8,50000	3,535534	2	
				Total	8,50000	3,535534	2	
			66	left	3,00000	,000000	2	
				Total	3,00000	,000000	2	
			69	righ	16,00000	,000000	3	
				Total	16,00000	,000000	3	
			70	righ	6,00000	,000000	3	
				Total	6,00000	,000000	3	
			71	left	4,00000	,000000	3	
				righ	12,50000	2,738613	6	
				Total	9,66667	4,769696	9	
			72	righ	7,70000	5,148592	6	
				Total	7,70000	5,148592	6	
			73	left	11,91111	3,074266	9	
				righ	13,00000	,000000	2	
				Total	12,10909	2,784764	11	
			74	righ	7,40000	2,190890	5	
				Total	7,40000	2,190890	5	
			75	left	14,00000	,000000	3	
				righ	14,00000	,000000	3	
				Total	14,00000	,000000	6	
			76	left	3,20000	4,381780	5	
				righ	12,00000	,000000	2	
				Total	5,71429	5,589105	7	
			77	left	14,56000	5,032693	5	
				Total	14,56000	5,032693	5	
			78	left	15,30000	3,014252	8	
				righ	2,40000	3,286335	5	
				Total	10,33846	7,181172	13	
			79	left	15,00000	,000000	3	
				righ	16,00000	,000000	3	
				Total	15,50000	,547723	6	
			80	left	9,87500	4,969550	8	
				righ	4,20000	1,095445	5	
				Total	7,69231	4,802510	13	
			81	righ	12,77500	,416619	8	
				Total	12,77500	,416619	8	
			82	left	12,40000	4,995998	9	
				righ	14,50000	,000000	2	
				Total	12,78182	4,548586	11	
			83	left	11,40000	,000000	3	
				righ	15,30000	8,106294	6	
				Total	14,00000	6,698694	9	
			84	left	17,70000	,000000	3	
				righ	6,56000	5,359768	10	
				Total	9,13077	6,738742	13	
			85	left	10,28235	2,734784	17	
				righ	16,17857	5,021060	14	
				Total	12,94516	4,879606	31	
			87	righ	8,00000	,000000	3	
				Total	8,00000	,000000	3	
			88	righ	5,00000	,000000	3	
				Total	5,00000	,000000	3	
			90	left	10,00000	,000000	2	
				Total	10,00000	,000000	2	
			96	left	15,00000	,000000	3	
				Total	15,00000	,000000	3	
			97	righ	12,50000	,000000	3	
				Total	12,50000	,000000	3	
			Total	left	10,74270	5,188797	89	
				righ	10,62970	5,555170	101	
				Total	10,68263	5,372807	190	
MDPost 90	,100	m	63	righ	5,75000	,353553	2	
				Total	5,75000	,353553	2	
			Total	righ	5,75000	,353553	2	
				Total	5,75000	,353553	2	
		Total	63	righ	5,75000	,353553	2	
				Total	5,75000	,353553	2	
			Total	righ	5,75000	,353553	2	
				Total	5,75000	,353553	2	
	,111	m	70	righ	5,23333	3,043572	3	
				Total	5,23333	3,043572	3	
			Total	righ	5,23333	3,043572	3	
				Total	5,23333	3,043572	3	
		Total	70	righ	5,23333	3,043572	3	
				Total	5,23333	3,043572	3	
			Total	righ	5,23333	3,043572	3	
				Total	5,23333	3,043572	3	
	,120	m	72	righ	1,00000	,000000	3	
				Total	1,00000	,000000	3	
			Total	righ	1,00000	,000000	3	
				Total	1,00000	,000000	3	
		Total	72	righ	1,00000	,000000	3	
				Total	1,00000	,000000	3	
			Total	righ	1,00000	,000000	3	
				Total	1,00000	,000000	3	
	,125	m	73	left	4,33333	2,886751	3	
				Total	4,33333	2,886751	3	
			Total	left	4,33333	2,886751	3	
				Total	4,33333	2,886751	3	
		Total	73	left	4,33333	2,886751	3	
				Total	4,33333	2,886751	3	
			Total	left	4,33333	2,886751	3	
				Total	4,33333	2,886751	3	
	,127	f	74	righ	2,00000	1,000000	3	
				Total	2,00000	1,000000	3	
			Total	righ	2,00000	1,000000	3	
				Total	2,00000	1,000000	3	
		Total	74	righ	2,00000	1,000000	3	
				Total	2,00000	1,000000	3	
			Total	righ	2,00000	1,000000	3	
				Total	2,00000	1,000000	3	
	,131	m	66	left	,50000	,707107	2	
				Total	,50000	,707107	2	
			Total	left	,50000	,707107	2	
				Total	,50000	,707107	2	
		Total	66	left	,50000	,707107	2	
				Total	,50000	,707107	2	
			Total	left	,50000	,707107	2	
				Total	,50000	,707107	2	
	,131	f	84	righ	3,00000	,000000	3	
				Total	3,00000	,000000	3	
			Total	righ	3,00000	,000000	3	
				Total	3,00000	,000000	3	
		Total	84	righ	3,00000	,000000	3	
				Total	3,00000	,000000	3	
			Total	righ	3,00000	,000000	3	
				Total	3,00000	,000000	3	
	,131	m	75	righ	6,00000	2,645751	3	
				Total	6,00000	2,645751	3	
			Total	righ	6,00000	2,645751	3	
				Total	6,00000	2,645751	3	
		Total	75	righ	6,00000	2,645751	3	
				Total	6,00000	2,645751	3	
			Total	righ	6,00000	2,645751	3	
				Total	6,00000	2,645751	3	
	,134	m	76	left	2,00000	3,464102	3	
				Total	2,00000	3,464102	3	
			Total	left	2,00000	3,464102	3	
				Total	2,00000	3,464102	3	
		Total	76	left	2,00000	3,464102	3	
				Total	2,00000	3,464102	3	
			Total	left	2,00000	3,464102	3	
				Total	2,00000	3,464102	3	
	,134	m	69	righ	5,83333	,288675	3	
				Total	5,83333	,288675	3	
			Total	righ	5,83333	,288675	3	
				Total	5,83333	,288675	3	
		Total	69	righ	5,83333	,288675	3	
				Total	5,83333	,288675	3	
			Total	righ	5,83333	,288675	3	
				Total	5,83333	,288675	3	
	,141	m	80	righ	3,50000	3,535534	2	
				Total	3,50000	3,535534	2	
			Total	righ	3,50000	3,535534	2	
				Total	3,50000	3,535534	2	
		Total	80	righ	3,50000	3,535534	2	
				Total	3,50000	3,535534	2	
			Total	righ	3,50000	3,535534	2	
				Total	3,50000	3,535534	2	
	,143	f	63	righ	,00000	,000000	3	
				Total	,00000	,000000	3	
			Total	righ	,00000	,000000	3	
				Total	,00000	,000000	3	
		Total	63	righ	,00000	,000000	3	
				Total	,00000	,000000	3	
			Total	righ	,00000	,000000	3	
				Total	,00000	,000000	3	
	,144	f	73	righ	8,35000	,494975	2	
				Total	8,35000	,494975	2	
			Total	righ	8,35000	,494975	2	
				Total	8,35000	,494975	2	
		Total	73	righ	8,35000	,494975	2	
				Total	8,35000	,494975	2	
			Total	righ	8,35000	,494975	2	
				Total	8,35000	,494975	2	
	,146	m	78	righ	5,15000	4,454773	2	
				Total	5,15000	4,454773	2	
			Total	righ	5,15000	4,454773	2	
				Total	5,15000	4,454773	2	
		Total	78	righ	5,15000	4,454773	2	
				Total	5,15000	4,454773	2	
			Total	righ	5,15000	4,454773	2	
				Total	5,15000	4,454773	2	
	,146	f	61	left	1,00000	,000000	3	
				Total	1,00000	,000000	3	
			Total	left	1,00000	,000000	3	
				Total	1,00000	,000000	3	
		Total	61	left	1,00000	,000000	3	
				Total	1,00000	,000000	3	
			Total	left	1,00000	,000000	3	
				Total	1,00000	,000000	3	
	,148	f	81	righ	4,33333	1,527525	3	
				Total	4,33333	1,527525	3	
			Total	righ	4,33333	1,527525	3	
				Total	4,33333	1,527525	3	
		Total	81	righ	4,33333	1,527525	3	
				Total	4,33333	1,527525	3	
			Total	righ	4,33333	1,527525	3	
				Total	4,33333	1,527525	3	
	,148	f	84	left	8,70000	,000000	3	
				Total	8,70000	,000000	3	
			Total	left	8,70000	,000000	3	
				Total	8,70000	,000000	3	
		Total	84	left	8,70000	,000000	3	
				Total	8,70000	,000000	3	
			Total	left	8,70000	,000000	3	
				Total	8,70000	,000000	3	
	,150	f	80	left	1,66667	1,154701	3	
				Total	1,66667	1,154701	3	
			Total	left	1,66667	1,154701	3	
				Total	1,66667	1,154701	3	
		Total	80	left	1,66667	1,154701	3	
				Total	1,66667	1,154701	3	
			Total	left	1,66667	1,154701	3	
				Total	1,66667	1,154701	3	
	,152	m	73	left	5,00000	,000000	3	
				Total	5,00000	,000000	3	
			Total	left	5,00000	,000000	3	
				Total	5,00000	,000000	3	
		Total	73	left	5,00000	,000000	3	
				Total	5,00000	,000000	3	
			Total	left	5,00000	,000000	3	
				Total	5,00000	,000000	3	
	,152	m	72	righ	3,00000	,000000	3	
				Total	3,00000	,000000	3	
			Total	righ	3,00000	,000000	3	
				Total	3,00000	,000000	3	
		Total	72	righ	3,00000	,000000	3	
				Total	3,00000	,000000	3	
			Total	righ	3,00000	,000000	3	
				Total	3,00000	,000000	3	
	,156	m	85	left	4,00000	1,732051	3	
				Total	4,00000	1,732051	3	
			Total	left	4,00000	1,732051	3	
				Total	4,00000	1,732051	3	
		Total	85	left	4,00000	1,732051	3	
				Total	4,00000	1,732051	3	
			Total	left	4,00000	1,732051	3	
				Total	4,00000	1,732051	3	
	,159	m	88	righ	1,66667	1,154701	3	
				Total	1,66667	1,154701	3	
			Total	righ	1,66667	1,154701	3	
				Total	1,66667	1,154701	3	
		Total	88	righ	1,66667	1,154701	3	
				Total	1,66667	1,154701	3	
			Total	righ	1,66667	1,154701	3	
				Total	1,66667	1,154701	3	
	,159	m	77	left	8,66667	2,081666	3	
				Total	8,66667	2,081666	3	
			Total	left	8,66667	2,081666	3	
				Total	8,66667	2,081666	3	
		Total	77	left	8,66667	2,081666	3	
				Total	8,66667	2,081666	3	
			Total	left	8,66667	2,081666	3	
				Total	8,66667	2,081666	3	
	,159	f	79	righ	5,00000	3,605551	3	
				Total	5,00000	3,605551	3	
			Total	righ	5,00000	3,605551	3	
				Total	5,00000	3,605551	3	
		Total	79	righ	5,00000	3,605551	3	
				Total	5,00000	3,605551	3	
			Total	righ	5,00000	3,605551	3	
				Total	5,00000	3,605551	3	
	,160	f	80	righ	2,66667	,577350	3	
				Total	2,66667	,577350	3	
			Total	righ	2,66667	,577350	3	
				Total	2,66667	,577350	3	
		Total	80	righ	2,66667	,577350	3	
				Total	2,66667	,577350	3	
			Total	righ	2,66667	,577350	3	
				Total	2,66667	,577350	3	
	,163	m	75	left	5,00000	3,605551	3	
				Total	5,00000	3,605551	3	
			Total	left	5,00000	3,605551	3	
				Total	5,00000	3,605551	3	
		Total	75	left	5,00000	3,605551	3	
				Total	5,00000	3,605551	3	
			Total	left	5,00000	3,605551	3	
				Total	5,00000	3,605551	3	
	,167	m	73	left	5,86667	,115470	3	
				Total	5,86667	,115470	3	
			Total	left	5,86667	,115470	3	
				Total	5,86667	,115470	3	
		Total	73	left	5,86667	,115470	3	
				Total	5,86667	,115470	3	
			Total	left	5,86667	,115470	3	
				Total	5,86667	,115470	3	
	,168	f	60	left	,33333	,577350	3	
				Total	,33333	,577350	3	
			Total	left	,33333	,577350	3	
				Total	,33333	,577350	3	
		Total	60	left	,33333	,577350	3	
				Total	,33333	,577350	3	
			Total	left	,33333	,577350	3	
				Total	,33333	,577350	3	
	,170	m	62	righ	6,00000	2,828427	2	
				Total	6,00000	2,828427	2	
			Total	righ	6,00000	2,828427	2	
				Total	6,00000	2,828427	2	
		Total	62	righ	6,00000	2,828427	2	
				Total	6,00000	2,828427	2	
			Total	righ	6,00000	2,828427	2	
				Total	6,00000	2,828427	2	
	,170	f	84	righ	5,00000	1,414214	2	
				Total	5,00000	1,414214	2	
			Total	righ	5,00000	1,414214	2	
				Total	5,00000	1,414214	2	
		Total	84	righ	5,00000	1,414214	2	
				Total	5,00000	1,414214	2	
			Total	righ	5,00000	1,414214	2	
				Total	5,00000	1,414214	2	
	,171	f	78	left	7,53333	1,327906	3	
				Total	7,53333	1,327906	3	
			Total	left	7,53333	1,327906	3	
				Total	7,53333	1,327906	3	
		Total	78	left	7,53333	1,327906	3	
				Total	7,53333	1,327906	3	
			Total	left	7,53333	1,327906	3	
				Total	7,53333	1,327906	3	
	,172	f	84	righ	1,00000	,000000	3	
				Total	1,00000	,000000	3	
			Total	righ	1,00000	,000000	3	
				Total	1,00000	,000000	3	
		Total	84	righ	1,00000	,000000	3	
				Total	1,00000	,000000	3	
			Total	righ	1,00000	,000000	3	
				Total	1,00000	,000000	3	
	,173	m	76	righ	6,50000	2,121320	2	
				Total	6,50000	2,121320	2	
			Total	righ	6,50000	2,121320	2	
				Total	6,50000	2,121320	2	
		Total	76	righ	6,50000	2,121320	2	
				Total	6,50000	2,121320	2	
			Total	righ	6,50000	2,121320	2	
				Total	6,50000	2,121320	2	
	,174	m	77	left	7,50000	6,363961	2	
				Total	7,50000	6,363961	2	
			Total	left	7,50000	6,363961	2	
				Total	7,50000	6,363961	2	
		Total	77	left	7,50000	6,363961	2	
				Total	7,50000	6,363961	2	
			Total	left	7,50000	6,363961	2	
				Total	7,50000	6,363961	2	
	,175	m	76	left	3,00000	4,242641	2	
				Total	3,00000	4,242641	2	
			Total	left	3,00000	4,242641	2	
				Total	3,00000	4,242641	2	
		Total	76	left	3,00000	4,242641	2	
				Total	3,00000	4,242641	2	
			Total	left	3,00000	4,242641	2	
				Total	3,00000	4,242641	2	
	,176	m	85	left	6,00000	,000000	3	
				Total	6,00000	,000000	3	
			Total	left	6,00000	,000000	3	
				Total	6,00000	,000000	3	
		Total	85	left	6,00000	,000000	3	
				Total	6,00000	,000000	3	
			Total	left	6,00000	,000000	3	
				Total	6,00000	,000000	3	
	,177	m	85	left	12,66667	4,041452	3	
				Total	12,66667	4,041452	3	
			Total	left	12,66667	4,041452	3	
				Total	12,66667	4,041452	3	
		Total	85	left	12,66667	4,041452	3	
				Total	12,66667	4,041452	3	
			Total	left	12,66667	4,041452	3	
				Total	12,66667	4,041452	3	
	,180	f	64	righ	3,50000	4,949747	2	
				Total	3,50000	4,949747	2	
			Total	righ	3,50000	4,949747	2	
				Total	3,50000	4,949747	2	
		Total	64	righ	3,50000	4,949747	2	
				Total	3,50000	4,949747	2	
			Total	righ	3,50000	4,949747	2	
				Total	3,50000	4,949747	2	
	,181	f	80	left	7,50000	,707107	2	
				Total	7,50000	,707107	2	
			Total	left	7,50000	,707107	2	
				Total	7,50000	,707107	2	
		Total	80	left	7,50000	,707107	2	
				Total	7,50000	,707107	2	
			Total	left	7,50000	,707107	2	
				Total	7,50000	,707107	2	
	,183	f	85	righ	8,00000	,000000	3	
				Total	8,00000	,000000	3	
			Total	righ	8,00000	,000000	3	
				Total	8,00000	,000000	3	
		Total	85	righ	8,00000	,000000	3	
				Total	8,00000	,000000	3	
			Total	righ	8,00000	,000000	3	
				Total	8,00000	,000000	3	
	,185	m	71	righ	,00000	,000000	3	
				Total	,00000	,000000	3	
			Total	righ	,00000	,000000	3	
				Total	,00000	,000000	3	
		Total	71	righ	,00000	,000000	3	
				Total	,00000	,000000	3	
			Total	righ	,00000	,000000	3	
				Total	,00000	,000000	3	
	,188	m	78	righ	,00000	,000000	3	
				Total	,00000	,000000	3	
			Total	righ	,00000	,000000	3	
				Total	,00000	,000000	3	
		Total	78	righ	,00000	,000000	3	
				Total	,00000	,000000	3	
			Total	righ	,00000	,000000	3	
				Total	,00000	,000000	3	
	,188	m	81	righ	6,43333	1,692139	3	
				Total	6,43333	1,692139	3	
			Total	righ	6,43333	1,692139	3	
				Total	6,43333	1,692139	3	
		Total	81	righ	6,43333	1,692139	3	
				Total	6,43333	1,692139	3	
			Total	righ	6,43333	1,692139	3	
				Total	6,43333	1,692139	3	
	,189	m	85	righ	4,16667	1,258306	3	
				Total	4,16667	1,258306	3	
			Total	righ	4,16667	1,258306	3	
				Total	4,16667	1,258306	3	
		Total	85	righ	4,16667	1,258306	3	
				Total	4,16667	1,258306	3	
			Total	righ	4,16667	1,258306	3	
				Total	4,16667	1,258306	3	
	,190	f	81	righ	3,00000	4,242641	2	
				Total	3,00000	4,242641	2	
			Total	righ	3,00000	4,242641	2	
				Total	3,00000	4,242641	2	
		Total	81	righ	3,00000	4,242641	2	
				Total	3,00000	4,242641	2	
			Total	righ	3,00000	4,242641	2	
				Total	3,00000	4,242641	2	
	,191	f	79	left	7,00000	,000000	3	
				Total	7,00000	,000000	3	
			Total	left	7,00000	,000000	3	
				Total	7,00000	,000000	3	
		Total	79	left	7,00000	,000000	3	
				Total	7,00000	,000000	3	
			Total	left	7,00000	,000000	3	
				Total	7,00000	,000000	3	
	,192	f	78	left	2,00000	1,414214	2	
				Total	2,00000	1,414214	2	
			Total	left	2,00000	1,414214	2	
				Total	2,00000	1,414214	2	
		Total	78	left	2,00000	1,414214	2	
				Total	2,00000	1,414214	2	
			Total	left	2,00000	1,414214	2	
				Total	2,00000	1,414214	2	
	,192	f	71	left	1,66667	1,154701	3	
				Total	1,66667	1,154701	3	
			Total	left	1,66667	1,154701	3	
				Total	1,66667	1,154701	3	
		Total	71	left	1,66667	1,154701	3	
				Total	1,66667	1,154701	3	
			Total	left	1,66667	1,154701	3	
				Total	1,66667	1,154701	3	
	,194	m	85	left	3,50000	,707107	2	
				Total	3,50000	,707107	2	
			Total	left	3,50000	,707107	2	
				Total	3,50000	,707107	2	
		Total	85	left	3,50000	,707107	2	
				Total	3,50000	,707107	2	
			Total	left	3,50000	,707107	2	
				Total	3,50000	,707107	2	
	,198	m	84	righ	2,00000	2,828427	2	
				Total	2,00000	2,828427	2	
			Total	righ	2,00000	2,828427	2	
				Total	2,00000	2,828427	2	
		Total	84	righ	2,00000	2,828427	2	
				Total	2,00000	2,828427	2	
			Total	righ	2,00000	2,828427	2	
				Total	2,00000	2,828427	2	
	,198	m	90	left	1,50000	2,121320	2	
				Total	1,50000	2,121320	2	
			Total	left	1,50000	2,121320	2	
				Total	1,50000	2,121320	2	
		Total	90	left	1,50000	2,121320	2	
				Total	1,50000	2,121320	2	
			Total	left	1,50000	2,121320	2	
				Total	1,50000	2,121320	2	
	,200	m	80	left	4,33333	2,309401	3	
				Total	4,33333	2,309401	3	
			Total	left	4,33333	2,309401	3	
				Total	4,33333	2,309401	3	
		Total	80	left	4,33333	2,309401	3	
				Total	4,33333	2,309401	3	
			Total	left	4,33333	2,309401	3	
				Total	4,33333	2,309401	3	
	,200	m	78	left	4,00000	3,605551	3	
				Total	4,00000	3,605551	3	
			Total	left	4,00000	3,605551	3	
				Total	4,00000	3,605551	3	
		Total	78	left	4,00000	3,605551	3	
				Total	4,00000	3,605551	3	
			Total	left	4,00000	3,605551	3	
				Total	4,00000	3,605551	3	
	,201	m	96	left	4,33333	3,785939	3	
				Total	4,33333	3,785939	3	
			Total	left	4,33333	3,785939	3	
				Total	4,33333	3,785939	3	
		Total	96	left	4,33333	3,785939	3	
				Total	4,33333	3,785939	3	
			Total	left	4,33333	3,785939	3	
				Total	4,33333	3,785939	3	
	,207	m	83	left	7,60000	,000000	3	
				Total	7,60000	,000000	3	
			Total	left	7,60000	,000000	3	
				Total	7,60000	,000000	3	
		Total	83	left	7,60000	,000000	3	
				Total	7,60000	,000000	3	
			Total	left	7,60000	,000000	3	
				Total	7,60000	,000000	3	
	,208	f	83	righ	4,66667	1,154701	3	
				Total	4,66667	1,154701	3	
			Total	righ	4,66667	1,154701	3	
				Total	4,66667	1,154701	3	
		Total	83	righ	4,66667	1,154701	3	
				Total	4,66667	1,154701	3	
			Total	righ	4,66667	1,154701	3	
				Total	4,66667	1,154701	3	
	,210	m	82	left	4,66667	1,527525	3	
				Total	4,66667	1,527525	3	
			Total	left	4,66667	1,527525	3	
				Total	4,66667	1,527525	3	
		Total	82	left	4,66667	1,527525	3	
				Total	4,66667	1,527525	3	
			Total	left	4,66667	1,527525	3	
				Total	4,66667	1,527525	3	
	,211	m	85	left	5,50000	,000000	3	
				Total	5,50000	,000000	3	
			Total	left	5,50000	,000000	3	
				Total	5,50000	,000000	3	
		Total	85	left	5,50000	,000000	3	
				Total	5,50000	,000000	3	
			Total	left	5,50000	,000000	3	
				Total	5,50000	,000000	3	
	,213	m	82	left	6,00000	,000000	3	
				Total	6,00000	,000000	3	
			Total	left	6,00000	,000000	3	
				Total	6,00000	,000000	3	
		Total	82	left	6,00000	,000000	3	
				Total	6,00000	,000000	3	
			Total	left	6,00000	,000000	3	
				Total	6,00000	,000000	3	
	,213	m	82	righ	6,00000	2,828427	2	
				Total	6,00000	2,828427	2	
			Total	righ	6,00000	2,828427	2	
				Total	6,00000	2,828427	2	
		Total	82	righ	6,00000	2,828427	2	
				Total	6,00000	2,828427	2	
			Total	righ	6,00000	2,828427	2	
				Total	6,00000	2,828427	2	
	,214	m	85	righ	5,33333	2,081666	3	
				Total	5,33333	2,081666	3	
			Total	righ	5,33333	2,081666	3	
				Total	5,33333	2,081666	3	
		Total	85	righ	5,33333	2,081666	3	
				Total	5,33333	2,081666	3	
			Total	righ	5,33333	2,081666	3	
				Total	5,33333	2,081666	3	
	,219	f	71	righ	7,33333	2,886751	3	
				Total	7,33333	2,886751	3	
			Total	righ	7,33333	2,886751	3	
				Total	7,33333	2,886751	3	
		Total	71	righ	7,33333	2,886751	3	
				Total	7,33333	2,886751	3	
			Total	righ	7,33333	2,886751	3	
				Total	7,33333	2,886751	3	
	,221	f	74	righ	1,50000	2,121320	2	
				Total	1,50000	2,121320	2	
			Total	righ	1,50000	2,121320	2	
				Total	1,50000	2,121320	2	
		Total	74	righ	1,50000	2,121320	2	
				Total	1,50000	2,121320	2	
			Total	righ	1,50000	2,121320	2	
				Total	1,50000	2,121320	2	
	,224	m	85	righ	7,50000	,707107	2	
				Total	7,50000	,707107	2	
			Total	righ	7,50000	,707107	2	
				Total	7,50000	,707107	2	
		Total	85	righ	7,50000	,707107	2	
				Total	7,50000	,707107	2	
			Total	righ	7,50000	,707107	2	
				Total	7,50000	,707107	2	
	,224	f	97	righ	,00000	,000000	3	
				Total	,00000	,000000	3	
			Total	righ	,00000	,000000	3	
				Total	,00000	,000000	3	
		Total	97	righ	,00000	,000000	3	
				Total	,00000	,000000	3	
			Total	righ	,00000	,000000	3	
				Total	,00000	,000000	3	
	,226	f	82	left	4,07333	2,521137	3	
				Total	4,07333	2,521137	3	
			Total	left	4,07333	2,521137	3	
				Total	4,07333	2,521137	3	
		Total	82	left	4,07333	2,521137	3	
				Total	4,07333	2,521137	3	
			Total	left	4,07333	2,521137	3	
				Total	4,07333	2,521137	3	
	,226	f	83	righ	1,22000	,000000	3	
				Total	1,22000	,000000	3	
			Total	righ	1,22000	,000000	3	
				Total	1,22000	,000000	3	
		Total	83	righ	1,22000	,000000	3	
				Total	1,22000	,000000	3	
			Total	righ	1,22000	,000000	3	
				Total	1,22000	,000000	3	
	,228	m	87	righ	3,33333	2,516611	3	
				Total	3,33333	2,516611	3	
			Total	righ	3,33333	2,516611	3	
				Total	3,33333	2,516611	3	
		Total	87	righ	3,33333	2,516611	3	
				Total	3,33333	2,516611	3	
			Total	righ	3,33333	2,516611	3	
				Total	3,33333	2,516611	3	
	,254	m	85	left	5,20000	2,884441	3	
				Total	5,20000	2,884441	3	
			Total	left	5,20000	2,884441	3	
				Total	5,20000	2,884441	3	
		Total	85	left	5,20000	2,884441	3	
				Total	5,20000	2,884441	3	
			Total	left	5,20000	2,884441	3	
				Total	5,20000	2,884441	3	
	,265	m	85	righ	5,00000	5,567764	3	
				Total	5,00000	5,567764	3	
			Total	righ	5,00000	5,567764	3	
				Total	5,00000	5,567764	3	
		Total	85	righ	5,00000	5,567764	3	
				Total	5,00000	5,567764	3	
			Total	righ	5,00000	5,567764	3	
				Total	5,00000	5,567764	3	
	Total	f	60	left	,33333	,577350	3	
				Total	,33333	,577350	3	
			61	left	1,00000	,000000	3	
				Total	1,00000	,000000	3	
			63	righ	,00000	,000000	3	
				Total	,00000	,000000	3	
			64	righ	3,50000	4,949747	2	
				Total	3,50000	4,949747	2	
			71	left	1,66667	1,154701	3	
				righ	7,33333	2,886751	3	
				Total	4,50000	3,674235	6	
			73	righ	8,35000	,494975	2	
				Total	8,35000	,494975	2	
			74	righ	1,80000	1,303840	5	
				Total	1,80000	1,303840	5	
			78	left	5,32000	3,250692	5	
				Total	5,32000	3,250692	5	
			79	left	7,00000	,000000	3	
				righ	5,00000	3,605551	3	
				Total	6,00000	2,529822	6	
			80	left	4,00000	3,316625	5	
				righ	2,66667	,577350	3	
				Total	3,50000	2,618615	8	
			81	righ	3,80000	2,489980	5	
				Total	3,80000	2,489980	5	
			82	left	4,07333	2,521137	3	
				Total	4,07333	2,521137	3	
			83	righ	2,94333	2,024151	6	
				Total	2,94333	2,024151	6	
			84	left	8,70000	,000000	3	
				righ	2,75000	1,752549	8	
				Total	4,37273	3,142321	11	
			85	righ	8,00000	,000000	3	
				Total	8,00000	,000000	3	
			97	righ	,00000	,000000	3	
				Total	,00000	,000000	3	
			Total	left	4,10429	3,264137	28	
				righ	3,48609	2,970695	46	
				Total	3,72000	3,077653	74	
		m	62	righ	6,00000	2,828427	2	
				Total	6,00000	2,828427	2	
			63	righ	5,75000	,353553	2	
				Total	5,75000	,353553	2	
			66	left	,50000	,707107	2	
				Total	,50000	,707107	2	
			69	righ	5,83333	,288675	3	
				Total	5,83333	,288675	3	
			70	righ	5,23333	3,043572	3	
				Total	5,23333	3,043572	3	
			71	righ	,00000	,000000	3	
				Total	,00000	,000000	3	
			72	righ	2,00000	1,095445	6	
				Total	2,00000	1,095445	6	
			73	left	5,06667	1,590597	9	
				Total	5,06667	1,590597	9	
			75	left	5,00000	3,605551	3	
				righ	6,00000	2,645751	3	
				Total	5,50000	2,880972	6	
			76	left	2,40000	3,286335	5	
				righ	6,50000	2,121320	2	
				Total	3,57143	3,457222	7	
			77	left	8,20000	3,563706	5	
				Total	8,20000	3,563706	5	
			78	left	4,00000	3,605551	3	
				righ	2,06000	3,594162	5	
				Total	2,78750	3,479096	8	
			80	left	4,33333	2,309401	3	
				righ	3,50000	3,535534	2	
				Total	4,00000	2,449490	5	
			81	righ	6,43333	1,692139	3	
				Total	6,43333	1,692139	3	
			82	left	5,33333	1,211060	6	
				righ	6,00000	2,828427	2	
				Total	5,50000	1,511858	8	
			83	left	7,60000	,000000	3	
				Total	7,60000	,000000	3	
			84	righ	2,00000	2,828427	2	
				Total	2,00000	2,828427	2	
			85	left	6,30000	3,663844	17	
				righ	5,31818	2,968777	11	
				Total	5,91429	3,384903	28	
			87	righ	3,33333	2,516611	3	
				Total	3,33333	2,516611	3	
			88	righ	1,66667	1,154701	3	
				Total	1,66667	1,154701	3	
			90	left	1,50000	2,121320	2	
				Total	1,50000	2,121320	2	
			96	left	4,33333	3,785939	3	
				Total	4,33333	3,785939	3	
			Total	left	5,20492	3,254506	61	
				righ	4,10545	2,903409	55	
				Total	4,68362	3,128660	116	
		Total	60	left	,33333	,577350	3	
				Total	,33333	,577350	3	
			61	left	1,00000	,000000	3	
				Total	1,00000	,000000	3	
			62	righ	6,00000	2,828427	2	
				Total	6,00000	2,828427	2	
			63	righ	2,30000	3,154362	5	
				Total	2,30000	3,154362	5	
			64	righ	3,50000	4,949747	2	
				Total	3,50000	4,949747	2	
			66	left	,50000	,707107	2	
				Total	,50000	,707107	2	
			69	righ	5,83333	,288675	3	
				Total	5,83333	,288675	3	
			70	righ	5,23333	3,043572	3	
				Total	5,23333	3,043572	3	
			71	left	1,66667	1,154701	3	
				righ	3,66667	4,412105	6	
				Total	3,00000	3,674235	9	
			72	righ	2,00000	1,095445	6	
				Total	2,00000	1,095445	6	
			73	left	5,06667	1,590597	9	
				righ	8,35000	,494975	2	
				Total	5,66364	1,952574	11	
			74	righ	1,80000	1,303840	5	
				Total	1,80000	1,303840	5	
			75	left	5,00000	3,605551	3	
				righ	6,00000	2,645751	3	
				Total	5,50000	2,880972	6	
			76	left	2,40000	3,286335	5	
				righ	6,50000	2,121320	2	
				Total	3,57143	3,457222	7	
			77	left	8,20000	3,563706	5	
				Total	8,20000	3,563706	5	
			78	left	4,82500	3,196762	8	
				righ	2,06000	3,594162	5	
				Total	3,76154	3,496793	13	
			79	left	7,00000	,000000	3	
				righ	5,00000	3,605551	3	
				Total	6,00000	2,529822	6	
			80	left	4,12500	2,799872	8	
				righ	3,00000	1,870829	5	
				Total	3,69231	2,462540	13	
			81	righ	4,78750	2,493671	8	
				Total	4,78750	2,493671	8	
			82	left	4,91333	1,703702	9	
				righ	6,00000	2,828427	2	
				Total	5,11091	1,820799	11	
			83	left	7,60000	,000000	3	
				righ	2,94333	2,024151	6	
				Total	4,49556	2,825222	9	
			84	left	8,70000	,000000	3	
				righ	2,60000	1,837873	10	
				Total	4,00769	3,112732	13	
			85	left	6,30000	3,663844	17	
				righ	5,89286	2,843201	14	
				Total	6,11613	3,271809	31	
			87	righ	3,33333	2,516611	3	
				Total	3,33333	2,516611	3	
			88	righ	1,66667	1,154701	3	
				Total	1,66667	1,154701	3	
			90	left	1,50000	2,121320	2	
				Total	1,50000	2,121320	2	
			96	left	4,33333	3,785939	3	
				Total	4,33333	3,785939	3	
			97	righ	,00000	,000000	3	
				Total	,00000	,000000	3	
			Total	left	4,85865	3,279465	89	
				righ	3,82337	2,935888	101	
				Total	4,30832	3,136306	190	
Shift pre	,100	m	63	righ	8,00000	,000000	2	
				Total	8,00000	,000000	2	
			Total	righ	8,00000	,000000	2	
				Total	8,00000	,000000	2	
		Total	63	righ	8,00000	,000000	2	
				Total	8,00000	,000000	2	
			Total	righ	8,00000	,000000	2	
				Total	8,00000	,000000	2	
	,111	m	70	righ	7,00000	,000000	3	
				Total	7,00000	,000000	3	
			Total	righ	7,00000	,000000	3	
				Total	7,00000	,000000	3	
		Total	70	righ	7,00000	,000000	3	
				Total	7,00000	,000000	3	
			Total	righ	7,00000	,000000	3	
				Total	7,00000	,000000	3	
	,120	m	72	righ	7,00000	,000000	3	
				Total	7,00000	,000000	3	
			Total	righ	7,00000	,000000	3	
				Total	7,00000	,000000	3	
		Total	72	righ	7,00000	,000000	3	
				Total	7,00000	,000000	3	
			Total	righ	7,00000	,000000	3	
				Total	7,00000	,000000	3	
	,125	m	73	left	10,00000	,000000	3	
				Total	10,00000	,000000	3	
			Total	left	10,00000	,000000	3	
				Total	10,00000	,000000	3	
		Total	73	left	10,00000	,000000	3	
				Total	10,00000	,000000	3	
			Total	left	10,00000	,000000	3	
				Total	10,00000	,000000	3	
	,127	f	74	righ	9,00000	,000000	3	
				Total	9,00000	,000000	3	
			Total	righ	9,00000	,000000	3	
				Total	9,00000	,000000	3	
		Total	74	righ	9,00000	,000000	3	
				Total	9,00000	,000000	3	
			Total	righ	9,00000	,000000	3	
				Total	9,00000	,000000	3	
	,131	m	66	left	6,00000	,000000	2	
				Total	6,00000	,000000	2	
			Total	left	6,00000	,000000	2	
				Total	6,00000	,000000	2	
		Total	66	left	6,00000	,000000	2	
				Total	6,00000	,000000	2	
			Total	left	6,00000	,000000	2	
				Total	6,00000	,000000	2	
	,131	f	84	righ	7,00000	,000000	3	
				Total	7,00000	,000000	3	
			Total	righ	7,00000	,000000	3	
				Total	7,00000	,000000	3	
		Total	84	righ	7,00000	,000000	3	
				Total	7,00000	,000000	3	
			Total	righ	7,00000	,000000	3	
				Total	7,00000	,000000	3	
	,131	m	75	righ	7,40000	,000000	3	
				Total	7,40000	,000000	3	
			Total	righ	7,40000	,000000	3	
				Total	7,40000	,000000	3	
		Total	75	righ	7,40000	,000000	3	
				Total	7,40000	,000000	3	
			Total	righ	7,40000	,000000	3	
				Total	7,40000	,000000	3	
	,134	m	76	left	5,00000	,000000	3	
				Total	5,00000	,000000	3	
			Total	left	5,00000	,000000	3	
				Total	5,00000	,000000	3	
		Total	76	left	5,00000	,000000	3	
				Total	5,00000	,000000	3	
			Total	left	5,00000	,000000	3	
				Total	5,00000	,000000	3	
	,134	m	69	righ	8,00000	,000000	3	
				Total	8,00000	,000000	3	
			Total	righ	8,00000	,000000	3	
				Total	8,00000	,000000	3	
		Total	69	righ	8,00000	,000000	3	
				Total	8,00000	,000000	3	
			Total	righ	8,00000	,000000	3	
				Total	8,00000	,000000	3	
	,141	m	80	righ	8,00000	,000000	2	
				Total	8,00000	,000000	2	
			Total	righ	8,00000	,000000	2	
				Total	8,00000	,000000	2	
		Total	80	righ	8,00000	,000000	2	
				Total	8,00000	,000000	2	
			Total	righ	8,00000	,000000	2	
				Total	8,00000	,000000	2	
	,143	f	63	righ	18,00000	,000000	3	
				Total	18,00000	,000000	3	
			Total	righ	18,00000	,000000	3	
				Total	18,00000	,000000	3	
		Total	63	righ	18,00000	,000000	3	
				Total	18,00000	,000000	3	
			Total	righ	18,00000	,000000	3	
				Total	18,00000	,000000	3	
	,144	f	73	righ	7,00000	,000000	2	
				Total	7,00000	,000000	2	
			Total	righ	7,00000	,000000	2	
				Total	7,00000	,000000	2	
		Total	73	righ	7,00000	,000000	2	
				Total	7,00000	,000000	2	
			Total	righ	7,00000	,000000	2	
				Total	7,00000	,000000	2	
	,146	m	78	righ	15,00000	,000000	2	
				Total	15,00000	,000000	2	
			Total	righ	15,00000	,000000	2	
				Total	15,00000	,000000	2	
		Total	78	righ	15,00000	,000000	2	
				Total	15,00000	,000000	2	
			Total	righ	15,00000	,000000	2	
				Total	15,00000	,000000	2	
	,146	f	61	left	8,00000	,000000	3	
				Total	8,00000	,000000	3	
			Total	left	8,00000	,000000	3	
				Total	8,00000	,000000	3	
		Total	61	left	8,00000	,000000	3	
				Total	8,00000	,000000	3	
			Total	left	8,00000	,000000	3	
				Total	8,00000	,000000	3	
	,148	f	81	righ	12,00000	,000000	3	
				Total	12,00000	,000000	3	
			Total	righ	12,00000	,000000	3	
				Total	12,00000	,000000	3	
		Total	81	righ	12,00000	,000000	3	
				Total	12,00000	,000000	3	
			Total	righ	12,00000	,000000	3	
				Total	12,00000	,000000	3	
	,148	f	84	left	10,00000	,000000	3	
				Total	10,00000	,000000	3	
			Total	left	10,00000	,000000	3	
				Total	10,00000	,000000	3	
		Total	84	left	10,00000	,000000	3	
				Total	10,00000	,000000	3	
			Total	left	10,00000	,000000	3	
				Total	10,00000	,000000	3	
	,150	f	80	left	5,00000	,000000	3	
				Total	5,00000	,000000	3	
			Total	left	5,00000	,000000	3	
				Total	5,00000	,000000	3	
		Total	80	left	5,00000	,000000	3	
				Total	5,00000	,000000	3	
			Total	left	5,00000	,000000	3	
				Total	5,00000	,000000	3	
	,152	m	73	left	9,00000	,000000	3	
				Total	9,00000	,000000	3	
			Total	left	9,00000	,000000	3	
				Total	9,00000	,000000	3	
		Total	73	left	9,00000	,000000	3	
				Total	9,00000	,000000	3	
			Total	left	9,00000	,000000	3	
				Total	9,00000	,000000	3	
	,152	m	72	righ	13,80000	,000000	3	
				Total	13,80000	,000000	3	
			Total	righ	13,80000	,000000	3	
				Total	13,80000	,000000	3	
		Total	72	righ	13,80000	,000000	3	
				Total	13,80000	,000000	3	
			Total	righ	13,80000	,000000	3	
				Total	13,80000	,000000	3	
	,156	m	85	left	11,90000	,000000	3	
				Total	11,90000	,000000	3	
			Total	left	11,90000	,000000	3	
				Total	11,90000	,000000	3	
		Total	85	left	11,90000	,000000	3	
				Total	11,90000	,000000	3	
			Total	left	11,90000	,000000	3	
				Total	11,90000	,000000	3	
	,159	m	88	righ	6,00000	,000000	3	
				Total	6,00000	,000000	3	
			Total	righ	6,00000	,000000	3	
				Total	6,00000	,000000	3	
		Total	88	righ	6,00000	,000000	3	
				Total	6,00000	,000000	3	
			Total	righ	6,00000	,000000	3	
				Total	6,00000	,000000	3	
	,159	m	77	left	4,00000	,000000	3	
				Total	4,00000	,000000	3	
			Total	left	4,00000	,000000	3	
				Total	4,00000	,000000	3	
		Total	77	left	4,00000	,000000	3	
				Total	4,00000	,000000	3	
			Total	left	4,00000	,000000	3	
				Total	4,00000	,000000	3	
	,159	f	79	righ	6,70000	,000000	3	
				Total	6,70000	,000000	3	
			Total	righ	6,70000	,000000	3	
				Total	6,70000	,000000	3	
		Total	79	righ	6,70000	,000000	3	
				Total	6,70000	,000000	3	
			Total	righ	6,70000	,000000	3	
				Total	6,70000	,000000	3	
	,160	f	80	righ	4,50000	,000000	3	
				Total	4,50000	,000000	3	
			Total	righ	4,50000	,000000	3	
				Total	4,50000	,000000	3	
		Total	80	righ	4,50000	,000000	3	
				Total	4,50000	,000000	3	
			Total	righ	4,50000	,000000	3	
				Total	4,50000	,000000	3	
	,163	m	75	left	4,00000	,000000	3	
				Total	4,00000	,000000	3	
			Total	left	4,00000	,000000	3	
				Total	4,00000	,000000	3	
		Total	75	left	4,00000	,000000	3	
				Total	4,00000	,000000	3	
			Total	left	4,00000	,000000	3	
				Total	4,00000	,000000	3	
	,167	m	73	left	7,00000	,000000	3	
				Total	7,00000	,000000	3	
			Total	left	7,00000	,000000	3	
				Total	7,00000	,000000	3	
		Total	73	left	7,00000	,000000	3	
				Total	7,00000	,000000	3	
			Total	left	7,00000	,000000	3	
				Total	7,00000	,000000	3	
	,168	f	60	left	15,00000	,000000	3	
				Total	15,00000	,000000	3	
			Total	left	15,00000	,000000	3	
				Total	15,00000	,000000	3	
		Total	60	left	15,00000	,000000	3	
				Total	15,00000	,000000	3	
			Total	left	15,00000	,000000	3	
				Total	15,00000	,000000	3	
	,170	m	62	righ	15,00000	,000000	2	
				Total	15,00000	,000000	2	
			Total	righ	15,00000	,000000	2	
				Total	15,00000	,000000	2	
		Total	62	righ	15,00000	,000000	2	
				Total	15,00000	,000000	2	
			Total	righ	15,00000	,000000	2	
				Total	15,00000	,000000	2	
	,170	f	84	righ	5,00000	,000000	2	
				Total	5,00000	,000000	2	
			Total	righ	5,00000	,000000	2	
				Total	5,00000	,000000	2	
		Total	84	righ	5,00000	,000000	2	
				Total	5,00000	,000000	2	
			Total	righ	5,00000	,000000	2	
				Total	5,00000	,000000	2	
	,171	f	78	left	12,00000	,000000	3	
				Total	12,00000	,000000	3	
			Total	left	12,00000	,000000	3	
				Total	12,00000	,000000	3	
		Total	78	left	12,00000	,000000	3	
				Total	12,00000	,000000	3	
			Total	left	12,00000	,000000	3	
				Total	12,00000	,000000	3	
	,172	f	84	righ	12,00000	,000000	3	
				Total	12,00000	,000000	3	
			Total	righ	12,00000	,000000	3	
				Total	12,00000	,000000	3	
		Total	84	righ	12,00000	,000000	3	
				Total	12,00000	,000000	3	
			Total	righ	12,00000	,000000	3	
				Total	12,00000	,000000	3	
	,173	m	76	righ	10,00000	,000000	2	
				Total	10,00000	,000000	2	
			Total	righ	10,00000	,000000	2	
				Total	10,00000	,000000	2	
		Total	76	righ	10,00000	,000000	2	
				Total	10,00000	,000000	2	
			Total	righ	10,00000	,000000	2	
				Total	10,00000	,000000	2	
	,174	m	77	left	11,00000	,000000	2	
				Total	11,00000	,000000	2	
			Total	left	11,00000	,000000	2	
				Total	11,00000	,000000	2	
		Total	77	left	11,00000	,000000	2	
				Total	11,00000	,000000	2	
			Total	left	11,00000	,000000	2	
				Total	11,00000	,000000	2	
	,175	m	76	left	6,50000	,000000	2	
				Total	6,50000	,000000	2	
			Total	left	6,50000	,000000	2	
				Total	6,50000	,000000	2	
		Total	76	left	6,50000	,000000	2	
				Total	6,50000	,000000	2	
			Total	left	6,50000	,000000	2	
				Total	6,50000	,000000	2	
	,176	m	85	left	9,60000	,000000	3	
				Total	9,60000	,000000	3	
			Total	left	9,60000	,000000	3	
				Total	9,60000	,000000	3	
		Total	85	left	9,60000	,000000	3	
				Total	9,60000	,000000	3	
			Total	left	9,60000	,000000	3	
				Total	9,60000	,000000	3	
	,177	m	85	left	10,00000	,000000	3	
				Total	10,00000	,000000	3	
			Total	left	10,00000	,000000	3	
				Total	10,00000	,000000	3	
		Total	85	left	10,00000	,000000	3	
				Total	10,00000	,000000	3	
			Total	left	10,00000	,000000	3	
				Total	10,00000	,000000	3	
	,180	f	64	righ	9,00000	,000000	2	
				Total	9,00000	,000000	2	
			Total	righ	9,00000	,000000	2	
				Total	9,00000	,000000	2	
		Total	64	righ	9,00000	,000000	2	
				Total	9,00000	,000000	2	
			Total	righ	9,00000	,000000	2	
				Total	9,00000	,000000	2	
	,181	f	80	left	6,60000	,000000	2	
				Total	6,60000	,000000	2	
			Total	left	6,60000	,000000	2	
				Total	6,60000	,000000	2	
		Total	80	left	6,60000	,000000	2	
				Total	6,60000	,000000	2	
			Total	left	6,60000	,000000	2	
				Total	6,60000	,000000	2	
	,183	f	85	righ	8,50000	,000000	3	
				Total	8,50000	,000000	3	
			Total	righ	8,50000	,000000	3	
				Total	8,50000	,000000	3	
		Total	85	righ	8,50000	,000000	3	
				Total	8,50000	,000000	3	
			Total	righ	8,50000	,000000	3	
				Total	8,50000	,000000	3	
	,185	m	71	righ	6,00000	,000000	3	
				Total	6,00000	,000000	3	
			Total	righ	6,00000	,000000	3	
				Total	6,00000	,000000	3	
		Total	71	righ	6,00000	,000000	3	
				Total	6,00000	,000000	3	
			Total	righ	6,00000	,000000	3	
				Total	6,00000	,000000	3	
	,188	m	78	righ	5,00000	,000000	3	
				Total	5,00000	,000000	3	
			Total	righ	5,00000	,000000	3	
				Total	5,00000	,000000	3	
		Total	78	righ	5,00000	,000000	3	
				Total	5,00000	,000000	3	
			Total	righ	5,00000	,000000	3	
				Total	5,00000	,000000	3	
	,188	m	81	righ	7,00000	,000000	3	
				Total	7,00000	,000000	3	
			Total	righ	7,00000	,000000	3	
				Total	7,00000	,000000	3	
		Total	81	righ	7,00000	,000000	3	
				Total	7,00000	,000000	3	
			Total	righ	7,00000	,000000	3	
				Total	7,00000	,000000	3	
	,189	m	85	righ	9,30000	,000000	3	
				Total	9,30000	,000000	3	
			Total	righ	9,30000	,000000	3	
				Total	9,30000	,000000	3	
		Total	85	righ	9,30000	,000000	3	
				Total	9,30000	,000000	3	
			Total	righ	9,30000	,000000	3	
				Total	9,30000	,000000	3	
	,190	f	81	righ	16,50000	,000000	2	
				Total	16,50000	,000000	2	
			Total	righ	16,50000	,000000	2	
				Total	16,50000	,000000	2	
		Total	81	righ	16,50000	,000000	2	
				Total	16,50000	,000000	2	
			Total	righ	16,50000	,000000	2	
				Total	16,50000	,000000	2	
	,191	f	79	left	11,00000	,000000	3	
				Total	11,00000	,000000	3	
			Total	left	11,00000	,000000	3	
				Total	11,00000	,000000	3	
		Total	79	left	11,00000	,000000	3	
				Total	11,00000	,000000	3	
			Total	left	11,00000	,000000	3	
				Total	11,00000	,000000	3	
	,192	f	78	left	9,00000	,000000	2	
				Total	9,00000	,000000	2	
			Total	left	9,00000	,000000	2	
				Total	9,00000	,000000	2	
		Total	78	left	9,00000	,000000	2	
				Total	9,00000	,000000	2	
			Total	left	9,00000	,000000	2	
				Total	9,00000	,000000	2	
	,192	f	71	left	14,00000	,000000	3	
				Total	14,00000	,000000	3	
			Total	left	14,00000	,000000	3	
				Total	14,00000	,000000	3	
		Total	71	left	14,00000	,000000	3	
				Total	14,00000	,000000	3	
			Total	left	14,00000	,000000	3	
				Total	14,00000	,000000	3	
	,194	m	85	left	4,00000	,000000	2	
				Total	4,00000	,000000	2	
			Total	left	4,00000	,000000	2	
				Total	4,00000	,000000	2	
		Total	85	left	4,00000	,000000	2	
				Total	4,00000	,000000	2	
			Total	left	4,00000	,000000	2	
				Total	4,00000	,000000	2	
	,198	m	84	righ	7,00000	,000000	2	
				Total	7,00000	,000000	2	
			Total	righ	7,00000	,000000	2	
				Total	7,00000	,000000	2	
		Total	84	righ	7,00000	,000000	2	
				Total	7,00000	,000000	2	
			Total	righ	7,00000	,000000	2	
				Total	7,00000	,000000	2	
	,198	m	90	left	12,00000	,000000	2	
				Total	12,00000	,000000	2	
			Total	left	12,00000	,000000	2	
				Total	12,00000	,000000	2	
		Total	90	left	12,00000	,000000	2	
				Total	12,00000	,000000	2	
			Total	left	12,00000	,000000	2	
				Total	12,00000	,000000	2	
	,200	m	80	left	10,00000	,000000	3	
				Total	10,00000	,000000	3	
			Total	left	10,00000	,000000	3	
				Total	10,00000	,000000	3	
		Total	80	left	10,00000	,000000	3	
				Total	10,00000	,000000	3	
			Total	left	10,00000	,000000	3	
				Total	10,00000	,000000	3	
	,200	m	78	left	8,60000	,000000	3	
				Total	8,60000	,000000	3	
			Total	left	8,60000	,000000	3	
				Total	8,60000	,000000	3	
		Total	78	left	8,60000	,000000	3	
				Total	8,60000	,000000	3	
			Total	left	8,60000	,000000	3	
				Total	8,60000	,000000	3	
	,201	m	96	left	8,00000	,000000	3	
				Total	8,00000	,000000	3	
			Total	left	8,00000	,000000	3	
				Total	8,00000	,000000	3	
		Total	96	left	8,00000	,000000	3	
				Total	8,00000	,000000	3	
			Total	left	8,00000	,000000	3	
				Total	8,00000	,000000	3	
	,207	m	83	left	9,00000	,000000	3	
				Total	9,00000	,000000	3	
			Total	left	9,00000	,000000	3	
				Total	9,00000	,000000	3	
		Total	83	left	9,00000	,000000	3	
				Total	9,00000	,000000	3	
			Total	left	9,00000	,000000	3	
				Total	9,00000	,000000	3	
	,208	f	83	righ	20,10000	,000000	3	
				Total	20,10000	,000000	3	
			Total	righ	20,10000	,000000	3	
				Total	20,10000	,000000	3	
		Total	83	righ	20,10000	,000000	3	
				Total	20,10000	,000000	3	
			Total	righ	20,10000	,000000	3	
				Total	20,10000	,000000	3	
	,210	m	82	left	5,00000	,000000	3	
				Total	5,00000	,000000	3	
			Total	left	5,00000	,000000	3	
				Total	5,00000	,000000	3	
		Total	82	left	5,00000	,000000	3	
				Total	5,00000	,000000	3	
			Total	left	5,00000	,000000	3	
				Total	5,00000	,000000	3	
	,211	m	85	left	8,00000	,000000	3	
				Total	8,00000	,000000	3	
			Total	left	8,00000	,000000	3	
				Total	8,00000	,000000	3	
		Total	85	left	8,00000	,000000	3	
				Total	8,00000	,000000	3	
			Total	left	8,00000	,000000	3	
				Total	8,00000	,000000	3	
	,213	m	82	left	8,00000	,000000	3	
				Total	8,00000	,000000	3	
			Total	left	8,00000	,000000	3	
				Total	8,00000	,000000	3	
		Total	82	left	8,00000	,000000	3	
				Total	8,00000	,000000	3	
			Total	left	8,00000	,000000	3	
				Total	8,00000	,000000	3	
	,213	m	82	righ	10,00000	,000000	2	
				Total	10,00000	,000000	2	
			Total	righ	10,00000	,000000	2	
				Total	10,00000	,000000	2	
		Total	82	righ	10,00000	,000000	2	
				Total	10,00000	,000000	2	
			Total	righ	10,00000	,000000	2	
				Total	10,00000	,000000	2	
	,214	m	85	righ	6,00000	,000000	3	
				Total	6,00000	,000000	3	
			Total	righ	6,00000	,000000	3	
				Total	6,00000	,000000	3	
		Total	85	righ	6,00000	,000000	3	
				Total	6,00000	,000000	3	
			Total	righ	6,00000	,000000	3	
				Total	6,00000	,000000	3	
	,219	f	71	righ	12,00000	,000000	3	
				Total	12,00000	,000000	3	
			Total	righ	12,00000	,000000	3	
				Total	12,00000	,000000	3	
		Total	71	righ	12,00000	,000000	3	
				Total	12,00000	,000000	3	
			Total	righ	12,00000	,000000	3	
				Total	12,00000	,000000	3	
	,221	f	74	righ	13,00000	,000000	2	
				Total	13,00000	,000000	2	
			Total	righ	13,00000	,000000	2	
				Total	13,00000	,000000	2	
		Total	74	righ	13,00000	,000000	2	
				Total	13,00000	,000000	2	
			Total	righ	13,00000	,000000	2	
				Total	13,00000	,000000	2	
	,224	m	85	righ	10,00000	,000000	2	
				Total	10,00000	,000000	2	
			Total	righ	10,00000	,000000	2	
				Total	10,00000	,000000	2	
		Total	85	righ	10,00000	,000000	2	
				Total	10,00000	,000000	2	
			Total	righ	10,00000	,000000	2	
				Total	10,00000	,000000	2	
	,224	f	97	righ	2,00000	,000000	3	
				Total	2,00000	,000000	3	
			Total	righ	2,00000	,000000	3	
				Total	2,00000	,000000	3	
		Total	97	righ	2,00000	,000000	3	
				Total	2,00000	,000000	3	
			Total	righ	2,00000	,000000	3	
				Total	2,00000	,000000	3	
	,226	f	82	left	7,70000	,000000	3	
				Total	7,70000	,000000	3	
			Total	left	7,70000	,000000	3	
				Total	7,70000	,000000	3	
		Total	82	left	7,70000	,000000	3	
				Total	7,70000	,000000	3	
			Total	left	7,70000	,000000	3	
				Total	7,70000	,000000	3	
	,226	f	83	righ	15,00000	,000000	3	
				Total	15,00000	,000000	3	
			Total	righ	15,00000	,000000	3	
				Total	15,00000	,000000	3	
		Total	83	righ	15,00000	,000000	3	
				Total	15,00000	,000000	3	
			Total	righ	15,00000	,000000	3	
				Total	15,00000	,000000	3	
	,228	m	87	righ	13,00000	,000000	3	
				Total	13,00000	,000000	3	
			Total	righ	13,00000	,000000	3	
				Total	13,00000	,000000	3	
		Total	87	righ	13,00000	,000000	3	
				Total	13,00000	,000000	3	
			Total	righ	13,00000	,000000	3	
				Total	13,00000	,000000	3	
	,254	m	85	left	6,00000	,000000	3	
				Total	6,00000	,000000	3	
			Total	left	6,00000	,000000	3	
				Total	6,00000	,000000	3	
		Total	85	left	6,00000	,000000	3	
				Total	6,00000	,000000	3	
			Total	left	6,00000	,000000	3	
				Total	6,00000	,000000	3	
	,265	m	85	righ	4,00000	,000000	3	
				Total	4,00000	,000000	3	
			Total	righ	4,00000	,000000	3	
				Total	4,00000	,000000	3	
		Total	85	righ	4,00000	,000000	3	
				Total	4,00000	,000000	3	
			Total	righ	4,00000	,000000	3	
				Total	4,00000	,000000	3	
	Total	f	60	left	15,00000	,000000	3	
				Total	15,00000	,000000	3	
			61	left	8,00000	,000000	3	
				Total	8,00000	,000000	3	
			63	righ	18,00000	,000000	3	
				Total	18,00000	,000000	3	
			64	righ	9,00000	,000000	2	
				Total	9,00000	,000000	2	
			71	left	14,00000	,000000	3	
				righ	12,00000	,000000	3	
				Total	13,00000	1,095445	6	
			73	righ	7,00000	,000000	2	
				Total	7,00000	,000000	2	
			74	righ	10,60000	2,190890	5	
				Total	10,60000	2,190890	5	
			78	left	10,80000	1,643168	5	
				Total	10,80000	1,643168	5	
			79	left	11,00000	,000000	3	
				righ	6,70000	,000000	3	
				Total	8,85000	2,355207	6	
			80	left	5,64000	,876356	5	
				righ	4,50000	,000000	3	
				Total	5,21250	,887110	8	
			81	righ	13,80000	2,464752	5	
				Total	13,80000	2,464752	5	
			82	left	7,70000	,000000	3	
				Total	7,70000	,000000	3	
			83	righ	17,55000	2,793385	6	
				Total	17,55000	2,793385	6	
			84	left	10,00000	,000000	3	
				righ	8,37500	3,113909	8	
				Total	8,81818	2,713602	11	
			85	righ	8,50000	,000000	3	
				Total	8,50000	,000000	3	
			97	righ	2,00000	,000000	3	
				Total	2,00000	,000000	3	
			Total	left	9,97500	3,138604	28	
				righ	10,46522	4,997209	46	
				Total	10,27973	4,369727	74	
		m	62	righ	15,00000	,000000	2	
				Total	15,00000	,000000	2	
			63	righ	8,00000	,000000	2	
				Total	8,00000	,000000	2	
			66	left	6,00000	,000000	2	
				Total	6,00000	,000000	2	
			69	righ	8,00000	,000000	3	
				Total	8,00000	,000000	3	
			70	righ	7,00000	,000000	3	
				Total	7,00000	,000000	3	
			71	righ	6,00000	,000000	3	
				Total	6,00000	,000000	3	
			72	righ	10,40000	3,724513	6	
				Total	10,40000	3,724513	6	
			73	left	8,66667	1,322876	9	
				Total	8,66667	1,322876	9	
			75	left	4,00000	,000000	3	
				righ	7,40000	,000000	3	
				Total	5,70000	1,862257	6	
			76	left	5,60000	,821584	5	
				righ	10,00000	,000000	2	
				Total	6,85714	2,249339	7	
			77	left	6,80000	3,834058	5	
				Total	6,80000	3,834058	5	
			78	left	8,60000	,000000	3	
				righ	9,00000	5,477226	5	
				Total	8,85000	4,145566	8	
			80	left	10,00000	,000000	3	
				righ	8,00000	,000000	2	
				Total	9,20000	1,095445	5	
			81	righ	7,00000	,000000	3	
				Total	7,00000	,000000	3	
			82	left	6,50000	1,643168	6	
				righ	10,00000	,000000	2	
				Total	7,37500	2,133910	8	
			83	left	9,00000	,000000	3	
				Total	9,00000	,000000	3	
			84	righ	7,00000	,000000	2	
				Total	7,00000	,000000	2	
			85	left	8,50000	2,562470	17	
				righ	7,08182	2,525794	11	
				Total	7,94286	2,598351	28	
			87	righ	13,00000	,000000	3	
				Total	13,00000	,000000	3	
			88	righ	6,00000	,000000	3	
				Total	6,00000	,000000	3	
			90	left	12,00000	,000000	2	
				Total	12,00000	,000000	2	
			96	left	8,00000	,000000	3	
				Total	8,00000	,000000	3	
			Total	left	7,84098	2,430184	61	
				righ	8,44545	3,082677	55	
				Total	8,12759	2,763224	116	
		Total	60	left	15,00000	,000000	3	
				Total	15,00000	,000000	3	
			61	left	8,00000	,000000	3	
				Total	8,00000	,000000	3	
			62	righ	15,00000	,000000	2	
				Total	15,00000	,000000	2	
			63	righ	14,00000	5,477226	5	
				Total	14,00000	5,477226	5	
			64	righ	9,00000	,000000	2	
				Total	9,00000	,000000	2	
			66	left	6,00000	,000000	2	
				Total	6,00000	,000000	2	
			69	righ	8,00000	,000000	3	
				Total	8,00000	,000000	3	
			70	righ	7,00000	,000000	3	
				Total	7,00000	,000000	3	
			71	left	14,00000	,000000	3	
				righ	9,00000	3,286335	6	
				Total	10,66667	3,605551	9	
			72	righ	10,40000	3,724513	6	
				Total	10,40000	3,724513	6	
			73	left	8,66667	1,322876	9	
				righ	7,00000	,000000	2	
				Total	8,36364	1,361817	11	
			74	righ	10,60000	2,190890	5	
				Total	10,60000	2,190890	5	
			75	left	4,00000	,000000	3	
				righ	7,40000	,000000	3	
				Total	5,70000	1,862257	6	
			76	left	5,60000	,821584	5	
				righ	10,00000	,000000	2	
				Total	6,85714	2,249339	7	
			77	left	6,80000	3,834058	5	
				Total	6,80000	3,834058	5	
			78	left	9,97500	1,685018	8	
				righ	9,00000	5,477226	5	
				Total	9,60000	3,449638	13	
			79	left	11,00000	,000000	3	
				righ	6,70000	,000000	3	
				Total	8,85000	2,355207	6	
			80	left	7,27500	2,351747	8	
				righ	5,90000	1,917029	5	
				Total	6,74615	2,221717	13	
			81	righ	11,25000	3,982103	8	
				Total	11,25000	3,982103	8	
			82	left	6,90000	1,430909	9	
				righ	10,00000	,000000	2	
				Total	7,46364	1,791800	11	
			83	left	9,00000	,000000	3	
				righ	17,55000	2,793385	6	
				Total	14,70000	4,811704	9	
			84	left	10,00000	,000000	3	
				righ	8,10000	2,806738	10	
				Total	8,53846	2,569546	13	
			85	left	8,50000	2,562470	17	
				righ	7,38571	2,296103	14	
				Total	7,99677	2,470693	31	
			87	righ	13,00000	,000000	3	
				Total	13,00000	,000000	3	
			88	righ	6,00000	,000000	3	
				Total	6,00000	,000000	3	
			90	left	12,00000	,000000	2	
				Total	12,00000	,000000	2	
			96	left	8,00000	,000000	3	
				Total	8,00000	,000000	3	
			97	righ	2,00000	,000000	3	
				Total	2,00000	,000000	3	
			Total	left	8,51236	2,835883	89	
				righ	9,36535	4,170238	101	
				Total	8,96579	3,623284	190	
Shift post 30	,100	m	63	righ	3,00000	,000000	2	
				Total	3,00000	,000000	2	
			Total	righ	3,00000	,000000	2	
				Total	3,00000	,000000	2	
		Total	63	righ	3,00000	,000000	2	
				Total	3,00000	,000000	2	
			Total	righ	3,00000	,000000	2	
				Total	3,00000	,000000	2	
	,111	m	70	righ	3,00000	,000000	3	
				Total	3,00000	,000000	3	
			Total	righ	3,00000	,000000	3	
				Total	3,00000	,000000	3	
		Total	70	righ	3,00000	,000000	3	
				Total	3,00000	,000000	3	
			Total	righ	3,00000	,000000	3	
				Total	3,00000	,000000	3	
	,120	m	72	righ	2,00000	,000000	3	
				Total	2,00000	,000000	3	
			Total	righ	2,00000	,000000	3	
				Total	2,00000	,000000	3	
		Total	72	righ	2,00000	,000000	3	
				Total	2,00000	,000000	3	
			Total	righ	2,00000	,000000	3	
				Total	2,00000	,000000	3	
	,125	m	73	left	3,00000	,000000	3	
				Total	3,00000	,000000	3	
			Total	left	3,00000	,000000	3	
				Total	3,00000	,000000	3	
		Total	73	left	3,00000	,000000	3	
				Total	3,00000	,000000	3	
			Total	left	3,00000	,000000	3	
				Total	3,00000	,000000	3	
	,127	f	74	righ	2,00000	,000000	3	
				Total	2,00000	,000000	3	
			Total	righ	2,00000	,000000	3	
				Total	2,00000	,000000	3	
		Total	74	righ	2,00000	,000000	3	
				Total	2,00000	,000000	3	
			Total	righ	2,00000	,000000	3	
				Total	2,00000	,000000	3	
	,131	m	66	left	3,00000	,000000	2	
				Total	3,00000	,000000	2	
			Total	left	3,00000	,000000	2	
				Total	3,00000	,000000	2	
		Total	66	left	3,00000	,000000	2	
				Total	3,00000	,000000	2	
			Total	left	3,00000	,000000	2	
				Total	3,00000	,000000	2	
	,131	f	84	righ	3,00000	,000000	3	
				Total	3,00000	,000000	3	
			Total	righ	3,00000	,000000	3	
				Total	3,00000	,000000	3	
		Total	84	righ	3,00000	,000000	3	
				Total	3,00000	,000000	3	
			Total	righ	3,00000	,000000	3	
				Total	3,00000	,000000	3	
	,131	m	75	righ	1,00000	,000000	3	
				Total	1,00000	,000000	3	
			Total	righ	1,00000	,000000	3	
				Total	1,00000	,000000	3	
		Total	75	righ	1,00000	,000000	3	
				Total	1,00000	,000000	3	
			Total	righ	1,00000	,000000	3	
				Total	1,00000	,000000	3	
	,134	m	76	left	,00000	,000000	3	
				Total	,00000	,000000	3	
			Total	left	,00000	,000000	3	
				Total	,00000	,000000	3	
		Total	76	left	,00000	,000000	3	
				Total	,00000	,000000	3	
			Total	left	,00000	,000000	3	
				Total	,00000	,000000	3	
	,134	m	69	righ	6,00000	,000000	3	
				Total	6,00000	,000000	3	
			Total	righ	6,00000	,000000	3	
				Total	6,00000	,000000	3	
		Total	69	righ	6,00000	,000000	3	
				Total	6,00000	,000000	3	
			Total	righ	6,00000	,000000	3	
				Total	6,00000	,000000	3	
	,141	m	80	righ	4,00000	,000000	2	
				Total	4,00000	,000000	2	
			Total	righ	4,00000	,000000	2	
				Total	4,00000	,000000	2	
		Total	80	righ	4,00000	,000000	2	
				Total	4,00000	,000000	2	
			Total	righ	4,00000	,000000	2	
				Total	4,00000	,000000	2	
	,143	f	63	righ	8,50000	,000000	3	
				Total	8,50000	,000000	3	
			Total	righ	8,50000	,000000	3	
				Total	8,50000	,000000	3	
		Total	63	righ	8,50000	,000000	3	
				Total	8,50000	,000000	3	
			Total	righ	8,50000	,000000	3	
				Total	8,50000	,000000	3	
	,144	f	73	righ	3,00000	,000000	2	
				Total	3,00000	,000000	2	
			Total	righ	3,00000	,000000	2	
				Total	3,00000	,000000	2	
		Total	73	righ	3,00000	,000000	2	
				Total	3,00000	,000000	2	
			Total	righ	3,00000	,000000	2	
				Total	3,00000	,000000	2	
	,146	m	78	righ	5,00000	,000000	2	
				Total	5,00000	,000000	2	
			Total	righ	5,00000	,000000	2	
				Total	5,00000	,000000	2	
		Total	78	righ	5,00000	,000000	2	
				Total	5,00000	,000000	2	
			Total	righ	5,00000	,000000	2	
				Total	5,00000	,000000	2	
	,146	f	61	left	5,00000	,000000	3	
				Total	5,00000	,000000	3	
			Total	left	5,00000	,000000	3	
				Total	5,00000	,000000	3	
		Total	61	left	5,00000	,000000	3	
				Total	5,00000	,000000	3	
			Total	left	5,00000	,000000	3	
				Total	5,00000	,000000	3	
	,148	f	81	righ	7,00000	,000000	3	
				Total	7,00000	,000000	3	
			Total	righ	7,00000	,000000	3	
				Total	7,00000	,000000	3	
		Total	81	righ	7,00000	,000000	3	
				Total	7,00000	,000000	3	
			Total	righ	7,00000	,000000	3	
				Total	7,00000	,000000	3	
	,148	f	84	left	7,00000	,000000	3	
				Total	7,00000	,000000	3	
			Total	left	7,00000	,000000	3	
				Total	7,00000	,000000	3	
		Total	84	left	7,00000	,000000	3	
				Total	7,00000	,000000	3	
			Total	left	7,00000	,000000	3	
				Total	7,00000	,000000	3	
	,150	f	80	left	2,00000	,000000	3	
				Total	2,00000	,000000	3	
			Total	left	2,00000	,000000	3	
				Total	2,00000	,000000	3	
		Total	80	left	2,00000	,000000	3	
				Total	2,00000	,000000	3	
			Total	left	2,00000	,000000	3	
				Total	2,00000	,000000	3	
	,152	m	73	left	6,00000	,000000	3	
				Total	6,00000	,000000	3	
			Total	left	6,00000	,000000	3	
				Total	6,00000	,000000	3	
		Total	73	left	6,00000	,000000	3	
				Total	6,00000	,000000	3	
			Total	left	6,00000	,000000	3	
				Total	6,00000	,000000	3	
	,152	m	72	righ	8,40000	,000000	3	
				Total	8,40000	,000000	3	
			Total	righ	8,40000	,000000	3	
				Total	8,40000	,000000	3	
		Total	72	righ	8,40000	,000000	3	
				Total	8,40000	,000000	3	
			Total	righ	8,40000	,000000	3	
				Total	8,40000	,000000	3	
	,156	m	85	left	5,40000	,000000	3	
				Total	5,40000	,000000	3	
			Total	left	5,40000	,000000	3	
				Total	5,40000	,000000	3	
		Total	85	left	5,40000	,000000	3	
				Total	5,40000	,000000	3	
			Total	left	5,40000	,000000	3	
				Total	5,40000	,000000	3	
	,159	m	88	righ	,00000	,000000	3	
				Total	,00000	,000000	3	
			Total	righ	,00000	,000000	3	
				Total	,00000	,000000	3	
		Total	88	righ	,00000	,000000	3	
				Total	,00000	,000000	3	
			Total	righ	,00000	,000000	3	
				Total	,00000	,000000	3	
	,159	m	77	left	2,50000	,000000	3	
				Total	2,50000	,000000	3	
			Total	left	2,50000	,000000	3	
				Total	2,50000	,000000	3	
		Total	77	left	2,50000	,000000	3	
				Total	2,50000	,000000	3	
			Total	left	2,50000	,000000	3	
				Total	2,50000	,000000	3	
	,159	f	79	righ	,00000	,000000	3	
				Total	,00000	,000000	3	
			Total	righ	,00000	,000000	3	
				Total	,00000	,000000	3	
		Total	79	righ	,00000	,000000	3	
				Total	,00000	,000000	3	
			Total	righ	,00000	,000000	3	
				Total	,00000	,000000	3	
	,160	f	80	righ	1,00000	,000000	3	
				Total	1,00000	,000000	3	
			Total	righ	1,00000	,000000	3	
				Total	1,00000	,000000	3	
		Total	80	righ	1,00000	,000000	3	
				Total	1,00000	,000000	3	
			Total	righ	1,00000	,000000	3	
				Total	1,00000	,000000	3	
	,163	m	75	left	,00000	,000000	3	
				Total	,00000	,000000	3	
			Total	left	,00000	,000000	3	
				Total	,00000	,000000	3	
		Total	75	left	,00000	,000000	3	
				Total	,00000	,000000	3	
			Total	left	,00000	,000000	3	
				Total	,00000	,000000	3	
	,167	m	73	left	2,00000	1,000000	3	
				Total	2,00000	1,000000	3	
			Total	left	2,00000	1,000000	3	
				Total	2,00000	1,000000	3	
		Total	73	left	2,00000	1,000000	3	
				Total	2,00000	1,000000	3	
			Total	left	2,00000	1,000000	3	
				Total	2,00000	1,000000	3	
	,168	f	60	left	,00000	,000000	3	
				Total	,00000	,000000	3	
			Total	left	,00000	,000000	3	
				Total	,00000	,000000	3	
		Total	60	left	,00000	,000000	3	
				Total	,00000	,000000	3	
			Total	left	,00000	,000000	3	
				Total	,00000	,000000	3	
	,170	m	62	righ	9,00000	,000000	2	
				Total	9,00000	,000000	2	
			Total	righ	9,00000	,000000	2	
				Total	9,00000	,000000	2	
		Total	62	righ	9,00000	,000000	2	
				Total	9,00000	,000000	2	
			Total	righ	9,00000	,000000	2	
				Total	9,00000	,000000	2	
	,170	f	84	righ	3,00000	,000000	2	
				Total	3,00000	,000000	2	
			Total	righ	3,00000	,000000	2	
				Total	3,00000	,000000	2	
		Total	84	righ	3,00000	,000000	2	
				Total	3,00000	,000000	2	
			Total	righ	3,00000	,000000	2	
				Total	3,00000	,000000	2	
	,171	f	78	left	6,60000	,000000	3	
				Total	6,60000	,000000	3	
			Total	left	6,60000	,000000	3	
				Total	6,60000	,000000	3	
		Total	78	left	6,60000	,000000	3	
				Total	6,60000	,000000	3	
			Total	left	6,60000	,000000	3	
				Total	6,60000	,000000	3	
	,172	f	84	righ	4,00000	,000000	3	
				Total	4,00000	,000000	3	
			Total	righ	4,00000	,000000	3	
				Total	4,00000	,000000	3	
		Total	84	righ	4,00000	,000000	3	
				Total	4,00000	,000000	3	
			Total	righ	4,00000	,000000	3	
				Total	4,00000	,000000	3	
	,173	m	76	righ	5,00000	,000000	2	
				Total	5,00000	,000000	2	
			Total	righ	5,00000	,000000	2	
				Total	5,00000	,000000	2	
		Total	76	righ	5,00000	,000000	2	
				Total	5,00000	,000000	2	
			Total	righ	5,00000	,000000	2	
				Total	5,00000	,000000	2	
	,174	m	77	left	2,50000	,707107	2	
				Total	2,50000	,707107	2	
			Total	left	2,50000	,707107	2	
				Total	2,50000	,707107	2	
		Total	77	left	2,50000	,707107	2	
				Total	2,50000	,707107	2	
			Total	left	2,50000	,707107	2	
				Total	2,50000	,707107	2	
	,175	m	76	left	,00000	,000000	2	
				Total	,00000	,000000	2	
			Total	left	,00000	,000000	2	
				Total	,00000	,000000	2	
		Total	76	left	,00000	,000000	2	
				Total	,00000	,000000	2	
			Total	left	,00000	,000000	2	
				Total	,00000	,000000	2	
	,176	m	85	left	6,60000	,000000	3	
				Total	6,60000	,000000	3	
			Total	left	6,60000	,000000	3	
				Total	6,60000	,000000	3	
		Total	85	left	6,60000	,000000	3	
				Total	6,60000	,000000	3	
			Total	left	6,60000	,000000	3	
				Total	6,60000	,000000	3	
	,177	m	85	left	2,00000	1,000000	3	
				Total	2,00000	1,000000	3	
			Total	left	2,00000	1,000000	3	
				Total	2,00000	1,000000	3	
		Total	85	left	2,00000	1,000000	3	
				Total	2,00000	1,000000	3	
			Total	left	2,00000	1,000000	3	
				Total	2,00000	1,000000	3	
	,180	f	64	righ	2,50000	,707107	2	
				Total	2,50000	,707107	2	
			Total	righ	2,50000	,707107	2	
				Total	2,50000	,707107	2	
		Total	64	righ	2,50000	,707107	2	
				Total	2,50000	,707107	2	
			Total	righ	2,50000	,707107	2	
				Total	2,50000	,707107	2	
	,181	f	80	left	2,40000	,000000	2	
				Total	2,40000	,000000	2	
			Total	left	2,40000	,000000	2	
				Total	2,40000	,000000	2	
		Total	80	left	2,40000	,000000	2	
				Total	2,40000	,000000	2	
			Total	left	2,40000	,000000	2	
				Total	2,40000	,000000	2	
	,183	f	85	righ	6,30000	,000000	3	
				Total	6,30000	,000000	3	
			Total	righ	6,30000	,000000	3	
				Total	6,30000	,000000	3	
		Total	85	righ	6,30000	,000000	3	
				Total	6,30000	,000000	3	
			Total	righ	6,30000	,000000	3	
				Total	6,30000	,000000	3	
	,185	m	71	righ	1,00000	,000000	3	
				Total	1,00000	,000000	3	
			Total	righ	1,00000	,000000	3	
				Total	1,00000	,000000	3	
		Total	71	righ	1,00000	,000000	3	
				Total	1,00000	,000000	3	
			Total	righ	1,00000	,000000	3	
				Total	1,00000	,000000	3	
	,188	m	78	righ	,00000	,000000	3	
				Total	,00000	,000000	3	
			Total	righ	,00000	,000000	3	
				Total	,00000	,000000	3	
		Total	78	righ	,00000	,000000	3	
				Total	,00000	,000000	3	
			Total	righ	,00000	,000000	3	
				Total	,00000	,000000	3	
	,188	m	81	righ	3,40000	,000000	3	
				Total	3,40000	,000000	3	
			Total	righ	3,40000	,000000	3	
				Total	3,40000	,000000	3	
		Total	81	righ	3,40000	,000000	3	
				Total	3,40000	,000000	3	
			Total	righ	3,40000	,000000	3	
				Total	3,40000	,000000	3	
	,189	m	85	righ	4,00000	,000000	3	
				Total	4,00000	,000000	3	
			Total	righ	4,00000	,000000	3	
				Total	4,00000	,000000	3	
		Total	85	righ	4,00000	,000000	3	
				Total	4,00000	,000000	3	
			Total	righ	4,00000	,000000	3	
				Total	4,00000	,000000	3	
	,190	f	81	righ	6,20000	,000000	2	
				Total	6,20000	,000000	2	
			Total	righ	6,20000	,000000	2	
				Total	6,20000	,000000	2	
		Total	81	righ	6,20000	,000000	2	
				Total	6,20000	,000000	2	
			Total	righ	6,20000	,000000	2	
				Total	6,20000	,000000	2	
	,191	f	79	left	7,00000	,000000	3	
				Total	7,00000	,000000	3	
			Total	left	7,00000	,000000	3	
				Total	7,00000	,000000	3	
		Total	79	left	7,00000	,000000	3	
				Total	7,00000	,000000	3	
			Total	left	7,00000	,000000	3	
				Total	7,00000	,000000	3	
	,192	f	78	left	4,00000	,000000	2	
				Total	4,00000	,000000	2	
			Total	left	4,00000	,000000	2	
				Total	4,00000	,000000	2	
		Total	78	left	4,00000	,000000	2	
				Total	4,00000	,000000	2	
			Total	left	4,00000	,000000	2	
				Total	4,00000	,000000	2	
	,192	f	71	left	2,00000	,000000	3	
				Total	2,00000	,000000	3	
			Total	left	2,00000	,000000	3	
				Total	2,00000	,000000	3	
		Total	71	left	2,00000	,000000	3	
				Total	2,00000	,000000	3	
			Total	left	2,00000	,000000	3	
				Total	2,00000	,000000	3	
	,194	m	85	left	2,90000	,000000	2	
				Total	2,90000	,000000	2	
			Total	left	2,90000	,000000	2	
				Total	2,90000	,000000	2	
		Total	85	left	2,90000	,000000	2	
				Total	2,90000	,000000	2	
			Total	left	2,90000	,000000	2	
				Total	2,90000	,000000	2	
	,198	m	84	righ	,00000	,000000	2	
				Total	,00000	,000000	2	
			Total	righ	,00000	,000000	2	
				Total	,00000	,000000	2	
		Total	84	righ	,00000	,000000	2	
				Total	,00000	,000000	2	
			Total	righ	,00000	,000000	2	
				Total	,00000	,000000	2	
	,198	m	90	left	,00000	,000000	2	
				Total	,00000	,000000	2	
			Total	left	,00000	,000000	2	
				Total	,00000	,000000	2	
		Total	90	left	,00000	,000000	2	
				Total	,00000	,000000	2	
			Total	left	,00000	,000000	2	
				Total	,00000	,000000	2	
	,200	m	80	left	1,00000	,000000	3	
				Total	1,00000	,000000	3	
			Total	left	1,00000	,000000	3	
				Total	1,00000	,000000	3	
		Total	80	left	1,00000	,000000	3	
				Total	1,00000	,000000	3	
			Total	left	1,00000	,000000	3	
				Total	1,00000	,000000	3	
	,200	m	78	left	4,20000	,000000	3	
				Total	4,20000	,000000	3	
			Total	left	4,20000	,000000	3	
				Total	4,20000	,000000	3	
		Total	78	left	4,20000	,000000	3	
				Total	4,20000	,000000	3	
			Total	left	4,20000	,000000	3	
				Total	4,20000	,000000	3	
	,201	m	96	left	,00000	,000000	3	
				Total	,00000	,000000	3	
			Total	left	,00000	,000000	3	
				Total	,00000	,000000	3	
		Total	96	left	,00000	,000000	3	
				Total	,00000	,000000	3	
			Total	left	,00000	,000000	3	
				Total	,00000	,000000	3	
	,207	m	83	left	6,00000	,000000	3	
				Total	6,00000	,000000	3	
			Total	left	6,00000	,000000	3	
				Total	6,00000	,000000	3	
		Total	83	left	6,00000	,000000	3	
				Total	6,00000	,000000	3	
			Total	left	6,00000	,000000	3	
				Total	6,00000	,000000	3	
	,208	f	83	righ	5,30000	,000000	3	
				Total	5,30000	,000000	3	
			Total	righ	5,30000	,000000	3	
				Total	5,30000	,000000	3	
		Total	83	righ	5,30000	,000000	3	
				Total	5,30000	,000000	3	
			Total	righ	5,30000	,000000	3	
				Total	5,30000	,000000	3	
	,210	m	82	left	,00000	,000000	3	
				Total	,00000	,000000	3	
			Total	left	,00000	,000000	3	
				Total	,00000	,000000	3	
		Total	82	left	,00000	,000000	3	
				Total	,00000	,000000	3	
			Total	left	,00000	,000000	3	
				Total	,00000	,000000	3	
	,211	m	85	left	5,40000	,000000	3	
				Total	5,40000	,000000	3	
			Total	left	5,40000	,000000	3	
				Total	5,40000	,000000	3	
		Total	85	left	5,40000	,000000	3	
				Total	5,40000	,000000	3	
			Total	left	5,40000	,000000	3	
				Total	5,40000	,000000	3	
	,213	m	82	left	3,00000	,000000	3	
				Total	3,00000	,000000	3	
			Total	left	3,00000	,000000	3	
				Total	3,00000	,000000	3	
		Total	82	left	3,00000	,000000	3	
				Total	3,00000	,000000	3	
			Total	left	3,00000	,000000	3	
				Total	3,00000	,000000	3	
	,213	m	82	righ	5,00000	,000000	2	
				Total	5,00000	,000000	2	
			Total	righ	5,00000	,000000	2	
				Total	5,00000	,000000	2	
		Total	82	righ	5,00000	,000000	2	
				Total	5,00000	,000000	2	
			Total	righ	5,00000	,000000	2	
				Total	5,00000	,000000	2	
	,214	m	85	righ	4,50000	,000000	3	
				Total	4,50000	,000000	3	
			Total	righ	4,50000	,000000	3	
				Total	4,50000	,000000	3	
		Total	85	righ	4,50000	,000000	3	
				Total	4,50000	,000000	3	
			Total	righ	4,50000	,000000	3	
				Total	4,50000	,000000	3	
	,219	f	71	righ	3,00000	,000000	3	
				Total	3,00000	,000000	3	
			Total	righ	3,00000	,000000	3	
				Total	3,00000	,000000	3	
		Total	71	righ	3,00000	,000000	3	
				Total	3,00000	,000000	3	
			Total	righ	3,00000	,000000	3	
				Total	3,00000	,000000	3	
	,221	f	74	righ	2,50000	,707107	2	
				Total	2,50000	,707107	2	
			Total	righ	2,50000	,707107	2	
				Total	2,50000	,707107	2	
		Total	74	righ	2,50000	,707107	2	
				Total	2,50000	,707107	2	
			Total	righ	2,50000	,707107	2	
				Total	2,50000	,707107	2	
	,224	m	85	righ	3,00000	,000000	2	
				Total	3,00000	,000000	2	
			Total	righ	3,00000	,000000	2	
				Total	3,00000	,000000	2	
		Total	85	righ	3,00000	,000000	2	
				Total	3,00000	,000000	2	
			Total	righ	3,00000	,000000	2	
				Total	3,00000	,000000	2	
	,224	f	97	righ	1,00000	,000000	3	
				Total	1,00000	,000000	3	
			Total	righ	1,00000	,000000	3	
				Total	1,00000	,000000	3	
		Total	97	righ	1,00000	,000000	3	
				Total	1,00000	,000000	3	
			Total	righ	1,00000	,000000	3	
				Total	1,00000	,000000	3	
	,226	f	82	left	,00000	,000000	3	
				Total	,00000	,000000	3	
			Total	left	,00000	,000000	3	
				Total	,00000	,000000	3	
		Total	82	left	,00000	,000000	3	
				Total	,00000	,000000	3	
			Total	left	,00000	,000000	3	
				Total	,00000	,000000	3	
	,226	f	83	righ	8,70000	,000000	3	
				Total	8,70000	,000000	3	
			Total	righ	8,70000	,000000	3	
				Total	8,70000	,000000	3	
		Total	83	righ	8,70000	,000000	3	
				Total	8,70000	,000000	3	
			Total	righ	8,70000	,000000	3	
				Total	8,70000	,000000	3	
	,228	m	87	righ	3,00000	,000000	3	
				Total	3,00000	,000000	3	
			Total	righ	3,00000	,000000	3	
				Total	3,00000	,000000	3	
		Total	87	righ	3,00000	,000000	3	
				Total	3,00000	,000000	3	
			Total	righ	3,00000	,000000	3	
				Total	3,00000	,000000	3	
	,254	m	85	left	2,00000	,000000	3	
				Total	2,00000	,000000	3	
			Total	left	2,00000	,000000	3	
				Total	2,00000	,000000	3	
		Total	85	left	2,00000	,000000	3	
				Total	2,00000	,000000	3	
			Total	left	2,00000	,000000	3	
				Total	2,00000	,000000	3	
	,265	m	85	righ	1,00000	,000000	3	
				Total	1,00000	,000000	3	
			Total	righ	1,00000	,000000	3	
				Total	1,00000	,000000	3	
		Total	85	righ	1,00000	,000000	3	
				Total	1,00000	,000000	3	
			Total	righ	1,00000	,000000	3	
				Total	1,00000	,000000	3	
	Total	f	60	left	,00000	,000000	3	
				Total	,00000	,000000	3	
			61	left	5,00000	,000000	3	
				Total	5,00000	,000000	3	
			63	righ	8,50000	,000000	3	
				Total	8,50000	,000000	3	
			64	righ	2,50000	,707107	2	
				Total	2,50000	,707107	2	
			71	left	2,00000	,000000	3	
				righ	3,00000	,000000	3	
				Total	2,50000	,547723	6	
			73	righ	3,00000	,000000	2	
				Total	3,00000	,000000	2	
			74	righ	2,20000	,447214	5	
				Total	2,20000	,447214	5	
			78	left	5,56000	1,424079	5	
				Total	5,56000	1,424079	5	
			79	left	7,00000	,000000	3	
				righ	,00000	,000000	3	
				Total	3,50000	3,834058	6	
			80	left	2,16000	,219089	5	
				righ	1,00000	,000000	3	
				Total	1,72500	,622782	8	
			81	righ	6,68000	,438178	5	
				Total	6,68000	,438178	5	
			82	left	,00000	,000000	3	
				Total	,00000	,000000	3	
			83	righ	7,00000	1,862257	6	
				Total	7,00000	1,862257	6	
			84	left	7,00000	,000000	3	
				righ	3,37500	,517549	8	
				Total	4,36364	1,747726	11	
			85	righ	6,30000	,000000	3	
				Total	6,30000	,000000	3	
			97	righ	1,00000	,000000	3	
				Total	1,00000	,000000	3	
			Total	left	3,62857	2,711684	28	
				righ	3,99565	2,660990	46	
				Total	3,85676	2,667721	74	
		m	62	righ	9,00000	,000000	2	
				Total	9,00000	,000000	2	
			63	righ	3,00000	,000000	2	
				Total	3,00000	,000000	2	
			66	left	3,00000	,000000	2	
				Total	3,00000	,000000	2	
			69	righ	6,00000	,000000	3	
				Total	6,00000	,000000	3	
			70	righ	3,00000	,000000	3	
				Total	3,00000	,000000	3	
			71	righ	1,00000	,000000	3	
				Total	1,00000	,000000	3	
			72	righ	5,20000	3,505424	6	
				Total	5,20000	3,505424	6	
			73	left	3,66667	1,870829	9	
				Total	3,66667	1,870829	9	
			75	left	,00000	,000000	3	
				righ	1,00000	,000000	3	
				Total	,50000	,547723	6	
			76	left	,00000	,000000	5	
				righ	5,00000	,000000	2	
				Total	1,42857	2,439750	7	
			77	left	2,50000	,353553	5	
				Total	2,50000	,353553	5	
			78	left	4,20000	,000000	3	
				righ	2,00000	2,738613	5	
				Total	2,82500	2,362656	8	
			80	left	1,00000	,000000	3	
				righ	4,00000	,000000	2	
				Total	2,20000	1,643168	5	
			81	righ	3,40000	,000000	3	
				Total	3,40000	,000000	3	
			82	left	1,50000	1,643168	6	
				righ	5,00000	,000000	2	
				Total	2,37500	2,133910	8	
			83	left	6,00000	,000000	3	
				Total	6,00000	,000000	3	
			84	righ	,00000	,000000	2	
				Total	,00000	,000000	2	
			85	left	4,11765	1,940114	17	
				righ	3,13636	1,467837	11	
				Total	3,73214	1,807403	28	
			87	righ	3,00000	,000000	3	
				Total	3,00000	,000000	3	
			88	righ	,00000	,000000	3	
				Total	,00000	,000000	3	
			90	left	,00000	,000000	2	
				Total	,00000	,000000	2	
			96	left	,00000	,000000	3	
				Total	,00000	,000000	3	
			Total	left	2,69016	2,223639	61	
				righ	3,27091	2,470843	55	
				Total	2,96552	2,351876	116	
		Total	60	left	,00000	,000000	3	
				Total	,00000	,000000	3	
			61	left	5,00000	,000000	3	
				Total	5,00000	,000000	3	
			62	righ	9,00000	,000000	2	
				Total	9,00000	,000000	2	
			63	righ	6,30000	3,012474	5	
				Total	6,30000	3,012474	5	
			64	righ	2,50000	,707107	2	
				Total	2,50000	,707107	2	
			66	left	3,00000	,000000	2	
				Total	3,00000	,000000	2	
			69	righ	6,00000	,000000	3	
				Total	6,00000	,000000	3	
			70	righ	3,00000	,000000	3	
				Total	3,00000	,000000	3	
			71	left	2,00000	,000000	3	
				righ	2,00000	1,095445	6	
				Total	2,00000	,866025	9	
			72	righ	5,20000	3,505424	6	
				Total	5,20000	3,505424	6	
			73	left	3,66667	1,870829	9	
				righ	3,00000	,000000	2	
				Total	3,54545	1,694912	11	
			74	righ	2,20000	,447214	5	
				Total	2,20000	,447214	5	
			75	left	,00000	,000000	3	
				righ	1,00000	,000000	3	
				Total	,50000	,547723	6	
			76	left	,00000	,000000	5	
				righ	5,00000	,000000	2	
				Total	1,42857	2,439750	7	
			77	left	2,50000	,353553	5	
				Total	2,50000	,353553	5	
			78	left	5,05000	1,286190	8	
				righ	2,00000	2,738613	5	
				Total	3,87692	2,418730	13	
			79	left	7,00000	,000000	3	
				righ	,00000	,000000	3	
				Total	3,50000	3,834058	6	
			80	left	1,72500	,622782	8	
				righ	2,20000	1,643168	5	
				Total	1,90769	1,088165	13	
			81	righ	5,45000	1,729575	8	
				Total	5,45000	1,729575	8	
			82	left	1,00000	1,500000	9	
				righ	5,00000	,000000	2	
				Total	1,72727	2,101947	11	
			83	left	6,00000	,000000	3	
				righ	7,00000	1,862257	6	
				Total	6,66667	1,554831	9	
			84	left	7,00000	,000000	3	
				righ	2,70000	1,494434	10	
				Total	3,69231	2,287087	13	
			85	left	4,11765	1,940114	17	
				righ	3,81429	1,863354	14	
				Total	3,98065	1,880323	31	
			87	righ	3,00000	,000000	3	
				Total	3,00000	,000000	3	
			88	righ	,00000	,000000	3	
				Total	,00000	,000000	3	
			90	left	,00000	,000000	2	
				Total	,00000	,000000	2	
			96	left	,00000	,000000	3	
				Total	,00000	,000000	3	
			97	righ	1,00000	,000000	3	
				Total	1,00000	,000000	3	
			Total	left	2,98539	2,412352	89	
				righ	3,60099	2,571906	101	
				Total	3,31263	2,510833	190	
Shift post 90	,100	m	63	righ	1,50	2,121	2	
				Total	1,50	2,121	2	
			Total	righ	1,50	2,121	2	
				Total	1,50	2,121	2	
		Total	63	righ	1,50	2,121	2	
				Total	1,50	2,121	2	
			Total	righ	1,50	2,121	2	
				Total	1,50	2,121	2	
	,111	m	70	righ	1,33	1,528	3	
				Total	1,33	1,528	3	
			Total	righ	1,33	1,528	3	
				Total	1,33	1,528	3	
		Total	70	righ	1,33	1,528	3	
				Total	1,33	1,528	3	
			Total	righ	1,33	1,528	3	
				Total	1,33	1,528	3	
	,120	m	72	righ	1,00	,000	3	
				Total	1,00	,000	3	
			Total	righ	1,00	,000	3	
				Total	1,00	,000	3	
		Total	72	righ	1,00	,000	3	
				Total	1,00	,000	3	
			Total	righ	1,00	,000	3	
				Total	1,00	,000	3	
	,125	m	73	left	1,33	1,528	3	
				Total	1,33	1,528	3	
			Total	left	1,33	1,528	3	
				Total	1,33	1,528	3	
		Total	73	left	1,33	1,528	3	
				Total	1,33	1,528	3	
			Total	left	1,33	1,528	3	
				Total	1,33	1,528	3	
	,127	f	74	righ	2,33	2,082	3	
				Total	2,33	2,082	3	
			Total	righ	2,33	2,082	3	
				Total	2,33	2,082	3	
		Total	74	righ	2,33	2,082	3	
				Total	2,33	2,082	3	
			Total	righ	2,33	2,082	3	
				Total	2,33	2,082	3	
	,131	m	66	left	2,00	1,414	2	
				Total	2,00	1,414	2	
			Total	left	2,00	1,414	2	
				Total	2,00	1,414	2	
		Total	66	left	2,00	1,414	2	
				Total	2,00	1,414	2	
			Total	left	2,00	1,414	2	
				Total	2,00	1,414	2	
	,131	f	84	righ	1,00	,000	3	
				Total	1,00	,000	3	
			Total	righ	1,00	,000	3	
				Total	1,00	,000	3	
		Total	84	righ	1,00	,000	3	
				Total	1,00	,000	3	
			Total	righ	1,00	,000	3	
				Total	1,00	,000	3	
	,131	m	75	righ	2,33	2,082	3	
				Total	2,33	2,082	3	
			Total	righ	2,33	2,082	3	
				Total	2,33	2,082	3	
		Total	75	righ	2,33	2,082	3	
				Total	2,33	2,082	3	
			Total	righ	2,33	2,082	3	
				Total	2,33	2,082	3	
	,134	m	76	left	2,00	1,732	3	
				Total	2,00	1,732	3	
			Total	left	2,00	1,732	3	
				Total	2,00	1,732	3	
		Total	76	left	2,00	1,732	3	
				Total	2,00	1,732	3	
			Total	left	2,00	1,732	3	
				Total	2,00	1,732	3	
	,134	m	69	righ	3,00	,000	3	
				Total	3,00	,000	3	
			Total	righ	3,00	,000	3	
				Total	3,00	,000	3	
		Total	69	righ	3,00	,000	3	
				Total	3,00	,000	3	
			Total	righ	3,00	,000	3	
				Total	3,00	,000	3	
	,141	m	80	righ	2,00	1,414	2	
				Total	2,00	1,414	2	
			Total	righ	2,00	1,414	2	
				Total	2,00	1,414	2	
		Total	80	righ	2,00	1,414	2	
				Total	2,00	1,414	2	
			Total	righ	2,00	1,414	2	
				Total	2,00	1,414	2	
	,143	f	63	righ	1,00	,000	3	
				Total	1,00	,000	3	
			Total	righ	1,00	,000	3	
				Total	1,00	,000	3	
		Total	63	righ	1,00	,000	3	
				Total	1,00	,000	3	
			Total	righ	1,00	,000	3	
				Total	1,00	,000	3	
	,144	f	73	righ	3,00	,000	2	
				Total	3,00	,000	2	
			Total	righ	3,00	,000	2	
				Total	3,00	,000	2	
		Total	73	righ	3,00	,000	2	
				Total	3,00	,000	2	
			Total	righ	3,00	,000	2	
				Total	3,00	,000	2	
	,146	m	78	righ	3,00	,000	2	
				Total	3,00	,000	2	
			Total	righ	3,00	,000	2	
				Total	3,00	,000	2	
		Total	78	righ	3,00	,000	2	
				Total	3,00	,000	2	
			Total	righ	3,00	,000	2	
				Total	3,00	,000	2	
	,146	f	61	left	1,00	,000	3	
				Total	1,00	,000	3	
			Total	left	1,00	,000	3	
				Total	1,00	,000	3	
		Total	61	left	1,00	,000	3	
				Total	1,00	,000	3	
			Total	left	1,00	,000	3	
				Total	1,00	,000	3	
	,148	f	81	righ	2,33	1,155	3	
				Total	2,33	1,155	3	
			Total	righ	2,33	1,155	3	
				Total	2,33	1,155	3	
		Total	81	righ	2,33	1,155	3	
				Total	2,33	1,155	3	
			Total	righ	2,33	1,155	3	
				Total	2,33	1,155	3	
	,148	f	84	left	3,00	,000	3	
				Total	3,00	,000	3	
			Total	left	3,00	,000	3	
				Total	3,00	,000	3	
		Total	84	left	3,00	,000	3	
				Total	3,00	,000	3	
			Total	left	3,00	,000	3	
				Total	3,00	,000	3	
	,150	f	80	left	1,00	,000	3	
				Total	1,00	,000	3	
			Total	left	1,00	,000	3	
				Total	1,00	,000	3	
		Total	80	left	1,00	,000	3	
				Total	1,00	,000	3	
			Total	left	1,00	,000	3	
				Total	1,00	,000	3	
	,152	m	73	left	1,67	1,155	3	
				Total	1,67	1,155	3	
			Total	left	1,67	1,155	3	
				Total	1,67	1,155	3	
		Total	73	left	1,67	1,155	3	
				Total	1,67	1,155	3	
			Total	left	1,67	1,155	3	
				Total	1,67	1,155	3	
	,152	m	72	righ	4,00	,000	3	
				Total	4,00	,000	3	
			Total	righ	4,00	,000	3	
				Total	4,00	,000	3	
		Total	72	righ	4,00	,000	3	
				Total	4,00	,000	3	
			Total	righ	4,00	,000	3	
				Total	4,00	,000	3	
	,156	m	85	left	1,67	1,528	3	
				Total	1,67	1,528	3	
			Total	left	1,67	1,528	3	
				Total	1,67	1,528	3	
		Total	85	left	1,67	1,528	3	
				Total	1,67	1,528	3	
			Total	left	1,67	1,528	3	
				Total	1,67	1,528	3	
	,159	m	88	righ	2,33	2,082	3	
				Total	2,33	2,082	3	
			Total	righ	2,33	2,082	3	
				Total	2,33	2,082	3	
		Total	88	righ	2,33	2,082	3	
				Total	2,33	2,082	3	
			Total	righ	2,33	2,082	3	
				Total	2,33	2,082	3	
	,159	m	77	left	1,67	1,528	3	
				Total	1,67	1,528	3	
			Total	left	1,67	1,528	3	
				Total	1,67	1,528	3	
		Total	77	left	1,67	1,528	3	
				Total	1,67	1,528	3	
			Total	left	1,67	1,528	3	
				Total	1,67	1,528	3	
	,159	f	79	righ	1,67	1,155	3	
				Total	1,67	1,155	3	
			Total	righ	1,67	1,155	3	
				Total	1,67	1,155	3	
		Total	79	righ	1,67	1,155	3	
				Total	1,67	1,155	3	
			Total	righ	1,67	1,155	3	
				Total	1,67	1,155	3	
	,160	f	80	righ	2,33	1,528	3	
				Total	2,33	1,528	3	
			Total	righ	2,33	1,528	3	
				Total	2,33	1,528	3	
		Total	80	righ	2,33	1,528	3	
				Total	2,33	1,528	3	
			Total	righ	2,33	1,528	3	
				Total	2,33	1,528	3	
	,163	m	75	left	1,00	,000	3	
				Total	1,00	,000	3	
			Total	left	1,00	,000	3	
				Total	1,00	,000	3	
		Total	75	left	1,00	,000	3	
				Total	1,00	,000	3	
			Total	left	1,00	,000	3	
				Total	1,00	,000	3	
	,167	m	73	left	1,00	1,732	3	
				Total	1,00	1,732	3	
			Total	left	1,00	1,732	3	
				Total	1,00	1,732	3	
		Total	73	left	1,00	1,732	3	
				Total	1,00	1,732	3	
			Total	left	1,00	1,732	3	
				Total	1,00	1,732	3	
	,168	f	60	left	2,33	1,155	3	
				Total	2,33	1,155	3	
			Total	left	2,33	1,155	3	
				Total	2,33	1,155	3	
		Total	60	left	2,33	1,155	3	
				Total	2,33	1,155	3	
			Total	left	2,33	1,155	3	
				Total	2,33	1,155	3	
	,170	m	62	righ	1,50	,707	2	
				Total	1,50	,707	2	
			Total	righ	1,50	,707	2	
				Total	1,50	,707	2	
		Total	62	righ	1,50	,707	2	
				Total	1,50	,707	2	
			Total	righ	1,50	,707	2	
				Total	1,50	,707	2	
	,170	f	84	righ	1,00	,000	2	
				Total	1,00	,000	2	
			Total	righ	1,00	,000	2	
				Total	1,00	,000	2	
		Total	84	righ	1,00	,000	2	
				Total	1,00	,000	2	
			Total	righ	1,00	,000	2	
				Total	1,00	,000	2	
	,171	f	78	left	3,00	,000	3	
				Total	3,00	,000	3	
			Total	left	3,00	,000	3	
				Total	3,00	,000	3	
		Total	78	left	3,00	,000	3	
				Total	3,00	,000	3	
			Total	left	3,00	,000	3	
				Total	3,00	,000	3	
	,172	f	84	righ	1,00	,000	3	
				Total	1,00	,000	3	
			Total	righ	1,00	,000	3	
				Total	1,00	,000	3	
		Total	84	righ	1,00	,000	3	
				Total	1,00	,000	3	
			Total	righ	1,00	,000	3	
				Total	1,00	,000	3	
	,173	m	76	righ	1,50	,707	2	
				Total	1,50	,707	2	
			Total	righ	1,50	,707	2	
				Total	1,50	,707	2	
		Total	76	righ	1,50	,707	2	
				Total	1,50	,707	2	
			Total	righ	1,50	,707	2	
				Total	1,50	,707	2	
	,174	m	77	left	1,50	2,121	2	
				Total	1,50	2,121	2	
			Total	left	1,50	2,121	2	
				Total	1,50	2,121	2	
		Total	77	left	1,50	2,121	2	
				Total	1,50	2,121	2	
			Total	left	1,50	2,121	2	
				Total	1,50	2,121	2	
	,175	m	76	left	1,50	2,121	2	
				Total	1,50	2,121	2	
			Total	left	1,50	2,121	2	
				Total	1,50	2,121	2	
		Total	76	left	1,50	2,121	2	
				Total	1,50	2,121	2	
			Total	left	1,50	2,121	2	
				Total	1,50	2,121	2	
	,176	m	85	left	3,00	,000	3	
				Total	3,00	,000	3	
			Total	left	3,00	,000	3	
				Total	3,00	,000	3	
		Total	85	left	3,00	,000	3	
				Total	3,00	,000	3	
			Total	left	3,00	,000	3	
				Total	3,00	,000	3	
	,177	m	85	left	,67	,577	3	
				Total	,67	,577	3	
			Total	left	,67	,577	3	
				Total	,67	,577	3	
		Total	85	left	,67	,577	3	
				Total	,67	,577	3	
			Total	left	,67	,577	3	
				Total	,67	,577	3	
	,180	f	64	righ	1,50	,707	2	
				Total	1,50	,707	2	
			Total	righ	1,50	,707	2	
				Total	1,50	,707	2	
		Total	64	righ	1,50	,707	2	
				Total	1,50	,707	2	
			Total	righ	1,50	,707	2	
				Total	1,50	,707	2	
	,181	f	80	left	,50	,707	2	
				Total	,50	,707	2	
			Total	left	,50	,707	2	
				Total	,50	,707	2	
		Total	80	left	,50	,707	2	
				Total	,50	,707	2	
			Total	left	,50	,707	2	
				Total	,50	,707	2	
	,183	f	85	righ	2,00	,000	3	
				Total	2,00	,000	3	
			Total	righ	2,00	,000	3	
				Total	2,00	,000	3	
		Total	85	righ	2,00	,000	3	
				Total	2,00	,000	3	
			Total	righ	2,00	,000	3	
				Total	2,00	,000	3	
	,185	m	71	righ	2,00	1,732	3	
				Total	2,00	1,732	3	
			Total	righ	2,00	1,732	3	
				Total	2,00	1,732	3	
		Total	71	righ	2,00	1,732	3	
				Total	2,00	1,732	3	
			Total	righ	2,00	1,732	3	
				Total	2,00	1,732	3	
	,188	m	78	righ	1,33	1,528	3	
				Total	1,33	1,528	3	
			Total	righ	1,33	1,528	3	
				Total	1,33	1,528	3	
		Total	78	righ	1,33	1,528	3	
				Total	1,33	1,528	3	
			Total	righ	1,33	1,528	3	
				Total	1,33	1,528	3	
	,188	m	81	righ	2,33	1,155	3	
				Total	2,33	1,155	3	
			Total	righ	2,33	1,155	3	
				Total	2,33	1,155	3	
		Total	81	righ	2,33	1,155	3	
				Total	2,33	1,155	3	
			Total	righ	2,33	1,155	3	
				Total	2,33	1,155	3	
	,189	m	85	righ	2,33	1,155	3	
				Total	2,33	1,155	3	
			Total	righ	2,33	1,155	3	
				Total	2,33	1,155	3	
		Total	85	righ	2,33	1,155	3	
				Total	2,33	1,155	3	
			Total	righ	2,33	1,155	3	
				Total	2,33	1,155	3	
	,190	f	81	righ	,50	,707	2	
				Total	,50	,707	2	
			Total	righ	,50	,707	2	
				Total	,50	,707	2	
		Total	81	righ	,50	,707	2	
				Total	,50	,707	2	
			Total	righ	,50	,707	2	
				Total	,50	,707	2	
	,191	f	79	left	4,00	,000	3	
				Total	4,00	,000	3	
			Total	left	4,00	,000	3	
				Total	4,00	,000	3	
		Total	79	left	4,00	,000	3	
				Total	4,00	,000	3	
			Total	left	4,00	,000	3	
				Total	4,00	,000	3	
	,192	f	78	left	1,00	,000	2	
				Total	1,00	,000	2	
			Total	left	1,00	,000	2	
				Total	1,00	,000	2	
		Total	78	left	1,00	,000	2	
				Total	1,00	,000	2	
			Total	left	1,00	,000	2	
				Total	1,00	,000	2	
	,192	f	71	left	2,00	1,732	3	
				Total	2,00	1,732	3	
			Total	left	2,00	1,732	3	
				Total	2,00	1,732	3	
		Total	71	left	2,00	1,732	3	
				Total	2,00	1,732	3	
			Total	left	2,00	1,732	3	
				Total	2,00	1,732	3	
	,194	m	85	left	2,00	1,414	2	
				Total	2,00	1,414	2	
			Total	left	2,00	1,414	2	
				Total	2,00	1,414	2	
		Total	85	left	2,00	1,414	2	
				Total	2,00	1,414	2	
			Total	left	2,00	1,414	2	
				Total	2,00	1,414	2	
	,198	m	84	righ	2,00	1,414	2	
				Total	2,00	1,414	2	
			Total	righ	2,00	1,414	2	
				Total	2,00	1,414	2	
		Total	84	righ	2,00	1,414	2	
				Total	2,00	1,414	2	
			Total	righ	2,00	1,414	2	
				Total	2,00	1,414	2	
	,198	m	90	left	1,50	2,121	2	
				Total	1,50	2,121	2	
			Total	left	1,50	2,121	2	
				Total	1,50	2,121	2	
		Total	90	left	1,50	2,121	2	
				Total	1,50	2,121	2	
			Total	left	1,50	2,121	2	
				Total	1,50	2,121	2	
	,200	m	80	left	1,33	1,528	3	
				Total	1,33	1,528	3	
			Total	left	1,33	1,528	3	
				Total	1,33	1,528	3	
		Total	80	left	1,33	1,528	3	
				Total	1,33	1,528	3	
			Total	left	1,33	1,528	3	
				Total	1,33	1,528	3	
	,200	m	78	left	1,67	1,155	3	
				Total	1,67	1,155	3	
			Total	left	1,67	1,155	3	
				Total	1,67	1,155	3	
		Total	78	left	1,67	1,155	3	
				Total	1,67	1,155	3	
			Total	left	1,67	1,155	3	
				Total	1,67	1,155	3	
	,201	m	96	left	,33	,577	3	
				Total	,33	,577	3	
			Total	left	,33	,577	3	
				Total	,33	,577	3	
		Total	96	left	,33	,577	3	
				Total	,33	,577	3	
			Total	left	,33	,577	3	
				Total	,33	,577	3	
	,207	m	83	left	3,00	,000	3	
				Total	3,00	,000	3	
			Total	left	3,00	,000	3	
				Total	3,00	,000	3	
		Total	83	left	3,00	,000	3	
				Total	3,00	,000	3	
			Total	left	3,00	,000	3	
				Total	3,00	,000	3	
	,208	f	83	righ	2,67	1,528	3	
				Total	2,67	1,528	3	
			Total	righ	2,67	1,528	3	
				Total	2,67	1,528	3	
		Total	83	righ	2,67	1,528	3	
				Total	2,67	1,528	3	
			Total	righ	2,67	1,528	3	
				Total	2,67	1,528	3	
	,210	m	82	left	1,33	1,528	3	
				Total	1,33	1,528	3	
			Total	left	1,33	1,528	3	
				Total	1,33	1,528	3	
		Total	82	left	1,33	1,528	3	
				Total	1,33	1,528	3	
			Total	left	1,33	1,528	3	
				Total	1,33	1,528	3	
	,211	m	85	left	3,00	,000	3	
				Total	3,00	,000	3	
			Total	left	3,00	,000	3	
				Total	3,00	,000	3	
		Total	85	left	3,00	,000	3	
				Total	3,00	,000	3	
			Total	left	3,00	,000	3	
				Total	3,00	,000	3	
	,213	m	82	left	1,67	1,155	3	
				Total	1,67	1,155	3	
			Total	left	1,67	1,155	3	
				Total	1,67	1,155	3	
		Total	82	left	1,67	1,155	3	
				Total	1,67	1,155	3	
			Total	left	1,67	1,155	3	
				Total	1,67	1,155	3	
	,213	m	82	righ	3,50	,707	2	
				Total	3,50	,707	2	
			Total	righ	3,50	,707	2	
				Total	3,50	,707	2	
		Total	82	righ	3,50	,707	2	
				Total	3,50	,707	2	
			Total	righ	3,50	,707	2	
				Total	3,50	,707	2	
	,214	m	85	righ	2,00	2,000	3	
				Total	2,00	2,000	3	
			Total	righ	2,00	2,000	3	
				Total	2,00	2,000	3	
		Total	85	righ	2,00	2,000	3	
				Total	2,00	2,000	3	
			Total	righ	2,00	2,000	3	
				Total	2,00	2,000	3	
	,219	f	71	righ	1,00	1,732	3	
				Total	1,00	1,732	3	
			Total	righ	1,00	1,732	3	
				Total	1,00	1,732	3	
		Total	71	righ	1,00	1,732	3	
				Total	1,00	1,732	3	
			Total	righ	1,00	1,732	3	
				Total	1,00	1,732	3	
	,221	f	74	righ	2,50	2,121	2	
				Total	2,50	2,121	2	
			Total	righ	2,50	2,121	2	
				Total	2,50	2,121	2	
		Total	74	righ	2,50	2,121	2	
				Total	2,50	2,121	2	
			Total	righ	2,50	2,121	2	
				Total	2,50	2,121	2	
	,224	m	85	righ	2,50	2,121	2	
				Total	2,50	2,121	2	
			Total	righ	2,50	2,121	2	
				Total	2,50	2,121	2	
		Total	85	righ	2,50	2,121	2	
				Total	2,50	2,121	2	
			Total	righ	2,50	2,121	2	
				Total	2,50	2,121	2	
	,224	f	97	righ	,67	,577	3	
				Total	,67	,577	3	
			Total	righ	,67	,577	3	
				Total	,67	,577	3	
		Total	97	righ	,67	,577	3	
				Total	,67	,577	3	
			Total	righ	,67	,577	3	
				Total	,67	,577	3	
	,226	f	82	left	3,00	,000	3	
				Total	3,00	,000	3	
			Total	left	3,00	,000	3	
				Total	3,00	,000	3	
		Total	82	left	3,00	,000	3	
				Total	3,00	,000	3	
			Total	left	3,00	,000	3	
				Total	3,00	,000	3	
	,226	f	83	righ	2,67	,577	3	
				Total	2,67	,577	3	
			Total	righ	2,67	,577	3	
				Total	2,67	,577	3	
		Total	83	righ	2,67	,577	3	
				Total	2,67	,577	3	
			Total	righ	2,67	,577	3	
				Total	2,67	,577	3	
	,228	m	87	righ	1,33	1,528	3	
				Total	1,33	1,528	3	
			Total	righ	1,33	1,528	3	
				Total	1,33	1,528	3	
		Total	87	righ	1,33	1,528	3	
				Total	1,33	1,528	3	
			Total	righ	1,33	1,528	3	
				Total	1,33	1,528	3	
	,254	m	85	left	1,67	1,155	3	
				Total	1,67	1,155	3	
			Total	left	1,67	1,155	3	
				Total	1,67	1,155	3	
		Total	85	left	1,67	1,155	3	
				Total	1,67	1,155	3	
			Total	left	1,67	1,155	3	
				Total	1,67	1,155	3	
	,265	m	85	righ	1,67	1,155	3	
				Total	1,67	1,155	3	
			Total	righ	1,67	1,155	3	
				Total	1,67	1,155	3	
		Total	85	righ	1,67	1,155	3	
				Total	1,67	1,155	3	
			Total	righ	1,67	1,155	3	
				Total	1,67	1,155	3	
	Total	f	60	left	2,33	1,155	3	
				Total	2,33	1,155	3	
			61	left	1,00	,000	3	
				Total	1,00	,000	3	
			63	righ	1,00	,000	3	
				Total	1,00	,000	3	
			64	righ	1,50	,707	2	
				Total	1,50	,707	2	
			71	left	2,00	1,732	3	
				righ	1,00	1,732	3	
				Total	1,50	1,643	6	
			73	righ	3,00	,000	2	
				Total	3,00	,000	2	
			74	righ	2,40	1,817	5	
				Total	2,40	1,817	5	
			78	left	2,20	1,095	5	
				Total	2,20	1,095	5	
			79	left	4,00	,000	3	
				righ	1,67	1,155	3	
				Total	2,83	1,472	6	
			80	left	,80	,447	5	
				righ	2,33	1,528	3	
				Total	1,38	1,188	8	
			81	righ	1,60	1,342	5	
				Total	1,60	1,342	5	
			82	left	3,00	,000	3	
				Total	3,00	,000	3	
			83	righ	2,67	1,033	6	
				Total	2,67	1,033	6	
			84	left	3,00	,000	3	
				righ	1,00	,000	8	
				Total	1,55	,934	11	
			85	righ	2,00	,000	3	
				Total	2,00	,000	3	
			97	righ	,67	,577	3	
				Total	,67	,577	3	
			Total	left	2,18	1,249	28	
				righ	1,72	1,186	46	
				Total	1,89	1,223	74	
		m	62	righ	1,50	,707	2	
				Total	1,50	,707	2	
			63	righ	1,50	2,121	2	
				Total	1,50	2,121	2	
			66	left	2,00	1,414	2	
				Total	2,00	1,414	2	
			69	righ	3,00	,000	3	
				Total	3,00	,000	3	
			70	righ	1,33	1,528	3	
				Total	1,33	1,528	3	
			71	righ	2,00	1,732	3	
				Total	2,00	1,732	3	
			72	righ	2,50	1,643	6	
				Total	2,50	1,643	6	
			73	left	1,33	1,323	9	
				Total	1,33	1,323	9	
			75	left	1,00	,000	3	
				righ	2,33	2,082	3	
				Total	1,67	1,506	6	
			76	left	1,80	1,643	5	
				righ	1,50	,707	2	
				Total	1,71	1,380	7	
			77	left	1,60	1,517	5	
				Total	1,60	1,517	5	
			78	left	1,67	1,155	3	
				righ	2,00	1,414	5	
				Total	1,88	1,246	8	
			80	left	1,33	1,528	3	
				righ	2,00	1,414	2	
				Total	1,60	1,342	5	
			81	righ	2,33	1,155	3	
				Total	2,33	1,155	3	
			82	left	1,50	1,225	6	
				righ	3,50	,707	2	
				Total	2,00	1,414	8	
			83	left	3,00	,000	3	
				Total	3,00	,000	3	
			84	righ	2,00	1,414	2	
				Total	2,00	1,414	2	
			85	left	2,00	1,173	17	
				righ	2,09	1,375	11	
				Total	2,04	1,232	28	
			87	righ	1,33	1,528	3	
				Total	1,33	1,528	3	
			88	righ	2,33	2,082	3	
				Total	2,33	2,082	3	
			90	left	1,50	2,121	2	
				Total	1,50	2,121	2	
			96	left	,33	,577	3	
				Total	,33	,577	3	
			Total	left	1,66	1,250	61	
				righ	2,11	1,356	55	
				Total	1,87	1,316	116	
		Total	60	left	2,33	1,155	3	
				Total	2,33	1,155	3	
			61	left	1,00	,000	3	
				Total	1,00	,000	3	
			62	righ	1,50	,707	2	
				Total	1,50	,707	2	
			63	righ	1,20	1,095	5	
				Total	1,20	1,095	5	
			64	righ	1,50	,707	2	
				Total	1,50	,707	2	
			66	left	2,00	1,414	2	
				Total	2,00	1,414	2	
			69	righ	3,00	,000	3	
				Total	3,00	,000	3	
			70	righ	1,33	1,528	3	
				Total	1,33	1,528	3	
			71	left	2,00	1,732	3	
				righ	1,50	1,643	6	
				Total	1,67	1,581	9	
			72	righ	2,50	1,643	6	
				Total	2,50	1,643	6	
			73	left	1,33	1,323	9	
				righ	3,00	,000	2	
				Total	1,64	1,362	11	
			74	righ	2,40	1,817	5	
				Total	2,40	1,817	5	
			75	left	1,00	,000	3	
				righ	2,33	2,082	3	
				Total	1,67	1,506	6	
			76	left	1,80	1,643	5	
				righ	1,50	,707	2	
				Total	1,71	1,380	7	
			77	left	1,60	1,517	5	
				Total	1,60	1,517	5	
			78	left	2,00	1,069	8	
				righ	2,00	1,414	5	
				Total	2,00	1,155	13	
			79	left	4,00	,000	3	
				righ	1,67	1,155	3	
				Total	2,83	1,472	6	
			80	left	1,00	,926	8	
				righ	2,20	1,304	5	
				Total	1,46	1,198	13	
			81	righ	1,88	1,246	8	
				Total	1,88	1,246	8	
			82	left	2,00	1,225	9	
				righ	3,50	,707	2	
				Total	2,27	1,272	11	
			83	left	3,00	,000	3	
				righ	2,67	1,033	6	
				Total	2,78	,833	9	
			84	left	3,00	,000	3	
				righ	1,20	,632	10	
				Total	1,62	,961	13	
			85	left	2,00	1,173	17	
				righ	2,07	1,207	14	
				Total	2,03	1,169	31	
			87	righ	1,33	1,528	3	
				Total	1,33	1,528	3	
			88	righ	2,33	2,082	3	
				Total	2,33	2,082	3	
			90	left	1,50	2,121	2	
				Total	1,50	2,121	2	
			96	left	,33	,577	3	
				Total	,33	,577	3	
			97	righ	,67	,577	3	
				Total	,67	,577	3	
			Total	left	1,82	1,266	89	
				righ	1,93	1,290	101	
				Total	1,88	1,277	190	
KPS PreOp	,100	m	63	righ	80,00	,000	2	
				Total	80,00	,000	2	
			Total	righ	80,00	,000	2	
				Total	80,00	,000	2	
		Total	63	righ	80,00	,000	2	
				Total	80,00	,000	2	
			Total	righ	80,00	,000	2	
				Total	80,00	,000	2	
	,111	m	70	righ	60,00	,000	3	
				Total	60,00	,000	3	
			Total	righ	60,00	,000	3	
				Total	60,00	,000	3	
		Total	70	righ	60,00	,000	3	
				Total	60,00	,000	3	
			Total	righ	60,00	,000	3	
				Total	60,00	,000	3	
	,120	m	72	righ	60,00	,000	3	
				Total	60,00	,000	3	
			Total	righ	60,00	,000	3	
				Total	60,00	,000	3	
		Total	72	righ	60,00	,000	3	
				Total	60,00	,000	3	
			Total	righ	60,00	,000	3	
				Total	60,00	,000	3	
	,125	m	73	left	60,00	,000	3	
				Total	60,00	,000	3	
			Total	left	60,00	,000	3	
				Total	60,00	,000	3	
		Total	73	left	60,00	,000	3	
				Total	60,00	,000	3	
			Total	left	60,00	,000	3	
				Total	60,00	,000	3	
	,127	f	74	righ	90,00	,000	3	
				Total	90,00	,000	3	
			Total	righ	90,00	,000	3	
				Total	90,00	,000	3	
		Total	74	righ	90,00	,000	3	
				Total	90,00	,000	3	
			Total	righ	90,00	,000	3	
				Total	90,00	,000	3	
	,131	m	66	left	40,00	,000	2	
				Total	40,00	,000	2	
			Total	left	40,00	,000	2	
				Total	40,00	,000	2	
		Total	66	left	40,00	,000	2	
				Total	40,00	,000	2	
			Total	left	40,00	,000	2	
				Total	40,00	,000	2	
	,131	f	84	righ	60,00	,000	3	
				Total	60,00	,000	3	
			Total	righ	60,00	,000	3	
				Total	60,00	,000	3	
		Total	84	righ	60,00	,000	3	
				Total	60,00	,000	3	
			Total	righ	60,00	,000	3	
				Total	60,00	,000	3	
	,131	m	75	righ	40,00	,000	3	
				Total	40,00	,000	3	
			Total	righ	40,00	,000	3	
				Total	40,00	,000	3	
		Total	75	righ	40,00	,000	3	
				Total	40,00	,000	3	
			Total	righ	40,00	,000	3	
				Total	40,00	,000	3	
	,134	m	76	left	60,00	,000	3	
				Total	60,00	,000	3	
			Total	left	60,00	,000	3	
				Total	60,00	,000	3	
		Total	76	left	60,00	,000	3	
				Total	60,00	,000	3	
			Total	left	60,00	,000	3	
				Total	60,00	,000	3	
	,134	m	69	righ	60,00	,000	3	
				Total	60,00	,000	3	
			Total	righ	60,00	,000	3	
				Total	60,00	,000	3	
		Total	69	righ	60,00	,000	3	
				Total	60,00	,000	3	
			Total	righ	60,00	,000	3	
				Total	60,00	,000	3	
	,141	m	80	righ	60,00	,000	2	
				Total	60,00	,000	2	
			Total	righ	60,00	,000	2	
				Total	60,00	,000	2	
		Total	80	righ	60,00	,000	2	
				Total	60,00	,000	2	
			Total	righ	60,00	,000	2	
				Total	60,00	,000	2	
	,143	f	63	righ	60,00	,000	3	
				Total	60,00	,000	3	
			Total	righ	60,00	,000	3	
				Total	60,00	,000	3	
		Total	63	righ	60,00	,000	3	
				Total	60,00	,000	3	
			Total	righ	60,00	,000	3	
				Total	60,00	,000	3	
	,144	f	73	righ	60,00	,000	2	
				Total	60,00	,000	2	
			Total	righ	60,00	,000	2	
				Total	60,00	,000	2	
		Total	73	righ	60,00	,000	2	
				Total	60,00	,000	2	
			Total	righ	60,00	,000	2	
				Total	60,00	,000	2	
	,146	m	78	righ	60,00	,000	2	
				Total	60,00	,000	2	
			Total	righ	60,00	,000	2	
				Total	60,00	,000	2	
		Total	78	righ	60,00	,000	2	
				Total	60,00	,000	2	
			Total	righ	60,00	,000	2	
				Total	60,00	,000	2	
	,146	f	61	left	60,00	,000	3	
				Total	60,00	,000	3	
			Total	left	60,00	,000	3	
				Total	60,00	,000	3	
		Total	61	left	60,00	,000	3	
				Total	60,00	,000	3	
			Total	left	60,00	,000	3	
				Total	60,00	,000	3	
	,148	f	81	righ	80,00	,000	3	
				Total	80,00	,000	3	
			Total	righ	80,00	,000	3	
				Total	80,00	,000	3	
		Total	81	righ	80,00	,000	3	
				Total	80,00	,000	3	
			Total	righ	80,00	,000	3	
				Total	80,00	,000	3	
	,148	f	84	left	60,00	,000	3	
				Total	60,00	,000	3	
			Total	left	60,00	,000	3	
				Total	60,00	,000	3	
		Total	84	left	60,00	,000	3	
				Total	60,00	,000	3	
			Total	left	60,00	,000	3	
				Total	60,00	,000	3	
	,150	f	80	left	60,00	,000	3	
				Total	60,00	,000	3	
			Total	left	60,00	,000	3	
				Total	60,00	,000	3	
		Total	80	left	60,00	,000	3	
				Total	60,00	,000	3	
			Total	left	60,00	,000	3	
				Total	60,00	,000	3	
	,152	m	73	left	60,00	,000	3	
				Total	60,00	,000	3	
			Total	left	60,00	,000	3	
				Total	60,00	,000	3	
		Total	73	left	60,00	,000	3	
				Total	60,00	,000	3	
			Total	left	60,00	,000	3	
				Total	60,00	,000	3	
	,152	m	72	righ	60,00	,000	3	
				Total	60,00	,000	3	
			Total	righ	60,00	,000	3	
				Total	60,00	,000	3	
		Total	72	righ	60,00	,000	3	
				Total	60,00	,000	3	
			Total	righ	60,00	,000	3	
				Total	60,00	,000	3	
	,156	m	85	left	40,00	,000	3	
				Total	40,00	,000	3	
			Total	left	40,00	,000	3	
				Total	40,00	,000	3	
		Total	85	left	40,00	,000	3	
				Total	40,00	,000	3	
			Total	left	40,00	,000	3	
				Total	40,00	,000	3	
	,159	m	88	righ	80,00	,000	3	
				Total	80,00	,000	3	
			Total	righ	80,00	,000	3	
				Total	80,00	,000	3	
		Total	88	righ	80,00	,000	3	
				Total	80,00	,000	3	
			Total	righ	80,00	,000	3	
				Total	80,00	,000	3	
	,159	m	77	left	60,00	,000	3	
				Total	60,00	,000	3	
			Total	left	60,00	,000	3	
				Total	60,00	,000	3	
		Total	77	left	60,00	,000	3	
				Total	60,00	,000	3	
			Total	left	60,00	,000	3	
				Total	60,00	,000	3	
	,159	f	79	righ	70,00	,000	3	
				Total	70,00	,000	3	
			Total	righ	70,00	,000	3	
				Total	70,00	,000	3	
		Total	79	righ	70,00	,000	3	
				Total	70,00	,000	3	
			Total	righ	70,00	,000	3	
				Total	70,00	,000	3	
	,160	f	80	righ	60,00	,000	3	
				Total	60,00	,000	3	
			Total	righ	60,00	,000	3	
				Total	60,00	,000	3	
		Total	80	righ	60,00	,000	3	
				Total	60,00	,000	3	
			Total	righ	60,00	,000	3	
				Total	60,00	,000	3	
	,163	m	75	left	60,00	,000	3	
				Total	60,00	,000	3	
			Total	left	60,00	,000	3	
				Total	60,00	,000	3	
		Total	75	left	60,00	,000	3	
				Total	60,00	,000	3	
			Total	left	60,00	,000	3	
				Total	60,00	,000	3	
	,167	m	73	left	60,00	,000	3	
				Total	60,00	,000	3	
			Total	left	60,00	,000	3	
				Total	60,00	,000	3	
		Total	73	left	60,00	,000	3	
				Total	60,00	,000	3	
			Total	left	60,00	,000	3	
				Total	60,00	,000	3	
	,168	f	60	left	40,00	,000	3	
				Total	40,00	,000	3	
			Total	left	40,00	,000	3	
				Total	40,00	,000	3	
		Total	60	left	40,00	,000	3	
				Total	40,00	,000	3	
			Total	left	40,00	,000	3	
				Total	40,00	,000	3	
	,170	m	62	righ	80,00	,000	2	
				Total	80,00	,000	2	
			Total	righ	80,00	,000	2	
				Total	80,00	,000	2	
		Total	62	righ	80,00	,000	2	
				Total	80,00	,000	2	
			Total	righ	80,00	,000	2	
				Total	80,00	,000	2	
	,170	f	84	righ	60,00	,000	2	
				Total	60,00	,000	2	
			Total	righ	60,00	,000	2	
				Total	60,00	,000	2	
		Total	84	righ	60,00	,000	2	
				Total	60,00	,000	2	
			Total	righ	60,00	,000	2	
				Total	60,00	,000	2	
	,171	f	78	left	60,00	,000	3	
				Total	60,00	,000	3	
			Total	left	60,00	,000	3	
				Total	60,00	,000	3	
		Total	78	left	60,00	,000	3	
				Total	60,00	,000	3	
			Total	left	60,00	,000	3	
				Total	60,00	,000	3	
	,172	f	84	righ	60,00	,000	3	
				Total	60,00	,000	3	
			Total	righ	60,00	,000	3	
				Total	60,00	,000	3	
		Total	84	righ	60,00	,000	3	
				Total	60,00	,000	3	
			Total	righ	60,00	,000	3	
				Total	60,00	,000	3	
	,173	m	76	righ	40,00	,000	2	
				Total	40,00	,000	2	
			Total	righ	40,00	,000	2	
				Total	40,00	,000	2	
		Total	76	righ	40,00	,000	2	
				Total	40,00	,000	2	
			Total	righ	40,00	,000	2	
				Total	40,00	,000	2	
	,174	m	77	left	60,00	,000	2	
				Total	60,00	,000	2	
			Total	left	60,00	,000	2	
				Total	60,00	,000	2	
		Total	77	left	60,00	,000	2	
				Total	60,00	,000	2	
			Total	left	60,00	,000	2	
				Total	60,00	,000	2	
	,175	m	76	left	80,00	,000	2	
				Total	80,00	,000	2	
			Total	left	80,00	,000	2	
				Total	80,00	,000	2	
		Total	76	left	80,00	,000	2	
				Total	80,00	,000	2	
			Total	left	80,00	,000	2	
				Total	80,00	,000	2	
	,176	m	85	left	60,00	,000	3	
				Total	60,00	,000	3	
			Total	left	60,00	,000	3	
				Total	60,00	,000	3	
		Total	85	left	60,00	,000	3	
				Total	60,00	,000	3	
			Total	left	60,00	,000	3	
				Total	60,00	,000	3	
	,177	m	85	left	80,00	,000	3	
				Total	80,00	,000	3	
			Total	left	80,00	,000	3	
				Total	80,00	,000	3	
		Total	85	left	80,00	,000	3	
				Total	80,00	,000	3	
			Total	left	80,00	,000	3	
				Total	80,00	,000	3	
	,180	f	64	righ	40,00	,000	2	
				Total	40,00	,000	2	
			Total	righ	40,00	,000	2	
				Total	40,00	,000	2	
		Total	64	righ	40,00	,000	2	
				Total	40,00	,000	2	
			Total	righ	40,00	,000	2	
				Total	40,00	,000	2	
	,181	f	80	left	60,00	,000	2	
				Total	60,00	,000	2	
			Total	left	60,00	,000	2	
				Total	60,00	,000	2	
		Total	80	left	60,00	,000	2	
				Total	60,00	,000	2	
			Total	left	60,00	,000	2	
				Total	60,00	,000	2	
	,183	f	85	righ	70,00	,000	3	
				Total	70,00	,000	3	
			Total	righ	70,00	,000	3	
				Total	70,00	,000	3	
		Total	85	righ	70,00	,000	3	
				Total	70,00	,000	3	
			Total	righ	70,00	,000	3	
				Total	70,00	,000	3	
	,185	m	71	righ	60,00	,000	3	
				Total	60,00	,000	3	
			Total	righ	60,00	,000	3	
				Total	60,00	,000	3	
		Total	71	righ	60,00	,000	3	
				Total	60,00	,000	3	
			Total	righ	60,00	,000	3	
				Total	60,00	,000	3	
	,188	m	78	righ	60,00	,000	3	
				Total	60,00	,000	3	
			Total	righ	60,00	,000	3	
				Total	60,00	,000	3	
		Total	78	righ	60,00	,000	3	
				Total	60,00	,000	3	
			Total	righ	60,00	,000	3	
				Total	60,00	,000	3	
	,188	m	81	righ	60,00	,000	3	
				Total	60,00	,000	3	
			Total	righ	60,00	,000	3	
				Total	60,00	,000	3	
		Total	81	righ	60,00	,000	3	
				Total	60,00	,000	3	
			Total	righ	60,00	,000	3	
				Total	60,00	,000	3	
	,189	m	85	righ	60,00	,000	3	
				Total	60,00	,000	3	
			Total	righ	60,00	,000	3	
				Total	60,00	,000	3	
		Total	85	righ	60,00	,000	3	
				Total	60,00	,000	3	
			Total	righ	60,00	,000	3	
				Total	60,00	,000	3	
	,190	f	81	righ	10,00	,000	2	
				Total	10,00	,000	2	
			Total	righ	10,00	,000	2	
				Total	10,00	,000	2	
		Total	81	righ	10,00	,000	2	
				Total	10,00	,000	2	
			Total	righ	10,00	,000	2	
				Total	10,00	,000	2	
	,191	f	79	left	60,00	,000	3	
				Total	60,00	,000	3	
			Total	left	60,00	,000	3	
				Total	60,00	,000	3	
		Total	79	left	60,00	,000	3	
				Total	60,00	,000	3	
			Total	left	60,00	,000	3	
				Total	60,00	,000	3	
	,192	f	78	left	80,00	,000	2	
				Total	80,00	,000	2	
			Total	left	80,00	,000	2	
				Total	80,00	,000	2	
		Total	78	left	80,00	,000	2	
				Total	80,00	,000	2	
			Total	left	80,00	,000	2	
				Total	80,00	,000	2	
	,192	f	71	left	40,00	,000	3	
				Total	40,00	,000	3	
			Total	left	40,00	,000	3	
				Total	40,00	,000	3	
		Total	71	left	40,00	,000	3	
				Total	40,00	,000	3	
			Total	left	40,00	,000	3	
				Total	40,00	,000	3	
	,194	m	85	left	60,00	,000	2	
				Total	60,00	,000	2	
			Total	left	60,00	,000	2	
				Total	60,00	,000	2	
		Total	85	left	60,00	,000	2	
				Total	60,00	,000	2	
			Total	left	60,00	,000	2	
				Total	60,00	,000	2	
	,198	m	84	righ	40,00	,000	2	
				Total	40,00	,000	2	
			Total	righ	40,00	,000	2	
				Total	40,00	,000	2	
		Total	84	righ	40,00	,000	2	
				Total	40,00	,000	2	
			Total	righ	40,00	,000	2	
				Total	40,00	,000	2	
	,198	m	90	left	80,00	,000	2	
				Total	80,00	,000	2	
			Total	left	80,00	,000	2	
				Total	80,00	,000	2	
		Total	90	left	80,00	,000	2	
				Total	80,00	,000	2	
			Total	left	80,00	,000	2	
				Total	80,00	,000	2	
	,200	m	80	left	60,00	,000	3	
				Total	60,00	,000	3	
			Total	left	60,00	,000	3	
				Total	60,00	,000	3	
		Total	80	left	60,00	,000	3	
				Total	60,00	,000	3	
			Total	left	60,00	,000	3	
				Total	60,00	,000	3	
	,200	m	78	left	70,00	,000	3	
				Total	70,00	,000	3	
			Total	left	70,00	,000	3	
				Total	70,00	,000	3	
		Total	78	left	70,00	,000	3	
				Total	70,00	,000	3	
			Total	left	70,00	,000	3	
				Total	70,00	,000	3	
	,201	m	96	left	40,00	,000	3	
				Total	40,00	,000	3	
			Total	left	40,00	,000	3	
				Total	40,00	,000	3	
		Total	96	left	40,00	,000	3	
				Total	40,00	,000	3	
			Total	left	40,00	,000	3	
				Total	40,00	,000	3	
	,207	m	83	left	50,00	,000	3	
				Total	50,00	,000	3	
			Total	left	50,00	,000	3	
				Total	50,00	,000	3	
		Total	83	left	50,00	,000	3	
				Total	50,00	,000	3	
			Total	left	50,00	,000	3	
				Total	50,00	,000	3	
	,208	f	83	righ	70,00	,000	3	
				Total	70,00	,000	3	
			Total	righ	70,00	,000	3	
				Total	70,00	,000	3	
		Total	83	righ	70,00	,000	3	
				Total	70,00	,000	3	
			Total	righ	70,00	,000	3	
				Total	70,00	,000	3	
	,210	m	82	left	70,00	,000	3	
				Total	70,00	,000	3	
			Total	left	70,00	,000	3	
				Total	70,00	,000	3	
		Total	82	left	70,00	,000	3	
				Total	70,00	,000	3	
			Total	left	70,00	,000	3	
				Total	70,00	,000	3	
	,211	m	85	left	50,00	,000	3	
				Total	50,00	,000	3	
			Total	left	50,00	,000	3	
				Total	50,00	,000	3	
		Total	85	left	50,00	,000	3	
				Total	50,00	,000	3	
			Total	left	50,00	,000	3	
				Total	50,00	,000	3	
	,213	m	82	left	60,00	,000	3	
				Total	60,00	,000	3	
			Total	left	60,00	,000	3	
				Total	60,00	,000	3	
		Total	82	left	60,00	,000	3	
				Total	60,00	,000	3	
			Total	left	60,00	,000	3	
				Total	60,00	,000	3	
	,213	m	82	righ	60,00	,000	2	
				Total	60,00	,000	2	
			Total	righ	60,00	,000	2	
				Total	60,00	,000	2	
		Total	82	righ	60,00	,000	2	
				Total	60,00	,000	2	
			Total	righ	60,00	,000	2	
				Total	60,00	,000	2	
	,214	m	85	righ	60,00	,000	3	
				Total	60,00	,000	3	
			Total	righ	60,00	,000	3	
				Total	60,00	,000	3	
		Total	85	righ	60,00	,000	3	
				Total	60,00	,000	3	
			Total	righ	60,00	,000	3	
				Total	60,00	,000	3	
	,219	f	71	righ	40,00	,000	3	
				Total	40,00	,000	3	
			Total	righ	40,00	,000	3	
				Total	40,00	,000	3	
		Total	71	righ	40,00	,000	3	
				Total	40,00	,000	3	
			Total	righ	40,00	,000	3	
				Total	40,00	,000	3	
	,221	f	74	righ	40,00	,000	2	
				Total	40,00	,000	2	
			Total	righ	40,00	,000	2	
				Total	40,00	,000	2	
		Total	74	righ	40,00	,000	2	
				Total	40,00	,000	2	
			Total	righ	40,00	,000	2	
				Total	40,00	,000	2	
	,224	m	85	righ	40,00	,000	2	
				Total	40,00	,000	2	
			Total	righ	40,00	,000	2	
				Total	40,00	,000	2	
		Total	85	righ	40,00	,000	2	
				Total	40,00	,000	2	
			Total	righ	40,00	,000	2	
				Total	40,00	,000	2	
	,224	f	97	righ	70,00	,000	3	
				Total	70,00	,000	3	
			Total	righ	70,00	,000	3	
				Total	70,00	,000	3	
		Total	97	righ	70,00	,000	3	
				Total	70,00	,000	3	
			Total	righ	70,00	,000	3	
				Total	70,00	,000	3	
	,226	f	82	left	40,00	,000	3	
				Total	40,00	,000	3	
			Total	left	40,00	,000	3	
				Total	40,00	,000	3	
		Total	82	left	40,00	,000	3	
				Total	40,00	,000	3	
			Total	left	40,00	,000	3	
				Total	40,00	,000	3	
	,226	f	83	righ	10,00	,000	3	
				Total	10,00	,000	3	
			Total	righ	10,00	,000	3	
				Total	10,00	,000	3	
		Total	83	righ	10,00	,000	3	
				Total	10,00	,000	3	
			Total	righ	10,00	,000	3	
				Total	10,00	,000	3	
	,228	m	87	righ	60,00	,000	3	
				Total	60,00	,000	3	
			Total	righ	60,00	,000	3	
				Total	60,00	,000	3	
		Total	87	righ	60,00	,000	3	
				Total	60,00	,000	3	
			Total	righ	60,00	,000	3	
				Total	60,00	,000	3	
	,254	m	85	left	40,00	,000	3	
				Total	40,00	,000	3	
			Total	left	40,00	,000	3	
				Total	40,00	,000	3	
		Total	85	left	40,00	,000	3	
				Total	40,00	,000	3	
			Total	left	40,00	,000	3	
				Total	40,00	,000	3	
	,265	m	85	righ	70,00	,000	3	
				Total	70,00	,000	3	
			Total	righ	70,00	,000	3	
				Total	70,00	,000	3	
		Total	85	righ	70,00	,000	3	
				Total	70,00	,000	3	
			Total	righ	70,00	,000	3	
				Total	70,00	,000	3	
	Total	f	60	left	40,00	,000	3	
				Total	40,00	,000	3	
			61	left	60,00	,000	3	
				Total	60,00	,000	3	
			63	righ	60,00	,000	3	
				Total	60,00	,000	3	
			64	righ	40,00	,000	2	
				Total	40,00	,000	2	
			71	left	40,00	,000	3	
				righ	40,00	,000	3	
				Total	40,00	,000	6	
			73	righ	60,00	,000	2	
				Total	60,00	,000	2	
			74	righ	70,00	27,386	5	
				Total	70,00	27,386	5	
			78	left	68,00	10,954	5	
				Total	68,00	10,954	5	
			79	left	60,00	,000	3	
				righ	70,00	,000	3	
				Total	65,00	5,477	6	
			80	left	60,00	,000	5	
				righ	60,00	,000	3	
				Total	60,00	,000	8	
			81	righ	52,00	38,341	5	
				Total	52,00	38,341	5	
			82	left	40,00	,000	3	
				Total	40,00	,000	3	
			83	righ	40,00	32,863	6	
				Total	40,00	32,863	6	
			84	left	60,00	,000	3	
				righ	60,00	,000	8	
				Total	60,00	,000	11	
			85	righ	70,00	,000	3	
				Total	70,00	,000	3	
			97	righ	70,00	,000	3	
				Total	70,00	,000	3	
			Total	left	55,00	11,706	28	
				righ	57,39	21,128	46	
				Total	56,49	18,089	74	
		m	62	righ	80,00	,000	2	
				Total	80,00	,000	2	
			63	righ	80,00	,000	2	
				Total	80,00	,000	2	
			66	left	40,00	,000	2	
				Total	40,00	,000	2	
			69	righ	60,00	,000	3	
				Total	60,00	,000	3	
			70	righ	60,00	,000	3	
				Total	60,00	,000	3	
			71	righ	60,00	,000	3	
				Total	60,00	,000	3	
			72	righ	60,00	,000	6	
				Total	60,00	,000	6	
			73	left	60,00	,000	9	
				Total	60,00	,000	9	
			75	left	60,00	,000	3	
				righ	40,00	,000	3	
				Total	50,00	10,954	6	
			76	left	68,00	10,954	5	
				righ	40,00	,000	2	
				Total	60,00	16,330	7	
			77	left	60,00	,000	5	
				Total	60,00	,000	5	
			78	left	70,00	,000	3	
				righ	60,00	,000	5	
				Total	63,75	5,175	8	
			80	left	60,00	,000	3	
				righ	60,00	,000	2	
				Total	60,00	,000	5	
			81	righ	60,00	,000	3	
				Total	60,00	,000	3	
			82	left	65,00	5,477	6	
				righ	60,00	,000	2	
				Total	63,75	5,175	8	
			83	left	50,00	,000	3	
				Total	50,00	,000	3	
			84	righ	40,00	,000	2	
				Total	40,00	,000	2	
			85	left	54,71	14,628	17	
				righ	59,09	10,445	11	
				Total	56,43	13,113	28	
			87	righ	60,00	,000	3	
				Total	60,00	,000	3	
			88	righ	80,00	,000	3	
				Total	80,00	,000	3	
			90	left	80,00	,000	2	
				Total	80,00	,000	2	
			96	left	40,00	,000	3	
				Total	40,00	,000	3	
			Total	left	58,69	11,758	61	
				righ	59,82	11,137	55	
				Total	59,22	11,432	116	
		Total	60	left	40,00	,000	3	
				Total	40,00	,000	3	
			61	left	60,00	,000	3	
				Total	60,00	,000	3	
			62	righ	80,00	,000	2	
				Total	80,00	,000	2	
			63	righ	68,00	10,954	5	
				Total	68,00	10,954	5	
			64	righ	40,00	,000	2	
				Total	40,00	,000	2	
			66	left	40,00	,000	2	
				Total	40,00	,000	2	
			69	righ	60,00	,000	3	
				Total	60,00	,000	3	
			70	righ	60,00	,000	3	
				Total	60,00	,000	3	
			71	left	40,00	,000	3	
				righ	50,00	10,954	6	
				Total	46,67	10,000	9	
			72	righ	60,00	,000	6	
				Total	60,00	,000	6	
			73	left	60,00	,000	9	
				righ	60,00	,000	2	
				Total	60,00	,000	11	
			74	righ	70,00	27,386	5	
				Total	70,00	27,386	5	
			75	left	60,00	,000	3	
				righ	40,00	,000	3	
				Total	50,00	10,954	6	
			76	left	68,00	10,954	5	
				righ	40,00	,000	2	
				Total	60,00	16,330	7	
			77	left	60,00	,000	5	
				Total	60,00	,000	5	
			78	left	68,75	8,345	8	
				righ	60,00	,000	5	
				Total	65,38	7,763	13	
			79	left	60,00	,000	3	
				righ	70,00	,000	3	
				Total	65,00	5,477	6	
			80	left	60,00	,000	8	
				righ	60,00	,000	5	
				Total	60,00	,000	13	
			81	righ	55,00	29,277	8	
				Total	55,00	29,277	8	
			82	left	56,67	13,229	9	
				righ	60,00	,000	2	
				Total	57,27	11,909	11	
			83	left	50,00	,000	3	
				righ	40,00	32,863	6	
				Total	43,33	26,458	9	
			84	left	60,00	,000	3	
				righ	56,00	8,433	10	
				Total	56,92	7,511	13	
			85	left	54,71	14,628	17	
				righ	61,43	10,271	14	
				Total	57,74	13,092	31	
			87	righ	60,00	,000	3	
				Total	60,00	,000	3	
			88	righ	80,00	,000	3	
				Total	80,00	,000	3	
			90	left	80,00	,000	2	
				Total	80,00	,000	2	
			96	left	40,00	,000	3	
				Total	40,00	,000	3	
			97	righ	70,00	,000	3	
				Total	70,00	,000	3	
			Total	left	57,53	11,801	89	
				righ	58,71	16,411	101	
				Total	58,16	14,412	190	
KPS PostOp	,100	m	63	righ	100,00	,000	2	
				Total	100,00	,000	2	
			Total	righ	100,00	,000	2	
				Total	100,00	,000	2	
		Total	63	righ	100,00	,000	2	
				Total	100,00	,000	2	
			Total	righ	100,00	,000	2	
				Total	100,00	,000	2	
	,111	m	70	righ	90,00	,000	3	
				Total	90,00	,000	3	
			Total	righ	90,00	,000	3	
				Total	90,00	,000	3	
		Total	70	righ	90,00	,000	3	
				Total	90,00	,000	3	
			Total	righ	90,00	,000	3	
				Total	90,00	,000	3	
	,120	m	72	righ	100,00	,000	3	
				Total	100,00	,000	3	
			Total	righ	100,00	,000	3	
				Total	100,00	,000	3	
		Total	72	righ	100,00	,000	3	
				Total	100,00	,000	3	
			Total	righ	100,00	,000	3	
				Total	100,00	,000	3	
	,125	m	73	left	90,00	,000	3	
				Total	90,00	,000	3	
			Total	left	90,00	,000	3	
				Total	90,00	,000	3	
		Total	73	left	90,00	,000	3	
				Total	90,00	,000	3	
			Total	left	90,00	,000	3	
				Total	90,00	,000	3	
	,127	f	74	righ	100,00	,000	3	
				Total	100,00	,000	3	
			Total	righ	100,00	,000	3	
				Total	100,00	,000	3	
		Total	74	righ	100,00	,000	3	
				Total	100,00	,000	3	
			Total	righ	100,00	,000	3	
				Total	100,00	,000	3	
	,131	m	66	left	90,00	,000	2	
				Total	90,00	,000	2	
			Total	left	90,00	,000	2	
				Total	90,00	,000	2	
		Total	66	left	90,00	,000	2	
				Total	90,00	,000	2	
			Total	left	90,00	,000	2	
				Total	90,00	,000	2	
	,131	f	84	righ	80,00	,000	3	
				Total	80,00	,000	3	
			Total	righ	80,00	,000	3	
				Total	80,00	,000	3	
		Total	84	righ	80,00	,000	3	
				Total	80,00	,000	3	
			Total	righ	80,00	,000	3	
				Total	80,00	,000	3	
	,131	m	75	righ	80,00	,000	3	
				Total	80,00	,000	3	
			Total	righ	80,00	,000	3	
				Total	80,00	,000	3	
		Total	75	righ	80,00	,000	3	
				Total	80,00	,000	3	
			Total	righ	80,00	,000	3	
				Total	80,00	,000	3	
	,134	m	76	left	90,00	,000	3	
				Total	90,00	,000	3	
			Total	left	90,00	,000	3	
				Total	90,00	,000	3	
		Total	76	left	90,00	,000	3	
				Total	90,00	,000	3	
			Total	left	90,00	,000	3	
				Total	90,00	,000	3	
	,134	m	69	righ	90,00	,000	3	
				Total	90,00	,000	3	
			Total	righ	90,00	,000	3	
				Total	90,00	,000	3	
		Total	69	righ	90,00	,000	3	
				Total	90,00	,000	3	
			Total	righ	90,00	,000	3	
				Total	90,00	,000	3	
	,141	m	80	righ	90,00	,000	2	
				Total	90,00	,000	2	
			Total	righ	90,00	,000	2	
				Total	90,00	,000	2	
		Total	80	righ	90,00	,000	2	
				Total	90,00	,000	2	
			Total	righ	90,00	,000	2	
				Total	90,00	,000	2	
	,143	f	63	righ	90,00	,000	3	
				Total	90,00	,000	3	
			Total	righ	90,00	,000	3	
				Total	90,00	,000	3	
		Total	63	righ	90,00	,000	3	
				Total	90,00	,000	3	
			Total	righ	90,00	,000	3	
				Total	90,00	,000	3	
	,144	f	73	righ	90,00	,000	2	
				Total	90,00	,000	2	
			Total	righ	90,00	,000	2	
				Total	90,00	,000	2	
		Total	73	righ	90,00	,000	2	
				Total	90,00	,000	2	
			Total	righ	90,00	,000	2	
				Total	90,00	,000	2	
	,146	m	78	righ	90,00	,000	2	
				Total	90,00	,000	2	
			Total	righ	90,00	,000	2	
				Total	90,00	,000	2	
		Total	78	righ	90,00	,000	2	
				Total	90,00	,000	2	
			Total	righ	90,00	,000	2	
				Total	90,00	,000	2	
	,146	f	61	left	90,00	,000	3	
				Total	90,00	,000	3	
			Total	left	90,00	,000	3	
				Total	90,00	,000	3	
		Total	61	left	90,00	,000	3	
				Total	90,00	,000	3	
			Total	left	90,00	,000	3	
				Total	90,00	,000	3	
	,148	f	81	righ	90,00	,000	3	
				Total	90,00	,000	3	
			Total	righ	90,00	,000	3	
				Total	90,00	,000	3	
		Total	81	righ	90,00	,000	3	
				Total	90,00	,000	3	
			Total	righ	90,00	,000	3	
				Total	90,00	,000	3	
	,148	f	84	left	80,00	,000	3	
				Total	80,00	,000	3	
			Total	left	80,00	,000	3	
				Total	80,00	,000	3	
		Total	84	left	80,00	,000	3	
				Total	80,00	,000	3	
			Total	left	80,00	,000	3	
				Total	80,00	,000	3	
	,150	f	80	left	90,00	,000	3	
				Total	90,00	,000	3	
			Total	left	90,00	,000	3	
				Total	90,00	,000	3	
		Total	80	left	90,00	,000	3	
				Total	90,00	,000	3	
			Total	left	90,00	,000	3	
				Total	90,00	,000	3	
	,152	m	73	left	80,00	,000	3	
				Total	80,00	,000	3	
			Total	left	80,00	,000	3	
				Total	80,00	,000	3	
		Total	73	left	80,00	,000	3	
				Total	80,00	,000	3	
			Total	left	80,00	,000	3	
				Total	80,00	,000	3	
	,152	m	72	righ	90,00	,000	3	
				Total	90,00	,000	3	
			Total	righ	90,00	,000	3	
				Total	90,00	,000	3	
		Total	72	righ	90,00	,000	3	
				Total	90,00	,000	3	
			Total	righ	90,00	,000	3	
				Total	90,00	,000	3	
	,156	m	85	left	80,00	,000	3	
				Total	80,00	,000	3	
			Total	left	80,00	,000	3	
				Total	80,00	,000	3	
		Total	85	left	80,00	,000	3	
				Total	80,00	,000	3	
			Total	left	80,00	,000	3	
				Total	80,00	,000	3	
	,159	m	88	righ	90,00	,000	3	
				Total	90,00	,000	3	
			Total	righ	90,00	,000	3	
				Total	90,00	,000	3	
		Total	88	righ	90,00	,000	3	
				Total	90,00	,000	3	
			Total	righ	90,00	,000	3	
				Total	90,00	,000	3	
	,159	m	77	left	90,00	,000	3	
				Total	90,00	,000	3	
			Total	left	90,00	,000	3	
				Total	90,00	,000	3	
		Total	77	left	90,00	,000	3	
				Total	90,00	,000	3	
			Total	left	90,00	,000	3	
				Total	90,00	,000	3	
	,159	f	79	righ	90,00	,000	3	
				Total	90,00	,000	3	
			Total	righ	90,00	,000	3	
				Total	90,00	,000	3	
		Total	79	righ	90,00	,000	3	
				Total	90,00	,000	3	
			Total	righ	90,00	,000	3	
				Total	90,00	,000	3	
	,160	f	80	righ	80,00	,000	3	
				Total	80,00	,000	3	
			Total	righ	80,00	,000	3	
				Total	80,00	,000	3	
		Total	80	righ	80,00	,000	3	
				Total	80,00	,000	3	
			Total	righ	80,00	,000	3	
				Total	80,00	,000	3	
	,163	m	75	left	90,00	,000	3	
				Total	90,00	,000	3	
			Total	left	90,00	,000	3	
				Total	90,00	,000	3	
		Total	75	left	90,00	,000	3	
				Total	90,00	,000	3	
			Total	left	90,00	,000	3	
				Total	90,00	,000	3	
	,167	m	73	left	90,00	,000	3	
				Total	90,00	,000	3	
			Total	left	90,00	,000	3	
				Total	90,00	,000	3	
		Total	73	left	90,00	,000	3	
				Total	90,00	,000	3	
			Total	left	90,00	,000	3	
				Total	90,00	,000	3	
	,168	f	60	left	90,00	,000	3	
				Total	90,00	,000	3	
			Total	left	90,00	,000	3	
				Total	90,00	,000	3	
		Total	60	left	90,00	,000	3	
				Total	90,00	,000	3	
			Total	left	90,00	,000	3	
				Total	90,00	,000	3	
	,170	m	62	righ	100,00	,000	2	
				Total	100,00	,000	2	
			Total	righ	100,00	,000	2	
				Total	100,00	,000	2	
		Total	62	righ	100,00	,000	2	
				Total	100,00	,000	2	
			Total	righ	100,00	,000	2	
				Total	100,00	,000	2	
	,170	f	84	righ	70,00	,000	2	
				Total	70,00	,000	2	
			Total	righ	70,00	,000	2	
				Total	70,00	,000	2	
		Total	84	righ	70,00	,000	2	
				Total	70,00	,000	2	
			Total	righ	70,00	,000	2	
				Total	70,00	,000	2	
	,171	f	78	left	90,00	,000	3	
				Total	90,00	,000	3	
			Total	left	90,00	,000	3	
				Total	90,00	,000	3	
		Total	78	left	90,00	,000	3	
				Total	90,00	,000	3	
			Total	left	90,00	,000	3	
				Total	90,00	,000	3	
	,172	f	84	righ	90,00	,000	3	
				Total	90,00	,000	3	
			Total	righ	90,00	,000	3	
				Total	90,00	,000	3	
		Total	84	righ	90,00	,000	3	
				Total	90,00	,000	3	
			Total	righ	90,00	,000	3	
				Total	90,00	,000	3	
	,173	m	76	righ	90,00	,000	2	
				Total	90,00	,000	2	
			Total	righ	90,00	,000	2	
				Total	90,00	,000	2	
		Total	76	righ	90,00	,000	2	
				Total	90,00	,000	2	
			Total	righ	90,00	,000	2	
				Total	90,00	,000	2	
	,174	m	77	left	90,00	,000	2	
				Total	90,00	,000	2	
			Total	left	90,00	,000	2	
				Total	90,00	,000	2	
		Total	77	left	90,00	,000	2	
				Total	90,00	,000	2	
			Total	left	90,00	,000	2	
				Total	90,00	,000	2	
	,175	m	76	left	100,00	,000	2	
				Total	100,00	,000	2	
			Total	left	100,00	,000	2	
				Total	100,00	,000	2	
		Total	76	left	100,00	,000	2	
				Total	100,00	,000	2	
			Total	left	100,00	,000	2	
				Total	100,00	,000	2	
	,176	m	85	left	90,00	,000	3	
				Total	90,00	,000	3	
			Total	left	90,00	,000	3	
				Total	90,00	,000	3	
		Total	85	left	90,00	,000	3	
				Total	90,00	,000	3	
			Total	left	90,00	,000	3	
				Total	90,00	,000	3	
	,177	m	85	left	100,00	,000	3	
				Total	100,00	,000	3	
			Total	left	100,00	,000	3	
				Total	100,00	,000	3	
		Total	85	left	100,00	,000	3	
				Total	100,00	,000	3	
			Total	left	100,00	,000	3	
				Total	100,00	,000	3	
	,180	f	64	righ	80,00	,000	2	
				Total	80,00	,000	2	
			Total	righ	80,00	,000	2	
				Total	80,00	,000	2	
		Total	64	righ	80,00	,000	2	
				Total	80,00	,000	2	
			Total	righ	80,00	,000	2	
				Total	80,00	,000	2	
	,181	f	80	left	90,00	,000	2	
				Total	90,00	,000	2	
			Total	left	90,00	,000	2	
				Total	90,00	,000	2	
		Total	80	left	90,00	,000	2	
				Total	90,00	,000	2	
			Total	left	90,00	,000	2	
				Total	90,00	,000	2	
	,183	f	85	righ	80,00	,000	3	
				Total	80,00	,000	3	
			Total	righ	80,00	,000	3	
				Total	80,00	,000	3	
		Total	85	righ	80,00	,000	3	
				Total	80,00	,000	3	
			Total	righ	80,00	,000	3	
				Total	80,00	,000	3	
	,185	m	71	righ	90,00	,000	3	
				Total	90,00	,000	3	
			Total	righ	90,00	,000	3	
				Total	90,00	,000	3	
		Total	71	righ	90,00	,000	3	
				Total	90,00	,000	3	
			Total	righ	90,00	,000	3	
				Total	90,00	,000	3	
	,188	m	78	righ	100,00	,000	3	
				Total	100,00	,000	3	
			Total	righ	100,00	,000	3	
				Total	100,00	,000	3	
		Total	78	righ	100,00	,000	3	
				Total	100,00	,000	3	
			Total	righ	100,00	,000	3	
				Total	100,00	,000	3	
	,188	m	81	righ	90,00	,000	3	
				Total	90,00	,000	3	
			Total	righ	90,00	,000	3	
				Total	90,00	,000	3	
		Total	81	righ	90,00	,000	3	
				Total	90,00	,000	3	
			Total	righ	90,00	,000	3	
				Total	90,00	,000	3	
	,189	m	85	righ	90,00	,000	3	
				Total	90,00	,000	3	
			Total	righ	90,00	,000	3	
				Total	90,00	,000	3	
		Total	85	righ	90,00	,000	3	
				Total	90,00	,000	3	
			Total	righ	90,00	,000	3	
				Total	90,00	,000	3	
	,190	f	81	righ	60,00	,000	2	
				Total	60,00	,000	2	
			Total	righ	60,00	,000	2	
				Total	60,00	,000	2	
		Total	81	righ	60,00	,000	2	
				Total	60,00	,000	2	
			Total	righ	60,00	,000	2	
				Total	60,00	,000	2	
	,191	f	79	left	80,00	,000	3	
				Total	80,00	,000	3	
			Total	left	80,00	,000	3	
				Total	80,00	,000	3	
		Total	79	left	80,00	,000	3	
				Total	80,00	,000	3	
			Total	left	80,00	,000	3	
				Total	80,00	,000	3	
	,192	f	78	left	100,00	,000	2	
				Total	100,00	,000	2	
			Total	left	100,00	,000	2	
				Total	100,00	,000	2	
		Total	78	left	100,00	,000	2	
				Total	100,00	,000	2	
			Total	left	100,00	,000	2	
				Total	100,00	,000	2	
	,192	f	71	left	90,00	,000	3	
				Total	90,00	,000	3	
			Total	left	90,00	,000	3	
				Total	90,00	,000	3	
		Total	71	left	90,00	,000	3	
				Total	90,00	,000	3	
			Total	left	90,00	,000	3	
				Total	90,00	,000	3	
	,194	m	85	left	100,00	,000	2	
				Total	100,00	,000	2	
			Total	left	100,00	,000	2	
				Total	100,00	,000	2	
		Total	85	left	100,00	,000	2	
				Total	100,00	,000	2	
			Total	left	100,00	,000	2	
				Total	100,00	,000	2	
	,198	m	84	righ	90,00	,000	2	
				Total	90,00	,000	2	
			Total	righ	90,00	,000	2	
				Total	90,00	,000	2	
		Total	84	righ	90,00	,000	2	
				Total	90,00	,000	2	
			Total	righ	90,00	,000	2	
				Total	90,00	,000	2	
	,198	m	90	left	90,00	,000	2	
				Total	90,00	,000	2	
			Total	left	90,00	,000	2	
				Total	90,00	,000	2	
		Total	90	left	90,00	,000	2	
				Total	90,00	,000	2	
			Total	left	90,00	,000	2	
				Total	90,00	,000	2	
	,200	m	80	left	90,00	,000	3	
				Total	90,00	,000	3	
			Total	left	90,00	,000	3	
				Total	90,00	,000	3	
		Total	80	left	90,00	,000	3	
				Total	90,00	,000	3	
			Total	left	90,00	,000	3	
				Total	90,00	,000	3	
	,200	m	78	left	90,00	,000	3	
				Total	90,00	,000	3	
			Total	left	90,00	,000	3	
				Total	90,00	,000	3	
		Total	78	left	90,00	,000	3	
				Total	90,00	,000	3	
			Total	left	90,00	,000	3	
				Total	90,00	,000	3	
	,201	m	96	left	60,00	,000	3	
				Total	60,00	,000	3	
			Total	left	60,00	,000	3	
				Total	60,00	,000	3	
		Total	96	left	60,00	,000	3	
				Total	60,00	,000	3	
			Total	left	60,00	,000	3	
				Total	60,00	,000	3	
	,207	m	83	left	80,00	,000	3	
				Total	80,00	,000	3	
			Total	left	80,00	,000	3	
				Total	80,00	,000	3	
		Total	83	left	80,00	,000	3	
				Total	80,00	,000	3	
			Total	left	80,00	,000	3	
				Total	80,00	,000	3	
	,208	f	83	righ	90,00	,000	3	
				Total	90,00	,000	3	
			Total	righ	90,00	,000	3	
				Total	90,00	,000	3	
		Total	83	righ	90,00	,000	3	
				Total	90,00	,000	3	
			Total	righ	90,00	,000	3	
				Total	90,00	,000	3	
	,210	m	82	left	50,00	,000	3	
				Total	50,00	,000	3	
			Total	left	50,00	,000	3	
				Total	50,00	,000	3	
		Total	82	left	50,00	,000	3	
				Total	50,00	,000	3	
			Total	left	50,00	,000	3	
				Total	50,00	,000	3	
	,211	m	85	left	70,00	,000	3	
				Total	70,00	,000	3	
			Total	left	70,00	,000	3	
				Total	70,00	,000	3	
		Total	85	left	70,00	,000	3	
				Total	70,00	,000	3	
			Total	left	70,00	,000	3	
				Total	70,00	,000	3	
	,213	m	82	left	90,00	,000	3	
				Total	90,00	,000	3	
			Total	left	90,00	,000	3	
				Total	90,00	,000	3	
		Total	82	left	90,00	,000	3	
				Total	90,00	,000	3	
			Total	left	90,00	,000	3	
				Total	90,00	,000	3	
	,213	m	82	righ	80,00	,000	2	
				Total	80,00	,000	2	
			Total	righ	80,00	,000	2	
				Total	80,00	,000	2	
		Total	82	righ	80,00	,000	2	
				Total	80,00	,000	2	
			Total	righ	80,00	,000	2	
				Total	80,00	,000	2	
	,214	m	85	righ	80,00	,000	3	
				Total	80,00	,000	3	
			Total	righ	80,00	,000	3	
				Total	80,00	,000	3	
		Total	85	righ	80,00	,000	3	
				Total	80,00	,000	3	
			Total	righ	80,00	,000	3	
				Total	80,00	,000	3	
	,219	f	71	righ	80,00	,000	3	
				Total	80,00	,000	3	
			Total	righ	80,00	,000	3	
				Total	80,00	,000	3	
		Total	71	righ	80,00	,000	3	
				Total	80,00	,000	3	
			Total	righ	80,00	,000	3	
				Total	80,00	,000	3	
	,221	f	74	righ	90,00	,000	2	
				Total	90,00	,000	2	
			Total	righ	90,00	,000	2	
				Total	90,00	,000	2	
		Total	74	righ	90,00	,000	2	
				Total	90,00	,000	2	
			Total	righ	90,00	,000	2	
				Total	90,00	,000	2	
	,224	m	85	righ	90,00	,000	2	
				Total	90,00	,000	2	
			Total	righ	90,00	,000	2	
				Total	90,00	,000	2	
		Total	85	righ	90,00	,000	2	
				Total	90,00	,000	2	
			Total	righ	90,00	,000	2	
				Total	90,00	,000	2	
	,224	f	97	righ	90,00	,000	3	
				Total	90,00	,000	3	
			Total	righ	90,00	,000	3	
				Total	90,00	,000	3	
		Total	97	righ	90,00	,000	3	
				Total	90,00	,000	3	
			Total	righ	90,00	,000	3	
				Total	90,00	,000	3	
	,226	f	82	left	90,00	,000	3	
				Total	90,00	,000	3	
			Total	left	90,00	,000	3	
				Total	90,00	,000	3	
		Total	82	left	90,00	,000	3	
				Total	90,00	,000	3	
			Total	left	90,00	,000	3	
				Total	90,00	,000	3	
	,226	f	83	righ	80,00	,000	3	
				Total	80,00	,000	3	
			Total	righ	80,00	,000	3	
				Total	80,00	,000	3	
		Total	83	righ	80,00	,000	3	
				Total	80,00	,000	3	
			Total	righ	80,00	,000	3	
				Total	80,00	,000	3	
	,228	m	87	righ	90,00	,000	3	
				Total	90,00	,000	3	
			Total	righ	90,00	,000	3	
				Total	90,00	,000	3	
		Total	87	righ	90,00	,000	3	
				Total	90,00	,000	3	
			Total	righ	90,00	,000	3	
				Total	90,00	,000	3	
	,254	m	85	left	70,00	,000	3	
				Total	70,00	,000	3	
			Total	left	70,00	,000	3	
				Total	70,00	,000	3	
		Total	85	left	70,00	,000	3	
				Total	70,00	,000	3	
			Total	left	70,00	,000	3	
				Total	70,00	,000	3	
	,265	m	85	righ	80,00	,000	3	
				Total	80,00	,000	3	
			Total	righ	80,00	,000	3	
				Total	80,00	,000	3	
		Total	85	righ	80,00	,000	3	
				Total	80,00	,000	3	
			Total	righ	80,00	,000	3	
				Total	80,00	,000	3	
	Total	f	60	left	90,00	,000	3	
				Total	90,00	,000	3	
			61	left	90,00	,000	3	
				Total	90,00	,000	3	
			63	righ	90,00	,000	3	
				Total	90,00	,000	3	
			64	righ	80,00	,000	2	
				Total	80,00	,000	2	
			71	left	90,00	,000	3	
				righ	80,00	,000	3	
				Total	85,00	5,477	6	
			73	righ	90,00	,000	2	
				Total	90,00	,000	2	
			74	righ	96,00	5,477	5	
				Total	96,00	5,477	5	
			78	left	94,00	5,477	5	
				Total	94,00	5,477	5	
			79	left	80,00	,000	3	
				righ	90,00	,000	3	
				Total	85,00	5,477	6	
			80	left	90,00	,000	5	
				righ	80,00	,000	3	
				Total	86,25	5,175	8	
			81	righ	78,00	16,432	5	
				Total	78,00	16,432	5	
			82	left	90,00	,000	3	
				Total	90,00	,000	3	
			83	righ	85,00	5,477	6	
				Total	85,00	5,477	6	
			84	left	80,00	,000	3	
				righ	81,25	8,345	8	
				Total	80,91	7,006	11	
			85	righ	80,00	,000	3	
				Total	80,00	,000	3	
			97	righ	90,00	,000	3	
				Total	90,00	,000	3	
			Total	left	88,57	5,245	28	
				righ	84,78	8,625	46	
				Total	86,22	7,711	74	
		m	62	righ	100,00	,000	2	
				Total	100,00	,000	2	
			63	righ	100,00	,000	2	
				Total	100,00	,000	2	
			66	left	90,00	,000	2	
				Total	90,00	,000	2	
			69	righ	90,00	,000	3	
				Total	90,00	,000	3	
			70	righ	90,00	,000	3	
				Total	90,00	,000	3	
			71	righ	90,00	,000	3	
				Total	90,00	,000	3	
			72	righ	95,00	5,477	6	
				Total	95,00	5,477	6	
			73	left	86,67	5,000	9	
				Total	86,67	5,000	9	
			75	left	90,00	,000	3	
				righ	80,00	,000	3	
				Total	85,00	5,477	6	
			76	left	94,00	5,477	5	
				righ	90,00	,000	2	
				Total	92,86	4,880	7	
			77	left	90,00	,000	5	
				Total	90,00	,000	5	
			78	left	90,00	,000	3	
				righ	96,00	5,477	5	
				Total	93,75	5,175	8	
			80	left	90,00	,000	3	
				righ	90,00	,000	2	
				Total	90,00	,000	5	
			81	righ	90,00	,000	3	
				Total	90,00	,000	3	
			82	left	70,00	21,909	6	
				righ	80,00	,000	2	
				Total	72,50	19,086	8	
			83	left	80,00	,000	3	
				Total	80,00	,000	3	
			84	righ	90,00	,000	2	
				Total	90,00	,000	2	
			85	left	84,12	12,776	17	
				righ	84,55	5,222	11	
				Total	84,29	10,338	28	
			87	righ	90,00	,000	3	
				Total	90,00	,000	3	
			88	righ	90,00	,000	3	
				Total	90,00	,000	3	
			90	left	90,00	,000	2	
				Total	90,00	,000	2	
			96	left	60,00	,000	3	
				Total	60,00	,000	3	
			Total	left	84,26	12,578	61	
				righ	89,82	6,233	55	
				Total	86,90	10,419	116	
		Total	60	left	90,00	,000	3	
				Total	90,00	,000	3	
			61	left	90,00	,000	3	
				Total	90,00	,000	3	
			62	righ	100,00	,000	2	
				Total	100,00	,000	2	
			63	righ	94,00	5,477	5	
				Total	94,00	5,477	5	
			64	righ	80,00	,000	2	
				Total	80,00	,000	2	
			66	left	90,00	,000	2	
				Total	90,00	,000	2	
			69	righ	90,00	,000	3	
				Total	90,00	,000	3	
			70	righ	90,00	,000	3	
				Total	90,00	,000	3	
			71	left	90,00	,000	3	
				righ	85,00	5,477	6	
				Total	86,67	5,000	9	
			72	righ	95,00	5,477	6	
				Total	95,00	5,477	6	
			73	left	86,67	5,000	9	
				righ	90,00	,000	2	
				Total	87,27	4,671	11	
			74	righ	96,00	5,477	5	
				Total	96,00	5,477	5	
			75	left	90,00	,000	3	
				righ	80,00	,000	3	
				Total	85,00	5,477	6	
			76	left	94,00	5,477	5	
				righ	90,00	,000	2	
				Total	92,86	4,880	7	
			77	left	90,00	,000	5	
				Total	90,00	,000	5	
			78	left	92,50	4,629	8	
				righ	96,00	5,477	5	
				Total	93,85	5,064	13	
			79	left	80,00	,000	3	
				righ	90,00	,000	3	
				Total	85,00	5,477	6	
			80	left	90,00	,000	8	
				righ	84,00	5,477	5	
				Total	87,69	4,385	13	
			81	righ	82,50	13,887	8	
				Total	82,50	13,887	8	
			82	left	76,67	20,000	9	
				righ	80,00	,000	2	
				Total	77,27	17,939	11	
			83	left	80,00	,000	3	
				righ	85,00	5,477	6	
				Total	83,33	5,000	9	
			84	left	80,00	,000	3	
				righ	83,00	8,233	10	
				Total	82,31	7,250	13	
			85	left	84,12	12,776	17	
				righ	83,57	4,972	14	
				Total	83,87	9,892	31	
			87	righ	90,00	,000	3	
				Total	90,00	,000	3	
			88	righ	90,00	,000	3	
				Total	90,00	,000	3	
			90	left	90,00	,000	2	
				Total	90,00	,000	2	
			96	left	60,00	,000	3	
				Total	60,00	,000	3	
			97	righ	90,00	,000	3	
				Total	90,00	,000	3	
			Total	left	85,62	10,971	89	
				righ	87,52	7,798	101	
				Total	86,63	9,440	190	


Multivariate Tests	
Effect	Value	F	Hypothesis df	Error df	Sig.	Partial Eta Squared	Noncent. Parameter	Observed Power	
Intercept	Pillai's Trace	,999	24494,801	4,000	117,000	,000	,999	97979,203	1,000	
	Wilks' Lambda	,001	24494,801	4,000	117,000	,000	,999	97979,203	1,000	
	Hotelling's Trace	837,429	24494,801	4,000	117,000	,000	,999	97979,203	1,000	
	Roy's Largest Root	837,429	24494,801	4,000	117,000	,000	,999	97979,203	1,000	
RCAindex	Pillai's Trace	2,306	7,423	88,000	480,000	,000	,576	653,202	1,000	
	Wilks' Lambda	,001	25,598	88,000	465,126	,000	,825	2198,318	1,000	
	Hotelling's Trace	84,137	110,430	88,000	462,000	,000	,955	9717,870	1,000	
	Roy's Largest Root	76,301	416,186	22,000	120,000	,000	,987	9156,098	1,000	
Sex	Pillai's Trace	,000	.	,000	,000	.	.	.	.	
	Wilks' Lambda	1,000	.	,000	118,500	.	.	.	.	
	Hotelling's Trace	,000	.	,000	2,000	.	.	.	.	
	Roy's Largest Root	,000	,000	4,000	116,000	1,000	,000	,000	,050	
Age	Pillai's Trace	,000	.	,000	,000	.	.	.	.	
	Wilks' Lambda	1,000	.	,000	118,500	.	.	.	.	
	Hotelling's Trace	,000	.	,000	2,000	.	.	.	.	
	Roy's Largest Root	,000	,000	4,000	116,000	1,000	,000	,000	,050	
Side	Pillai's Trace	,000	.	,000	,000	.	.	.	.	
	Wilks' Lambda	1,000	.	,000	118,500	.	.	.	.	
	Hotelling's Trace	,000	.	,000	2,000	.	.	.	.	
	Roy's Largest Root	,000	,000	4,000	116,000	1,000	,000	,000	,050	
RCAindex * Sex	Pillai's Trace	,000	.	,000	,000	.	.	.	.	
	Wilks' Lambda	1,000	.	,000	118,500	.	.	.	.	
	Hotelling's Trace	,000	.	,000	2,000	.	.	.	.	
	Roy's Largest Root	,000	,000	4,000	116,000	1,000	,000	,000	,050	
RCAindex * Age	Pillai's Trace	,000	.	,000	,000	.	.	.	.	
	Wilks' Lambda	1,000	.	,000	118,500	.	.	.	.	
	Hotelling's Trace	,000	.	,000	2,000	.	.	.	.	
	Roy's Largest Root	,000	,000	4,000	116,000	1,000	,000	,000	,050	
RCAindex * Side	Pillai's Trace	,000	.	,000	,000	.	.	.	.	
	Wilks' Lambda	1,000	.	,000	118,500	.	.	.	.	
	Hotelling's Trace	,000	.	,000	2,000	.	.	.	.	
	Roy's Largest Root	,000	,000	4,000	116,000	1,000	,000	,000	,050	
Sex * Age	Pillai's Trace	,000	.	,000	,000	.	.	.	.	
	Wilks' Lambda	1,000	.	,000	118,500	.	.	.	.	
	Hotelling's Trace	,000	.	,000	2,000	.	.	.	.	
	Roy's Largest Root	,000	,000	4,000	116,000	1,000	,000	,000	,050	
Sex * Side	Pillai's Trace	,000	.	,000	,000	.	.	.	.	
	Wilks' Lambda	1,000	.	,000	118,500	.	.	.	.	
	Hotelling's Trace	,000	.	,000	2,000	.	.	.	.	
	Roy's Largest Root	,000	,000	4,000	116,000	1,000	,000	,000	,050	
Age * Side	Pillai's Trace	,000	.	,000	,000	.	.	.	.	
	Wilks' Lambda	1,000	.	,000	118,500	.	.	.	.	
	Hotelling's Trace	,000	.	,000	2,000	.	.	.	.	
	Roy's Largest Root	,000	,000	4,000	116,000	1,000	,000	,000	,050	
RCAindex * Sex * Age	Pillai's Trace	,000	.	,000	,000	.	.	.	.	
	Wilks' Lambda	1,000	.	,000	118,500	.	.	.	.	
	Hotelling's Trace	,000	.	,000	2,000	.	.	.	.	
	Roy's Largest Root	,000	,000	4,000	116,000	1,000	,000	,000	,050	
RCAindex * Sex * Side	Pillai's Trace	,000	.	,000	,000	.	.	.	.	
	Wilks' Lambda	1,000	.	,000	118,500	.	.	.	.	
	Hotelling's Trace	,000	.	,000	2,000	.	.	.	.	
	Roy's Largest Root	,000	,000	4,000	116,000	1,000	,000	,000	,050	
RCAindex * Age * Side	Pillai's Trace	,000	.	,000	,000	.	.	.	.	
	Wilks' Lambda	1,000	.	,000	118,500	.	.	.	.	
	Hotelling's Trace	,000	.	,000	2,000	.	.	.	.	
	Roy's Largest Root	,000	,000	4,000	116,000	1,000	,000	,000	,050	
Sex * Age * Side	Pillai's Trace	,000	.	,000	,000	.	.	.	.	
	Wilks' Lambda	1,000	.	,000	118,500	.	.	.	.	
	Hotelling's Trace	,000	.	,000	2,000	.	.	.	.	
	Roy's Largest Root	,000	,000	4,000	116,000	1,000	,000	,000	,050	
RCAindex * Sex * Age * Side	Pillai's Trace	,000	.	,000	,000	.	.	.	.	
	Wilks' Lambda	1,000	.	,000	118,500	.	.	.	.	
	Hotelling's Trace	,000	.	,000	2,000	.	.	.	.	
	Roy's Largest Root	,000	,000	4,000	116,000	1,000	,000	,000	,050	


Tests of Between-Subjects Effects	
Source	Dependent Variable	Type III Sum of Squares	df	Mean Square	F	Sig.	Partial Eta Squared	Noncent. Parameter	Observed Power	
Corrected Model	MDPreop	5464,227	69	79,192	.	.	1,000	.	.	
	MDPost 30	5331,706	69	77,271	74,678	,000	,977	5152,790	1,000	
	MDPost 90	1278,709	69	18,532	3,832	,000	,688	264,390	1,000	
	Shift pre	2481,228	69	35,960	.	.	1,000	.	.	
	Shift post 30	1186,010	69	17,189	375,023	,000	,995	25876,575	1,000	
	Shift post 90	130,216	69	1,887	1,272	,124	,422	87,786	,993	
	KPS PreOp	39255,263	69	568,917	.	.	1,000	.	.	
	KPS PostOp	16844,211	69	244,119	.	.	1,000	.	.	
Intercept	MDPreop	84942,170	1	84942,170	.	.	1,000	.	.	
	MDPost 30	17776,283	1	17776,283	17179,763	,000	,993	17179,763	1,000	
	MDPost 90	2722,629	1	2722,629	562,940	,000	,824	562,940	1,000	
	Shift pre	13872,469	1	13872,469	.	.	1,000	.	.	
	Shift post 30	1747,812	1	1747,812	38134,076	,000	,997	38134,076	1,000	
	Shift post 90	572,094	1	572,094	385,682	,000	,763	385,682	1,000	
	KPS PreOp	537683,965	1	537683,965	.	.	1,000	.	.	
	KPS PostOp	1227413,591	1	1227413,591	.	.	1,000	.	.	
RCAindex	MDPreop	2494,110	22	113,369	.	.	1,000	.	.	
	MDPost 30	1478,644	22	67,211	64,956	,000	,923	1429,025	1,000	
	MDPost 90	336,977	22	15,317	3,167	,000	,367	69,675	1,000	
	Shift pre	611,478	22	27,794	.	.	1,000	.	.	
	Shift post 30	239,297	22	l	237,319	,000	,978	5221,029	1,000	
	Shift post 90	40,242	22	1,829	1,233	,234	,184	27,130	,844	
	KPS PreOp	19904,439	22	904,747	.	.	1,000	.	.	
	KPS PostOp	7831,992	22	356,000	.	.	1,000	.	.	
Sex	MDPreop	,000	0	.	.	.	.	.	.	
	MDPost 30	,000	0	.	.	.	,000	,000	.	
	MDPost 90	,000	0	.	.	.	,000	,000	.	
	Shift pre	,000	0	.	.	.	.	.	.	
	Shift post 30	,000	0	.	.	.	,000	,000	.	
	Shift post 90	,000	0	.	.	.	,000	,000	.	
	KPS PreOp	,000	0	.	.	.	.	.	.	
	KPS PostOp	,000	0	.	.	.	.	.	.	
Age	MDPreop	,000	0	.	.	.	.	.	.	
	MDPost 30	,000	0	.	.	.	,000	,000	.	
	MDPost 90	,000	0	.	.	.	,000	,000	.	
	Shift pre	,000	0	.	.	.	.	.	.	
	Shift post 30	,000	0	.	.	.	,000	,000	.	
	Shift post 90	,000	0	.	.	.	,000	,000	.	
	KPS PreOp	,000	0	.	.	.	.	.	.	
	KPS PostOp	,000	0	.	.	.	.	.	.	
Side	MDPreop	,000	0	.	.	.	.	.	.	
	MDPost 30	,000	0	.	.	.	,000	,000	.	
	MDPost 90	,000	0	.	.	.	,000	,000	.	
	Shift pre	,000	0	.	.	.	.	.	.	
	Shift post 30	,000	0	.	.	.	,000	,000	.	
	Shift post 90	,000	0	.	.	.	,000	,000	.	
	KPS PreOp	,000	0	.	.	.	.	.	.	
	KPS PostOp	,000	0	.	.	.	.	.	.	
RCAindex * Sex	MDPreop	,000	0	.	.	.	.	.	.	
	MDPost 30	,000	0	.	.	.	,000	,000	.	
	MDPost 90	,000	0	.	.	.	,000	,000	.	
	Shift pre	,000	0	.	.	.	.	.	.	
	Shift post 30	,000	0	.	.	.	,000	,000	.	
	Shift post 90	,000	0	.	.	.	,000	,000	.	
	KPS PreOp	,000	0	.	.	.	.	.	.	
	KPS PostOp	,000	0	.	.	.	.	.	.	
RCAindex * Age	MDPreop	,000	0	.	.	.	.	.	.	
	MDPost 30	,000	0	.	.	.	,000	,000	.	
	MDPost 90	,000	0	.	.	.	,000	,000	.	
	Shift pre	,000	0	.	.	.	.	.	.	
	Shift post 30	,000	0	.	.	.	,000	,000	.	
	Shift post 90	,000	0	.	.	.	,000	,000	.	
	KPS PreOp	,000	0	.	.	.	.	.	.	
	KPS PostOp	,000	0	.	.	.	.	.	.	
RCAindex * Side	MDPreop	,000	0	.	.	.	.	.	.	
	MDPost 30	,000	0	.	.	.	,000	,000	.	
	MDPost 90	,000	0	.	.	.	,000	,000	.	
	Shift pre	,000	0	.	.	.	.	.	.	
	Shift post 30	,000	0	.	.	.	,000	,000	.	
	Shift post 90	,000	0	.	.	.	,000	,000	.	
	KPS PreOp	,000	0	.	.	.	.	.	.	
	KPS PostOp	,000	0	.	.	.	.	.	.	
Sex * Age	MDPreop	,000	0	.	.	.	.	.	.	
	MDPost 30	,000	0	.	.	.	,000	,000	.	
	MDPost 90	,000	0	.	.	.	,000	,000	.	
	Shift pre	,000	0	.	.	.	.	.	.	
	Shift post 30	,000	0	.	.	.	,000	,000	.	
	Shift post 90	,000	0	.	.	.	,000	,000	.	
	KPS PreOp	,000	0	.	.	.	.	.	.	
	KPS PostOp	,000	0	.	.	.	.	.	.	
Sex * Side	MDPreop	,000	0	.	.	.	.	.	.	
	MDPost 30	,000	0	.	.	.	,000	,000	.	
	MDPost 90	,000	0	.	.	.	,000	,000	.	
	Shift pre	,000	0	.	.	.	.	.	.	
	Shift post 30	,000	0	.	.	.	,000	,000	.	
	Shift post 90	,000	0	.	.	.	,000	,000	.	
	KPS PreOp	,000	0	.	.	.	.	.	.	
	KPS PostOp	,000	0	.	.	.	.	.	.	
Age * Side	MDPreop	,000	0	.	.	.	.	.	.	
	MDPost 30	,000	0	.	.	.	,000	,000	.	
	MDPost 90	,000	0	.	.	.	,000	,000	.	
	Shift pre	,000	0	.	.	.	.	.	.	
	Shift post 30	,000	0	.	.	.	,000	,000	.	
	Shift post 90	,000	0	.	.	.	,000	,000	.	
	KPS PreOp	,000	0	.	.	.	.	.	.	
	KPS PostOp	,000	0	.	.	.	.	.	.	
RCAindex * Sex * Age	MDPreop	,000	0	.	.	.	.	.	.	
	MDPost 30	,000	0	.	.	.	,000	,000	.	
	MDPost 90	,000	0	.	.	.	,000	,000	.	
	Shift pre	,000	0	.	.	.	.	.	.	
	Shift post 30	,000	0	.	.	.	,000	,000	.	
	Shift post 90	,000	0	.	.	.	,000	,000	.	
	KPS PreOp	,000	0	.	.	.	.	.	.	
	KPS PostOp	,000	0	.	.	.	.	.	.	
RCAindex * Sex * Side	MDPreop	,000	0	.	.	.	.	.	.	
	MDPost 30	,000	0	.	.	.	,000	,000	.	
	MDPost 90	,000	0	.	.	.	,000	,000	.	
	Shift pre	,000	0	.	.	.	.	.	.	
	Shift post 30	,000	0	.	.	.	,000	,000	.	
	Shift post 90	,000	0	.	.	.	,000	,000	.	
	KPS PreOp	,000	0	.	.	.	.	.	.	
	KPS PostOp	,000	0	.	.	.	.	.	.	
RCAindex * Age * Side	MDPreop	,000	0	.	.	.	.	.	.	
	MDPost 30	,000	0	.	.	.	,000	,000	.	
	MDPost 90	,000	0	.	.	.	,000	,000	.	
	Shift pre	,000	0	.	.	.	.	.	.	
	Shift post 30	,000	0	.	.	.	,000	,000	.	
	Shift post 90	,000	0	.	.	.	,000	,000	.	
	KPS PreOp	,000	0	.	.	.	.	.	.	
	KPS PostOp	,000	0	.	.	.	.	.	.	
Sex * Age * Side	MDPreop	,000	0	.	.	.	.	.	.	
	MDPost 30	,000	0	.	.	.	,000	,000	.	
	MDPost 90	,000	0	.	.	.	,000	,000	.	
	Shift pre	,000	0	.	.	.	.	.	.	
	Shift post 30	,000	0	.	.	.	,000	,000	.	
	Shift post 90	,000	0	.	.	.	,000	,000	.	
	KPS PreOp	,000	0	.	.	.	.	.	.	
	KPS PostOp	,000	0	.	.	.	.	.	.	
RCAindex * Sex * Age * Side	MDPreop	,000	0	.	.	.	.	.	.	
	MDPost 30	,000	0	.	.	.	,000	,000	.	
	MDPost 90	,000	0	.	.	.	,000	,000	.	
	Shift pre	,000	0	.	.	.	.	.	.	
	Shift post 30	,000	0	.	.	.	,000	,000	.	
	Shift post 90	,000	0	.	.	.	,000	,000	.	
	KPS PreOp	,000	0	.	.	.	.	.	.	
	KPS PostOp	,000	0	.	.	.	.	.	.	
Error	MDPreop	,000	120	,000						
	MDPost 30	124,167	120	1,035						
	MDPost 90	580,374	120	4,836						
	Shift pre	,000	120	,000						
	Shift post 30	5,500	120	,046						
	Shift post 90	178,000	120	1,483						
	KPS PreOp	,000	120	,000						
	KPS PostOp	,000	120	,000						
Total	MDPreop	105643,300	190							
	MDPost 30	27138,410	190							
	MDPost 90	5385,784	190							
	Shift pre	17754,450	190							
	Shift post 30	3276,480	190							
	Shift post 90	979,000	190							
	KPS PreOp	681900,000	190							
	KPS PostOp	1442800,000	190							
Corrected Total	MDPreop	5464,227	189							
	MDPost 30	5455,873	189							
	MDPost 90	1859,082	189							
	Shift pre	2481,228	189							
	Shift post 30	1191,510	189							
	Shift post 90	308,216	189							
	KPS PreOp	39255,263	189							
	KPS PostOp	16844,211	189							


Any changes made to the working file since 09-AUG-2021 16:02:47 have been lost.
The time now is 16:20:57.
