# Supplementary material for: Cortical atrophy in chronic subdural hematoma from ultra-structures to physical properties
Source: Sci Rep. 2023 Feb 28;13:3400. doi: 10.1038/s41598-023-30135-8 (PMC9975247; doi:10.1038/s41598-023-30135-8)
Supplement: Supplementary file 10 — Supplementary Information 10. [file 41598_2023_30135_MOESM10_ESM.doc]

GET
  FILE='C:\Users\Placido\Desktop\articolo atrofia e sottodurale cronico\controlli\controlli correlazioni.sav'.
DATASET NAME Dataset1 WINDOW=FRONT.
SET TLook=None Small=0.0001 SUMMARY=None THREADS=AUTO TFit=Both DIGITGROUPING=No LEADZERO=No TABLERENDER=light.
BAYES ONESAMPLE
  /MISSING SCOPE=ANALYSIS
  /CRITERIA CILEVEL=95
  /INFERENCE DISTRIBUTION=NORMAL VARIABLES=RCAindex ANALYSIS=POSTERIOR
  /PRIOR VARDIST=DIFFUSE MEANDIST=DIFFUSE.


Bayesian One-Sample


Notes	
Output Created	18-JUL-2021 22:38:33	
Comments		
Input	Data	C:\Users\Placido\Desktop\articolo atrofia e sottodurale cronico\controlli\controlli correlazioni.sav	
	Active Dataset	Dataset1	
	Filter	<none>	
	Weight	<none>	
	Split File	<none>	
	N of Rows in Working Data File	190	
Missing Value Handling	Definition of Missing	User-defined missing values are treated as missing.	
	Cases Used	Each statistic is based on all valid data for the analysis variable(s) used in computing the statistic.	
Weight Handling	not applicable	
Syntax	BAYES ONESAMPLE
  /MISSING SCOPE=ANALYSIS
  /CRITERIA CILEVEL=95
  /INFERENCE DISTRIBUTION=NORMAL VARIABLES=RCAindex ANALYSIS=POSTERIOR
  /PRIOR VARDIST=DIFFUSE MEANDIST=DIFFUSE.	
Resources	Processor Time	00:00:00,27	
	Elapsed Time	00:00:00,39	


[Dataset1] C:\Users\Placido\Desktop\articolo atrofia e sottodurale cronico\controlli\controlli correlazioni.sav


Posterior Distribution Characterization for One-Sample Mean	
	N	Posterior	95% Credible Interval	
		Mode	Mean	Variance	Lower Bound	Upper Bound	
RCA index	190	,137163595834819	,137163595834819	,000	,130640063672186	,143687127997452	


REGRESSION
  /MISSING LISTWISE
  /STATISTICS COEFF OUTS R ANOVA
  /CRITERIA=PIN(.05) POUT(.10)
  /NOORIGIN
  /DEPENDENT RCAindex
  /METHOD=FORWARD Age
  /PARTIALPLOT ALL
  /SCATTERPLOT=(*ZPRED ,*ZRESID).


REGRESSION
  /MISSING LISTWISE
  /STATISTICS COEFF OUTS R ANOVA
  /CRITERIA=PIN(.05) POUT(.10)
  /NOORIGIN
  /DEPENDENT RCAindex
  /METHOD=FORWARD Age
  /PARTIALPLOT ALL
  /SCATTERPLOT=(*ZPRED ,*ZRESID)
  /RESIDUALS HISTOGRAM(ZRESID) NORMPROB(ZRESID).


Regression


Notes	
Output Created	18-JUL-2021 22:41:04	
Comments		
Input	Data	C:\Users\Placido\Desktop\articolo atrofia e sottodurale cronico\controlli\controlli correlazioni.sav	
	Active Dataset	Dataset1	
	Filter	<none>	
	Weight	<none>	
	Split File	<none>	
	N of Rows in Working Data File	190	
Missing Value Handling	Definition of Missing	User-defined missing values are treated as missing.	
	Cases Used	Statistics are based on cases with no missing values for any variable used.	
Syntax	REGRESSION
  /MISSING LISTWISE
  /STATISTICS COEFF OUTS R ANOVA
  /CRITERIA=PIN(.05) POUT(.10)
  /NOORIGIN
  /DEPENDENT RCAindex
  /METHOD=FORWARD Age
  /PARTIALPLOT ALL
  /SCATTERPLOT=(*ZPRED ,*ZRESID)
  /RESIDUALS HISTOGRAM(ZRESID) NORMPROB(ZRESID).	
Resources	Processor Time	00:00:01,22	
	Elapsed Time	00:00:00,58	
	Memory Required	2688 bytes	
	Additional Memory Required for Residual Plots	808 bytes	


Variables Entered/Removed	
Model	Variables Entered	Variables Removed	Method	
1	Age	.	Forward (Criterion: Probability-of-F-to-enter <= ,050)	


Model Summary	
Model	R	R Square	Adjusted R Square	Std. Error of the Estimate	
1	,850	,722	,720	,023980286223880	


ANOVA	
Model	Sum of Squares	df	Mean Square	F	Sig.	
1	Regression	,280	1	,280	487,642	,000	
	Residual	,108	188	,001			
	Total	,389	189				


Coefficients	
Model	Unstandardized Coefficients	Standardized Coefficients	t	Sig.	
	B	Std. Error	Beta			
1	(Constant)	,011	,006		1,760	,080	
	Age	,002	,000	,850	22,083	,000	


Residuals Statistics	
	Minimum	Maximum	Mean	Std. Deviation	N	
Predicted Value	,040701359510422	,199531778693199	,137163595834819	,038518894017303	190	
Residual	-,061785835772753	,091619379818439	,000000000000000	,023916762175275	190	
Std. Predicted Value	-2,504	1,619	,000	1,000	190	
Std. Residual	-2,577	3,821	,000	,997	190	


Charts


BOOTSTRAP
  /SAMPLING METHOD=SIMPLE
  /VARIABLES INPUT=RCAindex Age
  /CRITERIA CILEVEL=95 CITYPE=PERCENTILE  NSAMPLES=1000
  /MISSING USERMISSING=EXCLUDE.


Bootstrap


Notes	
Output Created	18-JUL-2021 22:42:39	
Comments		
Input	Data	C:\Users\Placido\Desktop\articolo atrofia e sottodurale cronico\controlli\controlli correlazioni.sav	
	Active Dataset	Dataset1	
	Filter	<none>	
	Weight	<none>	
	Split File	<none>	
	N of Rows in Working Data File	190	
Syntax	BOOTSTRAP
  /SAMPLING METHOD=SIMPLE
  /VARIABLES INPUT=RCAindex Age
  /CRITERIA CILEVEL=95 CITYPE=PERCENTILE  NSAMPLES=1000
  /MISSING USERMISSING=EXCLUDE.	
Resources	Processor Time	00:00:00,02	
	Elapsed Time	00:00:00,02	


Bootstrap Specifications	
Sampling Method	Simple	
Number of Samples	1000	
Confidence Interval Level	95,0%	
Confidence Interval Type	Percentile	

CORRELATIONS
  /VARIABLES=RCAindex Age
  /PRINT=TWOTAIL NOSIG
  /STATISTICS DESCRIPTIVES XPROD
  /MISSING=PAIRWISE.


Correlations


Notes	
Output Created	18-JUL-2021 22:42:40	
Comments		
Input	Data	C:\Users\Placido\Desktop\articolo atrofia e sottodurale cronico\controlli\controlli correlazioni.sav	
	Active Dataset	Dataset1	
	Filter	<none>	
	Weight	<none>	
	Split File	<none>	
	N of Rows in Working Data File	120361	
Missing Value Handling	Definition of Missing	User-defined missing values are treated as missing.	
	Cases Used	Statistics for each pair of variables are based on all the cases with valid data for that pair.	
Syntax	CORRELATIONS
  /VARIABLES=RCAindex Age
  /PRINT=TWOTAIL NOSIG
  /STATISTICS DESCRIPTIVES XPROD
  /MISSING=PAIRWISE.	
Resources	Processor Time	00:00:02,73	
	Elapsed Time	00:00:02,22	


Descriptive Statistics	
	Statistic	Bootstrap	
		Bias	Std. Error	95% Confidence Interval	
				Lower	
RCA index	Mean	,137163595834819	-,000025021194333	,003171772426963	,131172682623803	
	Std. Deviation	,045340012232738	-,000189754069455	,001846708468100	,041734720385718	
	N	190	0	0	190	
Age	Mean	62,98	-,03	1,33	60,38	
	Std. Deviation	19,159	-,063	,774	17,494	
	N	190	0	0	190	

Descriptive Statistics	
	Bootstrap	
	95% Confidence Interval	
	Upper	
RCA index	Mean	,143615129879578	
	Std. Deviation	,048767762433792	
	N	190	
Age	Mean	65,53	
	Std. Deviation	20,635	
	N	190	


Correlations	
	RCA index	Age	
RCA index	Pearson Correlation	1	,850	
	Sig. (2-tailed)		,000	
	Sum of Squares and Cross-products	,389	139,477	
	Covariance	,002	,738	
	N	190	190	
	Bootstrap	Bias	0	,001	
		Std. Error	0	,017	
		95% Confidence Interval	Lower	1	,816	
			Upper	1	,882	
Age	Pearson Correlation	,850	1	
	Sig. (2-tailed)	,000		
	Sum of Squares and Cross-products	139,477	69373,916	
	Covariance	,738	367,058	
	N	190	190	
	Bootstrap	Bias	,001	0	
		Std. Error	,017	0	
		95% Confidence Interval	Lower	,816	1	
			Upper	,882	1	

* Builder di grafico.
GGRAPH
  /GRAPHDATASET NAME="graphdataset" VARIABLES=RCAindex Age MISSING=LISTWISE REPORTMISSING=NO
  /GRAPHSPEC SOURCE=INLINE
  /FITLINE TOTAL=YES.
BEGIN GPL
  SOURCE: s=userSource(id("graphdataset"))
  DATA: RCAindex=col(source(s), name("RCAindex"))
  DATA: Age=col(source(s), name("Age"))
  GUIDE: axis(dim(1), label("RCA index"))
  GUIDE: axis(dim(2), label("Age"))
  GUIDE: text.title(label("Dispersione semplice con curva di adattamento di Age per RCA index"))
  ELEMENT: point(position(RCAindex*Age))
END GPL.


SET OLang=English Unicode=Yes Locale=Italian Small=0.0001 THREADS=AUTO Printback=On BASETEXTDIRECTION=AUTOMATIC DIGITGROUPING=No TLook=None SUMMARY=None MIOUTPUT=[observed imputed pooled diagnostics] TFit=Both LEADZERO=No TABLERENDER=light.
* Builder di grafico.
GGRAPH
  /GRAPHDATASET NAME="graphdataset" VARIABLES=RCAindex Age MISSING=LISTWISE REPORTMISSING=NO
  /GRAPHSPEC SOURCE=INLINE
  /FITLINE TOTAL=YES.
BEGIN GPL
  SOURCE: s=userSource(id("graphdataset"))
  DATA: RCAindex=col(source(s), name("RCAindex"))
  DATA: Age=col(source(s), name("Age"))
  GUIDE: axis(dim(1), label("RCA index"))
  GUIDE: axis(dim(2), label("Age"))
  GUIDE: text.title(label("Dispersione semplice con curva di adattamento di Age per RCA index"))
  ELEMENT: point(position(RCAindex*Age))
END GPL.


GGraph


Notes	
Output Created	18-JUL-2021 22:52:42	
Comments		
Input	Data	C:\Users\Placido\Desktop\articolo atrofia e sottodurale cronico\controlli\controlli correlazioni.sav	
	Active Dataset	Dataset1	
	Filter	<none>	
	Weight	<none>	
	Split File	<none>	
	N of Rows in Working Data File	190	
Syntax	GGRAPH
  /GRAPHDATASET NAME="graphdataset" VARIABLES=RCAindex Age MISSING=LISTWISE REPORTMISSING=NO
  /GRAPHSPEC SOURCE=INLINE
  /FITLINE TOTAL=YES.
BEGIN GPL
  SOURCE: s=userSource(id("graphdataset"))
  DATA: RCAindex=col(source(s), name("RCAindex"))
  DATA: Age=col(source(s), name("Age"))
  GUIDE: axis(dim(1), label("RCA index"))
  GUIDE: axis(dim(2), label("Age"))
  GUIDE: text.title(label("Dispersione semplice con curva di adattamento di Age per RCA index"))
  ELEMENT: point(position(RCAindex*Age))
END GPL.	
Resources	Processor Time	00:00:00,19	
	Elapsed Time	00:00:00,14	


BAYES ONESAMPLE
  /MISSING SCOPE=ANALYSIS
  /CRITERIA CILEVEL=95
  /INFERENCE DISTRIBUTION=NORMAL VARIABLES=RCAindex ANALYSIS=POSTERIOR
  /PRIOR VARDIST=DIFFUSE MEANDIST=DIFFUSE.


Bayesian One-Sample


Notes	
Output Created	18-JUL-2021 23:38:12	
Comments		
Input	Data	C:\Users\Placido\Desktop\articolo atrofia e sottodurale cronico\controlli\controlli correlazioni.sav	
	Active Dataset	Dataset1	
	Filter	<none>	
	Weight	<none>	
	Split File	<none>	
	N of Rows in Working Data File	190	
Missing Value Handling	Definition of Missing	User-defined missing values are treated as missing.	
	Cases Used	Each statistic is based on all valid data for the analysis variable(s) used in computing the statistic.	
Weight Handling	not applicable	
Syntax	BAYES ONESAMPLE
  /MISSING SCOPE=ANALYSIS
  /CRITERIA CILEVEL=95
  /INFERENCE DISTRIBUTION=NORMAL VARIABLES=RCAindex ANALYSIS=POSTERIOR
  /PRIOR VARDIST=DIFFUSE MEANDIST=DIFFUSE.	
Resources	Processor Time	00:00:00,22	
	Elapsed Time	00:00:00,43	


Posterior Distribution Characterization for One-Sample Mean	
	N	Posterior	95% Credible Interval	
		Mode	Mean	Variance	Lower Bound	Upper Bound	
RCA index	190	,13716	,13716	,000	,13064	,14369	


REGRESSION
  /MISSING LISTWISE
  /STATISTICS COEFF OUTS R ANOVA
  /CRITERIA=PIN(.05) POUT(.10)
  /NOORIGIN
  /DEPENDENT RCAindex
  /METHOD=FORWARD Age
  /SCATTERPLOT=(*ZPRED ,*ZRESID)
  /RESIDUALS HISTOGRAM(ZRESID) NORMPROB(ZRESID).


Regression


Notes	
Output Created	18-JUL-2021 23:41:12	
Comments		
Input	Data	C:\Users\Placido\Desktop\articolo atrofia e sottodurale cronico\controlli\controlli correlazioni.sav	
	Active Dataset	Dataset1	
	Filter	<none>	
	Weight	<none>	
	Split File	<none>	
	N of Rows in Working Data File	190	
Missing Value Handling	Definition of Missing	User-defined missing values are treated as missing.	
	Cases Used	Statistics are based on cases with no missing values for any variable used.	
Syntax	REGRESSION
  /MISSING LISTWISE
  /STATISTICS COEFF OUTS R ANOVA
  /CRITERIA=PIN(.05) POUT(.10)
  /NOORIGIN
  /DEPENDENT RCAindex
  /METHOD=FORWARD Age
  /SCATTERPLOT=(*ZPRED ,*ZRESID)
  /RESIDUALS HISTOGRAM(ZRESID) NORMPROB(ZRESID).	
Resources	Processor Time	00:00:00,47	
	Elapsed Time	00:00:00,41	
	Memory Required	2688 bytes	
	Additional Memory Required for Residual Plots	680 bytes	


Variables Entered/Removed	
Model	Variables Entered	Variables Removed	Method	
1	Age	.	Forward (Criterion: Probability-of-F-to-enter <= ,050)	


Model Summary	
Model	R	R Square	Adjusted R Square	Std. Error of the Estimate	
1	,850	,722	,720	,023980	


ANOVA	
Model	Sum of Squares	df	Mean Square	F	Sig.	
1	Regression	,280	1	,280	487,642	,000	
	Residual	,108	188	,001			
	Total	,389	189				


Coefficients	
Model	Unstandardized Coefficients	Standardized Coefficients	t	Sig.	
	B	Std. Error	Beta			
1	(Constant)	,011	,006		1,760	,080	
	Age	,002	,000	,850	22,083	,000	


Residuals Statistics	
	Minimum	Maximum	Mean	Std. Deviation	N	
Predicted Value	,04070	,19953	,13716	,038519	190	
Residual	-,061786	,091619	,000000	,023917	190	
Std. Predicted Value	-2,504	1,619	,000	1,000	190	
Std. Residual	-2,577	3,821	,000	,997	190	


Charts


FREQUENCIES VARIABLES=RCAindex
  /ORDER=ANALYSIS.


Frequencies


Notes	
Output Created	18-JUL-2021 23:44:05	
Comments		
Input	Data	C:\Users\Placido\Desktop\articolo atrofia e sottodurale cronico\controlli\controlli correlazioni.sav	
	Active Dataset	Dataset1	
	Filter	<none>	
	Weight	<none>	
	Split File	<none>	
	N of Rows in Working Data File	190	
Missing Value Handling	Definition of Missing	User-defined missing values are treated as missing.	
	Cases Used	Statistics are based on all cases with valid data.	
Syntax	FREQUENCIES VARIABLES=RCAindex
  /ORDER=ANALYSIS.	
Resources	Processor Time	00:00:00,02	
	Elapsed Time	00:00:00,02	


Statistics	
RCA index  	
N	Valid	190	
	Missing	0	


RCA index	
	Frequency	Percent	Valid Percent	Cumulative Percent	
Valid	,053	1	,5	,5	,5	
	,059	1	,5	,5	1,1	
	,061	1	,5	,5	1,6	
	,068	1	,5	,5	2,1	
	,069	1	,5	,5	2,6	
	,071	1	,5	,5	3,2	
	,071	1	,5	,5	3,7	
	,073	1	,5	,5	4,2	
	,073	1	,5	,5	4,7	
	,076	1	,5	,5	5,3	
	,076	1	,5	,5	5,8	
	,078	1	,5	,5	6,3	
	,079	1	,5	,5	6,8	
	,080	1	,5	,5	7,4	
	,080	1	,5	,5	7,9	
	,080	1	,5	,5	8,4	
	,081	1	,5	,5	8,9	
	,081	1	,5	,5	9,5	
	,082	1	,5	,5	10,0	
	,082	1	,5	,5	10,5	
	,082	1	,5	,5	11,1	
	,082	1	,5	,5	11,6	
	,083	1	,5	,5	12,1	
	,083	1	,5	,5	12,6	
	,084	1	,5	,5	13,2	
	,084	1	,5	,5	13,7	
	,085	1	,5	,5	14,2	
	,086	1	,5	,5	14,7	
	,086	1	,5	,5	15,3	
	,089	1	,5	,5	15,8	
	,090	1	,5	,5	16,3	
	,091	1	,5	,5	16,8	
	,091	1	,5	,5	17,4	
	,091	1	,5	,5	17,9	
	,092	1	,5	,5	18,4	
	,092	1	,5	,5	18,9	
	,092	1	,5	,5	19,5	
	,093	1	,5	,5	20,0	
	,093	1	,5	,5	20,5	
	,093	1	,5	,5	21,1	
	,093	1	,5	,5	21,6	
	,094	1	,5	,5	22,1	
	,095	1	,5	,5	22,6	
	,096	1	,5	,5	23,2	
	,097	1	,5	,5	23,7	
	,098	1	,5	,5	24,2	
	,099	1	,5	,5	24,7	
	,099	1	,5	,5	25,3	
	,099	1	,5	,5	25,8	
	,101	1	,5	,5	26,3	
	,102	1	,5	,5	26,8	
	,102	1	,5	,5	27,4	
	,102	1	,5	,5	27,9	
	,103	1	,5	,5	28,4	
	,103	1	,5	,5	28,9	
	,104	1	,5	,5	29,5	
	,104	1	,5	,5	30,0	
	,104	1	,5	,5	30,5	
	,105	1	,5	,5	31,1	
	,105	1	,5	,5	31,6	
	,106	1	,5	,5	32,1	
	,106	1	,5	,5	32,6	
	,106	1	,5	,5	33,2	
	,107	1	,5	,5	33,7	
	,109	1	,5	,5	34,2	
	,110	1	,5	,5	34,7	
	,110	1	,5	,5	35,3	
	,110	1	,5	,5	35,8	
	,111	1	,5	,5	36,3	
	,112	1	,5	,5	36,8	
	,113	1	,5	,5	37,4	
	,113	1	,5	,5	37,9	
	,114	1	,5	,5	38,4	
	,114	1	,5	,5	38,9	
	,115	1	,5	,5	39,5	
	,115	1	,5	,5	40,0	
	,115	1	,5	,5	40,5	
	,115	1	,5	,5	41,1	
	,117	1	,5	,5	41,6	
	,118	1	,5	,5	42,1	
	,118	1	,5	,5	42,6	
	,118	1	,5	,5	43,2	
	,120	1	,5	,5	43,7	
	,121	1	,5	,5	44,2	
	,123	1	,5	,5	44,7	
	,124	1	,5	,5	45,3	
	,125	1	,5	,5	45,8	
	,126	1	,5	,5	46,3	
	,127	1	,5	,5	46,8	
	,128	1	,5	,5	47,4	
	,130	1	,5	,5	47,9	
	,130	1	,5	,5	48,4	
	,130	1	,5	,5	48,9	
	,131	1	,5	,5	49,5	
	,132	1	,5	,5	50,0	
	,133	1	,5	,5	50,5	
	,133	1	,5	,5	51,1	
	,134	1	,5	,5	51,6	
	,134	1	,5	,5	52,1	
	,135	1	,5	,5	52,6	
	,135	1	,5	,5	53,2	
	,136	1	,5	,5	53,7	
	,138	1	,5	,5	54,2	
	,139	2	1,1	1,1	55,3	
	,141	1	,5	,5	55,8	
	,149	1	,5	,5	56,3	
	,149	1	,5	,5	56,8	
	,150	1	,5	,5	57,4	
	,150	1	,5	,5	57,9	
	,150	1	,5	,5	58,4	
	,150	1	,5	,5	58,9	
	,151	1	,5	,5	59,5	
	,152	1	,5	,5	60,0	
	,152	1	,5	,5	60,5	
	,153	1	,5	,5	61,1	
	,153	1	,5	,5	61,6	
	,154	1	,5	,5	62,1	
	,154	1	,5	,5	62,6	
	,155	1	,5	,5	63,2	
	,156	1	,5	,5	63,7	
	,156	1	,5	,5	64,2	
	,156	1	,5	,5	64,7	
	,159	1	,5	,5	65,3	
	,159	1	,5	,5	65,8	
	,160	1	,5	,5	66,3	
	,161	1	,5	,5	66,8	
	,161	1	,5	,5	67,4	
	,161	1	,5	,5	67,9	
	,162	1	,5	,5	68,4	
	,164	1	,5	,5	68,9	
	,165	1	,5	,5	69,5	
	,166	1	,5	,5	70,0	
	,166	1	,5	,5	70,5	
	,167	1	,5	,5	71,1	
	,168	1	,5	,5	71,6	
	,168	1	,5	,5	72,1	
	,169	1	,5	,5	72,6	
	,170	1	,5	,5	73,2	
	,170	1	,5	,5	73,7	
	,171	1	,5	,5	74,2	
	,172	1	,5	,5	74,7	
	,172	1	,5	,5	75,3	
	,173	1	,5	,5	75,8	
	,174	1	,5	,5	76,3	
	,174	1	,5	,5	76,8	
	,177	1	,5	,5	77,4	
	,178	1	,5	,5	77,9	
	,179	1	,5	,5	78,4	
	,179	1	,5	,5	78,9	
	,182	1	,5	,5	79,5	
	,183	1	,5	,5	80,0	
	,183	1	,5	,5	80,5	
	,184	1	,5	,5	81,1	
	,184	1	,5	,5	81,6	
	,185	1	,5	,5	82,1	
	,185	1	,5	,5	82,6	
	,185	1	,5	,5	83,2	
	,187	1	,5	,5	83,7	
	,187	1	,5	,5	84,2	
	,189	1	,5	,5	84,7	
	,190	1	,5	,5	85,3	
	,190	1	,5	,5	85,8	
	,190	1	,5	,5	86,3	
	,191	1	,5	,5	86,8	
	,191	1	,5	,5	87,4	
	,191	1	,5	,5	87,9	
	,191	1	,5	,5	88,4	
	,191	1	,5	,5	88,9	
	,193	1	,5	,5	89,5	
	,194	1	,5	,5	90,0	
	,194	1	,5	,5	90,5	
	,194	1	,5	,5	91,1	
	,195	1	,5	,5	91,6	
	,196	1	,5	,5	92,1	
	,197	1	,5	,5	92,6	
	,202	1	,5	,5	93,2	
	,202	1	,5	,5	93,7	
	,207	1	,5	,5	94,2	
	,207	1	,5	,5	94,7	
	,209	1	,5	,5	95,3	
	,222	1	,5	,5	95,8	
	,223	1	,5	,5	96,3	
	,223	1	,5	,5	96,8	
	,223	1	,5	,5	97,4	
	,226	1	,5	,5	97,9	
	,238	1	,5	,5	98,4	
	,240	1	,5	,5	98,9	
	,255	1	,5	,5	99,5	
	,269	1	,5	,5	100,0	
	Total	190	100,0	100,0		

DESCRIPTIVES VARIABLES=RCAindex
  /STATISTICS=MEAN STDDEV MIN MAX.


Descriptives


Notes	
Output Created	18-JUL-2021 23:46:16	
Comments		
Input	Data	C:\Users\Placido\Desktop\articolo atrofia e sottodurale cronico\controlli\controlli correlazioni.sav	
	Active Dataset	Dataset1	
	Filter	<none>	
	Weight	<none>	
	Split File	<none>	
	N of Rows in Working Data File	190	
Missing Value Handling	Definition of Missing	User defined missing values are treated as missing.	
	Cases Used	All non-missing data are used.	
Syntax	DESCRIPTIVES VARIABLES=RCAindex
  /STATISTICS=MEAN STDDEV MIN MAX.	
Resources	Processor Time	00:00:00,00	
	Elapsed Time	00:00:00,01	


Descriptive Statistics	
	N	Minimum	Maximum	Mean	Std. Deviation	
RCA index	190	,053	,269	,13716	,045340	
Valid N (listwise)	190					
