# Supplementary material for: Cortical atrophy in chronic subdural hematoma from ultra-structures to physical properties
Source: Sci Rep. 2023 Feb 28;13:3400. doi: 10.1038/s41598-023-30135-8 (PMC9975247; doi:10.1038/s41598-023-30135-8)
Supplement: Supplementary file 11 — Supplementary Information 11. [file 41598_2023_30135_MOESM11_ESM.doc]

Custom Tables


Notes	
Output Created	04-AUG-2021 19:24:33	
Comments		
Input	Data	C:\Users\Placido\Desktop\articolo atrofia e sottodurale cronico\casi\analisi ult.sav	
	Active Dataset	Dataset1	
	Filter	<none>	
	Weight	<none>	
	Split File	<none>	
	N of Rows in Working Data File	190	
Syntax	CTABLES
  /VLABELS VARIABLES=Age Sex Side RCAindex MDPreop MDPost30 MDPost90 Shiftpre Shiftpost30
    Shiftpost90 KPSPreOp KPSPostOp
    DISPLAY=LABEL
  /TABLE Age [MEAN] + Sex [COUNT F40.0, UCOUNT F40.0, ROWPCT.COUNT PCT40.1] + Side [COUNT F40.0,
    UCOUNT F40.0, ROWPCT.COUNT PCT40.1] + RCAindex [MEAN] + MDPreop [MEAN] + MDPost30 [MEAN] + MDPost90
    [MEAN] + Shiftpre [MEAN] + Shiftpost30 [MEAN] + Shiftpost90 [MEAN] + KPSPreOp [MEAN] + KPSPostOp
    [MEAN]
  /CATEGORIES VARIABLES=Sex Side ORDER=A KEY=VALUE EMPTY=EXCLUDE
  /CRITERIA CILEVEL=95
  /TITLES
    TITLE='Characteristics of CSDH Group'
  /COMPARETEST TYPE=MEAN ALPHA=0.05 ADJUST=BONFERRONI ORIGIN=COLUMN INCLUDEMRSETS=YES
    CATEGORIES=ALLVISIBLE MEANSVARIANCE=TESTEDCATS MERGE=YES STYLE=SIMPLE SHOWSIG=NO.	
Resources	Processor Time	00:00:00,02	
	Elapsed Time	00:00:00,01	


Characteristics of CSDH Group	
	Mean	Count	
Age	79		
Sex	f		74	
	m		116	
Side	left		89	
	righ		101	
RCA index	,177		
MDPreop	22,962		
MDPost 30	10,683		
MDPost 90	4,308		
Shift pre	8,966		
Shift post 30	3,313		
Shift post 90	2		
KPS PreOp	58		
KPS PostOp	87		

ONEWAY MDPreop MDPost30 MDPost90 Shiftpre Shiftpost30 Shiftpost90 KPSPreOp KPSPostOp Age BY RCAindex
  /STATISTICS DESCRIPTIVES EFFECTS
  /MISSING ANALYSIS.


Oneway


Notes	
Output Created	04-AUG-2021 19:30:32	
Comments		
Input	Data	C:\Users\Placido\Desktop\articolo atrofia e sottodurale cronico\casi\analisi ult.sav	
	Active Dataset	Dataset1	
	Filter	<none>	
	Weight	<none>	
	Split File	<none>	
	N of Rows in Working Data File	190	
Missing Value Handling	Definition of Missing	User-defined missing values are treated as missing.	
	Cases Used	Statistics for each analysis are based on cases with no missing data for any variable in the analysis.	
Syntax	ONEWAY MDPreop MDPost30 MDPost90 Shiftpre Shiftpost30 Shiftpost90 KPSPreOp KPSPostOp Age BY RCAindex
  /STATISTICS DESCRIPTIVES EFFECTS
  /MISSING ANALYSIS.	
Resources	Processor Time	00:00:00,08	
	Elapsed Time	00:00:00,05	


ANOVA	
	Sum of Squares	df	Mean Square	F	Sig.	
MDPreop	Between Groups	5464,227	69	79,192	.	.	
	Within Groups	,000	120	,000			
	Total	5464,227	189				
MDPost 30	Between Groups	5331,706	69	77,271	74,678	,000	
	Within Groups	124,167	120	1,035			
	Total	5455,873	189				
MDPost 90	Between Groups	1278,709	69	18,532	3,832	,000	
	Within Groups	580,374	120	4,836			
	Total	1859,082	189				
Shift pre	Between Groups	2481,228	69	35,960	.	.	
	Within Groups	,000	120	,000			
	Total	2481,228	189				
Shift post 30	Between Groups	1186,010	69	17,189	375,023	,000	
	Within Groups	5,500	120	,046			
	Total	1191,510	189				
Shift post 90	Between Groups	130,216	69	1,887	1,272	,124	
	Within Groups	178,000	120	1,483			
	Total	308,216	189				
KPS PreOp	Between Groups	39255,263	69	568,917	.	.	
	Within Groups	,000	120	,000			
	Total	39255,263	189				
KPS PostOp	Between Groups	16844,211	69	244,119	.	.	
	Within Groups	,000	120	,000			
	Total	16844,211	189				
Age	Between Groups	11034,863	69	159,926	.	.	
	Within Groups	,000	120	,000			
	Total	11034,863	189				

CORRELATIONS
  /VARIABLES=Age RCAindex MDPreop MDPost30 MDPost90 Shiftpre Shiftpost30 Shiftpost90 KPSPreOp
    KPSPostOp
  /PRINT=TWOTAIL NOSIG
  /STATISTICS DESCRIPTIVES
  /MISSING=PAIRWISE.


Correlations


Notes	
Output Created	04-AUG-2021 19:35:52	
Comments		
Input	Data	C:\Users\Placido\Desktop\articolo atrofia e sottodurale cronico\casi\analisi ult.sav	
	Active Dataset	Dataset1	
	Filter	<none>	
	Weight	<none>	
	Split File	<none>	
	N of Rows in Working Data File	190	
Missing Value Handling	Definition of Missing	User-defined missing values are treated as missing.	
	Cases Used	Statistics for each pair of variables are based on all the cases with valid data for that pair.	
Syntax	CORRELATIONS
  /VARIABLES=Age RCAindex MDPreop MDPost30 MDPost90 Shiftpre Shiftpost30 Shiftpost90 KPSPreOp
    KPSPostOp
  /PRINT=TWOTAIL NOSIG
  /STATISTICS DESCRIPTIVES
  /MISSING=PAIRWISE.	
Resources	Processor Time	00:00:00,02	
	Elapsed Time	00:00:00,02	


Descriptive Statistics of CSDH Group	
	Mean	Std. Deviation	N	
Age	78,56	7,641	190	
RCA index	,17691	,034633	190	
MDPreop	22,96211	5,376919	190	
MDPost 30	10,68263	5,372807	190	
MDPost 90	4,30832	3,136306	190	
Shift pre	8,96579	3,623284	190	
Shift post 30	3,31263	2,510833	190	
Shift post 90	1,88	1,277	190	
KPS PreOp	58,16	14,412	190	
KPS PostOp	86,63	9,440	190	


Correlations	
	Age	RCA index	MDPreop	MDPost 30	MDPost 90	
Age	Pearson Correlation	1	,512	,157	,261	,142	
	Sig. (2-tailed)		,000	,030	,000	,051	
	N	190	190	190	190	190	
RCA index	Pearson Correlation	,512	1	,286	,283	,059	
	Sig. (2-tailed)	,000		,000	,000	,419	
	N	190	190	190	190	190	
MDPreop	Pearson Correlation	,157	,286	1	,437	,090	
	Sig. (2-tailed)	,030	,000		,000	,216	
	N	190	190	190	190	190	
MDPost 30	Pearson Correlation	,261	,283	,437	1	,395	
	Sig. (2-tailed)	,000	,000	,000		,000	
	N	190	190	190	190	190	
MDPost 90	Pearson Correlation	,142	,059	,090	,395	1	
	Sig. (2-tailed)	,051	,419	,216	,000		
	N	190	190	190	190	190	
Shift pre	Pearson Correlation	-,224	,029	,177	-,059	-,026	
	Sig. (2-tailed)	,002	,686	,015	,421	,725	
	N	190	190	190	190	190	
Shift post 30	Pearson Correlation	-,129	-,090	,152	,348	,177	
	Sig. (2-tailed)	,075	,214	,037	,000	,014	
	N	190	190	190	190	190	
Shift post 90	Pearson Correlation	-,012	,026	-,079	,166	,133	
	Sig. (2-tailed)	,869	,723	,280	,022	,068	
	N	190	190	190	190	190	
KPS PreOp	Pearson Correlation	,037	-,255	-,155	-,071	,090	
	Sig. (2-tailed)	,610	,000	,033	,334	,219	
	N	190	190	190	190	190	
KPS PostOp	Pearson Correlation	-,334	-,333	-,073	-,212	-,117	
	Sig. (2-tailed)	,000	,000	,320	,003	,108	
	N	190	190	190	190	190	

Correlations	
	Shift pre	Shift post 30	Shift post 90	KPS PreOp	
Age	Pearson Correlation	-,224	-,129	-,012	,037	
	Sig. (2-tailed)	,002	,075	,869	,610	
	N	190	190	190	190	
RCA index	Pearson Correlation	,029	-,090	,026	-,255	
	Sig. (2-tailed)	,686	,214	,723	,000	
	N	190	190	190	190	
MDPreop	Pearson Correlation	,177	,152	-,079	-,155	
	Sig. (2-tailed)	,015	,037	,280	,033	
	N	190	190	190	190	
MDPost 30	Pearson Correlation	-,059	,348	,166	-,071	
	Sig. (2-tailed)	,421	,000	,022	,334	
	N	190	190	190	190	
MDPost 90	Pearson Correlation	-,026	,177	,133	,090	
	Sig. (2-tailed)	,725	,014	,068	,219	
	N	190	190	190	190	
Shift pre	Pearson Correlation	1	,565	,149	-,226	
	Sig. (2-tailed)		,000	,040	,002	
	N	190	190	190	190	
Shift post 30	Pearson Correlation	,565	1	,253	-,096	
	Sig. (2-tailed)	,000		,000	,187	
	N	190	190	190	190	
Shift post 90	Pearson Correlation	,149	,253	1	-,044	
	Sig. (2-tailed)	,040	,000		,548	
	N	190	190	190	190	
KPS PreOp	Pearson Correlation	-,226	-,096	-,044	1	
	Sig. (2-tailed)	,002	,187	,548		
	N	190	190	190	190	
KPS PostOp	Pearson Correlation	,051	-,046	,010	,417	
	Sig. (2-tailed)	,485	,531	,892	,000	
	N	190	190	190	190	

Correlations	
	KPS PostOp	
Age	Pearson Correlation	-,334	
	Sig. (2-tailed)	,000	
	N	190	
RCA index	Pearson Correlation	-,333	
	Sig. (2-tailed)	,000	
	N	190	
MDPreop	Pearson Correlation	-,073	
	Sig. (2-tailed)	,320	
	N	190	
MDPost 30	Pearson Correlation	-,212	
	Sig. (2-tailed)	,003	
	N	190	
MDPost 90	Pearson Correlation	-,117	
	Sig. (2-tailed)	,108	
	N	190	
Shift pre	Pearson Correlation	,051	
	Sig. (2-tailed)	,485	
	N	190	
Shift post 30	Pearson Correlation	-,046	
	Sig. (2-tailed)	,531	
	N	190	
Shift post 90	Pearson Correlation	,010	
	Sig. (2-tailed)	,892	
	N	190	
KPS PreOp	Pearson Correlation	,417	
	Sig. (2-tailed)	,000	
	N	190	
KPS PostOp	Pearson Correlation	1	
	Sig. (2-tailed)		
	N	190	

NONPAR CORR
  /VARIABLES=Age RCAindex MDPreop MDPost30 MDPost90 Shiftpre Shiftpost30 Shiftpost90 KPSPreOp
    KPSPostOp
  /PRINT=BOTH TWOTAIL NOSIG
  /MISSING=PAIRWISE.


Nonparametric Correlations


Notes	
Output Created	04-AUG-2021 19:35:52	
Comments		
Input	Data	C:\Users\Placido\Desktop\articolo atrofia e sottodurale cronico\casi\analisi ult.sav	
	Active Dataset	Dataset1	
	Filter	<none>	
	Weight	<none>	
	Split File	<none>	
	N of Rows in Working Data File	190	
Missing Value Handling	Definition of Missing	User-defined missing values are treated as missing.	
	Cases Used	Statistics for each pair of variables are based on all the cases with valid data for that pair.	
Syntax	NONPAR CORR
  /VARIABLES=Age RCAindex MDPreop MDPost30 MDPost90 Shiftpre Shiftpost30 Shiftpost90 KPSPreOp
    KPSPostOp
  /PRINT=BOTH TWOTAIL NOSIG
  /MISSING=PAIRWISE.	
Resources	Processor Time	00:00:00,02	
	Elapsed Time	00:00:00,03	
	Number of Cases Allowed	241979 cases	


Correlations	
	Age	RCA index	MDPreop	MDPost 30	MDPost 90	Shift pre	Shift post 30	Shift post 90	KPS PreOp	KPS PostOp	
Kendall's tau_b	Age	Correlation Coefficient	1,000	,388	,085	,143	,099	-,091	-,034	-,001	,077	-,266	
		Sig. (2-tailed)	.	,000	,098	,005	,059	,076	,514	,989	,172	,000	
		N	190	190	190	190	190	190	190	190	190	190	
	RCA index	Correlation Coefficient	,388	1,000	,210	,160	,046	,031	-,062	,018	-,146	-,223	
		Sig. (2-tailed)	,000	.	,000	,001	,367	,535	,224	,737	,008	,000	
		N	190	190	190	190	190	190	190	190	190	190	
	MDPreop	Correlation Coefficient	,085	,210	1,000	,367	,090	,160	,127	-,037	-,066	-,076	
		Sig. (2-tailed)	,098	,000	.	,000	,083	,002	,014	,506	,242	,185	
		N	190	190	190	190	190	190	190	190	190	190	
	MDPost 30	Correlation Coefficient	,143	,160	,367	1,000	,335	-,039	,226	,128	,014	-,235	
		Sig. (2-tailed)	,005	,001	,000	.	,000	,448	,000	,022	,807	,000	
		N	190	190	190	190	190	190	190	190	190	190	
	MDPost 90	Correlation Coefficient	,099	,046	,090	,335	1,000	,041	,197	,115	,017	-,164	
		Sig. (2-tailed)	,059	,367	,083	,000	.	,435	,000	,044	,762	,005	
		N	190	190	190	190	190	190	190	190	190	190	
	Shift pre	Correlation Coefficient	-,091	,031	,160	-,039	,041	1,000	,432	,127	-,144	,021	
		Sig. (2-tailed)	,076	,535	,002	,448	,435	.	,000	,024	,011	,709	
		N	190	190	190	190	190	190	190	190	190	190	
	Shift post 30	Correlation Coefficient	-,034	-,062	,127	,226	,197	,432	1,000	,196	-,030	-,142	
		Sig. (2-tailed)	,514	,224	,014	,000	,000	,000	.	,001	,604	,015	
		N	190	190	190	190	190	190	190	190	190	190	
	Shift post 90	Correlation Coefficient	-,001	,018	-,037	,128	,115	,127	,196	1,000	-,061	-,062	
		Sig. (2-tailed)	,989	,737	,506	,022	,044	,024	,001	.	,324	,322	
		N	190	190	190	190	190	190	190	190	190	190	
	KPS PreOp	Correlation Coefficient	,077	-,146	-,066	,014	,017	-,144	-,030	-,061	1,000	,380	
		Sig. (2-tailed)	,172	,008	,242	,807	,762	,011	,604	,324	.	,000	
		N	190	190	190	190	190	190	190	190	190	190	
	KPS PostOp	Correlation Coefficient	-,266	-,223	-,076	-,235	-,164	,021	-,142	-,062	,380	1,000	
		Sig. (2-tailed)	,000	,000	,185	,000	,005	,709	,015	,322	,000	.	
		N	190	190	190	190	190	190	190	190	190	190	
Spearman's rho	Age	Correlation Coefficient	1,000	,535	,126	,200	,138	-,126	-,036	,000	,094	-,330	
		Sig. (2-tailed)	.	,000	,082	,006	,058	,084	,620	,996	,197	,000	
		N	190	190	190	190	190	190	190	190	190	190	
	RCA index	Correlation Coefficient	,535	1,000	,301	,240	,066	,060	-,082	,024	-,189	-,298	
		Sig. (2-tailed)	,000	.	,000	,001	,365	,409	,261	,744	,009	,000	
		N	190	190	190	190	190	190	190	190	190	190	
	MDPreop	Correlation Coefficient	,126	,301	1,000	,493	,117	,225	,178	-,049	-,087	-,091	
		Sig. (2-tailed)	,082	,000	.	,000	,108	,002	,014	,504	,231	,212	
		N	190	190	190	190	190	190	190	190	190	190	
	MDPost 30	Correlation Coefficient	,200	,240	,493	1,000	,433	-,050	,296	,170	,018	-,301	
		Sig. (2-tailed)	,006	,001	,000	.	,000	,492	,000	,019	,807	,000	
		N	190	190	190	190	190	190	190	190	190	190	
	MDPost 90	Correlation Coefficient	,138	,066	,117	,433	1,000	,055	,261	,149	,024	-,204	
		Sig. (2-tailed)	,058	,365	,108	,000	.	,454	,000	,041	,747	,005	
		N	190	190	190	190	190	190	190	190	190	190	
	Shift pre	Correlation Coefficient	-,126	,060	,225	-,050	,055	1,000	,570	,163	-,176	,023	
		Sig. (2-tailed)	,084	,409	,002	,492	,454	.	,000	,024	,015	,750	
		N	190	190	190	190	190	190	190	190	190	190	
	Shift post 30	Correlation Coefficient	-,036	-,082	,178	,296	,261	,570	1,000	,241	-,042	-,172	
		Sig. (2-tailed)	,620	,261	,014	,000	,000	,000	.	,001	,568	,018	
		N	190	190	190	190	190	190	190	190	190	190	
	Shift post 90	Correlation Coefficient	,000	,024	-,049	,170	,149	,163	,241	1,000	-,071	-,072	
		Sig. (2-tailed)	,996	,744	,504	,019	,041	,024	,001	.	,329	,324	
		N	190	190	190	190	190	190	190	190	190	190	
	KPS PreOp	Correlation Coefficient	,094	-,189	-,087	,018	,024	-,176	-,042	-,071	1,000	,425	
		Sig. (2-tailed)	,197	,009	,231	,807	,747	,015	,568	,329	.	,000	
		N	190	190	190	190	190	190	190	190	190	190	
	KPS PostOp	Correlation Coefficient	-,330	-,298	-,091	-,301	-,204	,023	-,172	-,072	,425	1,000	
		Sig. (2-tailed)	,000	,000	,212	,000	,005	,750	,018	,324	,000	.	
		N	190	190	190	190	190	190	190	190	190	190	

REGRESSION
  /MISSING LISTWISE
  /STATISTICS COEFF OUTS R ANOVA
  /CRITERIA=PIN(.05) POUT(.10)
  /NOORIGIN
  /DEPENDENT RCAindex
  /METHOD=ENTER Age MDPreop MDPost30 MDPost90 Shiftpre Shiftpost30 Shiftpost90 KPSPreOp KPSPostOp
  /SCATTERPLOT=(*ZPRED ,*ZRESID)
  /RESIDUALS HISTOGRAM(ZRESID) NORMPROB(ZRESID).


Regression


Notes	
Output Created	04-AUG-2021 19:44:54	
Comments		
Input	Data	C:\Users\Placido\Desktop\articolo atrofia e sottodurale cronico\casi\analisi ult.sav	
	Active Dataset	Dataset1	
	Filter	<none>	
	Weight	<none>	
	Split File	<none>	
	N of Rows in Working Data File	190	
Missing Value Handling	Definition of Missing	User-defined missing values are treated as missing.	
	Cases Used	Statistics are based on cases with no missing values for any variable used.	
Syntax	REGRESSION
  /MISSING LISTWISE
  /STATISTICS COEFF OUTS R ANOVA
  /CRITERIA=PIN(.05) POUT(.10)
  /NOORIGIN
  /DEPENDENT RCAindex
  /METHOD=ENTER Age MDPreop MDPost30 MDPost90 Shiftpre Shiftpost30 Shiftpost90 KPSPreOp KPSPostOp
  /SCATTERPLOT=(*ZPRED ,*ZRESID)
  /RESIDUALS HISTOGRAM(ZRESID) NORMPROB(ZRESID).	
Resources	Processor Time	00:00:03,58	
	Elapsed Time	00:00:35,45	
	Memory Required	8624 bytes	
	Additional Memory Required for Residual Plots	552 bytes	


Variables Entered/Removed	
Model	Variables Entered	Variables Removed	Method	
1	KPS PostOp, Shift post 90, MDPreop, MDPost 90, Shift pre , Age, KPS PreOp, MDPost 30 , Shift post 30	.	Enter	


Model Summary	
Model	R	R Square	Adjusted R Square	Std. Error of the Estimate	
1	,648	,420	,391	,027029	


ANOVA	
Model	Sum of Squares	df	Mean Square	F	Sig.	
1	Regression	,095	9	,011	14,479	,000	
	Residual	,131	180	,001			
	Total	,227	189				


Coefficients	
Model	Unstandardized Coefficients	Standardized Coefficients	t	Sig.	
	B	Std. Error	Beta			
1	(Constant)	,036	,035		1,032	,303	
	Age	,002	,000	,440	6,757	,000	
	MDPreop	,001	,000	,094	1,378	,170	
	MDPost 30	,001	,001	,220	2,733	,007	
	MDPost 90	,000	,001	-,042	-,670	,503	
	Shift pre	,002	,001	,246	3,118	,002	
	Shift post 30	-,004	,001	-,286	-3,544	,001	
	Shift post 90	,001	,002	,037	,609	,543	
	KPS PreOp	,000	,000	-,169	-2,512	,013	
	KPS PostOp	,000	,000	-,092	-1,333	,184	


Residuals Statistics	
	Minimum	Maximum	Mean	Std. Deviation	N	
Predicted Value	,12316	,24943	,17691	,022443	190	
Residual	-,060993	,065492	,000000	,026377	190	
Std. Predicted Value	-2,395	3,231	,000	1,000	190	
Std. Residual	-2,257	2,423	,000	,976	190	


Charts
