# Supplementary material for: Cortical atrophy in chronic subdural hematoma from ultra-structures to physical properties
Source: Sci Rep. 2023 Feb 28;13:3400. doi: 10.1038/s41598-023-30135-8 (PMC9975247; doi:10.1038/s41598-023-30135-8)
Supplement: Supplementary file 11 [file 41598_2023_30135_MOESM6_ESM.doc]

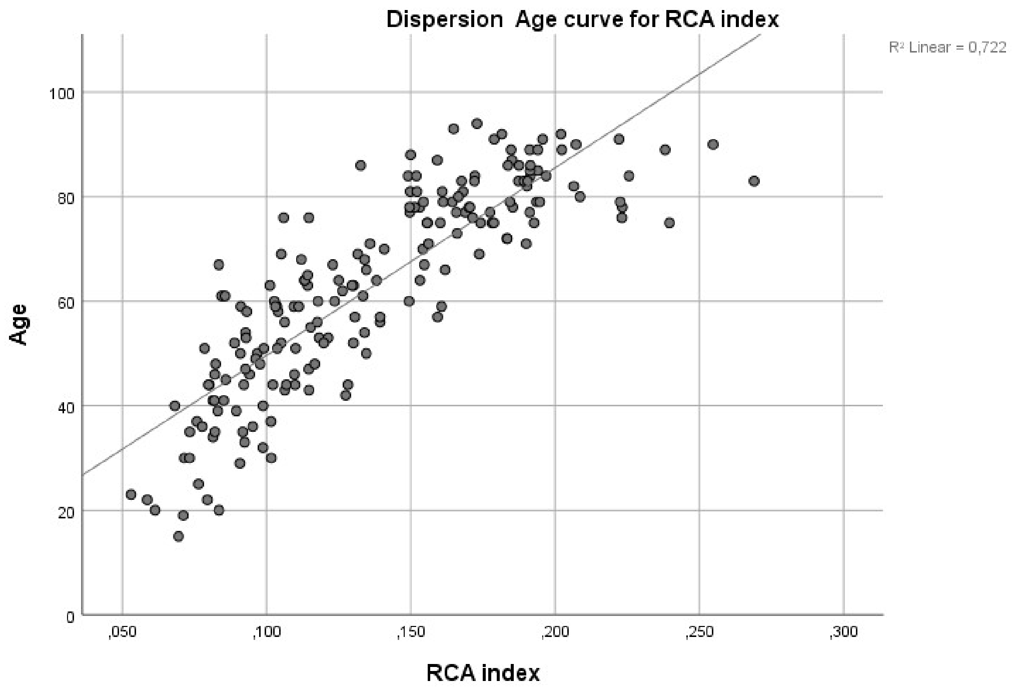

Dispersion Age curve for RCA index
R² Linear = 0,722
Age
100
80
60
40
20
0
,050
,100
,150
,200
,250
,300
RCA index
